# Correction: Determining optimal timing of birth for women with chronic or gestational hypertension at term: The WILL (When to Induce Labour to Limit risk in pregnancy hypertension) randomised trial

**DOI:** 10.1371/journal.pmed.1004627

**Published:** 2025-05-22

**Authors:** Laura A. Magee, Katie Kirkham, Sue Tohill, Eleni Gkini, Catherine A. Moakes, Jon Dorling, Marcus Green, Jennifer A. Hutcheon, Mishal Javed, Jesse Kigozi, Ben W. M. Mol, Joel Singer, Pollyanna Hardy, Clive Stubbs, James G. Thornton, Peter von Dadelszen, for the WILL Trial Study Group

S3 Appendix is uploaded incorrectly. Please see the correct S3 Appendix here.

In the Outcomes subsection of the Results, there is an error in the first sentence of the fifth paragraph. The correct sentence is: For the woman, there was an association between the intervention (versus control) and preeclampsia (56/201 [28%] versus 76/202 [38%], respectively; aRR 0.74, 95% CI 0.56 to 0.98; *p* = 0.039; Table 3), before and after birth; the absolute reduction translates into a number-needed-to-treat-for-benefit (NNTB) of 10.

## Supporting information

S3 Appendix Tables A-K. (DOCX)
