# Supplementary material for: Determining optimal timing of birth for women with chronic or gestational hypertension at term: The WILL (When to Induce Labour to Limit risk in pregnancy hypertension) randomised trial
Source: PLoS Med. 2024 Nov 26;21(11):e1004481. doi: 10.1371/journal.pmed.1004481 (PMC11593758; doi:10.1371/journal.pmed.1004481)
Supplement: S1 Consort Checklist — (DOCX) [file pmed.1004481.s001.docx]

**Supplementary Appendix B**

| Document | Page number |
| --- | --- |
| CONSORT 2010-Checklist | 2 |
| CONSERVE-CONSORT Checklist | 5 |


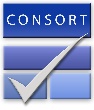
CONSORT 2010 checklist of information to include when reporting a randomised trial*

| Section/Topic | Item No | Checklist item | Reported on page No |
| --- | --- | --- | --- |
| Title and abstract | | | |
|  | 1a | Identification as a randomised trial in the title | Title |
|  | 1b | Structured summary of trial design, methods, results, and conclusions (for specific guidance see CONSORT for abstracts) | Abstract |
| Introduction | | | |
| Background and objectives | 2a | Scientific background and explanation of rationale | Introduction, 1^st^ and 2^nd^ paragraphs |
|  | 2b | Specific objectives or hypotheses | Introduction, 3^rd^ paragraph |
| Methods | | | |
| Trial design | 3a | Description of trial design (such as parallel, factorial) including allocation ratio | Methods/Study design and participants, 1^st^ and 2^nd^ paragraphs |
|  | 3b | Important changes to methods after trial commencement (such as eligibility criteria), with reasons | Methods/Procedures  Results, 1^st^ paragraph |
| Participants | 4a | Eligibility criteria for participants | Methods/Study design and participants, 2^nd^ paragraph |
|  | 4b | Settings and locations where the data were collected | Methods/Study design and participants, 2^nd^ paragraph |
| Interventions | 5 | The interventions for each group with sufficient details to allow replication, including how and when they were actually administered | Methods/Randomisation and masking, 1^st^ paragraph  Methods/Procedures, 1^st^ and 3^rd^ paragraphs |
| Outcomes | 6a | Completely defined pre-specified primary and secondary outcome measures, including how and when they were assessed | Methods/Outcomes, 2^nd^ to 4^th^ paragraphs |
|  | 6b | Any changes to trial outcomes after the trial commenced, with reasons | N/A |
| Sample size | 7a | How sample size was determined | Methods/Statistical analysis, 1^st^ paragraph |
|  | 7b | When applicable, explanation of any interim analyses and stopping guidelines | N/A |
| Randomisation: |  |  |  |
| Sequence generation | 8a | Method used to generate the random allocation sequence | Methods/Randomisation and masking, 1^st^ paragraph |
|  | 8b | Type of randomisation; details of any restriction (such as blocking and block size) | Methods/Randomisation and masking, 1^st^ paragraph |
| Allocation concealment mechanism | 9 | Mechanism used to implement the random allocation sequence (such as sequentially numbered containers), describing any steps taken to conceal the sequence until interventions were assigned | Methods/Randomisation and masking, 1^st^ paragraph |
| Implementation | 10 | Who generated the random allocation sequence, who enrolled participants, and who assigned participants to interventions | Methods/Randomisation and masking, 1^st^ paragraph |
| Blinding | 11a | If done, who was blinded after assignment to interventions (for example, participants, care providers, those assessing outcomes) and how | N/A |
|  | 11b | If relevant, description of the similarity of interventions | N/A |
| Statistical methods | 12a | Statistical methods used to compare groups for primary and secondary outcomes | Methods/Statistical analysis, 2^nd^ paragraph |
|  | 12b | Methods for additional analyses, such as subgroup analyses and adjusted analyses | Methods/Statistical analysis, 3^rd^ to 5^th^ paragraphs |
| Results | | | |
| Participant flow (a diagram is strongly recommended) | 13a | For each group, the numbers of participants who were randomly assigned, received intended treatment, and were analysed for the primary outcome | Results, 2^nd^ paragraph  Fig 1 |
|  | 13b | For each group, losses and exclusions after randomisation, together with reasons | Fig 1 |
| Recruitment | 14a | Dates defining the periods of recruitment and follow-up | Results, 1^st^ paragraph |
|  | 14b | Why the trial ended or was stopped | Results, 1^st^ paragraph |
| Baseline data | 15 | A table showing baseline demographic and clinical characteristics for each group | Table 1 |
| Numbers analysed | 16 | For each group, number of participants (denominator) included in each analysis and whether the analysis was by original assigned groups | Fig 1 |
| Outcomes and estimation | 17a | For each primary and secondary outcome, results for each group, and the estimated effect size and its precision (such as 95% confidence interval) | Tables 2-4 |
|  | 17b | For binary outcomes, presentation of both absolute and relative effect sizes is recommended | Tables 2-4 |
| Ancillary analyses | 18 | Results of any other analyses performed, including subgroup analyses and adjusted analyses, distinguishing pre-specified from exploratory | S3 Appendix |
| Harms | 19 | All important harms or unintended effects in each group (for specific guidance see CONSORT for harms) | N/A |
| Discussion | | | |
| Limitations | 20 | Trial limitations, addressing sources of potential bias, imprecision, and, if relevant, multiplicity of analyses | Discussion/Strengths and limitations, 2^nd^ paragraph |
| Generalisability | 21 | Generalisability (external validity, applicability) of the trial findings | Discussion/Strengths and limitations, 2^nd^ paragraph |
| Interpretation | 22 | Interpretation consistent with results, balancing benefits and harms, and considering other relevant evidence | Discussion/Interpretation, 1^st^ paragraph |
| Other information | | |  |
| Registration | 23 | Registration number and name of trial registry | Abstract  Methods/Study design and participants, 1^st^ paragraph |
| Protocol | 24 | Where the full trial protocol can be accessed, if available | Methods/Study design and participants, 1^st^ paragraph |
| Funding | 25 | Sources of funding and other support (such as supply of drugs), role of funders | Abstract  Funding statement |

Citation: Schulz KF, Altman DG, Moher D, for the CONSORT Group. CONSORT 2010 Statement: updated guidelines for reporting parallel group randomised trials. BMC Medicine. 2010;8:18.
© 2010 Schulz et al. This is an Open Access article distributed under the terms of the Creative Commons Attribution License (<http://creativecommons.org/licenses/by/2.0>), which permits unrestricted use, distribution, and reproduction in any medium, provided the original work is properly cited.

*We strongly recommend reading this statement in conjunction with the CONSORT 2010 Explanation and Elaboration for important clarifications on all the items. If relevant, we also recommend reading CONSORT extensions for cluster randomised trials, non-inferiority and equivalence trials, non-pharmacological treatments, herbal interventions, and pragmatic trials. Additional extensions are forthcoming: for those and for up-to-date references relevant to this checklist, see [www.consort-statement.org](http://www.consort-statement.org).

# **CONSERVE Checklists**

| CONSERVE-CONSORT Extension: [25 June 2024] | | | | | |
| --- | --- | --- | --- | --- | --- |
| Item | Item Title | Description | | | Section & Line No. |
| I. | Extenuating Circumstances | The control arm of the trial was changed due to temporal changes in clinical practice, particularly associated with the COVID-19 pandemic. | | | Methods/ Procedures, 1^st^ paragraph |
|  |  | The trial was stopped early by the funder as part of ‘post-pandemic reset’, due to slower-than-anticipated recruitment. | | | Results, 1^st^ 1^st^ paragraph |
| II. | Important Modifications | 1. The nature of the control arm is critical for evaluation of the intervention.   Early termination of the trial meant that we did not achieve our target sample size. | | | Results, 1^st^ and 2^nd^ paragraphs |
|  |  | 1. Sensitivity analysis was planned to assess heterogeneity of treatment effect due to the change to usual care (control arm).   We evaluated whether the pre-specified difference in the co-primary and key secondary outcomes was included in the 95% CI for the treatment effect. | | | Methods/Statistical analysis, 4^th^ paragraph |
|  |  | 1. On 11/August/2022 (after randomisation of 348 women), the control group was changed from ‘expectant care until at least 40+0 weeks’ to ‘usual care at term’, for the reasons stated above,   Trial recruitment began on 03/June/2019 and finished on 19/December/2022, for the reasons stated above, | | | Results, 1^st^ paragraph |
| III. | Responsible Parties | The change in control arm was approved by the Trial Oversight Committees (i.e., Management, Steering, and Data Monitoring), and the relevant Research Ethics Board.  The funder (NIHR, UK) withdrew funding for recruitment independently, without knowledge of the trial results. | | | Results, 1^st^ paragraph |
| IV. | Interim data | If modifications were informed by trial data, describe how the interim data were used, including whether they were examined by study group, and whether the individuals reviewing the data were blinded to the treatment allocation. | | | Not applicable |
| CONSORT Number and Item | | For each row, if important modifications occurred check “direct impact” and/or “mitigating strategy” and describe the changes in the trial manuscript or supplement. Check “no change” for items that are unaffected in the extenuating circumstance. | | | Page No. |
|  |  | No Change | Impact* | Mitigating Strategy** |  |
| 1 | Title and abstract | - | Reduced sample size | X (see IIb) | Title  Abstract |
| 2 | Introduction | X | - | - | - |
| 3 | Methods: Trial Design | X (for early termination of trial) | X (for change in control group) | Changing the control arm was a mitigating strategy to deal with a change in current practice. | N/A |
| 4 | Methods: Participants | X (for early termination of trial, as all recruits completed the trial as planned) | X (for change in control group) | The protocol and patient-facing materials were revised (and approved), accordingly. | Methods/Study design and participants1^st^ paragraph |
| 5 | Methods: Interventions | X (for early termination of trial) | Control arm changed to ‘usual care at term’ from ‘expectant care until at least 40+0 weeks’ gestation’. | Not applicable, as the change was a mitigating strategy to deal with contemporaneous changes in practice. | Methods/ Procedures, 1^st^ paragraph |
| 6 | Methods: Outcomes | X (for early termination of trial and change in control group) | - | - | - |
| 7 | Methods: Sample Size | X (for early termination of trial and change in control group) | - | - | - |
| 8-10 | Methods: Randomisation | X (for early termination of trial and change in control group) | - | - | - |
| 11 | Methods: Blinding | X (for early termination of trial and change in control group) | - | - | - |
| 12 | Methods: Statistical methods | - | Smaller-than-anticipated sample size | Sensitivity analysis was planned to assess heterogeneity of treatment effect due to the change to usual care (control arm).  We evaluated whether the pre-specified difference in the co-primary and key secondary outcomes was included in the 95% CI for the treatment effect | Methods/ Statistics, 3^rd^ paragraph  Results/ Outcomes, 1^st^, 2^nd^, and 4^th^ paragraphs |
| 13 | Results: Participant flow | X (for early termination of trial and change in control group) | - | - | - |
| 14 | Results: Recruitment | - | Smaller-than-anticipated sample size overall, but improved recruitment prior to that point. | Not applicable | - |
| 15 | Results: Baseline data | X (for early termination of trial and change in control group) | - | - | - |
| 16 | Results: Numbers analysed | - | Smaller-than-anticipated sample size. | We evaluated whether the pre-specified difference in the co-primary and key secondary outcomes was included in the 95% CI for the treatment effect | Results/ Outcomes, 1st, 2^nd^, and 4^th^ paragraphs |
| 17 | Results: Outcomes and estimation | - | Less precise estimates of treatment effect | We evaluated whether the pre-specified difference in the co-primary and key secondary outcomes was included in the 95% CI for the treatment effect | Results/ Outcomes, 1st, 2^nd^, and 4^th^ paragraphs |
| 18 | Results: Ancillary analyses | - | Additional considerations | Sensitivity analysis was planned to assess heterogeneity of treatment effect due to the change to usual care (control arm).  We evaluated whether the pre-specified difference in the co-primary and key secondary outcomes was included in the 95% CI for the treatment effect | Methods/ Statistics, 3^rd^ paragraph  Results/ Outcomes, 1^st^, 2^nd^, and 4^th^ paragraphs |
| 19 | Results: Harms | X | - | - | - |
| 20 | Discussion: Limitations | - | Smaller-than-anticipated sample size | Examination of 95% CI relative to pre-specified differences | Results/ Outcomes, 1st, 2^nd^, and 4^th^ paragraphs |
| 21 | Discussion: Generalisability | X | - | - | - |
| 23 | Other information: Registration | X | - | - | - |
| 24 | Other information: Protocol | - | Revised as relevant (see section I) | Not applicable | - |
| 25 | Other information: Funding | - | Total funding marginally reduced | Funder’s decision was made independently | Results, 1^st^ paragraph |
| *Aspects of the trial that are directly affected or changed by the extenuating circumstance and are not under the control of investigators, sponsor or funder.  **Aspects of the trial that are modified by the study investigators, sponsor or funder to respond to the extenuating circumstance or manage the direct impacts on the trial. | | | | | |
