# Supplementary material for: Determining optimal timing of birth for women with chronic or gestational hypertension at term: The WILL (When to Induce Labour to Limit risk in pregnancy hypertension) randomised trial
Source: PLoS Med. 2024 Nov 26;21(11):e1004481. doi: 10.1371/journal.pmed.1004481 (PMC11593758; doi:10.1371/journal.pmed.1004481)
Supplement: S1 Protocol — (PDF) [file pmed.1004481.s002.pdf]

TRIAL PROTOCOL

WILL

When to Induce Labour to Limit risk in pregnancy hypertension – a multicentre, randomised controlled trial

Version Number: 4.0

25th May 2022

Funding and Support in Kind	
**Funder (s)**	**Financial and non-financial support given:**
National Institute for Health Research (NIHR) – Health Technology Assessment (HTA) programme	£1,849,961.60 (5 year, 8 months)
Funding Scheme (if applicable)	HTA (Health Technology Assessment) Programme
Funder’s reference number	16/167/123
This Protocol presents independent research funded by the NIHR. The views expressed are those of the author(s) and not necessarily those of the National Health Service (NHS), the NIHR or the Department of Health.	

CI Signature Page

The undersigned confirm that the following protocol has been agreed and accepted and that the Chief Investigator agrees to conduct the trial in compliance with the approved protocol.

I agree to ensure that the confidential information contained in this document will not be used for any other purpose other than the evaluation or conduct of the clinical investigation without the prior written consent of the Sponsor.

I also confirm that I will make the findings of the study publicly available through publication or other dissemination tools without any unnecessary delay and that an honest accurate and transparent account of the study will be given; and that any discrepancies from the study as planned in this protocol will be explained.

This protocol has been approved by:

Trial Name:	WILL (When to Induce Labour to Limit risk in pregnancy hypertension) – a multicentre randomised controlled trial
Protocol Version Number:	Version: 4.0
Protocol Version Date:	25th May 2022
CI Name:	Laura A. Magee
Trial Role:	Chief Investigator
Signature and date:	07/07/2022

Sponsor statement:

By signing the IRAS form for this trial, King's College London and Guy's and St. Thomas' NHS Foundation Trust, acting as co-sponsors of this trial confirm approval of this protocol.

**Reference Numbers**	
Sponsor reference number	IRAS 252294
ISRCTN reference number	ISRCTN 77258279
IRAS reference number	252294

PI Signature Page	
The undersigned confirm that the following protocol has been agreed and accepted and that the Principal Investigator agrees to conduct the trial in compliance with the approved protocol.<br><br>I agree to ensure that the confidential information contained in this document will not be used for any other purpose other than the evaluation or conduct of the clinical investigation without the prior written consent of the Sponsor.	
Trial Name:	WILL (When to Induce Labour to Limit risk in pregnancy hypertension) – a multicentre randomised controlled trial
Protocol Version Number:	Version: 4.0
Protocol Version Date:	<date>
PI Name:	
Name of Site:	
Signature and date:	______________________ __ __/ __ __ __/ __ __ __ __

Administrative Information

**Sponsor Representative**	
King's College London*	Professor Reza Razavi
Contact Details:	Vice President & Vice Principal (Research)<br>King's College London<br>Room 5.31, James Clerk Maxwell Building<br>57 Waterloo Road<br>London SE1 8WA<br>Tel: +44 (0)207 8483224<br>Email: reza.razavi@kcl.ac.uk
Guys and St Thomas' NHS Foundation Trust	Rachel Fay
Contact Details:	Guy's & St Thomas' Foundation NHS Trust<br>R&D Department<br>16th Floor, Tower Wing<br>Great Maze pond<br>London SE1 9RT<br>Ext Tel: 020 7188 7188<br>Int Tel: 54462<br>Fax: 0207 188 1295<br>Email: R&D@gstt.nhs.uk

King's College London will take primary responsibility for the study

**Chief Investigator**	
Laura A. Magee, MD, FRCPC, MSc, FACP	Professor of Women's Health<br>King's College London
Becket House, 5th Floor, 1 Lambeth Palace Road, London, SE1 7EU	+44 (0)20 7848 8573<br>Laura.A.Magee@kcl.ac.uk (preferred)<br>laura.magee1@nhs.net
**Data Monitoring Committee - DMC**	
**Chair**<br>Professor Diana Elbourne (Statistician)<br>Professor of Healthcare Evaluation<br>London School of Hygiene and Tropical Medicine	
**Vice-Chair**<br>Professor Henk Groen (Statistician)<br>Assistant Professor Reproductive and Perinatal Epidemiology<br>University Medical Center Groningen, University of Groningen	
**Independent Members**<br>Consultant Obstetrician to be appointed<br><br>Doctor Edile Murdoch (Neonatologist)<br>Senior Clinical Lecturer in Neonatal Medicine<br>University of Edinburgh	

Trial Steering Committee – TSC

Chair
Professor Tim Draycott (Obstetrician and Gynaecologist)
Professor of Obstetrics and Gynaecology North Bristol NHS Trust – Southmead Hospital
North Bristol NHS Trust – Southmead Hospital

Independent Members
Doctor Lucy MacKillop (Obstetric Physician)
Consultant Obstetric Physician
Oxford University Hospitals NHS Foundation Trust – John Radcliffe Hospital

Doctor Paul Mannix (Neonatologist)
Consultant Neonatal Medicine
North Bristol NHS Trust – Southmead Hospital

Professor Graeme MacLennan (Statistician)
Professor of Medical Statistics
University of Aberdeen

Ms. Ruth Unstead-Joss (PPI representative)
Mr. Marcus Green

Non-independent Members
Professor Laura A. Magee (Chief Investigator)
Professor of Women's Health
King's College London

Observer from the Funder
As determined by the National Institute for Health Research

Trial Management Group – TMG

Chief Investigator: Professor Laura A. Magee
Senior Trial Manager: Katie Kirkham
Trial Statistician: Eleni Gkini
Senior Trial Statistician: Catherine Moakes
Research Midwife: Sue Tohill
Data Manager: Sean Cole
Trial Programmer: Paul Riley (by invitation)
Team Lead: Clive Stubbs
Other members: Professor Jim Thornton (trialist, obstetrician), Professor Peter von Dadelszen (obstetrician), Professor Katie Morris (trialist, obstetrician).

Co-investigator Group – CiG
Chief Investigator: Professor Laura A. Magee<br><br>Senior Trial Manager: Katie Kirkham<br><br>Senior Trial Statistician: Catherine Moakes<br><br>Trial Statistician: Eleni Gkini<br><br>Other members: Professor Peter Brocklehurst, Professor Peter von Dadelszen, Associate Professor Jon Dorling, Clive Stubbs, Marcus Green, Dr Jennifer Hutcheon, Professor Ben W. Mol, Professor Tracy Roberts, Professor Joel Singer, Professor Jim Thornton, Janet Scott

BCTU Quality Assurance Team	
TBA	Quality Assurance Manager
Birmingham Clinical Trials Unit (BCTU)<br>Public Health Building<br>University of Birmingham<br>Birmingham B15 2TT	0121 414 7557 / 3351

Trial Office Contact Details	
Katie Kirkham	Senior Trial Manager
Clive Stubbs	Team Leader
Birmingham Clinical Trials Unit (BCTU)<br>Public Health Building<br>University of Birmingham<br>Birmingham B15 2TT	0121 4159109<br>WILL@trials.bham.ac.uk
Randomisation and database website:<br>https://www.trials.bham.ac.uk/WILL	Randomisation telephone: 0800 953 0274
Trial website:<br>https://www.birmingham.ac.uk/research/bctu/trials/womens/WILL/WILL.aspx	
Trial Twitter: https://twitter.com/WillTrial	

ABBREVIATIONS

Abbreviation	Term
**AE**	Adverse Event
**APEC**	Action on Pre-eclampsia Charity
**BCTU**	Birmingham Clinical Trials Unit
**BP**	Blood Pressure
**CI**	Confidence Interval
**CiG**	Co-investigators Group
**CRF**	Case Report Form
**CRN**	Clinical Research Network
**dBP**	diastolic BP
**DMC**	Data Monitoring Committee
**fullPIERS**	full Pre-eclampsia Integrated Estimate of Risk Score
**GCP**	Good Clinical Practice
**GCS**	Glasgow Coma Scale
**GP**	General Practitioner
**HDP**	Hypertensive Disorders of Pregnancy
**HIE**	Hypoxic-ischaemic encephalopathy
**ICF**	Informed Consent Form
**INR**	International Normalised Ratio
**IQR**	Interquartile Range
**ISF**	Investigator Site File
**ISRCTN**	International Standard Randomised Controlled Trials Number
**ITT**	Intention-to-Treat
**ITU**	Intensive Therapy Unit
**NICE**	National Institute of Clinical Excellence
**NIHR**	National Institute for Health Research
**NHS**	National Health Service
**NNT**	Number-Needed-to-Treat
**PI**	Principal Investigator

**PIS**	Participant Information Sheet
**PPH**	Postpartum Haemorrhage
**PPI**	Public-Patient Involvement
**RCT**	Randomised Controlled Trial
**RD**	Risk Difference
**REC**	Research Ethics Committee
**RR**	Risk Ratio
**SAE**	Serious Adverse Event
**SAP**	Statistical Analysis Plan
**sBP**	systolic BP
**SOP**	Standard Operating Procedure
**SpO2**	Peripheral capillary oxygen saturation
**STM**	Senior Trial Manager
**TIA**	Transient Ischaemic Attack
**TMG**	Trial Management Group
**TSC**	Trial Steering Committee
**UoB**	University of Birmingham

DEFINITIONS

Term	Abbreviation	Description
**Birmingham Clinical Trials Unit**	BCTU	The co-ordinating centre for the WILL trial.
**Policies**	POL	Policies are developed to describe the approach of the University of Birmingham (UoB) on areas that are heavily regulated. Policies may also be developed when there is ambiguity in how regulatory requirements should be implemented in the Quality Management System (QMS) or when procedures to be captured in the QMS address areas controversial within the UoB at the time of implementation. Policies explain why the UoB has its procedures, especially when they seem to deviate from the regulatory requirements. Policies should be read in conjunction with the relevant SOP. Policies that are not part of a Quality Manual are coded up as 'POL'.
**Quality Control Documents**	QCD	Quality Control Documents can be instructions, forms, templates or checklists. They are developed to share best practices, promote standardisation to guarantee quality standards are maintained and reduce resources otherwise needed to develop similar documents. Unless indicated otherwise in the relevant Standard Operating Procedure (SOP), QCDs are not mandatory and are designed to be an optional aid to UoB staff.
**Quality Management System**	QMS	A Quality Management System (QMS) is a system that includes procedures and policies to describe how certain tasks should be performed and that encapsulate any standards and/or regulatory requirements that may apply to those tasks. By adhering to the Quality Management System, the user and the UoB will be assured that applicable regulations are adhered to.
**Source Data**		All information in original records and certified copies of original records of clinical findings, observations, or other activities in a clinical trial necessary for the reconstruction and evaluation of the trial.
**Standard Operating Procedures**	SOP	Standard Operating Procedures are detailed written instructions to achieve uniformity in the performance of a specific function. They define tasks, allocate responsibilities, detail processes, indicate documents and templates to be used and cross-reference to other work instructions and guidance or policy documents. They are standards to which the UoB may be audited or inspected.

TRIAL SUMMARY

Title WILL (When to Induce Labour to Limit risk in pregnancy hypertension) - a multicentre, randomised controlled trial

Objectives To investigate the clinical effectiveness and cost-consequences of planned early term delivery at 38^{+0} to 38^{+3} weeks' gestation, compared with usual care at term, in pregnant women with chronic or gestational hypertension that develops by 37^{+6} weeks' gestation.

Trial Design: Pragmatic, parallel-group, open-label, multicentre, randomised controlled trial (with an internal pilot) with two co-primary outcomes: a maternal outcome assessing superiority and a neonatal outcome assessing non-inferiority.

Participant Population and Sample Size: The trial will recruit 1,080 pregnant women with chronic or gestational hypertension, from NHS consultant-led maternity units in the UK.

Eligibility Criteria:

INCLUSION CRITERIA:

(i) Maternal age ≥16 years;
(ii) Diagnosis of chronic or gestational hypertension (see Section 4.1);
(iii) Singleton pregnancy;
(iv) Live fetus;
(v) Gestational age of 36^{+0} to 37^{+6} weeks; and
(vi) Able to give documented informed consent to participate.

EXCLUSION CRITERIA:

(i) Contraindication to either one of the trial arms (e.g., evidence of pre-eclampsia);
(ii) Severe hypertension [i.e., blood pressure (BP) ≥160mmHg systolic or ≥110mmHg diastolic] until BP falls below this level (i.e. it is 'controlled', see Section 4.2);
(iii) Major fetal anomaly anticipated to require neonatal unit admission; or
(iv) Participation in another timing of delivery trial.

NOTE: Neither maternal co-morbidities (e.g., diabetes) nor fetal size will be exclusion criteria.

Interventions:

Planned early term delivery at 38^{+0} to 38^{+3} weeks by labour induction (local protocol) or elective Caesarean.

Usual care at term, with maternal and fetal monitoring (local protocol), awaiting spontaneous labour or delivery indicated by clinical need (e.g., refractory severe hypertension or pre-eclampsia).

Primary Outcome Measures:

MOTHER: Composite of poor maternal outcome until primary hospital discharge home or 28 days after birth (whichever is *earlier*), defined as:

- Severe hypertension (i.e., systolic BP (sBP) ≥160 or diastolic BP (dBP) ≥110mmHg); or
- Maternal death; or
- Maternal morbidity defined as any of the following: GCS<13; stroke; TIA; eclampsia; blindness; uncontrolled hypertension; inotropic support; pulmonary oedema; respiratory failure; SpO2 <90%; myocardial ischaemia or infarction; hepatic dysfunction, hepatic haematoma or rupture; acute kidney injury or dialysis; platelet count $<50x10^9/L$; transfusion; or placental abruption. These were adapted from a Delphi consensus in hypertensive pregnancy (10;11).

BABY: Neonatal care unit admission for ≥ 4 hours, until primary hospital discharge home or 28 days after birth (whichever is earlier).

Secondary Outcome Measures (assessed at hospital discharge or 28 days postpartum, whichever is earlier, unless otherwise stated)**:**

Key Maternal

- Caesarean delivery

Other Maternal

- Instrumental vaginal delivery or Caesarean delivery (vs. spontaneous vaginal delivery)
- Infection of the Caesarean wound, episiotomy, or vaginal tear, as applicable, assessed at six weeks postpartum
- Individual components of maternal co-primary outcome
- 'Poor maternal outcome' assessed at six weeks postpartum
- Elevated liver enzymes
- Platelet count $<100x10^9$/L
- Pre-eclampsia
- Postpartum haemorrhage (PPH)
- Sepsis
- Intensive therapy unit (ITU) admission
- Potential co-interventions, including antihypertensive therapy taken; magnesium sulphate; bedrest at home; use of home BP monitoring; maternal blood or urine testing at the laboratory prior to delivery admission; outpatient visits; medical, day, or maternity assessment unit visits; acute care visits; antenatal admissions; fetal cardiotocography; and fetal ultrasound
- Clinical indications for birth
- Maternal satisfaction, as measured by the Childbirth Experience Questionnaire

Fetal/Neonatal

- Neonatal care unit admission ≥4 hours assessed at 28 days after birth
- Indication for neonatal care unit admission ≥ 4 hours
- Respiratory morbidity
- Clinical respiratory problem
- Chest x-ray
- Hypoxic-ischaemic encephalopathy (HIE)
- Sepsis
- Major operation
- Birthweight
- Apgar scores at 1, 5 and 10 minutes
- Stillbirth
- Neonatal death
- Breastfeeding established
- Exclusive breastfeeding

Health Economics

- Cost-consequence analysis from NHS perspective (enrolment to hospital discharge)

WILL Trial Schema

Inclusion criteria

1. Maternal age ≥16 years;
2. Diagnosis of chronic or gestational hypertension;
3. Singleton pregnancy;
4. Live fetus;
5. Gestational age of 36^{+0} to 37^{+6} weeks*;
6. Able to give documented informed consent to participate.

Exclusion criteria

1. Contraindication to either one of trial arms (e.g. evidence of pre-eclampsia);
2. sBP≥160mmHg or dBP≥110mmHg until BP is controlled;
3. Major fetal anomaly anticipated to require neonatal unit admission;
4. Participation in another timing of delivery trial

NOTE: Neither maternal co-morbidities (e.g., diabetes) nor fetal size will be exclusion criteria.

Randomisation

(1:1 allocation, minimised by centre, hypertension type, and prior Caesarean)

Planned early term delivery

Planned early term delivery at 38^{+0} to 38^{+3} weeks' gestation, by labour induction (local protocol†) or elective Caesarean

Usual care at term

Usual care at term, with maternal and fetal monitoring (local protocol†), awaiting spontaneous labour or delivery indicated by clinical need (e.g., refractory severe hypertension or pre-eclampsia)

Follow-up of mother and baby until primary hospital discharge or 28 days after birth, whichever is *earlier*

Co-primary outcome measure (mother)

Composite of 'poor maternal outcome' defined as:

- **Severe hypertension** (sBP ≥160 or dBP ≥110mmHg, as highlighted by national MBRRACE report); *or*
- **Maternal death**; *or*
- **Maternal morbidity** defined as one or more of the following: GCS <13; stroke; TIA; blindness; uncontrolled hypertension; inotropic support; pulmonary oedema; respiratory failure; SpO2 <90%; myocardial ischaemia or infarction; hepatic dysfunction, hepatic haematoma or rupture; acute kidney injury or dialysis; platelet count $<50x10^9$/L; transfusion; or placental abruption. These were adapted from Delphi consensus in hypertensive pregnancy.

Co-primary outcome measure (baby)

Neonatal care unit admission ≥4 hours

Follow-up of mother (to 6 weeks after birth) and baby (to 28 days after birth)

'Poor maternal outcome' for the mother (secondary outcome) **and**
Neonatal care unit admission ≥4 hours for the baby (secondary outcome)

dBP (diastolic blood pressure), GCS (Glasgow Coma Scale), sBP (systolic blood pressure), SpO2 (peripheral capillary oxygen saturation), TIA (transient ischaemic attack)

**Women will be consented at 36^{+0} to 37^{+6} weeks' gestation, but will be randomised if they remain undelivered and well, from 37^{+0} to 37^{+6} weeks' gestation. This approach should optimise recruitment, minimise the number of women (<20%) who may go into spontaneous labour or require delivery for maternal/fetal reasons prior to 38^{+0} weeks' gestation, and allow for sufficient time for booking of labour induction (or elective Caesarean) in the 'Planned delivery' group.*

† NICE guidance compliant

WILL Flow Diagram V6.0 Apr 2022

TABLE OF CONTENTS

BACKGROUND AND RATIONALE 17

1.1 Background 17

1.2 Trial Rationale 17

1.2.1 Justification for participant population 18

1.2.2 Justification for design 18

1.2.3 Choice of intervention 18

1.2.4 Choice of outcome 19

2. OBJECTIVES 19

2.1 Primary Objective 19

2.2 Secondary Objective 19

3. TRIAL DESIGN AND SETTING 19

3.1 Trial Design 19

3.2 Trial Setting 20

3.3 Identification of Participants 20

3.4 Sub-Studies 21

3.5 Assessment of Risk 21

4. ELIGIBILITY 21

4.1 Inclusion Criteria 21

4.2 Exclusion Criteria 21

4.3 Co-enrolment 22

5. CONSENT 22

5.1 Approach 22

5.2 Consent process 22

5.3 Remote consent 22

5.4 Consent for data linkage and future research 23

5.5 Following consent 23

6. RECRUITMENT, SCREENING, ENROLMENT AND RANDOMISATION 24

6.1 Recruitment, Screening and Enrolment 24

6.2 Randomisation 24

6.3 Informing the Participant's GP 25

6.4 Blinding 25

6.5 Non-adherence 26

7. TRIAL TREATMENT / INTERVENTION 26

7.1 Intervention arm: Planned early term delivery at 38^{+0-3} weeks' gestation 26

7.2 Control arm: Usual care at term 26
7.3 Care in both groups 27
7.4 Accountability Procedures 27
7.5 Intervention Modification 27
8. OUTCOME MEASURES AND STUDY PROCEDURES **28**
8.1 Co-primary Outcomes 28
8.1.1 Maternal co-primary outcome 28
8.1.2 Neonatal co-primary outcome 28
8.2 Secondary Outcomes 28
8.2.1 Maternal 28
8.2.1.1 Key Maternal 28
8.2.1.2 Other Maternal 28
8.2.2 Fetal/Neonatal 30
8.2.3 Health Economics 31
8.3 Schedule of Assessments 32
8.4 Participant Withdrawal 34
9. ADVERSE EVENT REPORTING **35**
9.1 Definitions 35
9.2 Reporting Requirements 35
9.3 Serious Adverse Events (SAEs) Requiring Reporting in WILL 36
9.3.1 Protocol-exempt SAEs do NOT require reporting on a SAE form 36
9.3.2 Expeditable SAEs require expedited reporting on the SAE Form 37
9.4 Reporting Procedures and Follow-up at Site 37
9.4.1 Reporting procedure for Serious Adverse Events by sites 37
9.4.2 Assessment of Causality (Relatedness) by the PI 38
9.4.3 Provision of follow-up information 38
9.5 Reporting Procedures by the WILL Trial Office 38
9.5.1 Assessment of Expectedness by CI 38
9.5.2 Reporting SAEs to third parties 39
9.6 Urgent Safety Measures 39
10. DATA HANDLING AND RECORD KEEPING **40**
10.1 Source Data 40
10.2 Case Report Form (CRF) Completion 40
10.3 Data Management 41
10.4 Data Security 42
10.5 Archiving 42
11. QUALITY CONTROL AND QUALITY ASSURANCE **43**

11.1 Site Set-up and Initiation 43
11.2 Monitoring 43
11.3 Onsite Monitoring 43
11.4 Central Monitoring 43
11.5 Audit and Inspection 44
11.6 Notification of Serious Breaches 44
12. END OF TRIAL DEFINITION **44**
13. STATISTICAL CONSIDERATIONS **44**
13.1 Sample Size 44
13.2 Analysis of Outcome Measures 45
13.2.1 Primary Outcome Measures 45
13.2.2 Secondary Outcome Measures 45
13.2.3 Subgroup Analyses 46
13.2.4 Missing Data and Sensitivity Analyses 46
13.3 Planned Interim Analysis 46
13.4 Planned Final Analyses 46
14. TRIAL ORGANISATIONAL STRUCTURE **47**
14.1 Funder 47
14.2 Sponsor 47
14.3 Coordinating Centre 47
14.4 Trial Management Group 47
14.5 Trial Steering Committee 47
14.6 Data Monitoring Committee 47
14.7 Co-investigator Group 48
14.8 Finance 48
15. ETHICAL CONSIDERATIONS **48**
16. CONFIDENTIALITY AND DATA PROTECTION **48**
17. FINANCIAL AND OTHER COMPETING INTERESTS **49**
18. INSURANCE AND INDEMNITY **49**
19. AMENDMENTS **49**
20. PUBLICATION POLICY **49**
21. REFERENCE LIST **51**
22. APPENDIX **56**

BACKGROUND AND RATIONALE

1.1 Background

In the UK, up to 55,000 pregnancies/year are complicated by chronic hypertension (diagnosed before pregnancy or at <20 weeks' gestation) or gestational hypertension (diagnosed at ≥20 weeks), and half of these women will reach term gestational age. Early term delivery (at 37-38 weeks) may reduce maternal complications and stillbirth, but it may also increase neonatal morbidity and costs, related primarily to the cost of maternal and fetal surveillance during expectant care and possibly increased Caesarean deliveries that are greater than the costs of labour induction(18-20). There are no high-quality data on which to base clinical decision-making.

Variation in guidelines and practice supports equipoise. NICE (2010) advises that timing of birth for women with chronic or gestational hypertension, "be agreed upon with the woman" (6). Current care at term involves maternal and fetal surveillance, and intervention for maternal morbidity or fetal compromise, either of which may be rapid or unexpected. Practice varies widely; in a survey of Control of Hypertension In Pregnancy Study Trial (including the UK), 70 respondents highlighted variable practice, with delivery currently offered at 37 weeks' (16%), 38 weeks' (33%), 39 weeks' (20%), 40 weeks' (20%), and 41 weeks' gestation (12%).

To guide care for these high-risk women, the WILL trial (When to Induce Labour to Limit risk in pregnancy hypertension) compares a policy of delivery at 38 weeks, against usual care at term (or as clinical need dictates), with regards to maternal complications (and Caesareans), whilst ensuring that neonatal health is not compromised(2,3).

Among women with chronic(1) or gestational hypertension(18), observational data suggest that delivery between 38^{+0} and 39^{+6} weeks may optimise outcomes for the baby, by minimising stillbirth that rises in incidence with advancing gestational age at term, and neonatal morbidity that falls with advancing gestational age. However, observational studies are confounded by indication for delivery, so it is not possible to estimate with any certainty the impact of different gestations of delivery on perinatal outcomes. Also, the impact of planned delivery at term (i.e., at 37^{+0} to 41^{+6} weeks) on maternal morbidity or Caesarean could not be assessed.

There are no definitive trials that have established how best to manage women with chronic or gestational hypertension who reach 37 weeks and require delivery at term; yet, these women represent 1/3 of all women with pregnancy hypertension in the UK. There are only limited relevant data from five trials (1,819 women) in the 2017 Cochrane review (3) (see Section 22, Appendix 1, Table 1). The vast majority of women in prior trials either had proteinuric pre-eclampsia, or were randomised at earlier or later gestational ages than we plan (or both).

1.2 Trial Rationale

There are limited data to inform care of women who would be eligible for WILL (i.e., 50 women with chronic hypertension in Hamed 2014 (4) and at most, 188 women with gestational hypertension in the 37^{+0-6} week subgroup of HYPITAT I (5)). These data suggest that earlier delivery at term may be beneficial to women with chronic or gestational hypertension, without increasing risk to babies or Caesarean delivery. However, the number of women enrolled was very small, and both trials were conducted in settings (Egypt and The Netherlands) where compared with the UK, there are differences in antenatal care, including less frequent use of antihypertensives for BP of ≥150/100mmHg (as advised by NICE)(6).

Our analysis is consistent with the 2017 Cochrane review that concluded, "*Further studies are needed to look at the different types of hypertensive diseases and that optimal timing of delivery for these conditions*."(3) Our views are consistent with NICE (2010) guidance, published after HYPITAT I, that advises that timing of birth for women with chronic or gestational hypertension, "*be agreed upon between the woman and the senior obstetrician*" due to a lack of adequate evidence to guide practice(6); as part of the 2017 update of this guidance, no evidence was identified to revise timing of delivery recommendations for women with chronic or gestational hypertension (www.nice.org.uk, accessed 01 Sept 2017).

Also of note are two reviews of trials for induction at term in non-hypertensive pregnancies. Induction was associated with a reduction in Caesareans, stillbirth, and neonatal death and morbidity (46;47). There was no negative impact of induction on maternal death, operative delivery, or postpartum haemorrhage. In a recent trial of 6106 low-risk nulliparous women at term, induction at 39 weeks was associated with fewer Caesareans and development of a maternal hypertensive disorder, without an increase in perinatal mortality/morbidity(66). Also, in a large RCT, induction (vs. expectant care) was associated with more reassurance, less worry, and no perception of a decrease in maternal control during birth(48). Limited non-RCT data suggest that following induction, maternal pain is similar and satisfaction high(49).

WILL aims to address optimal timing of delivery for women with chronic or gestational hypertension who reach term gestational age and are otherwise well. The study will provide data for women to make informed choices about maternal and perinatal risk and the NHS to plan services. We anticipate receptiveness to the WILL results as induction is a familiar intervention, and earlier delivery rates have been successfully implemented for women with pre-eclampsia at term(50;51).

1.2.1 Justification for participant population

Women with chronic or gestational hypertension near term gestational age make up more than one third of women with pregnancy hypertension, yet there are scant RCT data with which to guide their care. We are targeting women with either chronic or gestational hypertension, but it is a historical view that they are completely different entities; increasingly, there is recognition of a shared underlying pathophysiology (e.g., genetics, metabolic syndrome) and despite differences in maternal age and environment, there is a convergence of clinical hypertension and cardiovascular events over time(54-56).

Women will not need to be hypertensive at the time of eligibility (see Section 4.1). For women with chronic hypertension, BP typically falls in pregnancy and the risk of adverse outcomes is related to vascular accelerated placental ageing, rather than the level of BP itself(1). Women with gestational hypertension may have normal BP because they have been started on antihypertensive medication(1). If women are hypertensive, their sBP must be <160mmHg and their dBP <110mmHg as women with BP values in excess of this are likely to have plans for delivery.

We will consent women from 36^{+0} to 37^{+6} weeks when they present for routine care, but we will not randomise them until $37^{+0\text{-}6}$ weeks, to minimise inclusion of women who deliver spontaneously or develop an indication for delivery before this time.

1.2.2 Justification for design

WILL is a pragmatic trial, enrolling 'all comers' with chronic or gestational hypertension who reach term and evidence of problems that warrant delivery. WILL compares real clinical care that cannot be masked: planned early term delivery (by labour induction or elective Caesarean) and ongoing surveillance at term. A nine-month internal pilot will ensure that the trial design and processes are feasible.

1.2.3 Choice of intervention

Early term delivery (at 37-38 weeks) may reduce maternal complications and stillbirth, but it may also

increase neonatal morbidity and costs, related primarily to the cost of maternal and fetal surveillance during expectant care and possibly, increased Caesarean deliveries that are greater than the costs of labour induction(18-20).

We have considered carefully how best to enrol women who will inform the research question, and achieve separation in gestational age at birth that should translate into benefits for mothers, without increasing risk for babies. We will not plan birth before 38^{+0} weeks, given concerns raised in previous trials that birth before this may increase neonatal respiratory distress (4;12). As usual care at term is likely to be delivery from no earlier than 39 weeks in the absence of clinical need, we are confident that we will achieve our goal of an actual difference in gestational age at birth of one week.

1.2.4 Choice of outcome

With regards to our maternal co-primary outcome, severe hypertension is stated by the national MBRRACE-UK reports (of Confidential Enquiry into Maternal Deaths and Morbidity) as a clinical emergency requiring urgent treatment(65). Maternal death or morbidity was adapted from fullPIERS and iHOPE Delphi consensus (10;11). The combined primary outcome is also being used in the HTA-funded PHOENIX trial (ISRCTN01879376), and reflects the multisystem nature of pregnancy hypertension complications that are not always mediated through pre-eclampsia(10;11). Also, we capitalised on prior PPI as part of iHOPE, the JLA Priority Setting Partnership on stillbirth (that prioritised antenatal interventions), and the RCOG 'Each Baby Counts' initiative and MBRRACE-UK perinatal enquiry that emphasise reduction of TERM stillbirth(14,15).

With regards to our neonatal co-primary outcome, admission to a neonatal unit is distressing to women (at the very least due to the separation from their baby) and expensive for the health system, even though such admission is usually for neonatal morbidity which at term, is mainly mild and usually not life or health-threatening(17).

2. OBJECTIVES

2.1 Primary Objective

To evaluate if planned early term delivery at 38^{+0} to 38^{+3} weeks' gestation, compared with usual care at term in pregnant women with chronic or gestational hypertension that develops by 37^{+6} weeks' gestation reduces a composite of 'poor maternal outcome', without unduly increasing neonatal care unit admission for ≥ 4 hour measured to hospital discharge or 28 days after delivery (whichever is earlier).

2.2 Secondary Objective

To evaluate the response of planned early term delivery at 38^{+0} to 38^{+3} weeks, compared with usual care at term, in pregnant women with chronic or gestational hypertension that develops by 37^{+6} weeks on maternal and neonatal clinical outcomes and cost-consequence outcomes from an NHS perspective.

3. TRIAL DESIGN AND SETTING

3.1 Trial Design

WILL is a pragmatic, parallel-group, open-label, multicentre, randomised controlled trial (with a nine-month internal pilot), with two co-primary outcomes: a maternal outcome assessing superiority and a neonatal outcome assessing non-inferiority.

This nine-month **internal pilot** was undertaken in centres chosen to be representative of sites overall (e.g., number of births and region to test processes of the main trial prior to all centres opening). 'Stop-go' criteria (see Table 2) were used by the Trial Steering Committee (TSC) and Data Monitoring Committee (DMC) in a joint meeting held at the end of the pilot period to review the data and examine whether the progression criteria have been met. It was recommended that recruitment to the main trial should proceed with no break and data from the pilot phase will be analysed together with the main trial data collected.

Table 2: 'Stop-go' criteria used in the internal pilot trial (N, % women or median)

	**Progression criteria**		
	**Green (go)**	**Amber (assess and adapt)**	**Red (stop)**
**Randomised of those consented**	>80%	50-79%	<50%
**Recruitment** rate relative to overall target for 20 pilot sites*	>60%	30-59%	<30%
**Randomised & delivered at <38+0 weeks** (of those consented)	<15%	15-50%	>50%
**Median between-group difference in gestational age at birth†**	≥7 days	4-6 days	0-3 days
**Action to be taken based on type and number of criteria met**	Proceed with protocol unchanged if ALL criteria are met	Adapt protocol if ONE/MORE criteria are met	Project not feasible and trial will be ended if TWO/MORE criteria are met

** Allowing for a delay in recruitment following site opening we will look for an average recruitment target of 110 women from 20 sites in 8 months.*

† Only the DMC reviewed this criterion, and reported to the TSC which of green, amber, or red criteria were met.

3.2 Trial Setting

NHS consultant-led maternity units in the UK.

3.3 Identification of Participants

We will consent women with a diagnosis of chronic or gestational hypertension at a gestational age of 36^{+0} to 37^{+6} weeks (see Section 4.1).

Ideally, women will learn about WILL early in their pregnancy, but eligibility will not be confirmed until 36^{+0} to 37^{+6} weeks to minimise enrolment of women (approximately 18%)(52) who may develop an indication for delivery (e.g., abruption) or go into spontaneous labour prior to $38^{+0\text{-}3}$ weeks, the planned timing of delivery in the intervention arm.

At 36^{+0} to 37^{+6} weeks, ideally at their routine antenatal visit, women will be assessed by the research midwife for eligibility. The research midwife or medically-qualified member of the obstetric team will obtain informed consent (as per locally-accepted practice). The research midwife will collect baseline data.

At $37^{+0\text{-}6}$ weeks, women will be re-contacted by the midwife, by phone or in person, to confirm that no new plans have been made for birth, and the women 'remains well' and does not need to be reassessed for an indication for delivery. 'Well' is defined as:

(i) reporting no new symptoms of possible pre-eclampsia (i.e., severe headache, persistent visual scotomata, or right upper quadrant or epigastric abdominal pain);

(ii) having acceptable BP if she is undertaking home BP monitoring, according to criteria set by the woman's care provider; and

(iii) reporting no decrease or a change in the pattern of fetal movement.

Baseline characteristics collected at consent that could change between consent and randomisation and that will be used in subgroup analyses, will be reconfirmed. These baselines characteristics are as follows: severe hypertension in the index pregnancy, antihypertensive therapy, gestational diabetes mellitus, and if the woman smokes.

Women who 'remain well' will be randomised.

3.4 Sub-Studies

There are currently no sub-studies associated with the WILL trial.

3.5 Assessment of Risk

The WILL trial involves no new intervention and has no added risk to the mother or baby; instead, WILL *measures* risk. The interventions of planned early term delivery and usual care at term are both standards in current daily clinical practice in the UK (and internationally). As such, in WILL, there will be no risks to women or babies over and above those associated with the current practice within the NHS associated with timing of delivery for women with chronic or gestational hypertension by term.

4. ELIGIBILITY

4.1 Inclusion Criteria

Women must meet ALL of the criteria at consent:

• **Maternal age ≥16 years**;

• **Diagnosis of chronic* or gestational hypertension****;

• **Singleton pregnancy**;

• **Live fetus** (confirmed by auscultation of fetal heart tones within one week before consent);

• **Gestational age of 36^{+0} to 37^{+6} weeks** (measured by dating ultrasound or the last menstrual period); and

• **Able to give documented informed consent to participate**.

** The diagnosis of chronic hypertension will be based on either hypertension diagnosed before pregnancy or an elevated BP (≥140mmHg systolic or ≥90mmHg diastolic) before 20 weeks' gestation, according to contemporaneous NICE guidance (currently NG133). The diagnosis should have been made by a doctor or midwife. These women do not need to have elevated BP or be on antihypertensive medication at consent, as BP typically falls in pregnancy and the risk of adverse outcomes is related to accelerated vascular placental aging, rather than the level of BP itself(1).*

*** The diagnosis of gestational hypertension will be based on an elevated BP (≥140mmHg systolic or ≥90mmHg diastolic) at ≥20 weeks, without new proteinuria (≥2+ by dipstick, ≥30mg/mmol by spot protein:creatinine ratio, or ≥0.3g/d by 24hr urine collection) according to contemporaneous NICE guidance (currently NG133). The diagnosis should have been made by a doctor or midwife. Women do not need to be hypertensive at consent.*

4.2 Exclusion Criteria

Women must NOT have ANY of the criteria below at consent:

- **Contraindication to either one of the trial arms** (e.g., evidence of pre-eclampsia). Note that pre-eclampsia may develop after randomisation (see secondary outcomes, Section 8.2.1);
- **Severe hypertension (i.e., BP ≥160mmHg systolic or ≥110mmHg diastolic) until BP is controlled** (For WILL, 'controlled' blood pressure is defined as an sBP <160mmHg and dBP <110mmHg.)
- **Major fetal anomaly anticipated to require neonatal care unit admission**; or
- **Participation in another timing of delivery trial.** This includes prior participation in WILL.

4.3 Co-enrolment

Co-enrolment will be allowed to trials other than those with a timing of delivery intervention, as long as such participation does not represent a threat to retention of women in the WILL trial. Advice should be sought from TMG via the WILL trial office.

5. CONSENT

5.1 Approach

A Participant Information Sheet (PIS) will be provided to facilitate the consent process. Ideally, women will receive this (or an introductory pamphlet) when identified as having chronic or gestational hypertension, even if much earlier in pregnancy. If some routine hospital appointments are taking place virtually at the site, or some component of remote care is locally-accepted practice, a member of the site research team may contact the woman by telephone or videoconference, for verbal consent to discuss the PIS with her. If she is interested in participating in the trial, permission will be obtained to send her the PIS (and ICF in case this is needed in the future), electronically or in hard copy, as she prefers, and have a follow-up call. Sites should adhere to their local policies when emailing identifiable or confidential information to women.

5.2 Consent process

At 36^{+0} to 37^{+6} weeks, ideally at a routine antenatal visit, fully informed and documented consent will be obtained after the eligibility criteria have been checked. Consent will be obtained by a GCP-trained midwife or medically-qualified member of the obstetric team, as per locally accepted practice. If the woman is still undelivered at 37^{+0-6} weeks, she will be randomised (as described in Section 6.2) by the research midwife. The responsibility to check eligibility criteria and obtain documented informed consent for each woman prior to performing any trial related procedure will be the responsibility of the PI or their delegate(s), as captured on the **Site Signature and Delegation Log**.

Details of the informed consent discussions will be recorded in the woman's medical notes. The woman will be given the opportunity to ask questions before the signing and dating of the latest version on the REC-approved Informed Consent Form (ICF). The Investigator or delegate(s) will then sign and date the ICF.

If in-person consent is possible and acceptable to the woman, she will sign and date the latest version of the REC-approved ICF, and then the Investigator or delegate(s) will sign and date the ICF.

5.3 Remote consent

If in-person consent is not possible or desirable by the woman, remote documented consent may be undertaken. The informed consent discussions will proceed as detailed in sections 5.1 and 5.2 by telephone or video-conference and the details will be recorded. The woman will be asked to initial the boxes on the ICF, and sign and date the form whilst on the call. If the ICF were sent to the woman by post the woman will be asked to take a photo or scan of the signed ICF, and email it to the site staff, who will print it off and counter-sign. If the ICF were sent to the woman electronically, and she has

access to a printer, she can print a hard copy and will be asked to take a photo or scan of the signed ICF, and email it to the site staff, who will print it off and counter-sign. The original signed and counter-signed ICF will be placed in the Investigator Site File (ISF), a copy will be sent to the participant (electronically or by post), and a copy will be filed in the woman's medical notes. Alternatively, if there is concern that the woman cannot email the signed ICF in a timely manner or she does not wish to do this, she will be offered the following alternative. The informed consent discussions will proceed and be recorded as in sections 5.1 and 5.2 for the in person option, however, the site staff will initial the boxes on the ICF in discussion with the woman, sign and date the ICF with a witness present, and then send a copy of the completed ICF to the woman for her records.

5.4 Consent for data linkage and future research

Potential participants will be asked for their permission to link study data collected about them and their babies with other routinely-collected health, educational, or social data, in order to learn more about the impact of different planned timing of delivery on long-term health for women with high blood pressure in a term pregnancy. Also, potential participants will be asked if they would be willing to be contacted in the future about other studies requiring collection of new data and tracking their or their babies' long-term health, and development and progress of their child (subject to additional funding). The consent form will record the response by the woman. The consent form will state that by ticking the "Yes" box the woman or her child is not obliged to participate in any future studies requiring collection of new data. Data linkage will only occur once the appropriate data agreement(s) are fully executed.

<u>Examples of databases that may be linked with participant's and their babies' study data</u>:

- Hospital Episodes Statistics;
- Genvasc Primary Care;
- HIC Acute Coronary Syndromes (from 2010);
- NHSBSA;
- Education Pupil School;
- Personal Demographics Service.

5.5 Following consent

Following consent, a confirmatory e-mail will be sent to the BCTU, local research midwife and local PI, responsible clinician and Chief Investigator.

A copy of the signed ICF will be forwarded (according to local NHS policy) to the Trials Office at Birmingham Clinical Trials Unit (BCTU). Explicit consent will be sought for this transfer of identifiable information on the ICF itself. With the woman's consent their GP will be informed that they are taking part in the WILL trial.

Once the woman is entered into the study, the woman's study number will be entered on the ICF maintained in the ISF.

At each visit, after consent is obtained, the woman's willingness to continue in the trial will be ascertained and documented in the medical notes. Throughout the trial, the woman will have the opportunity to ask questions about the trial. Any new information that may be relevant to the woman's continued participation will be provided. Where new information becomes available which may affect the woman's decision to continue, she will be given time to consider and asked to re-consent to their further participation in the trial. Re-consent will be documented in the medical notes. The woman's right to withdraw from the trial will remain (see Section 8.4).

Electronic copies of the PIS and ICF will be available from the Trials Office and printed or photocopied onto the headed paper of the local institution for this UK trial. Details of all women approached about

the trial will be recorded in the electronic Screening Consent and Key Baseline Variables Form.

6. RECRUITMENT, SCREENING, ENROLMENT AND RANDOMISATION

6.1 Recruitment, Screening and Enrolment

A flowchart of the trial is presented on page 13 and the participant recruitment process is shown in Section 22, Appendix 2. Women will not receive any payment for participating in the WILL study

Careful consideration has been given to when to enrol women in order to inform the research question, and achieve separation in gestational age at birth that should translate into benefits for mothers, without increasing risk for mothers or their babies. We will consent women from 36^{+0} to 37^{+6} weeks when they present for routine care and collect baseline data following consent. Participants will not be randomised until 37^{+0-6} weeks' gestation, to minimise inclusion of women who deliver spontaneously or develop an indication for delivery before this time.

Recruitment strategies will vary depending on site logistics, but it is generally anticipated that women will be informed about the study at their regular clinical prenatal visits or at admission to hospital. Women with chronic hypertension will be informed about the study in early pregnancy; with a view to consenting women at 36^{+0} to 37^{+6} weeks (as per inclusion criteria) if no preterm maternal or fetal complications have occurred that merited delivery. Although anticipated to be less common, some eligible women will be identified while inpatients.

Research staff will be asked to screen clinic booking lists, medical assessment unit lists, and inpatient lists for women with chronic or gestational hypertension, and liaise with their clinical colleagues to identify potentially eligible participants. Clinical staff with access to the clinical records will make the initial approach. Eligibility will be assessed initially by the research team on the **Site Signature and Delegation Log**. An electronic Screening Consent and Key Baseline Variables Form will be completed for all women who are assessed for eligibility and will be used centrally to monitor recruitment and offer support (where required); each woman will be assigned a study number. Consent will be obtained by a GCP-trained midwife or medically-qualified member of the obstetric team (according to local policies).

Trial promotional materials (such as posters and pamphlets) will be available to sites to raise awareness of the study in both women attending the clinics and care staff.

At 36^{+0} to 37^{+6} weeks, ideally at their routine antenatal visit, women's eligibility will be assessed by the research midwife. The research midwife or GCP-trained medically-qualified member of the obstetric team will obtain informed consent (according to locally-accepted practice). Following consent, baseline data collected by the research midwife (see Section 5). With the participant's consent, a confirmatory letter will be sent to the participant's local GP.

Investigators will keep their own study file log which links patients with their allocated study number in a Participant Recruitment/Consent Log. The Investigator must maintain this document, which is not for submission to the Trials Office. The Participant Recruitment/Consent Log should be held securely and in strict confidence.

6.2 Randomisation

If women were consented at 37^{+0} to 37^{+6} weeks, then they may be randomised at the same visit. However, women who were consented at 36^{+0} to 36^{+6} weeks must be re-contacted, at 37^{+0} to 37^{+6} weeks, by the midwife, by telephone or in person. This contact is to confirm verbally that the woman 'remains well', does not need to be reassessed for an indication for delivery, and remains willing to be randomised. (See Section 3.3 for the definition of 'well'.)

Women who 'remain well' will be randomised into the WILL trial using the computerised randomisation service at the BCTU. A paper-based randomisation system will be available as a back-up. Women who are not randomised will have data collected according to the Schedule of Assessments, Section 8.2.

Women will be randomised at 37^{+0} to 37^{+6} weeks, at an individual level in a 1:1 ratio to either:

1. Planned early term delivery at 38^{+0-3} weeks, or
2. Usual care at term

Randomisation will be provided by a computer-generated programme hosted by the University of Birmingham and checked by a statistician from BCTU using a minimisation algorithm to ensure balance in the treatment allocation over the following variables:

- Randomising centre (i.e., the recruiting centre);
- Hypertension type (chronic or gestational hypertension, see Section 4.1); and
- Prior Caesarean (yes/no)

A 'random element' will be included in the minimisation algorithm, so that each woman has a probability, of being randomised to the opposite intervention that they would have otherwise received. Full details of the algorithm used will be stored in a confidential document at BCTU. To avoid bias, the random allocation sequence is concealed from those responsible for recruiting women into the study. The minimisation balance will be monitored by the central Trial Team at BCTU.

Randomisation will be available by a secure online randomisation system hosted at the University of Birmingham (available at www.trials.bham.ac.uk/WILL), or by telephone (0800 953 0274). The online randomisation system will be available 24 hours a day, 7 days a week, apart from short periods of scheduled maintenance. The telephone randomisation line is available Monday to Friday, 09:00 to 17:00 (UK time), except for bank holidays and University of Birmingham closed days.

Unique log-in usernames and passwords will be provided to those who wish to use the online system and who have been delegated the role of randomising participants into the study as detailed on the **Site Signature and Delegation Log**.

When all eligibility criteria and data informing the minimisation variables have been provided to the online randomisation system, randomisation can proceed and a woman be allocated to a treatment group.

Following randomisation, a confirmatory e-mail will be sent to the BCTU, local research midwife and local PI, responsible clinician and Chief Investigator. With the participant's consent, a letter confirming randomisation will be sent to the participant's local GP. The woman will be informed verbally of the group she has been allocated to, and the group allocation recorded in the relevant notes.

6.3 Informing the Participant's GP

As discussed in Sections 6.1 and 6.2, with the participant's consent, her GP will be notified of consent and randomisation in the WILL Trial**.**

6.4 Blinding

WILL compares clinical interventions that cannot be masked: planned early term delivery and usual care (i.e., ongoing surveillance) at term. It is not possible to mask care providers or women participating in the study to the interventions allocated or undertaken. As such, the WILL study is not 'blinded' (i.e., 'masked').

For our co-primary maternal outcome (of poor maternal outcome), there will be local site PI/delegate sign-off based on review, masked to allocated group, of primary case notes; photocopies (of the relevant pages only) will be prepared by the research staff to ensure that the PI/delegate is masked to group allocation. Should the local PI have been involved in the care of the woman, s/he will arrange

for a designate to undertake sign-off of the validity of the maternal co-primary outcome component(s). Adjudication will not be necessary for the neonatal co-primary (safety) outcome and Caesarean delivery for which ascertainment will not be biased.

6.5 Non-adherence

It is possible that women in the planned early term delivery (at 38^{+0-3} weeks) may be delivered at times different from the arms to which they have been assigned. Reasons for this will be collected on the CRFs.

In the 'planned early term delivery at 38^{+0-3} weeks' group, reasons for midwife or hospital doctor-initiated delivery before 38^{+0} weeks are set out in Section 7.5. Reasons for failure to *initiate* delivery (by induction or elective Caesarean) before 38^{+3} weeks may include busy hospital induction or theatre schedules that allow for *initiation* of delivery only after 38^{+3} weeks. Spontaneous onset of labour before 38^{+0} weeks will not be considered non-adherence as the intervention is one of *planned* early term delivery at 38^{+0-3} weeks.

Reasons for timing of delivery (i.e., spontaneous, based on clinical need, or non-adherence) will be recorded and monitored. Adherence (as a binary outcome) will be defined only in the 'planned early term delivery at 38^{+0-3} weeks' group, as timing of delivery initiation that is consistent with the allocated group or a result of either spontaneous onset of labour or delivery for clinical need. Non-adherence will be identified when the timing of delivery is not consistent with this allocated group, because of busy labour ward or theatre schedules, or the reason for the timing of delivery was clinician preference or the woman's preference; timing of delivery based on clinician preference or the woman's preference will require completion of a **Protocol Deviation Form**. Repeated deviations from the same site may be regarded as a serious breach and will trigger an investigation.

In both the 'planned early term delivery at 38^{+0-3} weeks' and 'usual care at term' groups, adherence will be further assessed by gestational age at birth, monitored to encourage adequate separation of gestational age at birth between the trial arms.

6.6. Co-interventions

The protocol allows centres to provide their usual 'real-world' care. Balance between groups in centre-related practices should be achieved by minimisation of randomisation by centre. Data will be collected on potential co-interventions, such as: numbers/type of outpatient antenatal visits, hospitalisation or bedrest, home BP monitoring, antihypertensive therapy, maternal blood and urine testing, and tests of fetal well-being.

7. TRIAL TREATMENT / INTERVENTION

7.1 Intervention arm: Planned early term delivery at 38^{+0-3} weeks' gestation

Labour induction or elective Caesareans at 38 weeks will proceed according to local protocols and procedures.

7.2 Control arm: Usual care at term

Usual care is in accordance with the routine practice at individual sites. This will involve maternal and fetal surveillance (clinical, laboratory, and/or ultrasonographic) and management (e.g., antihypertensive therapy), as an integrated package of care based on current NICE NG133 care pathways (usual care). NICE guidelines state that, "…*a consultant or specialist review of the individual case is essential and that a care plan should be developed to include the acceptable thresholds of all monitored variables for each pregnancy*." Clinicians will be asked to follow these guidelines, with clear indication of the parameters that should prompt intervention by delivery for clinical need (e.g.,

refractory severe hypertension or pre-eclampsia(6)).

7.3 Care in both groups

Maternal and fetal surveillance will be recorded on the CRFs. Typically, women are reminded to report, between and at routine antenatal visits, any new symptoms consistent with abruption (abdominal pain, vaginal bleeding, and decreased fetal movement) or possible pre-eclampsia (see Section 3.3).

Any clinical concerns should be investigated by maternal proteinuria testing and blood tests. These tests should include, as a minimum, a complete blood count, serum creatinine, and liver enzymes. Fetal concerns are addressed by measurement of fetal heart rate and pattern and, if indicated, ultrasonographic assessment of growth, amniotic fluid volume, Doppler velocimetry of the umbilical artery, or (less often) biophysical profile.

BP management will be for any maternal BP ≥140/90mmHg, as per NICE guidance (NG133).

Indications for delivery in routine practice are:

For the mother:

(i) sustained systolic BP ≥160mmHg or diastolic BP ≥110mmHg for at least 4 hours despite antihypertensive therapy; or

(ii) development of pre-eclampsia, eclampsia, abruption, or another complication of hypertensive pregnancy (e.g., pulmonary oedema) including abnormal haematologic or biochemical parameters; or

(iii) other obstetric complications.

For the baby:

(i) abnormal cardiotocography;
(ii) intrauterine fetal growth restriction or oligohydramnios (local criteria);
(iii) abnormal umbilical artery Doppler velocimetry; or
(iv) stillbirth

7.4 Accountability Procedures

In order to monitor adherence with the study protocol, the gestational age at birth in both study arms will be monitored. These will be reported back to the sites on a quarterly basis. Any outlier sites may be investigated to determine why their site differs from the norm. This approach has proved to be successful in a trial of BP management in this patient population(9).

7.5 Intervention Modification

We anticipate that, given their underlying hypertensive disease, some women in each of the planned early term delivery (at 38^{+0-3} weeks) and usual care at term groups may be delivered earlier for clinical need. Examples of such indications include development of pre-eclampsia or a maternal end-organ complication associated with hypertensive disease (e.g., pulmonary oedema), or abnormal fetal heart rate or pattern. As per NICE NG133 care pathways (usual care), timing of birth and the maternal and fetal indications for birth "*…should be agreed between the woman and the senior obstetrician*." For women in the usual care at term group, clinicians will be asked to follow these guidelines, with clear indication of the parameters that should prompt intervention by delivery, and these will be recorded in the CRFs. Other reasons for earlier (or later) delivery will also be recorded, such as patient choice or busy hospital induction schedules.

8. OUTCOME MEASURES AND STUDY PROCEDURES

8.1 Co-primary Outcomes

8.1.1 Maternal co-primary outcome

The maternal co-primary outcome is a composite of poor maternal outcome until primary hospital discharge home or 28 days after birth (whichever is earlier) and includes:

• **Systolic BP ≥160mmHg or diastolic BP ≥110mmHg**; or

• **Maternal death**; or

• **Maternal morbidity**, defined as one or more of the following: GCS<13; stroke; TIA; eclampsia; blindness; uncontrolled hypertension; inotropic support; pulmonary oedema; respiratory failure; SpO2 <90%; myocardial ischaemia or infarction; hepatic dysfunction; hepatic haematoma or rupture; acute kidney injury or dialysis; platelet count $<50x10^9/L$; transfusion; or placental abruption. (For definitions, see individual components listed as secondary outcomes, below.) These were adapted from Delphi consensus in hypertensive pregnancy(10;11).

8.1.2 Neonatal co-primary outcome

• **Neonatal care unit admission for ≥ 4 hours**, up to primary hospital discharge home or 28 days of life, whichever is earlier(16). By definition, stillbirths, neonatal deaths without admission, or neonatal deaths with admission for <4 hours are not counted in this outcome, but sensitivity analyses will be conducted (see Section 13.2)).

Neonatal admission is to any of the following types of units, according to definitions provided in the BAPM 2011 classification of neonatal care:

- Intensive care: This is care provided for babies who are the most unwell or unstable and have the greatest needs in relation to staff skills and staff to patient ratios.
- High dependency care: This is care provided for babies who require highly skilled staff but where the ratio of nurse to patient is less than intensive care.
- Special care: Special care is provided for babies who require additional care delivered by the neonatal service but do not require either Intensive or High Dependency care.

This does not include transitional care because the baby is with the mother.

8.2 Secondary Outcomes

8.2.1 Maternal

These will be assessed until primary discharge home or 28 days after delivery, whichever is earlier, unless otherwise specified.

8.2.1.1 Key Maternal

- Caesarean delivery
  - Indications will be presented descriptively

8.2.1.2 Other Maternal

- Instrumental vaginal delivery or Caesarean delivery (vs. spontaneous vaginal delivery)
  - Indications will be presented descriptively (i.e., maternal, fetal, or both, not mutually exclusive)
- Infection of the caesarean wound, episiotomy, or vaginal tear, as applicable will be presented descriptively, assessed at six weeks postpartum
- Individual components of maternal co-primary outcome, up to discharge or 28 days postpartum

(whichever is earlier)

- Systolic BP ≥160mmHg or diastolic BP ≥110mmHg (measured twice, 15min apart) which the national MBRRACE-UK reports (of Confidential Enquiry into Maternal Deaths and Morbidity) states is a clinical emergency requiring urgent treatment
- Maternal death
- Maternal morbidity as adapted from Delphi consensus in hypertensive pregnancy(10;11):
  - GCS<13;
  - Stroke (i.e., acute symptoms of focal brain injury that have lasted over 24 hours, with type [ischaemic or haemorrhage] confirmed by neuroimaging);
  - Transient ischaemic attack (i.e., acute symptoms of focal brain injury that have lasted less than 24 hours);
  - Eclampsia (i.e., the onset of convulsions in a woman with pre-eclampsia not attributable to other causes);
  - Blindness (i.e., partial/complete, or either retinal or cortical). Retinal detachment is defined as the peeling away of the retina from its underlying layer of support tissue diagnosed by ophthalmological exam. Cortical blindness is defined as loss of visual acuity in the presence of intact pupillary response to light;
  - Uncontrolled hypertension (i.e., need for a third parenteral antihypertensive agent (hypertension requiring administration of 3 or more different parenteral [intravenous or intramuscular] antihypertensive agents within a 12 hour period);
  - Inotropic support (i.e, use of vasopressors to keep sBP > 90 mm Hg or a MAP >70 mmHg);
  - Pulmonary oedema (i.e., excess fluid in the lungs diagnosed clinically with one/more of oxygen saturation < 95%, directive treatment (e.g., diuretic therapy), or x-ray confirmation);
  - Respiratory failure (i.e., intubation, ventilation either by endotracheal tube or non-invasively, or need for > 50% oxygen for > 1 hr, none of which is due to Caesarean delivery);
  - SpO2 <90%;
  - Myocardial ischaemia or infarction (i.e., by characteristic ECG changes and markers of myocardial necrosis);
  - Hepatic dysfunction (i.e., INR>1.2 in the absence of disseminated intravascular coagulation (DIC) or treatment with warfarin, OR, in the presence of DIC or treatment with warfarin: either mixed hyperbilirubinemia >1.0 mg/dL (or >17 µM) or hypoglycaemia <45 mg/dL (<2.5 mM) in the absence of insulin);
  - Hepatic haematoma or rupture (i.e., presence of a blood collection under the hepatic capsule as confirmed by imaging or at laparotomy);
  - Acute kidney injury (i.e., serum creatinine >150µM in the absence of a baseline serum creatinine; or rise in serum creatinine ≥26µM within 48 hours, or >50% rise in serum creatinine within the past 7 days; or urine output <0.5ml/kg/hr for >6hr) or new dialysis (of any type);
  - Platelet count <50x10^{9}/L;
  - Transfusion (of any blood product); or
  - Placental abruption, diagnosed either (i) clinically by abdominal pain or uterine contractions of sudden onset with one/more of: vaginal bleeding other than show, intrauterine fetal death or DIC; (ii) by the presence of a retroplacental clot at the time of delivery; or (iii) by placental pathology demonstrating the presence of retroplacental clot or histological findings of a chronic abruption.

- Poor maternal outcome (assessed as one or more of the components [presented descriptively] of the maternal co-primary outcome) measured at six weeks postpartum (as assessed post-discharge after birth by maternal questionnaire, see Section 10.3)

- Elevated liver enzymes (aspartate aminotransferase or alanine aminotransferase >40 IU/L)
- Platelet count <100x10^9/L
- Pre-eclampsia by ISSHP 2018 criteria(67), defined among women with chronic or gestational hypertension, development of one or more of the following new-onset conditions at ≥20 weeks: (i) proteinuria; (ii) serum creatinine ≥90µM; (iii) elevated AST or ALT to >40 IU/L; (iv) neurological complications including eclampsia, altered mental status [as measured by GCS<13], blindness, stroke, clonus, severe headache, persistent visual scotomata); (iv) haematological complications (i.e., platelet count <150x10^9/L, DIC, haemolysis); or (v) uteroplacental dysfunction (including fetal growth restriction defined as birthweight<10th centile(63), abnormal umbilical artery Doppler waveform analysis, or stillbirth)
- PPH (perceived abnormal bleeding following birth and either hypotension or medical/surgical intervention for postpartum haemorrhage)
- Sepsis (known or suspected maternal infection with two or more of Quick Sequential Organ Failure Assessment (qSOFA) criteria: respiratory rate ≥22/min, altered mentation, or systolic BP ≤100mmHg)
- ITU admission (to receive advanced respiratory support alone or monitoring and support for two or more organ systems)
- Potential co-interventions (post-randomisation), before birth admission unless otherwise specified:
    - Antihypertensive therapy taken
        - Antepartum (even after admission for birth)
            - Type of antihypertensive therapy presented descriptively (as labetalol, methyldopa, nifedipine, other [specify])
        - Postpartum
            - Type of antihypertensive therapy presented descriptively (as labetalol, methyldopa, nifedipine, other [specify])
        - Both antepartum and postpartum
    - Magnesium sulphate (antepartum or postpartum)
    - Bedrest at home
    - Use of home BP monitoring
    - Maternal blood or urine testing at the laboratory prior to birth admission, and number of such episodes of testing (median [IQR])
    - Seen as outpatient (in office/clinic) and number of visits (median [IQR])
    - Seen as outpatient (in her home) and number of visits (median [IQR])
    - Where available, seen in medical, day, or maternity assessment unit and number of visits (median [IQR])
    - Seen in an acute care area (such as Accident & Emergency) for urgent/emergent visit other than in labour and number of such visits (median [IQR])
    - Number of antenatal admission days prior to birth (median [IQR])
    - Underwent fetal cardiotocography
    - Underwent fetal ultrasound
- Clinical indications for birth, presented descriptively
- Maternal satisfaction assessed at hospital discharge or 28 days postpartum (whichever is earlier), as measured by the Childbirth Experience Questionnaire, assessed as the overall score, and domain scores (i.e., own capacity, professional support, perceived safety, and participation) (see Section 10.3)

8.2.2 Fetal/Neonatal

- Neonatal care unit admission ≥4 hours assessed to 28 days after birth
- Indication for neonatal care unit admission for ≥4 hours as a respiratory problem, as identified by

the clinical team by the principle indication for admission on the BadgerNet discharge summary (with the clinical diagnosis presented descriptively, as meconium aspiration syndrome, pneumonia, pneumothorax/pneumomediastinum, transient tachypnoea of the newborn, or 'other' [specified])

  - Other indications, as identified clinically, will be presented descriptively (e.g., 5-min Apgar score <7, birthweight <10th centile, birthweight >90th centile, sepsis work-up, hyper- or hypo-glycaemia, or other)

- Respiratory morbidity, defined as the need for supplemental oxygen and/or positive pressure ventilation beyond the initial resuscitation period
- Clinical respiratory problem, defined as meconium aspiration syndrome, pneumonia, pneumothorax/pneumomediastinum, transient tachypnoea of newborn, or other [unspecified])
- Chest x-ray, N performed, N abnormal and nature of abnormality (i.e., meconium aspiration syndrome, pneumonia, pneumothorax/pneumomediastinum, transient tachypnoea of newborn, or other [unspecified])
- HIE, defined as therapeutic hypothermia for ≥72 hours;
- Sepsis requiring antibiotics for at least five days, with confirmed blood or cerebrospinal fluid culture;
- Major operation (laparotomy, thoracotomy, craniotomy, or other)
- Birthweight, birthweight <10th centile (descriptive (63))
- Apgar scores (recorded at 1, 5, and 10 minutes)
- Stillbirth (i.e., death of a fetus after randomisation)
- Neonatal death (of a liveborn infant within the first 28 days of birth)
- Breastfeeding established assessed at hospital discharge or 28 days postpartum (whichever is earlier)
- Exclusive breastfeeding assessed at hospital discharge or 28 days postpartum (whichever is earlier)

8.2.3 Health Economics

- Cost-consequence analysis from NHS perspective (enrolment to hospital discharge)

8.3 Schedule of Assessments

An overview of the scheduled assessments for the WILL trial is given in the Table 3 below.

Women who were consented but not randomised will have data collected at baseline and on maternal and neonatal co-primary outcomes, Caesarean delivery, and other secondary outcomes (other than satisfaction) until primary hospital discharge.

Table 3: Trial participant schedule of events and summary of assessments

VISIT	Antenatal care at < 36^{+0} weeks' gestation	36^{+0} - 36^{+6} weeks' gestation	37^{+0} - 37^{+6} weeks' gestation	38^{+0} weeks' gestation to delivery	Randomisation to delivery	Postnatal (1) delivery to hospital discharge	Postnatal (2) hospital discharge to 6 weeks postpartum†
**Screening***	X	X	X				
**Eligibility check**		X	X				
**Valid informed consent**		X	X				
**Baseline data collection**		X (all consented)	X (all consented)				
**Randomisation‡**			X				
**Weekly contact until birth**				X (all randomised)			
**Outcome data collection**							
Maternal and fetal surveillance					X (all randomised)		
Maternal & neonatal outcomes prior hospital discharge						X (all consented)	
Maternal satisfaction outcome						X (all randomised)	
Maternal & neonatal outcomes that occur after hospital discharge							X (all randomised)
**SAEs** (as defined in Section 9.1)			X (all randomised)	X (all randomised)		X (all randomised)	X (all randomised)

** This will occur over the course of pregnancy. Women will be given a PIS to consider and discuss with their families based on potential eligibility related to chronic or gestational hypertension.*

† 'Poor maternal outcome' will be measured to 6 weeks after birth for the mother, and neonatal unit admission for ≥4 hours will be measured to 28 days for the baby.

‡ This can occur over the telephone if the woman had been screened and consented at $36^{+0\text{-}6}$ weeks' gestation during a face-to-face visit, and the woman is confirmed to remain well, without an indication for delivery (see Section 6.2).

¶ Collected via text messaging or online through Textlocal, or post or telephone, if necessary

8.4 Participant Withdrawal

Informed consent is defined as the process of learning the key facts about a clinical trial before deciding whether or not to participate. It is a continuous and dynamic process and participants should be asked about their ongoing willingness to continue participation. Participants' ongoing assent must be recorded in their medical notes.

Participants should be aware at the beginning of the trial that they can freely withdraw (discontinue participation) from the trial (or part of) at any time, without giving a reason and without implications for their continued healthcare provision.

There are different types of withdrawal and a list of potential examples, are detailed below. Should a participant wish to withdraw from the study, then the reason why needs to be documented clearly in the woman's medical records. If a woman withdraws consent for subsequent data collection, all data collected to that point will be retained unless she explicitly requests redaction of all her data. If she loses capacity during the trial, data until the point of loss of capacity will be retained.

For women who consent to participation but who are NOT randomised:

1. After consent, the woman declines to be randomised;
2. After consent, the woman who did not have the opportunity to be randomised (because she delivered, became ineligible, or it was not possible to randomise her prior to 38^{+0} weeks) has agreed that data can be collected at standard clinic visits and used in the trial analysis, including data collected to date, but she is NOT willing to have data collected from any central UK NHS bodies for long-term outcomes;
3. After consent, the woman who did not have the opportunity to be randomised is NOT willing to have any further data collected from medical records or any central UK NHS bodies for long-term outcomes. She has agreed that data collected prior to the withdrawal can be used in the trial analysis;
4. After consent, the woman who did not have the opportunity to be randomised is NOT willing to have any of her data, including those already collected, used in any future trial analysis.

For women who consent and are randomised:

5. After randomisation, the woman does NOT wish to have follow-up in accordance with the Schedule of Assessments (i.e., receive weekly contact from randomisation until birth from the research midwife, complete the Childbirth Experience Questionnaire, and/or complete the 6-week postpartum questionnaire sent by text message), but she has agreed that data can be collected at standard clinic visits and used in the trial analysis, including data collected to date and those from any central UK NHS bodies for long-term outcomes;
6. After randomisation, the woman does NOT wish to have follow-up in accordance with the Schedule of Assessments (i.e., receive weekly contact from randomisation until birth from the research midwife, complete the Childbirth Experience Questionnaire, and/or complete the 6-week postpartum questionnaire sent by text message). She has agreed that data can be collected at standard clinic visits and used in the trial analysis, including data collected to date; however, she has NOT agreed to use of data from any central UK NHS bodies for long-term outcomes;
7. After randomisation, the woman is NOT willing to either be followed up in any way for the purposes of the trial, or have any further data collected from medical records or any central UK NHS bodies for long-term outcomes. She has agreed that data collected prior to the withdrawal can be used in the trial analysis; or
8. After randomisation, the woman would like to withdraw completely from all follow-up. She is NOT willing to have any of her data, including those already collected, used in any future trial analysis.

The following details of withdrawal should be clearly documented on the CRF and medical notes and

a **Trial Withdrawal Form** should be completed, which includes:

1. The date the woman withdrew consent;
2. The reason, if given; and
3. Type of withdrawal, from the definitions above.

9. ADVERSE EVENT REPORTING

9.1 Definitions

The definitions of adverse events are given in the Table 4. **Table 4**: General definitions for adverse events

Term	Definition
Adverse Event (AE)	**Any untoward medical occurrence in a trial participant, which is identified as having begun at any point between randomisation and 6 weeks postpartum, and does not necessarily have a causal relationship with the intervention.**
Serious Adverse Event (SAE)	**Any AE that:**<br>• **results in death;**<br>• **is life-threatening*;**<br>• **requires hospitalisation or prolongation of existing hospitalisation (with exceptions†);**<br>• **results in persistent or significant disability or incapacity; or**<br>• **may jeopardise the pregnancy or may require intervention to prevent one of the other outcomes listed above.**
Related Event (AE or SAE)	**An event (AE or SAE) which resulted from the administration of any of the research procedures.**
Protocol-exempt SAE	**A SAE that is listed in the protocol as not requiring reporting on a separate SAE form.†**
Expeditable SAE	**A SAE that requires reporting on a SAE form.**
Unexpected SAE	**A SAE that is not listed in the protocol as an expected occurrence.‡**
Unexpected and Related SAE	**A SAE that meets the definition of both an Unexpected SAE and a Related SAE**
*** Life-threatening in the definition of a SAE refers to an event in which the mother was at risk of death at the time of the event. It does not refer to an event which hypothetically might have caused death if it were more severe.**<br>***† Some SAEs are 'protocol-exempt' SAEs because they are either expected given the high-risk nature of WILL participants, or unrelated to the WILL intervention.***<br>***‡ See Section 9.3.2.***	

9.2 Reporting Requirements

Due to the high incidence of adverse events routinely expected in this patient population (e.g.

abnormal laboratory findings and new symptoms), only those adverse events identified as serious will be recorded for the trial.

9.3 Serious Adverse Events (SAEs) Requiring Reporting in WILL

Due to the high incidence of SAEs anticipated in the clinically high-risk population of women to be enrolled in WILL, most are documented on the CRFs. Only SAEs specified in Section 9.3.2 are reportable on the SAE Form to the the WILL Team at the BCTU.

The Investigator should document in the source data, all SAEs experienced by the trial participant, from randomisation until 6 weeks postpartum; all SAEs must be followed to their resolution, even if this extends the time frame beyond 6 weeks postpartum. Specific comment must be made about the causality (relatedness) of the SAE with reference to the protocol. This is all that is required for protocol-exempt SAEs which do not require reporting to the WILL Trial Office at the BCTU. However, expeditable SAEs must be reported to the WILL Trial Office at the BCTU on the SAE Form.

9.3.1 Protocol-exempt SAEs do NOT require reporting on a SAE form

The following SAEs are protocol-exempt, expected SAEs as a consequence of the high-risk nature of the patient population enrolled in WILL. These events are pre-specified outcomes and are all captured on the CRFs, as components of either the primary or secondary outcomes. While these events should still be recorded in medical notes, they do NOT require completion of a SAE form and they do NOT require reporting to the WILL trial office:

- Maternal events
    - Severe hypertension;
    - Maternal morbidity: GCS; TIA; eclampsia; blindness; uncontrolled hypertension; inotropic support; pulmonary oedema; respiratory failure; SpO2 <90%; myocardial ischaemia or infarction; hepatic dysfunction, hepatic haematoma or rupture; acute kidney injury; or transfusion;
    - Pre-eclampsia;
    - PPH;
    - Lower genital tract bleeding;
    - Sepsis;
    - Admission to hospital for pre-eclampsia, monitoring of hypertension, or cervical ripening or induction of labour; or
    - Antenatal admission for assessment for suspected fetal compromise, including poor growth, or reduced fetal movements.
- Newborn events
    - Abnormal Doppler velocimetry of the umbilical artery;
    - Meconium-staining of the amniotic fluid or placenta;
    - Low birthweight or SGA infants;
    - Neonatal care unit admission;
    - Requirement for supplemental oxygen or ventilatory support;
    - Seizures;
    - HIE;
    - Sepsis;
    - Major operation;
    - Hypoglycaemia; or
    - Hypothermia.

The following SAEs are protocol exempt, unrelated SAEs because the serious nature of the event is related to the woman's routine care. These events do NOT require reporting on the SAE form or to the WILL trial office:

- Pre-planned hospitalisation;
- Diagnostic and therapeutic procedures, such as Caesarean delivery;
- Consequences of diagnostic and therapeutic procedures unrelated to timing of delivery (i.e. urinary tract infection; UTI);
- Worsening pruritis;
- A pre-existing maternal condition (such as renal disease), unless it causes increased clinical concern;
- Admission for psychiatric or social reasons;
- Antenatal admission for monitoring for antepartum haemorrhage, suspected preterm labour, pre-labour rupture of the membranes, or other reasons for enhanced maternal surveillance;
- Admission for unstable lie or external cephalic version;
- Admission in active labour, whether at term or preterm;
- Admission for Caesarean delivery;
- Retained placenta;
- Extended hospital stay of the mother due to the need to keep her baby in hospital;
- Neonatal care unit admission for indications unrelated to pregnancy hypertension, such as neonatal hyperbilirubinaemia or unanticipated care for a fetal anomaly; or
- Fetal congenital anomaly, as the intervention is given towards the end of labour beyond 38 weeks' gestation where it cannot have any possible teratogenic effect.

All other SAEs are considered expeditable and must be reported on a WILL SAE Form.

9.3.2 Expeditable SAEs require expedited reporting on the SAE Form

All SAEs other than those listed in Sections 9.3.1 (as protocol-exempt), are considered to be expeditable and require reporting on the SAE Form to the WILL trial office. This must be done by the PI (or delegate) within 24 hours of the site becoming aware of the event.

Expeditable SAEs include, but are not limited to, the list below. While it is known that maternal, fetal, and newborn death, as well as maternal stroke can, and do, occur in association with chronic or gestational hypertension, these events are still rare and it is important to assess their relatedness to the intervention.

- Maternal death
- Maternal stroke
- Stillbirth
- Neonatal death

9.4 Reporting Procedures and Follow-up at Site

9.4.1 Reporting procedure for Serious Adverse Events by sites

On becoming aware that a participant has experienced an expeditable SAE (Section 9.3.2), the PI or delegate(s) should report the expeditable SAE to: (i) their own Trust in accordance with local practice, and (ii) the WILL trial office at BCTU. This must be done within 24 hours of the Investigator or delegate becoming aware of the event.

To report an expeditable SAE to the WILL trial office, the PI or delegate(s) must:

(i) complete the WILL SAE form by logging onto the WILL trial database; and

(ii) send any additional relevant information, appropriately anonymised, to the WILL trial team at **WILL@trials.bham.ac.uk** within **24 hours** of first becoming aware of the event.

On receipt of the SAE form, the WILL database will allocate each a SAE reference number. The site and

the WILL trial office should ensure that the SAE reference number is quoted on all correspondence and follow-up reports regarding the SAE and filed with the SAE in the Site File.

9.4.2 Assessment of Causality (Relatedness) by the PI

When completing the SAE form, the PI will be asked to define the nature of the seriousness and causality (relatedness; see Table 5) of the event. In defining the causality the PI must consider if any concomitant events or medications may have contributed to the event and, where this is so, these events or medications should be reported on the SAE form. It is not necessary to report concomitant events or medications which did not contribute to the event. As per Table 5 below, all events considered at the site to be 'possibly', 'probably', or 'definitely' related to the intervention will be reported by the WILL trial office as 'related'; all events considered at site to be 'unlikely' or 'unrelated' to the intervention will be reported by the WILL trials office as 'unrelated'. The same categorisation should be used when describing AEs and protocol-exempt SAEs in the source data.

Table 5: Categorisation of causality (relatedness) for AEs and SAEs

Category	Definition	Causality
**Definitely**	There is clear evidence to suggest a causal relationship, and other possible contributing factors can be ruled out	**Related**
**Probably**	There is evidence to suggest a causal relationship, and the influence of other factors is unlikely	
**Possibly**	There is some evidence to suggest a causal relationship (e.g., the event occurred within a reasonable time after the intervention was started). However, the influence of other factors may have contributed to the event (e.g., the patient's clinical condition, other concomitant events)	
**Unlikely**	There is little evidence to suggest there is a causal relationship (e.g., the event did not occur within a reasonable time after the intervention was started). There is another reasonable explanation for the event (e.g., the patient's clinical condition, other concomitant treatments)	**Unrelated**
**Unrelated**	**There is no evidence of any causal relationship**	

9.4.3 Provision of follow-up information

Following reporting of a SAE, the woman should be followed up until resolution or stabilisation of the event. Follow-up information should be provided using the SAE reference number provided by the WILL database. Once the SAE has been resolved, all critical follow-up information has been received and the paperwork is complete, a copy of the original SAE form must be kept in the Site File.

9.5 Reporting Procedures by the WILL Trial Office

On receipt of a SAE Form from the site, the WILL trial office will forward a notification email, with the unique reference number, to the CI or delegate(s) who will log into the WILL database and independently categorise the causality of the SAE, using the same criteria as outlined in Section 9.4.2. The causality assessment given by the PI will not be downgraded by the CI or delegate(s); if the CI or delegate(s) disagrees with the PI's causality assessment, the opinion of both parties will be documented, and where the event requires further reporting, the opinion of both will be provided with the report.

9.5.1 Assessment of Expectedness by CI

The CI or delegate(s) will also assess all expedited SAEs for expectedness with reference to the criteria

provided in Table 6. The CI may request further information from the clinical team at site. This information should be made available immediately upon request. The CI will not overrule the severity or causality assessment given by the PI, but the CI may add additional comments. If the SAE is confirmed to be unexpected (i.e., is not defined in the protocol as an expected event, as in Section 9.3.1), it will be classified as an Unexpected and Related SAE.

Table 6: Definition of expectedness for SAEs

Category	Definition
Expected	**A SAE that is classed in nature as serious and is consistent with the list of expected SAEs defined in the protocol.**
Unexpected	**A SAE that is classed in nature as serious and which is inconsistent with the list of expected SAEs defined in the protocol.**

9.5.2 Reporting SAEs to third parties

If any Unexpected and Related SAEs occur, BCTU will report them to the PI, CI, main REC, and Sponsor within 15 days; a copy of any such correspondence will be filed in the ISF and TMF. In addition, if an additional, significant safety issue is identified during the course of the trial, BCTU will notify the PI, CI, main REC, and Sponsor immediately; a copy of any such correspondence will be filed in the ISF and TMF.

The independent DMC for the WILL trial will review SAEs at their meetings.

9.6 Urgent Safety Measures

If any urgent safety measures must be taken by the BCTU, the Unit shall immediately, and in any event no later than three days from the date the measures are taken, give written notice to the REC of the measures taken and the circumstances giving rise to those measures. The timelines for the above and other measures with regards to safety reporting are summarised in Table 7.

Table 7: Urgent safety measures

Safety Measure	Who	When	How	To Whom
**SAE**	BCTU	Report to Sponsor within 24 hours of learning of the event	SAE Report form for Non-CTIMPs, available from NRES website.	Sponsor and MREC
		Report to the MREC within 15 days of learning of the event		
**Urgent Safety Measures**	BCTU	Contact the Sponsor and MREC Immediately	By phone	Main REC and Sponsor
		Within 3 days	Substantial amendment form giving notice in writing setting out the reasons for the urgent safety measures and the plan for future action	Main REC with a copy also sent to the sponsor. The MREC will acknowledge this within 30 days of receipt

Safety Measure	Who	When	How	To Whom
**Progress Reports**	BCTU	Annually (starting 12 months after date of favourable opinion)	Annual Progress Report Form (non-CTIMPs) available from the NRES website	Main REC
**Declaration of the conclusion or early termination of the study**	BCTU	Within 90 days (conclusion)	End of Study Declaration form available from the NRES website	Main REC with a copy to be sent to the sponsor
		Within 15 days (early termination)		
		The end of study should be defined in the protocol		
**Summary of final Report**	CI	Within one year of conclusion of the Research	No Standard Format. However, the following Information should be included: where the study has met its objectives, the main findings and arrangements for publication or dissemination, including feedback to participants	Main REC with a copy to be sent to the sponsor

10. DATA HANDLING AND RECORD KEEPING

10.1 Source Data

In order to allow for the accurate reconstruction of the trial and clinical management of the woman, source data will be accessible and maintained. The source date for all data other than the maternal questionnaire will be the woman's medical notes and the neonatal notes. The maternal questionnaire is source data, being a participant reported outcome, which will be stored at site or University of Birmingham.

10.2 Case Report Form (CRF) Completion

Data reported on each form will be consistent with the source data and any discrepancies will need to be clarified by site staff. All missing and ambiguous data will be queried by the BCTU staff with site staff. Staff delegated to complete CRFs will be trained to adhere to procedures for:

- CRF completion and corrections;
- Date format and partial dates;
- Time format and unknown times;
- Rounding conventions;
- Trial-specific interpretation of data fields;
- Entry requirements for concomitant medications (generic or brand names);
- Which forms to complete and when;
- What to do in certain scenarios, for example when a woman withdraws from the trial;
- Missing/incomplete data;

- Completing SAE forms and reporting SAEs; and
- Protocol and GCP non-compliances.

In all cases it remains the responsibility of the site's PI to ensure that the CRF has been completed correctly and that the data are accurate.

Participant Completed Questionnaires

Women will be asked to complete two questionnaires:

Satisfaction with care: Women's satisfaction with the interventions and trial participation will be evaluated according to the Childbirth Experience Questionnaire (CEQ), a 22-item self-administered questionnaire that has been validated in the UK(64). Higher scales reflect greater satisfaction, as do higher domain scores that cover own capacity (8 items), professional support (5 items), perceived safety (6 items), and participation (3 items). If the questionnaire cannot be completed prior to hospital discharge for whatever reason, it will be self-administered at home, or administered by research staff either over the phone or in person at a postnatal medical appointment.

Post-discharge maternal morbidity (to six weeks postpartum) or neonatal morbidity (to 28 days after birth): Such morbidity will be collected directly from the mother for her and her baby, unless *either* is known to have died, *both* are known to have experienced the primary outcome prior to hospital discharge, or the *mother* has become incapacitated to such an extent that she is unable to complete the questionnaire. We have modified the relevant CHIPS questionnaire, previously NRES-approved (2009-12), for administration by text message or online through TextLocal (*www.textlocal.com*), or by post or by phone, if necessary.

In order for Textlocal to contact the participant, Textlocal will be sent the woman's mobile telephone number. So that we can link the responses given by the woman back to her record on the study database Textlocal will also be given the woman's study number. No other information about the participant or her baby will be given to Textlocal. The woman's study number, telephone number and responses will be encrypted whilst being stored by Textlocal, and these data will not be used by Textlocal for any other purpose. Once the responses have been transferred from Textlocal to the study database held at the University of Birmingham, Textlocal will securely delete all of the study data that they hold.

If we are unable to contact mothers directly, information will be requested of the GP, if consent to do so was provided.

10.3 Data Management

Processes will be employed to ensure the accuracy of the data included in the final report. These processes will be detailed in the trial specific data management plan. Coding and validation will be agreed between the trial manager, statistician and programmer and the trial database will be signed off once the implementation of these has been agreed.

Electronic CRFs will be entered online at www.trials.bham.ac.uk/WILL. Authorised staff will require an individual secure login username and password to access this online data entry system. Those entering data will receive written work instructions on the process (a copy of which should be filed in the ISF and TMF). CRFs should be filed within the ISF.

If changes need to be made to a CRF that has already been entered and submitted on to the database, the site should contact the WILL trial office so that the form can be unlocked for them and an explanation of the errors entered.

Data reported on each CRF should be consistent with the source data or the discrepancies should be explained. If information is unknown, this must be clearly indicated on the CRF. Completed questionnaires will be reviewed by the WILL trial office for completeness. All missing and

ambiguous data will be queried. An online data base system will be used to generate any data clarification forms. These will be generated on a regular basis by WILL trial office staff and reported to the site for clarification within 28 days. The process of entering data on to the database, itself forms a data quality check, as ranges are put in place to ensure that only viable data values can be input. It will be the responsibility of the Principal Investigator to ensure the accuracy of all data entered in the CRFs on behalf of their site. The **Site Signature and Delegation Log** will identify all those personnel with responsibilities for data collection

Questionnaires completed remotely by the women will be received by BCTU and will be transcribed directly onto the database. Given that these are patient-reported outcomes, a data query process cannot be implemented.

Self-evident corrections by the WILL trial office are not permitted.

CRFs may be amended and the versions updated by the WILL trial office, as appropriate, throughout the duration of the trial.

10.4 Data Security

The security of the System is governed by the policies of the University of Birmingham. The University's Data Protection Policy and the Conditions of Use of Computing and Network Facilities set out the security arrangements under which sensitive data should be processed and stored. All studies at the University of Birmingham have to be registered with the Data Protection Officer and data held in accordance with the Data Protection Act. The University will designate a Data Protection Officer upon registration of the study. The Study Centre has arrangements in place for the secure storage and processing of the study data which comply with the University of Birmingham policies.

The System incorporates the following security countermeasures:

- Physical security measures: restricted access to the building, supervised onsite repairs and storages of back-up tapes/disks are stored in a fire-proof safe.
- Logical measures for access control and privilege management: including restricted accessibility, access-controlled servers, separate controls used non-identifiable data etc.
- Network security measures: including site firewalls, antivirus software, separate secure network protected hosting etc.
- System Management: the System shall be developed by the BCTU Programming Team and will be implemented and maintained by the BCTU Programming Team.
- System Design: the system shall comprise of a database and a data entry application with firewalls, restricted access, encryption and role-based security controls.
- Operational Processes: the data will be processed and stored within the Study Centre (University of Birmingham).
- Data processing: Statisticians will have access to anonymised data.
- System Audit: The System shall benefit from the following internal/external audit arrangements:
- Internal audit of the system
- Periodic IT risk assessments
- Data Protection Registration: The University of Birmingham has Data Protection Registration to cover the purposes of analysis and for the classes of data requested. The University's Data Protection Registration number is Z6195856.

10.5 Archiving

Archiving will be authorised by BCTU on behalf of the Sponsor following submission of the end of trial report.

It is the responsibility of the Principal Investigator to ensure all essential trial documentation and

source documents (e.g. signed Informed Consent Forms, ISFs, women's hospital notes, copies of CRFs) at their site are securely retained as per their NHS Trust policy, for at least 25 years after completion of the trial.

Destruction of essential documents will require authorisation from the BCTU on behalf of the Sponsor.

11. QUALITY CONTROL AND QUALITY ASSURANCE

11.1 Site Set-up and Initiation

The Statement of Activities/Organisation Information Document will act as the site agreement and is required to be signed by the co-applicants' institutions, confirming their intention to regulate their rights and obligations in accordance with the terms and conditions for the WILL trial. A collaborators agreement, including a delegation of duties agreement, is required to be signed by the Co-sponsors and UoB to document the expectations of parties, the CI and any third parties. The collaboration agreement and delegation documents must be completed prior to the start of the trial. In addition all local PIs will be asked to sign the necessary agreements including a **Site Signature and Delegation log** between the PI and BCTU and supply a current ink signed and dated CV and current GCP certificate to BCTU. All members of the site research team are required to sign the **Site Signature and Delegation Log**, which details which tasks have been delegated to them by the PI.

Prior to commencing recruitment, each recruiting site will undergo a process of initiation, either by a meeting or a teleconference, which key members of the site research team are required to attend. This initiation meeting will cover aspects of the trial design, protocol procedures, adverse event reporting, collection and reporting of data and record keeping. Sites will be provided with an Investigator Site File containing essential documentation, instructions, and other documentation required for the conduct of the trial. The WILL trial office must be informed immediately of any change in the site research team.

11.2 Monitoring

Monitoring is necessary to ensure both the safety of participants and the credibility of the data. Monitoring can be performed either by visiting the trial site(s) ('on-site monitoring') which affords access to source documents, or by centralised monitoring. The risk assessment should be performed to identify the risks and how these can be mitigated through either on-site or centralised monitoring, or a combination of the two. The risk assessment should inform a trial monitoring plan. Findings generated from monitoring should be shared with local R&D departments who may have plans to perform quality checks on the same trial.

11.3 Onsite Monitoring

Monitoring is carried out as required following trial specific risk assessment and as documented in the monitoring plan. The monitoring plan should be approved by the QA Manager before it is implemented. The number of sites to be monitored and the basis for selecting those sites for this trial will be specified in the trial monitoring plan. Any monitoring activities will be reported to the trials team and any issues noted will be followed up to resolution. Additional on-site monitoring visits may be triggered, for example by poor CRF return, poor data quality, low SAE reporting rates, excessive number of participant withdrawals or deviations. If a monitoring visit is required, the WILL trial team will contact the site to arrange a date for the proposed visit and will provide the site with written confirmation. Investigators will allow the WILL trial staff access to source documents and the ISF as requested. The monitoring will be conducted by BCTU.

11.4 Central Monitoring

WILL Trial staff will be in regular contact with the site research team to check on progress and address

any queries that they may have. WILL Trial staff will check incoming ICFs for compliance with the protocol, data consistency, missing data and timing. Sites will be sent DCFs requesting missing data or clarification of inconsistencies or discrepancies.

Source data can be requested for the purpose of central monitoring (e.g. for checking eligibility or endpoints). If such source data is requested, documents should be redacted and labelled with the participant's trial specific ID number.

11.5 Audit and Inspection

The Investigator will permit trial-related monitoring, audits, ethical review, and regulatory inspection(s) at their site, providing direct access to source data and/or documents. The investigator will comply with these visits and any required follow up. Sites are also requested to notify BCTU of any relevant inspections.

11.6 Notification of Serious Breaches

The sponsor is responsible for notifying the REC of any serious breach of the conditions and principles of GCP in connection with that trial or the protocol relating to that trial. Sites are therefore requested to notify BCTU of any suspected trial-related serious breach of GCP and/or the trial protocol. Where BCTU is investigating whether or not a serious breach has occurred sites are also requested to cooperate with BCTU in providing sufficient information to report the breach to the REC where required and in undertaking any corrective and/or preventive action.

Sites may be suspended from further recruitment in the event of serious and persistent non-compliance with the protocol and/or GCP, and/or poor recruitment. Any major problems identified during monitoring may be reported to trial specific committees and/or stakeholders (e.g. Trial Management Group, Trial Steering Committee, the Sponsor), and the REC. This includes reporting serious breaches of GCP and/or the trial protocol to the REC.

12. END OF TRIAL DEFINITION

The end of trial will be five months after the last woman is recruited, based on the time needed for: women to deliver (up to five weeks after consent), follow-up to six weeks postpartum for maternal morbidity (six weeks), and data collection and cleaning (up to another eight weeks). The WILL trial office will notify the main REC and the Sponsor that the trial has ended and a summary of the clinical trial report will be provided within 12 months of the end of trial.

13. STATISTICAL CONSIDERATIONS

13.1 Sample Size

A total sample size of 1,080 women (540 per group) will be required to detect an 8% reduction in the maternal co-primary outcome from 25% to 17% (RR 0.68; estimate of 25% based on women who experienced poor maternal outcome at term in the CHIPS Trial(9) [unpublished data]), assuming 90% power, a two-sided type I error rate of 5%, using the standard method of difference between proportions and based on a superiority hypothesis.

Assuming a control group (usual care at term) incidence of our neonatal co-primary (safety) outcome (of neonatal care unit admission ≥4 hrs) of 23%, a sample size of 1,080 will achieve 94% power to provide a non-inferiority margin of difference in incidence between groups of 9% (i.e., the upper bound of the 95% CI is <9%), and 88% power to provide a margin of 8% (one-sided 2.5% type I error rate, non-inferiority hypothesis).

This sample size will detect a 10% decrease in Caesarean delivery assuming a control group risk of 45% (45% to 35%; 90% power; 5% type I error rate, superiority hypothesis), similar to changes in HYPITAT I. In this way, women and clinicians will have the information that they require (about complications for them and their babies, and Caesarean delivery) in order to make informed decisions about care.

There is no single minimum clinically important difference in maternal or perinatal outcomes that is likely to influence all clinicians. The anticipated relative risk reduction in our maternal co-primary outcome was chosen because a similar reduction was seen in HYPITAT I, and this effect size was shown to be of sufficient magnitude to change clinical practice in the Netherlands (51). The incidence in the control group of our neonatal co-primary (safety) outcome of high-level neonatal care for ≥4hr is based on a rate of 23% in HYPITAT. We have reasonable power to detect a non-inferiority margin of difference in the incidence of neonatal care unit admission ≥4 hrs as small as 8 to 9%.

Given the short time between consent (at 36^{+0}-37^{+6} weeks), randomisation (at $37^{+0\text{-}6}$ weeks), and birth (by 41^{+6} weeks, even in the usual care arm), no adjustment has been made for loss to follow-up or drop-outs.

13.2 Analysis of Outcome Measures

A separate Statistical Analysis Plan will be produced and will provide a more comprehensive description of the planned statistical analyses. A brief outline of these analyses is given below.

The primary comparison groups will be composed of women or babies of women randomised to planned early term delivery at 38^{+0} to 38^{+3} weeks versus those randomised to usual care at term. In the first instance, all analyses will be based on the ITT principle (i.e., all participants will be analysed in the treatment group to which they were randomised irrespective of compliance or other protocol deviation). Although the neonatal co-primary outcome is based on a non-inferiority hypothesis, an ITT analysis (rather than a per-protocol analysis) was considered the most appropriate primary analysis since the intervention is about planned timing of delivery (as opposed to actual timing of delivery). A sensitivity analysis will be performed on the per-protocol population to assess the robustness of the findings (see Section 13.2.4).

For all outcome measures, summary statistics and treatment effects, (e.g. mean differences, relative risks) will be presented. Treatment effects will be adjusted for the minimisation variables listed in Section 6.2, where possible(58). 95% confidence intervals (CI) will be presented for all outcomes. No adjustment for multiple comparisons will be made.

Women who are consented at 36^{+0}-37^{+6} weeks, but who were not randomised (possible only at $37^{+0\text{-}6}$ weeks) will be included in separate descriptive analyses of the maternal and neonatal co-primary outcomes, Caesarean delivery, and other secondary outcomes (other than satisfaction) to hospital discharge only.

13.2.1 Primary Outcome Measures

The co-primary outcomes are binary outcomes (i.e. yes/no) and will be analysed using a generalised linear model (with binomial distribution and log link), adjusting for minimisation variables listed in Section 6.2. Treatment effects will be expressed as adjusted risk ratios with 95% CIs. If the model does not converge, log Poisson regression models with robust variance estimation will be used (59).

13.2.2 Secondary Outcome Measures

The secondary outcomes that are binary (i.e., yes/no) will be analysed using the same methods described for the primary outcomes (see Section 13.2.1), with corresponding 95% CIs. For those secondary outcomes that are continuous (e.g. the Childbirth Experience Questionnaire scores), linear regression methods will be used if the outcome is sufficiently normally distributed (or where data can be suitably transformed), to calculate an adjusted mean difference and 95% confidence interval. For skewed continuous outcomes, unadjusted median differences and 95% confidence intervals will be

presented.

13.2.3 Subgroup Analyses

Subgroup analyses will be undertaken on: (i) variables used in the minimisation algorithm other than centre (i.e. hypertension type [chronic or gestational hypertension] and prior Caesarean [yes/no], see Section 6.2); and (ii) other variables of prognostic significance pre-specified as ethnicity, BMI, prior severe hypertension in the index pregnancy, antihypertensive therapy at randomisation, gestational diabetes mellitus at randomisation, and smoking status at randomisation. Subgroup analyses will be limited to the co-primary outcomes. Tests for statistical heterogeneity (e.g. by including the treatment group by subgroup interaction parameter in the regression model) will be performed prior to any examination of effect estimate within subgroups. Results will be presented as adjusted risk ratios with 95% confidence intervals. The results of subgroup analyses will be treated with caution and will be used for the purposes of hypothesis generation only.

13.2.4 Missing Data and Sensitivity Analyses

Every attempt will be made to collect full follow-up data on all study participants; it is thus anticipated that missing data will be minimal. Participants with missing primary outcome data will not be included in the primary analysis in the first instance. This presents a risk of bias, and sensitivity analyses will be undertaken to assess the possible impact of the risk. This will include a multiple imputation approach. Full details will be included in the Statistical Analysis Plan.

To examine the robustness of the conclusions, sensitivity analyses will be undertaken for: (i) the two co-primary outcomes, whereby women will be excluded if they were both randomised and delivered before 38^{+0} weeks (prior to when the intervention could be delivered); (ii) the two co-primary outcomes, whereby women will be excluded if their timing of delivery was not as per protocol (such as expectant care beyond $38^{+0\text{-}3}$ weeks in the intervention group); (iii) the neonatal co-primary outcome among liveborns, and (iv) the neonatal co-primary outcome including babies who either died without admission to neonatal care, or died following admission to neonatal care for <4 hours, where newborn death is of a liveborn infant until primary discharge home or within the first 28 days of birth, whichever is earlier.

We will also perform a range of exploratory analyses, such as investigation of the impact of including severe hypertension as part of the maternal co-primary composite outcome on the interpretation of our results.

13.3 Planned Interim Analysis

Interim analyses for efficacy and safety for presentation to the independent DMC will take place during the study. The committee will meet prior to study commencement to agree the manner and timing of such analyses, but this is likely to include full assessment of safety (SAEs) at least at annual intervals. Criteria for stopping or modifying the study based on this information will be ratified by the DMC. Details of the agreed plan will be written into the Statistical Analysis Plan. Further details of DMC arrangements are given in Section 14.6.

13.4 Planned Final Analyses

The primary analysis for the study will occur once all women have completed the 6 week postpartum assessment and corresponding outcome data have been entered onto the study database and validated as being ready for analysis. This analysis will include data items up to and including the 6-week assessment and no further.

14. TRIAL ORGANISATIONAL STRUCTURE

14.1 Funder

The National Institute for Health Research is funding the WILL trial through their Health Technology Assessment funding stream, which was awarded following a competitive two stage application and review process.

14.2 Sponsor

King's College London will act as sponsor for the WILL trial, taking overall responsibility for the initiation and management of the trial, and oversight of financing. Given the trial will be conducted within the NHS, Guy's and St. Thomas' NHS Foundation Trust will act as co-sponsor.

14.3 Coordinating Centre

Birmingham Clinical Trials Unit (BCTU) is responsible for the management of the sites. This includes providing all trial materials, including the trial folders containing printed materials and the update slides. These will be supplied to each collaborating centre, after relevant R&D approval has been obtained. Additional supplies of any printed material can be obtained on request. BCTU will provide the central randomisation service and is responsible for collection and checking of data (including reports of SAEs thought to be due to trial interventions), for reporting of serious and unexpected adverse events to the Sponsor and/or the Research Ethics Committee and for analyses. BCTU will facilitate collaborating centres to resolve any local problems that may be encountered in trial participation.

14.4 Trial Management Group

The Trial Management Group (TMG) should include those individuals responsible for the day-to-day management of the trial, such as the CI, senior statistician, trial statistician, team leader, trial manager, research midwife, data manager. The role of the group is to monitor all aspects of the conduct and progress of the trial, ensure that the protocol is adhered to and take appropriate action to safeguard participants and the quality of the trial itself. TMG will meet monthly at face-to-face meetings.

14.5 Trial Steering Committee

The role of the Trial Steering Committee (TSC) is to provide the overall supervision of the trial. The TSC includes members who are independent of the investigators, their employing organisations, funders and sponsors. The TSC will operate in accordance with a trial specific charter and will meet at least annually. The TSC should monitor trial progress and conduct and provide advice on scientific credibility of the WILL trial. The TSC will consider and act, as appropriate, upon the recommendations of the Data Monitoring Committee (DMC) or equivalent and ultimately carries the responsibility for deciding whether a trial needs to be stopped on grounds of safety or efficacy.

14.6 Data Monitoring Committee

An independent data-monitoring committee will be established for the sponsor to assess at intervals the progress of a clinical trial, the safety data, and the critical efficacy endpoints, and to recommend to the sponsor whether to continue, modify, or stop a trial.

Data analyses will be supplied in confidence to an independent Data Monitoring Committee (DMC), which will be asked to give advice on whether the accumulated data from the trial, together with the results from other relevant research, justifies the continuing recruitment of further participants. The DMC will operate in accordance with a trial specific charter based upon the template created by the Damocles Group. The DMC will be scheduled to meet annually (e.g., one year after the trial opens to recruitment and then annually thereafter until the trial closes to recruitment). The DMC will meet annually unless there is a specific reason (e.g. safety phase) to amend the schedule.

Additional meetings may be called if recruitment is much faster than anticipated and the DMC may, at their discretion, request to meet more frequently or continue to meet following completion of recruitment. An emergency meeting may also be convened if a safety issue is identified. The DMC will report directly to the Trial Steering Committee who will convey the findings of the DMC to the Trial Management Group, Sponsors and funders.

The DMC may consider recommending the discontinuation of the trial if the recruitment rate or data quality are unacceptable or if any issues are identified which may compromise participant safety. The trial will stop early if the interim analyses showed differences between interventions that were deemed to be convincing to the clinical community. The trial stopping rules will be outlined in the DMC charter.

14.7 Co-investigator Group

The Co-investigator Group (CiG) is an extended TMG and will meet every 3 months initially, then every 3-4 months to review progress, troubleshoot and plan strategically. The CiG consists of all members of the co-applicant group and representatives of the PPI Working Group.

14.8 Finance

This is a researcher led trial funded by the NIHR. The grant will be administered by the Kings College London. The Clinical Research Network will automatically adopt the WILL trial onto the NIHR portfolio, which will entitle the WILL trial to CRN support.

15. ETHICAL CONSIDERATIONS

The trial will be performed in accordance with the recommendations guiding physicians in biomedical research involving human subjects, adopted by the 18th World Medical Association General Assembly, Helsinki, Finland, 1964, amended by the 48th WMA General Assembly, Somerset West, Republic of South Africa, 1996 (website: *http://www.wma.net/en/30publications/10policies/b3/index.html*).

The trial will be conducted in accordance with the UK Policy Framework for Health and Social Care Research, the applicable UK Statutory Instruments, (which include the General Data Protection Regulation) and the Principles of GCP. The protocol will be submitted to and approved by the main REC prior to circulation.

Before any participants are enrolled into the trial, the PI at each site will obtain local R&D approval. Sites will not be permitted to enrol participants until written confirmation of R&D approval is received by the WILL trial office.

It is the responsibility of the PI to ensure that all subsequent amendments gain the necessary local approval. This does not affect the individual clinicians' responsibility to take immediate action if thought necessary to protect the health and interest of individual participants.

16. CONFIDENTIALITY AND DATA PROTECTION

Personal data recorded on all documents will be regarded as strictly confidential and will be handled and stored in accordance with the 2018 General Data Protection Regulations and other relevant legislation.

Participants will always be identified using their unique trial identification number, last four digits of their NHS number, and month and year of birth on the CRF and correspondence between BCTU and the trial site. As they contain identifiable information, women will give their explicit consent for the movement of their consent and randomisation form to BCTU.

The Investigator must maintain documents not for submission to BCTU (e.g. Participant Identification Logs) in strict confidence. In the case of specific issues and/or queries from the regulatory authorities, it will be necessary to have access to the complete trial records, provided that participant confidentiality is protected.

BCTU will maintain the confidentiality of all participants' data and will not disclose information by which participants may be identified to any third party, other than those directly involved in the treatment of the participant and organisations for which the participant has given explicit consent for data transfer. Representatives of the WILL trial team, sponsor, and other oversight organisations may be required to have access to participant's notes for quality assurance purposes but participants should be reassured that their confidentiality will be respected at all times.

17. FINANCIAL AND OTHER COMPETING INTERESTS

The Chief Investigator declares that there are no ownership interests that may be related to products, services, or interventions considered for use in the trial or that may be significantly affected by the trial. There are no commercial ties that require disclosure, which include any pharmaceutical, behaviour modification and/or technology company. Furthermore, there are no non-commercial potential conflicts (e.g. professional collaborations that may impact on academic promotion). It should be noted that at the time of writing the *current version of the* protocol*,* not all *staff or* sites have been identified. When this is the case, any financial and other competing interest will be documented.

18. INSURANCE AND INDEMNITY

King's College London has in place Clinical Trials indemnity coverage for this trial which provides cover to the University for harm which comes about through the University's, or its staff's, negligence in relation to the design or management of the trial and may alternatively, and at the University's discretion provide cover for non-negligent harm to participants. With respect to the conduct of the trial at site and other clinical care of the patient, responsibility for the care of the patients remains with the NHS organisation responsible for the clinical site and is therefore indemnified through the NHS Litigation Authority. King's College London is independent of any pharmaceutical company, and as such it is not covered by the Association of the British Pharmaceutical Industry (ABPI) guidelines for participant compensation.

19. AMENDMENTS

All amendments will be tracked in the 'Protocol Amendments' section of the protocol. The decision to amend the protocol and associated trial documentation will be initiated by the TMG. The Sponsor will be responsible for deciding whether an amendment is substantial or non-substantial. Substantive changes will be submitted to REC and HRA for approval. Once this has been received, R&D departments will be notified of the amendment, and requested to provide their approval. If no response is received within 35 days, an assumption will be made that the site has no objection to the amendment and it will be implemented at the site.

20. PUBLICATION POLICY

Results of this trial will be submitted for publication in peer reviewed journals. The manuscript will be

prepared by the TMG; all contributors to the trial will be listed, with their contribution identified and specifically, all collaborating site teams will be listed in an Appendix as the 'WILL Study Group'. Abstracts will be submitted to international medical congresses. Trial participants will be able to access the final results of the trial via the trial website. All publications/presentations that use data from this trial to undertake original analyses will be submitted to the Funders for review before release; these must be submitted in a timely fashion and in advance of being submitted for publication, to allow time for review and resolution of any outstanding issues.

On all publications, the authors must acknowledge that the trial was: (i) performed with the support of The UofB BCTU, King's College London, and Guy's and St. Thomas' Foundation NHS Trust; and (ii) funded by the NIHR. To safeguard the scientific integrity of the trial, data from this trial will not be presented in public before the main results are published without the prior consent of the funder and the TMG.

21. REFERENCE LIST

(1) Hutcheon JA, Lisonkova S, Joseph KS. Epidemiology of pre-eclampsia and the other hypertensive disorders of pregnancy. Best Pract Res Clin Obstet Gynaecol 2011 Aug;25(4):391-403.

(2) National Statistics. Vital Statistics: Population and Health Reference Tables. www ons gov uk 2015.

(3) Cluver C, Novikova N, Koopmans CM, West HM. Planned early delivery versus expectant management for hypertensive disorders from 34 weeks gestation to term. Cochrane Database Syst Rev 2017 Jan 15;1:CD009273.

(4) Hamed HO, Alsheeha MA, Abu-Elhasan AM, Abd Elmoniem AE, Kamal MM. Pregnancy outcomes of expectant management of stable mild to moderate chronic hypertension as compared with planned delivery. Int J Gynaecol Obstet 2014 Oct;127(1):15-20.

(5) Koopmans CM, Bijlenga D, Groen H, Vijgen SM, Aarnoudse JG, Bekedam DJ, et al. Induction of labour versus expectant monitoring for gestational hypertension or mild pre-eclampsia after 36 weeks' gestation (HYPITAT): a multicentre, open-label randomised controlled trial. Lancet 2009 Sep 19;374(9694):979-88.

(6) Hypertension in pregnancy: diagnosis and management NICE guideline NG133. www nice org/ng 133 2019.

(7) Bramham K, Parnell B, Nelson-Piercy C, Seed PT, Poston L, Chappell LC. Chronic hypertension and pregnancy outcomes: systematic review and meta-analysis. BMJ 2014 Apr 15;348:g2301.

(8) Magee LA, von Dadelszen P., Stones W, Mathai M. The FIGO Textbook of Pregnancy Hypertension. The Global Library of Women's Medicine; 2016.

(9) Magee LA, von Dadelszen P., Rey E, Ross S, Asztalos E, Murphy KE, et al. Less-tight versus tight control of hypertension in pregnancy. N Engl J Med 2015 Jan 29;372(5):407-17.

(10) von Dadelszen P, Payne B, Li J, Ansermino JM, Broughton PF, Cote AM, et al. Prediction of adverse maternal outcomes in pre-eclampsia: development and validation of the fullPIERS model. Lancet 2011 Jan 15;377(9761):219-27.

(11) Duffy JM, van 't HJ, Gale C, Brown M, Grobman W, Fitzpatrick R, et al. A protocol for developing, disseminating, and implementing a core outcome set for pre-eclampsia. Pregnancy Hypertens 2016 Oct;6(4):274-8.

(12) Broekhuijsen K, van Baaren GJ, van Pampus MG, Ganzevoort W, Sikkema JM, Woiski MD, et al. Immediate delivery versus expectant monitoring for hypertensive disorders of pregnancy between 34 and 37 weeks of gestation (HYPITAT-II): an open-label, randomised controlled trial. Lancet 2015 Jun 20;385(9986):2492-501.

(13) Nijjar SK, D'Amico MI, Wimalaweera NA, Cooper N, Zamora J, Khan KS. Participation in clinical trials improves outcomes in women's health: a systematic review and meta-analysis. BJOG 2017 May;124(6):863-71.

(14) RCOG Each Baby Counts iniative (https://www.rcog.org.uk/en/news/rcog-statement-mbrrace-ukreport-on-stillbirths-and-neonatal-deaths/)). 2015.

(15) Manktelow BN, Smith LK, Prunet C, Smith PW, Boby T, Hyman-Taylor P, et al. MBRRACE-UK Perinatal Mortality Surveillance Report, UK Perinatal Deaths for Births from January to December 2015. 2017. Leicester, The Infant Mortality and Morbidity Studies, Department of Health Sciences, University of Leicester.

(16) British Association of Perinatal Medicine, Categories of Care (http://www.bapm.org/publications/documents/guidelines/CatsofcarereportAug11.pdf). 2011.

(17) Ahmed RJ, Gafni A, Hutton EK, Hu ZJ, Pullenayegum E, von DP, et al. The Cost Implications of Less Tight Versus Tight Control of Hypertension in Pregnancy (CHIPS Trial). Hypertension 2016 Oct;68(4):1049-55.

(18) Cruz MO, Gao W, Hibbard JU. What is the optimal time for delivery in women with gestational hypertension? Am J Obstet Gynecol 2012 Sep;207(3):214-6.

(19) Hutcheon JA, Lisonkova S, Magee LA, von DP, Woo HL, Liu S, et al. Optimal timing of delivery in pregnancies with pre-existing hypertension. BJOG 2011 Jan;118(1):49-54.

(20) Vijgen SM, Koopmans CM, Opmeer BC, Groen H, Bijlenga D, Aarnoudse JG, et al. An economic analysis of induction of labour and expectant monitoring in women with gestational hypertension or pre-eclampsia at term (HYPITAT trial). BJOG 2010 Dec;117(13):1577-85.

(21) Magee LA, von DP, Bohun CM, Rey E, El-Zibdeh M, Stalker S, et al. Serious perinatal complications of non-proteinuric hypertension: an international, multicentre, retrospective cohort study. J Obstet Gynaecol Can 2003 May;25(5):372-82.

(22) Magee LA, von Dadelszen P, Chan S, Gafni A, Gruslin A, Helewa M, et al. The Control of Hypertension In Pregnancy Study Pilot Trial. BJOG 2007 Jun;114(6):770, e13-770, e20.

(23) Barton JR, O'Brien JM, Bergauer NK, Jacques DL, Sibai BM. Mild gestational hypertension remote from term: progression and outcome. Am J Obstet Gynecol 2001 Apr;184(5):979-83.

(24) Buchbinder A, Sibai BM, Caritis S, Macpherson C, Hauth J, Lindheimer MD, et al. Adverse perinatal outcomes are significantly higher in severe gestational hypertension than in mild preeclampsia. Am J Obstet Gynecol 2002 Jan;186(1):66-71.

(25) Chappell LC, Enye S, Seed P, Briley AL, Poston L, Shennan AH. Adverse perinatal outcomes and risk factors for preeclampsia in women with chronic hypertension: a prospective study. Hypertension 2008 Apr;51(4):1002-9.

(26) Magee LA, Abalos E, von DP, Sibai B, Walkinshaw SA. Control of hypertension in pregnancy. Curr Hypertens Rep 2009 Dec;11(6):429-36.

(27) Ray JG, Burrows RF, Burrows EA, Vermeulen MJ. MOS HIP: McMaster outcome study of hypertension in pregnancy. Early Hum Dev 2001 Sep;64(2):129-43.

(28) Terrone DA, Rinehart BK, May WL, Martin RW, Martin JN, Jr. The myth of transient hypertension: descriptor or disease process? Am J Perinatol 2001;18(2):73-7.

(29) Kuklina EV, Ayala C, Callaghan WM. Hypertensive disorders and severe obstetric morbidity in the United States. Obstet Gynecol 2009 Jun;113(6):1299-306.

(30) Caughey AB, Sundaram V, Kaimal AJ, Cheng YW, Gienger A, Little SE, et al. Maternal and neonatal outcomes of elective induction of labor. Evid Rep Technol Assess (Full Rep) 2009 Mar;(176):1-257.

(31) Joseph KS, Rouleau J, Kramer MS, Young DC, Liston RM, Baskett TF. Investigation of an increase in postpartum haemorrhage in Canada. BJOG 2007 Jun;114(6):751-9.

(32) Mehrabadi A, Hutcheon JA, Lee L, Liston RM, Joseph KS. Trends in postpartum hemorrhage from 2000 to 2009: a population-based study. BMC Pregnancy Childbirth 2012 Oct 11;12:108.

(33) Rossen J, Okland I, Nilsen OB, Eggebo TM. Is there an increase of postpartum hemorrhage, and is severe hemorrhage associated with more frequent use of obstetric interventions? Acta Obstet Gynecol Scand 2010 Oct;89(10):1248-55.

(34) Zetterstrom K, Lindeberg SN, Haglund B, Hanson U. Maternal complications in women with chronic hypertension: a population-based cohort study. Acta Obstet Gynecol Scand 2005 May;84(5):419-24.

(35) Ghosh B, Mittal S, Kumar S, Dadhwal V. Prediction of perinatal asphyxia with nucleated red blood cells in cord blood of newborns. Int J Gynaecol Obstet 2003 Jun;81(3):267-71.

(36) Canadian Institutes for Health Information. The cost of hospital stays: why costs vary. 2008. CIHI.

(37) Chiossi G, Lai Y, Landon MB, Spong CY, Rouse DJ, Varner MW, et al. Timing of delivery and adverse outcomes in term singleton repeat cesarean deliveries. Obstet Gynecol 2013 Mar;121(3):561-9.

(38) Oshiro BT, Henry E, Wilson J, Branch DW, Varner MW. Decreasing elective deliveries before 39 weeks of gestation in an integrated health care system. Obstet Gynecol 2009 Apr;113(4):804-11.

(39) Tita AT, Landon MB, Spong CY, Lai Y, Leveno KJ, Varner MW, et al. Timing of elective repeat cesarean delivery at term and neonatal outcomes. N Engl J Med 2009 Jan 8;360(2):111-20.

(40) Wilmink FA, Hukkelhoven CW, Lunshof S, Mol BW, van der Post JA, Papatsonis DN. Neonatal outcome following elective cesarean section beyond 37 weeks of gestation: a 7-year retrospective analysis of a national registry. Am J Obstet Gynecol 2010 Mar;202(3):250-8.

(41) Yee W, Amin H, Wood S. Elective cesarean delivery, neonatal intensive care unit admission, and neonatal respiratory distress. Obstet Gynecol 2008 Apr;111(4):823-8.

(42) Zhang X, Kramer MS. Variations in mortality and morbidity by gestational age among infants born at term. J Pediatr 2009 Mar;154(3):358-62, 362.

(43) Mackay DF, Smith GC, Dobbie R, Pell JP. Gestational age at delivery and special educational need: retrospective cohort study of 407,503 schoolchildren. PLoS Med 2010 Jun 8;7(6):e1000289.

(44) Owens MY, Thigpen B, Parrish MR, Keiser SD, Sawardecker S, Wallace K, et al. Management of preeclampsia when diagnosed between 34-37 weeks gestation: deliver now or deliberate until 37 weeks? J Miss State Med Assoc 2014 Jul;55(7):208-11.

(45) Magee LA, Yong PJ, Espinosa V, Cote AM, Chen I, von DP. Expectant management of severe preeclampsia remote from term: a structured systematic review. Hypertens Pregnancy 2009;28(3):312-47.

(46) Gulmezoglu AM, Crowther CA, Middleton P, Heatley E. Induction of labour for improving birth outcomes for women at or beyond term. Cochrane Database Syst Rev 2012 Jun 13;(6):CD004945.

(47) Mishanina E, Rogozinska E, Thatthi T, Uddin-Khan R, Khan KS, Meads C. Use of labour induction and risk of cesarean delivery: a systematic review and meta-analysis. CMAJ 2014 Jun 10;186(9):665-73.

(48) Hodnett ED, Hannah ME, Weston JA, Ohlsson A, Myhr TL, Wang EE, et al. Women's evaluations of induction of labor versus expectant management for prelabor rupture of the membranes at term. TermPROM Study Group. Birth 1997 Dec;24(4):214-20.

(49) Shetty A, Burt R, Rice P, Templeton A. Women's perceptions, expectations and satisfaction with induced labour--a questionnaire-based study. Eur J Obstet Gynecol Reprod Biol 2005 Nov 1;123(1):56-61.

(50) Pauli JM, Lauring JR, Stetter CM, Repke JT, Botti JJ, Ural SH, et al. Management of gestational hypertension - the impact of HYPITAT. J Perinat Med 2013 Jul;41(4):415-20.

(51) van der Tuuk K, Koopmans CM, Groen H, Mol BW, van Pampus MG. Impact of the HYPITAT trial on doctors' behaviour and prevalence of eclampsia in the Netherlands. BJOG 2011 Dec;118(13):1658-60.

(52) Public Health Agency of Canada. Canadian Perinatal Health Report. 2008.

(53) Harper LM, Biggio JR, Anderson S, Tita AT. Gestational Age of Delivery in Pregnancies Complicated by Chronic Hypertension. Obstet Gynecol 2016 Jun;127(6):1101-9.

(54) Girouard J, Giguere Y, Moutquin JM, Forest JC. Previous hypertensive disease of pregnancy is associated with alterations of markers of insulin resistance. Hypertension 2007 May;49(5):1056-62.

(55) Newstead J, von DP, Magee LA. Preeclampsia and future cardiovascular risk. Expert Rev Cardiovasc Ther 2007 Mar;5(2):283-94.

(56) Ray JG, Schull MJ, Kingdom JC, Vermeulen MJ. Heart failure and dysrhythmias after maternal placental syndromes: HAD MPS Study. Heart 2012 Aug;98(15):1136-41.

(57) Walker KF, Bugg GJ, Macpherson M, McCormick C, Grace N, Wildsmith C, et al. Randomized Trial of Labor Induction in Women 35 Years of Age or Older. N Engl J Med 2016 Mar 3;374(9):813-22 (orphaned from this version).

(58) Kahan BC, Morris TP. Reporting and analysis of trials using stratified randomisation in leading medical journals: review and reanalysis. BMJ 2012 Sep 14;345:e5840.

(59) Zou G. A modified poisson regression approach to prospective studies with binary data. Am J Epidemiol 2004 Apr 1;159(7):702-6.

(60) Pocock SJ, Ariti CA, Collier TJ, Wang D. The win ratio: a new approach to the analysis of composite endpoints in clinical trials based on clinical priorities. Eur Heart J 2012;33:176-82 (orphaned from this version).

(61) Wood S, Cooper S, Ross S. Does induction of labour increase the risk of caesarean section? A

systematic review and meta-analysis of trials in women with intact membranes. BJOG 2014 May;121(6):674-85 (orphaned from this version).

(62) Magee LA (for the CHIPS Study Group), von Dadelszen P, Singer J, Lee T, Rey E, Ross S, Asztalos E, Murphy KE, Menzies J, Sanchez J, Gafni A, Gruslin A, Helewa M, Hutton E, Lee SK, Logan AG, Ganzevoort W, Welch R, Thornton JG, Moutquin J-M. The Control of Hypertension In Pregnancy Study (CHIPS) randomised controlled trial – is severe hypertension just an elevated blood pressure? Hypertension 2016;68(5):1153-9 (orphaned from this version).

(63) Villar J, Cheikh L, Victora CG, Ohuma EO, Bertino E, Altman DG, Lambert A, Papageorghiou AT, Carvalho M, Jaffer YA, Gravett MG, Purwar M, Frederick IO, Noble AJ, Pang R, Barros FC, Chumlea C, Bhutta ZA, Kennedy SH, for the International Fetal and Newborn Growth Consortium for the 21st Century (INTERGROWTH-21st). International standards for newborn weight, olength, and head circumference by gestational age and sex: the Newborn Cross-Sectional Study of the INTERGROWTH-21ST Project. Lancet 2014;384:857-68.

(64) Walker KF, Wilson P, Bugg, GJ, Dencker A, Thornton JG. Childbirth experience questionnaire: validating its use in the United Kingdom. BMC Pregnancy and Childbirth 2015;15:86.

(65) https://www.npeu.ox.ac.uk/mbrrace-uk/reports/confidential-enquiry-into-maternal-deaths

(66) Grobman WA, Rice MM, Reddy UM, Tita ATN, Silver RM, Mallett G, Hill K, Thom EA, El-Sayed YY, Perez-Delboy AP, Rouse EJ, Saade GR, Boggess KA, Chauhan AP, Iams JD, Chien EK, Casey BM, Gibbs RS, Srinivas SK, Swamy GK, Simhan HN, Macones GA, for the Eunice Kennedy Shriver National Institute of Child Health and Human Development Maternal-Fetal Medicine Units Network. Labor induction versus expectant management in low-risk nulliparous women. NEJM 2018;379:513-23.

(67) Brown MA, Magee LA, Kenny LC, Karumanchi SA, McCarthy FP, Saito S, Hall DR, Warren CE, Adoyi G, Ishaku S, on behalf of the International Society for the Study of Hypertension in Pregnancy (ISSHP). Hypertensive Disorders of Pregnancy - ISSHP Classification, Diagnosis, and Management Recommendations for International Practice. Hypertension 2018;72:24-43.

22. APPENDIX

Appendix 1: Background for WILL Trial

In the UK, up to 55,000 pregnancies/year are complicated by chronic hypertension (diagnosed before pregnancy or at <20 weeks' gestation) or gestational hypertension (diagnosed at ≥20 weeks), and half of these women will reach term gestational age. Early term delivery (at 37-38 weeks) may reduce maternal complications and stillbirth, but it may also increase neonatal morbidity and costs, related primarily to the cost of maternal and fetal surveillance during expectant care and possibly increased Caesarean deliveries that are greater than the costs of labour induction(18-20). There are no high-quality data on which to base clinical decision-making.

Rates of chronic and gestational hypertension (7% of deliveries) are rising with an ageing and an increasingly obese obstetric population(1). Every year in the UK, these pregnancies are responsible for approximately 12,000 poor maternal outcomes, 25,000 Caesareans and 240 stillbirths(1). Given the potential for life-long morbidity in women with serious complications and/or Caesareans, delivery at early term may improve substantive outcomes and NHS resource use. However, any potentially negative impact of the intervention on neonatal morbidity must be explored and justified.

Variation in guidelines and practice supports equipoise. NICE (2010) advises that timing of birth for women with chronic or gestational hypertension, "be agreed upon with the woman" (expert opinion)(6). Current care at term involves maternal and fetal surveillance, and intervention for maternal morbidity or fetal compromise, either of which may be rapid or unexpected. Practice varies widely; in a survey of Control of Hypertension In Pregnancy Study Trial (including the UK), 70 respondents highlighted variable practice, with delivery currently offered at 37 weeks' (16%), 38 weeks' (33%), 39 weeks' (20%), 40 weeks' (20%), and 41 weeks' gestation (12%).

To guide care for these high-risk women, the WILL trial (When to Induce Labour to Limit risk in pregnancy hypertension) compares a policy of delivery at 38 weeks, against usual care at term (or as clinical need dictates), with regards to maternal complications (and Caesareans), whilst ensuring that neonatal health is not compromised(2,3).

- **Risks of usual care at term**

With increasing gestational age at term, women with chronic or gestational hypertension are at risk of 'placental ageing' and dysfunction, which may lead to adverse maternal or baby outcomes, or an increased risk of a Caesarean.

For the mother, placental abruption (2% of women) or serious end-organ complications (4%, eg, pulmonary oedema) may occur(9;21;22), even in the absence of pre-eclampsia (13-17%)(23-28). These risks are up to 10-fold higher compared with normotensive pregnancy(18;29).

Contrary to widely-held beliefs, expectant care at term probably *increases* (not decreases) emergency Caesareans (see Section 1.2). The pervasive belief that induction increases Caesareans(30-33) arises from methodological bias: outcomes following induction have most often been compared with those of spontaneous labour. Yet, clinicians do not decide between induction and spontaneous labour, but between induction and expectant care that may lead to either spontaneous labour onset or medically-indicated delivery. Caesarean delivery itself increases maternal risk in index (e.g., thromboembolism) and future pregnancies (e.g., adherent placenta).

For the baby, the risk of expectant care is stillbirth related to poor fetal growth or perinatal asphyxia - for example, acute due to placental abruption(34) or chronic due to placental ageing and

insufficiency(35). At 36 weeks, the stillbirth risk in chronic or gestationally hypertensive pregnancies is 1/1000 - the risk for women with uncomplicated pregnancy at 41 weeks when routine induction is recommended(1).

- **Risks of early term delivery**

There is no compelling evidence that maternal risk is increased by induction, the most common method of early delivery. RCTs show that induction does not increase Caesarean delivery and risks in the index and future pregnancy(ies), related to repeat Caesarean, placenta accreta/percreta, hysterectomy, or thromboembolism. However, induction incurs costs of one-to-one midwifery and extended hospital stay(36). Also, a policy of induction at 38 weeks for women with chronic or gestational hypertension who reach term would represent a policy shift and unnecessary medical intervention if such a policy did not confer benefits or cost-savings.

The risks of early term delivery relate to the newborn. Delivery at 38 weeks (vs. 40 weeks) may increase neonatal mortality and morbidity (at least 2-fold) requiring special neonatal care(37-42), and possibly, special educational needs above the baseline of ~5% (OR 1.19 [1.04, 1.14] at 38 vs. 40 weeks)(43).

- **The literature**

Among women with chronic(1) or gestational hypertension(18), observational data suggest that delivery between 38^{+0} and 39^{+6} weeks may optimise outcomes for the baby, by minimising intra-uterine death that rises in incidence with advancing gestational age at term, and neonatal morbidity that falls with advancing gestational age. However, observational studies are confounded by indication for delivery, so it is not possible to estimate with any certainty the impact of different gestations of delivery on perinatal outcomes. Also, the impact of planned delivery at term (i.e., at 37^{+0} to 41^{+6} weeks) on maternal morbidity or Caesarean could not be assessed.

There are no definitive trials that have established how best to manage women with chronic or gestational hypertension who reach 37 weeks and require delivery at term; yet, these women represent 1/3 of all women with pregnancy hypertension in the UK. The five trials (1,819 women) in the 2017 Cochrane review(3) are summarised (Table 1, over) and discussed below.

For well women with chronic or gestational hypertension who reach term gestational age (37^{+0} weeks), the specific issue we seek to address is *not* delivery before 37 weeks, when only more severe maternal disease might justify prematurity-related neonatal risks(4;12). Rather, we ask whether delivery later, at an early term gestational age of 38^{+0-3} weeks, will be better for the mother, without increasing morbidity for the baby, something that is unlikely at this gestation. When one considers the type of pregnancy hypertension and the timing of enrolment and planned delivery of the five previous trials detailed below, it is clear that the vast majority of women in prior trials either had proteinuric pre-eclampsia, or were randomised at earlier or later gestational ages than we plan (or both).

Table 1: Trials of planned delivery vs. expectant usual care in hypertensive pregnancy at late preterm/term gestational ages(3)

	**WILL trial**	**HYPITAT II 2015(12)**	**Hamed 2014(4)**	**Owens 2014(44)**	**HYPITAT I 2009(5)**	**Majeed 2014(3)***
N enrolled	1,080 women	754 women	76 women	183 women	756 women	100 women
N WILL eligible	1,080	0	50†	0	188‡	?*
**Type of HDP**						
Chronic hypertension	Yes	Yes (N=98)	Yes (N=50)	No	No	No
Gestational hypertension	Yes	Yes (N=182)	No	No	Yes (N=493†overall)	Yes (N=?)*
Pre-eclampsia	No	Yes	No	Yes	Yes	Yes
Gestational age at enrolment	Early term (37^{0-6} wks)	Preterm (34^0-37^0 wks	Preterm (24^0-36^6 wks)	Preterm (34^0-37^0 wks)	Term (36^0-41^0 wks)	Term (36^0-40^0 wks)
**Delivery timing**	Term	Preterm	Term	Preterm	Term	Term
**Primary outcome¥**	Maternal composite¥	Maternal composite¥	Pre-eclampsia	Maternal composite¥	Maternal composite¥	Maternal composite¥

HDP = Hypertensive Disorder of Pregnancy

* *Abstract only despite further inquiries from Cochrane.*

† *50/76 women were still pregnant at 37^{+0} weeks.*

‡ *188/756 women were recruited at 37^{+0-6} weeks. It is not known what proportion of these women had gestational hypertension (which was 65% overall), as the Cochrane review presented enrolment by gestational age or type of hypertension, but not both(3).*

¥ *Maternal composite outcomes varied. HYPITAT I & II and Owens 2014 included maternal mortality. HYPITAT I, HYPITAT II, and Majeed 2014 each included maternal morbidity of eclampsia, HELLP syndrome, pulmonary oedema, and placental abruption; Owens 2014 included all severe features of pre-eclampsia according to the American College of Obstetricians and Gynaecologists, and Hamed 2014 included only abruption. Other morbidities were included by different trials: thromboembolic disease (HYPITAT I & II), severe hypertension (HYPITAT II, Majeed 2014), superimposed pre-eclampsia (Hamed 2014), severe features of pre-eclampsia (Owens 2014), severe proteinuria (HYPITAT II, Majeed 2014), major PPH (HYPITAT II, Majeed 2014), DIC and retinal haemorrhage (Majeed 2014).*

1. ***Owens 2014*** enrolled only women with pre-eclampsia(44), a distinct hypertensive disorder of pregnancy characterised by a shorter natural history and higher risk of complications(8;45). Chronic and gestational hypertension are sufficiently distinct from pre-eclampsia, and sufficiently common (7% of all pregnancies) to justify a trial of their own.

2. ***HYPITAT II*** enrolled *and* delivered women (of whom 280 [40%] had chronic or gestational hypertension) by 37^{+0} weeks, leading to neonatal respiratory morbidity (RR 3·3, 95% CI 1·4, 8·2)(12). This indicates that delivery before 37^{+0} weeks may be too early from the newborn perspective.

3. The results of ***Hamed 2014*** raise concern that even 37^{+0} weeks may be too early to plan delivery, at least for women with chronic hypertension. In Hamed 2014, 50/76 women with chronic hypertension who were enrolled between 24 and 36 weeks were randomised to planned delivery at either 37^{+0} or 41^{+6} weeks, and reached 37^{+0} weeks(4). Planned earlier term delivery was associated with lower birthweight (by mean 400g) and more frequent admission to neonatal intensive care (5.3% vs. 2.6%; both p<0.01).

4. In ***HYPITAT I***, a subgroup of 188 (24.9%) women were enrolled at 37^{+0-6} weeks, so 'immediate delivery' would be similar to planned early term delivery at 38^{+0-3} weeks in WILL(5). In this subgroup, the composite of maternal mortality and morbidity [OR 0.75, 95% CI 0.52, 1.08] suggested benefits of earlier delivery, as seen for all women in the trial [OR 0.71, 95% CI 0.59, 0.86]). Neonatal mortality and morbidity did not increase with earlier delivery (OR 0.48, 95% CI 0.17, 1.35), as seen overall in the trial (OR 0.75, 95% CI 0.45, 1.26). Overall in HYPITAT I, earlier delivery did not increase Caesareans (RR 0.75, 95% CI 0.55, 1.04) or adversely affect health-related quality of life, and costs were *reduced* (by €831)(20). Of note, both the subgroup and overall results include women with pre-eclampsia, as subgroup analyses are available according to either hypertensive disorder or gestational age at enrolment, but not both(3).

5. Despite the similarities in design of ***Majeed 2014*** to HYPITAT I, it is not known whether there are similarities in results as discussed above, as the trial has been published only in abstract form and details have not been available by direct correspondence with the authors in Cochrane.

Appendix 2: WILL Participant Recruitment Flow Chart

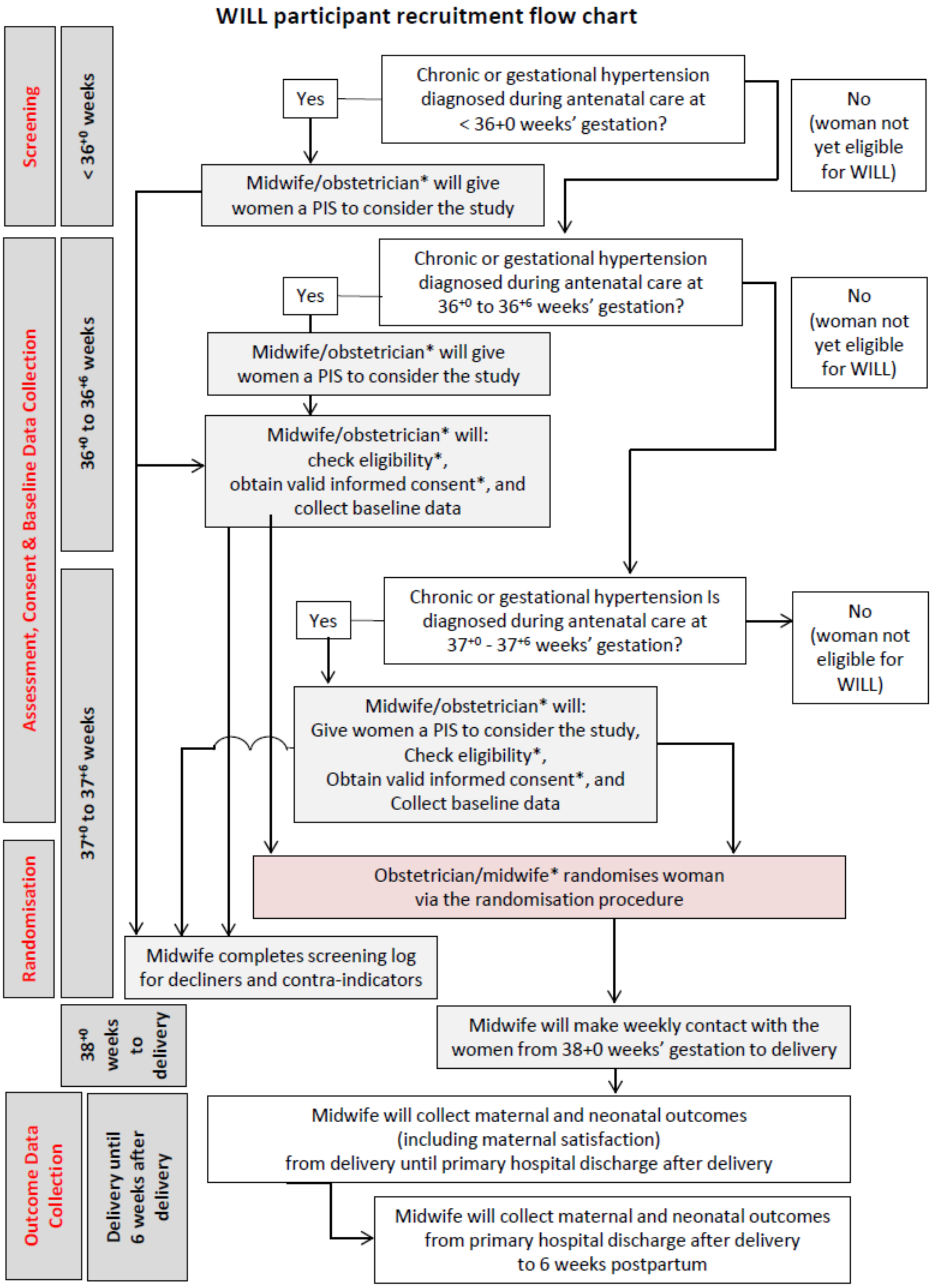

Patient Recruitment Flow Chart v0.1, 10 June 2018

In accordance with local policy. Abbreviations: PIS (Patient Informaton Sheet)
