# Supplementary material for: Determining optimal timing of birth for women with chronic or gestational hypertension at term: The WILL (When to Induce Labour to Limit risk in pregnancy hypertension) randomised trial
Source: PLoS Med. 2024 Nov 26;21(11):e1004481. doi: 10.1371/journal.pmed.1004481 (PMC11593758; doi:10.1371/journal.pmed.1004481)
Supplement: S1 Appendix — (PDF) [file pmed.1004481.s003.pdf]

When to Induce Labour to Limit risk in pregnancy hypertension – a multicentre, randomised controlled trial

The WILL Trial

Trial Registration: ISRCTN 77258279

Statistical Analysis Plan

SAP Version Number	Protocol Version Number
2.0	4.0

Name of Author:	Eleni Gkini	Role:	Trial Statistician	Affiliation:	BCTU<br>University of Birmingham
Signature of Author:	Eleni Gkini	Date:	28 JUN 2023		
Name of Blind Reviewer:	Jon Bishop	Role:	Senior Statistician	Affiliation:	BCTU<br>University of Birmingham
Signature of Reviewer:	J R B Bishop	Date:	28 JUN 2023		
Name of Chief Investigator:	Prof Laura A. Magee	Role:	Chief Investigator	Affiliation:	King's College London
Signature of Chief Investigator:		Date:	28 JUN 2023		
**This Statistical Analysis Plan has been approved by:**					
Name of Approver:	Catherine Moakes	Role:	Senior Statistician	Affiliation:	BCTU<br>University of Birmingham
Signature of Approver:	C A MOAKES	Date:	28 JUN 2023		

Statistical Analysis Plan (SAP) Amendments

SAP version number	SAP section number	Description of and reason for change	Timing of change with respect to interim analysis/ final analysis/ database lock	Blind Reviewer	
2.0	2, 3, 4.2, 4.6, 4.7, 4.8, 4.13, 9.10	Amendment of the wording the 'expectant care at term until at least 40 weeks' to 'usual care at term'.	After version 4.0 of the protocol was released. Prior to database hard lock.	Name:	Jon Bishop
				Signature:	J R B Bishop
				Date:	28 JUN 2023
	5.3/5.4/7.2/9.10	Redefinition of adherence (and presentation of adherence data), including addition of a CACE analysis.		Name:	Jon Bishop
				Signature:	J R B Bishop
				Date:	28 JUN 2023
	9.1/9.5	Primary analysis estimation of a RR and RD (added to this version of the SAP) changed to a standardisation approach. Removal of references to a log-binomial model (or Poisson model for estimating a RR when convergence is an issue).		Name:	Jon Bishop
				Signature:	J R B Bishop
				Date:	28 JUN 2023
	9.2/9.10	Clarification of sensitivity analyses corresponding to outliers and distributional assumptions.		Name:	Jon Bishop
				Signature:	J R B Bishop
				Date:	28 JUN 2023
	9.6	Details provided for analysis of secondary count outcomes.		Name:	Jon Bishop
				Signature:	J R B Bishop
				Date:	28 JUN 2023

SAP version number	SAP section number	Description of and reason for change	Timing of change with respect to interim analysis/ final analysis/ database lock	Blind Reviewer	
	9.9	Ratio of ratios added to the subgroup analyses.		Name:	Jon Bishop
				Signature:	J R B Bishop
				Date:	28 JUN 2023
	9.10	Addition of new sensitivity analyses for both co-primary outcomes: a restricted analysis for achieving a week separation between arms in GA at births, a sub-group analysis of exploring the heterogeneity of treatment effect due to the change in the usual care arm, and tipping point analyses to assess missing data.		Name:	Jon Bishop
				Signature:	J R B Bishop
				Date:	28 JUN 2023
	9.10	Sensitivity analyses expanded to include the key secondary outcome (adjustment of baseline covariates, CACE, restricted analysis, heterogeneity of treatment effect due to protocol change).		Name:	Jon Bishop
				Signature:	J R B Bishop
				Date:	28 JUN 2023
	Appendix B	Amendment of the trial schema.		Name:	Jon Bishop
				Signature:	J R B Bishop
				Date:	28 JUN 2023
	Appendix E	Data manipulations moved to appendices.		Name:	Jon Bishop
				Signature:	J R B Bishop
				Date:	28 JUN 2023

Abbreviations & Definitions	
**Abbreviation/Acronym**	**Meaning**
ALT	Alanine Aminotransferase
AST	Aspartate Aminotransferase
BAPM	British Association of Perinatal Medicine
BCTU	Birmingham Clinical Trials Unit
BMI	Body Mass Index
BP	Blood Pressure
CACE	Complier Average Causal Effect
CRFs	Case Report Forms
CIG	Co-Investigator Group
CIs	Confidence Intervals
CONSORT	Consolidated Standards of Reporting Trials
CRF	Case Report Form
dBP	Diastolic Blood Pressure
DIC	Disseminated Intravascular Coagulation
DMC	Data Monitoring Committee
ECG	Electrocardiogram
EDD	Estimated Date of Delivery
GA	Gestational Age
GCS	Glasgow Coma Scale
GDM	Gestational Diabetes Mellitus
HEAP	Health Economics Analysis Plan
HIE	Hypoxic Ischemic Encephalopathy
HIV	Human Immunodeficiency Virus
INR	International Normalised Ratio
IQR	Interquartile Range
IRR	Incidence Rate Ratio
ISRCTN	International Standard Randomised Controlled Trial Number
ISSHP	International Society for the Study of Hypertension in Pregnancy
ITT	Intention to Treat
ITU	Intensive Treatment Unit
MAP	Mean Arterial Pressure
MBRRACE-UK	Mothers and Babies: Reducing Risk through Audits and Confidential Enquiries across the UK
NHS	National Health Service
NICE	National Institute of Health and Care Excellence
PPH	Postpartum Hemorrhage
qSOFA	Quick Sequential Organ Failure
RD	Risk difference
RR	Risk ratio
RSUSAE	Related Unexpected Serious Adverse Event
SAEs	Serious Adverse Events
SAP	Statistical Analysis Plan
sBP	Systolic Blood Pressure
SpO2	Peripheral capillary oxygen saturation
TIA	Transient Ischaemic Attack
TMG	Trial Management Group
TSC	Trial Steering Committee

UK	United Kingdom
**Term**	**Definition**
International Standard Randomised Controlled Trial Number	A clinical trial registry
Protocol	Document that details the rationale, objectives, design, methodology and statistical considerations of the study
Randomisation	The process of assigning trial participants to intervention or control groups using an element of chance to determine the assignments in order to reduce bias.
Statistical Analysis Plan	Pre-specified statistical methodology documented for the trial, either in the protocol or in a separate document.
WILL Final Report Template	This is a separate document to the SAP that contains the full set of dummy tables for the final report.

TABLE OF CONTENTS

1. **Introduction** ... 9
2. **Background and rationale** ... 9
3. **Trial objectives** ... 9
4. **Trial methods** ... 10
   - 4.1. Trial design ... 10
   - 4.2. Trial interventions ... 10
   - 4.3. Primary outcomes ... 10
   - 4.4. Secondary outcomes ... 11
   - 4.5. Timing of outcome assessments ... 13
   - 4.6. Randomisation ... 13
   - 4.7. Sample size ... 14
   - 4.8. Framework ... 14
   - 4.9. Interim analyses and stopping guidance ... 14
   - 4.10. Internal Pilot Progression Rules ... 15
   - 4.11. Timing of final analysis ... 15
   - 4.12. Timing of other analyses ... 15
   - 4.13. Trial comparisons ... 15
5. **Statistical Principles** ... 15
   - 5.1. Confidence intervals and p-values ... 15
   - 5.2. Adjustments for multiplicity ... 15
   - 5.3. Analysis populations ... 15
   - 5.4. Definition of adherence ... 15
   - 5.5. Handing protocol deviations ... 16
   - 5.6. Unblinding ... 16
6. **Trial population** ... 16
   - 6.1. Recruitment ... 16
   - 6.2. Baseline characteristics ... 16
7. **Intervention** ... 17
   - 7.1. Description of the intervention ... 17
   - 7.2. Adherence to allocated intervention ... 17
8. **Protocol deviations** ... 17
9. **Analysis methods** ... 17
   - 9.1. Covariate adjustment ... 17
   - 9.2. Distributional assumptions and outlying responses ... 17
   - 9.3. Handling missing data ... 17

9.4. Data manipulations ... 17
   - 9.5. Analysis methods – primary outcomes ... 17
   - 9.6. Analysis methods – secondary outcomes ... 18
   - 9.7. Analysis methods – exploratory outcomes and analyses ... 18
   - 9.8. Safety data ... 18

9.9. Planned subgroup analyses ... 18
9.10. Sensitivity & Supportive analyses ... 19
10. Analysis of sub-randomisations ... 20
11. Health economic analysis ... 20
12. Statistical software ... 20
13. References ... 20
Appendix A: Deviations from SAP ... 22
Appendix B: Trial schema ... 23
Appendix C: Schedule of assessments ... 24
Appendix D: 'Stop-go' criteria ... 25
Appendix E: Data manipulations ... 26

1. Introduction

This document is the Statistical Analysis Plan (SAP) for the WILL study, and should be read in conjunction with the current trial protocol. This SAP details the proposed analyses and presentation of the data for the main paper(s) reporting the results for the WILL study. A separate document, named "WILL Final Report Template", contains the dummy tables of the final analysis and is referred to throughout this SAP.

The results reported in these papers will follow the strategy set out here. Subsequent analyses of a more exploratory nature will not be bound by this strategy, though they are expected to follow the broad principles laid down here. The principles are not intended to curtail exploratory analysis (e.g. to decide cut-points for categorisation of continuous variables), nor to prohibit accepted practices (e.g. transformation of data prior to analysis), but they are intended to establish rules that will be followed, as closely as possible, when analysing and reporting data.

Any deviations from this SAP will be described and justified in the final report or publication of the trial (using a table as shown in Appendix A). The analysis will be carried out by an appropriately qualified statistician, who should ensure integrity of the data during their data cleaning processes.

2. Background and rationale

The background and rationale for the trial are outlined in detail in the protocol. In brief, in the United Kingdom (UK), up to 55,000 pregnancies/year are complicated by chronic hypertension (diagnosed before pregnancy or at <20 weeks' gestation) or gestational hypertension (diagnosed at ≥20 weeks), and half of these women will reach term gestational age. Early term delivery (at 37-38 weeks) may reduce maternal complications and stillbirth, but it may also increase neonatal morbidity and costs, related primarily to the cost of maternal and fetal surveillance during expectant care and possibly increased Caesarean deliveries that are greater than the costs of labour induction[1-3].

To guide care for these high-risk women, the WILL trial compares a policy of delivery at 38 weeks, against usual care at term (or as clinical need dictates), with regards to maternal complications (and Caesareans), whilst ensuring that neonatal health is not compromised[4,5].

Among women with chronic[6] or gestational hypertension[1], observational data suggest that delivery between 38^{+0} and 39^{+6} weeks may optimise outcomes for the baby, by minimising stillbirth that rises in incidence with advancing gestational age at term, and neonatal morbidity that falls with advancing gestational age. However, observational studies are confounded by indication for delivery, so it is not possible to estimate with any certainty the impact of different gestations of delivery on perinatal outcomes. Also, the impact of planned delivery at term (i.e., at 37^{+0} to 41^{+6} weeks) on maternal morbidity or Caesarean could not be assessed.

There are no definitive trials that have established how best to manage women with chronic or gestational hypertension who reach 37 weeks and require delivery at term. There are limited data to inform care of women who would be eligible for WILL (i.e., 50 women with chronic hypertension in Hamed 2014[7] and at most, 188 women with gestational hypertension in the 37^{+0-6} week subgroup of HYPITAT I[8]). These data suggest that earlier delivery at term may be beneficial to women with chronic or gestational hypertension, without increasing risk to babies or Caesarean delivery. However, the number of women enrolled was very small, and both trials were conducted in settings (Egypt and the Netherlands) where compared with the UK, there are differences in antenatal care, including less frequent use of antihypertensives for Blood Pressure (BP) of ≥150/100mmHg (as advised by contemporaneous National Institute of Health and Care Excellence (NICE) guidance [CG107])[9].

WILL aims to address optimal timing of delivery for women with chronic or gestational hypertension who reach term gestational age and are otherwise well. The study will provide data for women to make informed choices about maternal and perinatal risk and the National Health Service (NHS) to plan services.

3. Trial objectives

The primary objective is to evaluate if planned early term delivery at 38^{+0} to 38^{+3} weeks' gestation, compared with usual care at term, in pregnant women with chronic or gestational hypertension that develops by 37^{+6}

weeks' gestation reduces a composite of 'poor maternal outcome', without unduly increasing neonatal care unit admission for ≥4 hours measured to hospital discharge or 28 days after delivery (whichever is earlier).

Secondary objectives are as follows: to evaluate the response of planned early term delivery at 38^{+0} to 38^{+3} weeks compared with usual care at term, in pregnant women with chronic or gestational hypertension that develops by 37^{+6} weeks on maternal and neonatal clinical outcomes and cost-consequence outcomes from an NHS perspective.

4. Trial methods

4.1. Trial design

WILL is a pragmatic, two-arm, parallel-group, open-label, multicentre, randomised controlled trial (with a 9-month internal pilot), with two co-primary outcomes: a maternal outcome assessing superiority and a neonatal outcome assessing non-inferiority (see Appendix B for trial schema).

The 9-month internal pilot was undertaken in centres chosen to be representative of sites overall (e.g. number of births and region) to test processes of the main trial prior to all centres opening. 'Stop-go' criteria were used by the Trial Steering Committee (TSC) and Data Monitoring Committee (DMC) in a joint meeting held at the end of the pilot period to review the data and examine whether the progression criteria had been met. It was recommended that recruitment to the main trial should proceed with no break and data from the pilot phase will be analysed together with the main trial data collected. For the progression rules, see Appendix D.

4.2. Trial interventions

Planned early term delivery at 38^{+0} to 38^{+3} weeks by labour induction (local protocol) or elective Caesarean.

Usual care at term, with maternal and fetal monitoring (local protocol), awaiting spontaneous labour or delivery indicated by clinical need (e.g., refractory severe hypertension or pre-eclampsia).

4.3. Primary outcomes

There are two co-primary outcomes defined: a maternal outcome assessing superiority and a neonatal outcome assessing non-inferiority.

MOTHER: composite of poor maternal outcome until primary hospital discharge home or 28 days after birth (whichever is earlier), defined as:

• Severe hypertension (i.e., systolic BP (sBP) ≥160 or diastolic BP (dBP) ≥110mmHg); or
• Maternal death; or
• Maternal morbidity defined as any of the following: Glasgow Coma Scale (GCS) <13; stroke; Transient Ischaemic Attack (TIA); eclampsia; blindness; uncontrolled hypertension; inotropic support; pulmonary oedema; respiratory failure; SpO_2 <90%; myocardial ischaemia or infarction; hepatic dysfunction, hepatic haematoma or rupture; acute kidney injury or dialysis; platelet count $<50 \times 10^9$/L; transfusion; or placental abruption. These were adapted from a Delphi consensus in hypertensive pregnancy[10, 11].

BABY: Neonatal care unit admission for ≥4 hours, until primary hospital discharge home or 28 days of life (whichever is earlier). By definition, stillbirths, neonatal deaths without admission, or neonatal deaths with admission for <4 hours are not counted in this outcome, but sensitivity analyses will be conducted (see Section 9.10). Neonatal admission is to any of the following types of units, according to definitions provided in the British Association of Perinatal Medicine (BAPM) 2011 classification of neonatal care[12]:

- Intensive care: This is care provided for babies who are the most unwell or unstable and have the greatest needs in relation to staff skills and staff to patient ratios.
- High dependency care: This is care provided for babies who require highly skilled staff but where the ratio of nurse to patient is less than intensive care.
- Special care: Special care is provided for babies who require additional care delivered by the neonatal

service but do not require either Intensive or High Dependency care.

This does not include transitional care because the baby is with the mother.

See Appendix E: Data manipulations on how the primary outcomes will be derived.

4.4. Secondary outcomes

These will be assessed until primary discharge home or 28 days after delivery, whichever is earlier, unless otherwise specified.

Outcomes indicated by an asterisk (*) will be presented with a treatment effect and confidence interval (CI). All other outcomes will be presented with summary statistics only.

Key Maternal

- Caesarean delivery*
    - Indications will be presented descriptively (i.e., maternal, fetal, or both, not mutually exclusive)

Other Maternal

- Instrumental vaginal delivery or Caesarean delivery (vs. spontaneous vaginal delivery)*
    - Indications will be presented descriptively (i.e., maternal, fetal, or both, not mutually exclusive)
- Infection of the stitches from the Caesarean wound, episiotomy, or vaginal tear, as applicable will be presented descriptively, assessed at six weeks postpartum
- Individual components of maternal co-primary outcome, up to discharge or 28 days postpartum (whichever is earlier)
    - sBP ≥160mmHg or dBP ≥110mmHg (measured twice, 15min apart) which the national Mothers and Babies: Reducing Risk through Audits and Confidential Enquiries across the UK (MBRRACE-UK) reports (of Confidential Enquiry into Maternal Deaths and Morbidity) states is a clinical emergency requiring urgent treatment*
    - Maternal death*
    - Maternal morbidity as adapted from Delphi consensus in hypertensive pregnancy(10;11):
        - GCS<13*;
        - Stroke (i.e., acute symptoms of focal brain injury that have lasted over 24 hours, with type [ischaemic or haemorrhage] confirmed by neuroimaging) *;
        - Transient ischaemic attack (i.e., acute symptoms of focal brain injury that have lasted less than 24 hours) *;
        - Eclampsia (i.e., the onset of convulsions in a woman with pre-eclampsia not attributable to other causes) *;
        - Blindness (i.e., partial/complete, or either retinal or cortical). Retinal detachment is defined as the peeling away of the retina from its underlying layer of support tissue diagnosed by ophthalmological exam. Cortical blindness is defined as loss of visual acuity in the presence of intact pupillary response to light*;
        - Uncontrolled hypertension (i.e., need for a third parenteral antihypertensive agent (hypertension requiring administration of 3 or more different parenteral [intravenous or intramuscular] antihypertensive agents within a 12 hour period) *;
        - Inotropic support (i.e, use of vasopressors to keep sBP > 90 mm Hg or a Mean Arterial Pressure (MAP) >70 mmHg) *;
        - Pulmonary oedema (i.e., excess fluid in the lungs diagnosed clinically with one/more of oxygen saturation < 95%, directive treatment (e.g., diuretic therapy), or x-ray confirmation) *;
        - Respiratory failure (i.e., intubation, ventilation either by endotracheal tube or non-invasively, or need for > 50% oxygen for > 1 hr, none of which is due to Caesarean delivery) *;
        - SpO_2 <90%*;
        - Myocardial ischaemia or infarction (i.e., by characteristic Electrocardiogram (ECG) changes

and markers of myocardial necrosis) *;
        - Hepatic dysfunction (i.e., International Normalised Ratio (INR) >1.2 in the absence of disseminated intravascular coagulation (DIC) or treatment with warfarin, OR, in the presence of DIC or treatment with warfarin: either mixed hyperbilirubinemia >1.0 mg/dL (or >17 µM) or hypoglycaemia <45 mg/dL (<2.5 mM) in the absence of insulin) *;
        - Hepatic haematoma or rupture (i.e., presence of a blood collection under the hepatic capsule as confirmed by imaging or at laparotomy) *;
        - Acute kidney injury (i.e., serum creatinine >150µM in the absence of a baseline serum creatinine; or rise in serum creatinine ≥26µM within 48 hours, or >50% rise in serum creatinine within the past 7 days; or urine output <0.5ml/kg/hr for >6hr) or new dialysis (of any type) *;
        - Platelet count <50x10^9/L*;
        - Transfusion (of any blood product) *; or
        - Placental abruption, diagnosed either (i) clinically by abdominal pain or uterine contractions of sudden onset with one/more of: vaginal bleeding other than show, intrauterine fetal death or DIC; (ii) by the presence of a retroplacental clot at the time of delivery; or (iii) by placental pathology demonstrating the presence of retroplacental clot or histological findings of a chronic abruption*
- Poor maternal outcome (assessed as one or more of the components [presented descriptively] of the maternal co-primary outcome) measured at six weeks postpartum (as assessed post-discharge after birth by maternal questionnaire)*
- Elevated liver enzymes (Aspartate Aminotransferase (AST) or Alanine Aminotransferase (ALT) >40 IU/L)*
- Platelet count <100x10^9/L*
- Pre-eclampsia by International Society for the Study of Hypertension in Pregnancy (ISSHP) 2018 criteria, defined as gestational hypertension accompanied by one or more of the following new-onset conditions at ≥20 weeks: (i) proteinuria; (ii) serum creatinine ≥90µM; (iii) elevated AST or ALT to >40 IU/L; (iv) neurological complications including eclampsia, altered mental status [as measured by GCS<13], blindness, stroke, clonus, severe headache, persistent visual scotomata; (iv) haematological complications (i.e., platelet count <150x10^9/L, DIC, haemolysis); or (v) uteroplacental dysfunction (including fetal growth restriction defined as birthweight <10th centile, abnormal umbilical artery Doppler waveform analysis, or stillbirth)*
- Postpartum Haemorrhage (PPH) (perceived abnormal bleeding following birth and either hypotension or medical/surgical intervention for postpartum haemorrhage)*
- Sepsis (known or suspected maternal infection with two or more of Quick Sequential Organ Failure Assessment (Quick Sequential Organ Failure (qSOFA)) criteria: respiratory rate ≥22/min, altered mentation, or sBP ≤100mmHg)*
- Intensive Treatment Unit (ITU) admission (to receive advanced respiratory support alone or monitoring and support for two or more organ systems)*
- Potential co-interventions (post-randomisation), before birth admission unless otherwise specified:
    - Antihypertensive therapy taken
        - Antepartum (even after admission for birth)*
            - Type of antihypertensive therapy presented descriptively (as labetalol, methyldopa, nifedipine, other [specify])
        - Postpartum*
            - Type of antihypertensive therapy presented descriptively (as labetalol, methyldopa, nifedipine, other [specify])
        - Both antepartum and postpartum*
    - Magnesium sulphate (antepartum or postpartum)*
    - Bedrest at home*
    - Use of home BP monitoring*
    - Maternal blood or urine testing at the laboratory prior to birth admission*, and number of such episodes of testing (median [Interquartile Range (IQR)])
    - Seen as outpatient (in office/clinic)* and number of visits (median [IQR])

- Seen as outpatient (in her home)* and number of visits (median [IQR])
- Where available, seen in medical, day, or maternity assessment unit* and number of visits (median [IQR])
- Seen in an acute care area (such as Accident & Emergency) for urgent/emergent visit other than in labour* and number of such visits (median [IQR])
- Number of antenatal admission days prior to birth (median [IQR])*
- Underwent fetal cardiotocography*
- Underwent fetal ultrasound*

- Clinical indications for birth, presented descriptively
- Maternal satisfaction assessed at hospital discharge or 28 days postpartum (whichever is earlier), as measured by the Childbirth Experience Questionnaire, assessed as the overall score, and domain scores (i.e., own capacity, professional support, perceived safety, and participation)*

Fetal/Neonatal

- Neonatal care unit admission ≥4 hours, assessed to 28 days after birth
- Indication for neonatal care unit admission for ≥4 hours as a respiratory problem*, as identified by the clinical team by the principle indication for admission on the BadgerNet discharge summary (with the clinical diagnosis presented descriptively, as meconium aspiration syndrome, pneumonia,pneumothorax/pneumomediastinum, transient tachypnoea of the newborn, or 'other' [specified])
    - Other indications, as identified clinically, will be presented descriptively (e.g., 5-min Apgar score <7, birthweight <10th centile, birthweight >90th centile, sepsis work-up, hyper- or hypoglycaemia, or other)
- Respiratory morbidity, defined as the need for supplemental oxygen and/or positive pressure ventilation beyond the initial resuscitation period*
- Clinical respiratory problem, defined as meconium aspiration syndrome, pneumonia, pneumothorax/pneumomediastinum, transient tachypnoea of newborn, or other [unspecified])*
- Chest x-ray, N performed, N abnormal and nature of abnormality (i.e., meconium aspiration syndrome, pneumonia, pneumothorax/pneumomediastinum, transient tachypnoea of newborn, or other [unspecified])*
- Hypoxic Ischemic Encephalopathy (HIE), defined as therapeutic hypothermia for ≥72 hours*
- Sepsis requiring antibiotics for at least five days, with confirmed blood or cerebrospinal fluid culture*
- Major operation (laparotomy, thoracotomy, craniotomy, or other)*
- Birthweight (centiles)*
- Apgar scores (recorded at 1, 5, and 10 minutes)*
- Stillbirth (i.e., death of a fetus after randomisation)*
- Neonatal death (of a liveborn infant within the first 28 days of birth)*
- Breastfeeding established*
- Exclusive breastfeeding*

Health Economics (Aa separate document will detail the analysis plan for the health economic outcomes)

- Cost-consequence analysis from NHS perspective (enrolment to hospital discharge)

See Appendix E: Data manipulations for how the secondary outcomes will be derived.

4.5. Timing of outcome assessments

The schedule of trial procedures and outcome assessments are given in Appendix C.

4.6. Randomisation

Women will be randomised at 37^{+0} to 37^{+6} weeks gestational age, at an individual level in a 1:1 ratio to either:

1. Planned early term delivery at 38^{+0-3} weeks, or
2. Usual care at term

Randomisation will be provided by a computer-generated programme hosted by the University of Birmingham using a minimisation algorithm to ensure balance in the treatment allocation over the following variables:

- Randomising centre (i.e. the recruiting centre);
- Hypertension type (chronic/gestational hypertension);
- Prior Caesarean (yes/no).

The minimisation balance will be monitored by the central Trial Team at Birmingham Clinical Trials Unit (BCTU). A 'random element' will be included in the minimisation algorithm, so that each participant has a probability (unspecified here), of being randomised to the opposite intervention that they would have otherwise received. Full details of the algorithm used will be stored in a confidential document at BCTU.

4.7. Sample size

A total sample size of 1,080 women (540 per group) will be required to detect an 8% absolute reduction in the maternal co-primary outcome from 25% to 17% (Risk Ratio (RR) 0.68; estimate of 25% based on women who experienced poor maternal outcome at term in the CHIPS Trial[13] [unpublished data]), assuming 90% power, a two-sided type I error rate of 5%, using the standard method of difference between proportions and based on a superiority hypothesis. The anticipated relative risk reduction in our maternal co-primary outcome was chosen because a similar reduction was seen in HYPITAT I[8], and this effect size was shown to be of sufficient magnitude to change clinical practice in the Netherlands[14]

Assuming a control group (usual care at term) incidence of our neonatal co-primary (safety) outcome (of neonatal care unit admission ≥4 hrs) of 23%, a sample size of 1,080 will achieve 94% power to provide a non-inferiority margin of difference in incidence between groups of 9% (i.e., the upper bound of the 95% CI around the risk difference is <0.09), and 88% power to provide a margin of 8% (one-sided 2.5% type I error rate, non-inferiority hypothesis). The incidence in the control group of our neonatal co-primary (safety) outcome of high-level neonatal care for ≥4hr is based on a rate of 23% in HYPITAT[3].

This sample size will also provide sufficient power to detect a 10% decrease in Caesarean delivery assuming a control group risk of 45% (45% to 35%; 90% power; 5% type I error rate, superiority hypothesis), similar to changes in HYPITAT I[8]. In this way, women and clinicians will have the information that they require (about complications for them and their babies, and Caesarean delivery) in order to make informed decisions about care.

Given the short time between consent (at 36^{+0}-37^{+6} weeks), randomisation (at $37^{+0\text{-}6}$ weeks), and birth (by 41^{+6} weeks, even in the usual care arm), no adjustment has been made for loss to follow-up or drop-outs.

4.8. Framework

There are two primary objectives of the trial.

The first objective is to assess the superiority of the intervention of 'planned early term delivery at $38^{+0\text{-}3}$ weeks' to 'usual care at term' on the composite poor maternal co-primary outcome.

The second objective is to assess the non-inferiority of 'planned early term delivery at $38^{+0\text{-}3}$ weeks' to 'usual care at term' on the neonatal co-primary outcome.

Null hypotheses have not been stated as we are not presenting p-values or discussing statistical significance, but will focus on the treatment effect measures and 95% CIs.

4.9. Interim analyses and stopping guidance

There is a separate DMC reporting template that has been agreed by the DMC, and this includes agreement on which outcomes will be reported at interim analyses. The statistical methods stated in this SAP will be followed for the outcomes included in the DMC report, where possible.

During the main phase of the trial using interim data and other evidence from relevant studies, the DMC will inform the TSC if, in its view, there is proof beyond reasonable doubt that the data indicate that the trial, or part of it, should be terminated. A decision to inform the TSC of such a finding will in part be based on statistical considerations. Appropriate proof beyond reasonable doubt cannot be specified precisely. A difference of at least 3 standard errors in the interim analysis of a major endpoint may be needed to justify halting, or modifying, such a study prematurely.

4.10. Internal Pilot Progression Rules

'Stop-go' criteria were used by the TSC and DMC in a joint meeting held at the end of the internal pilot period to review the data and examine whether the progression criteria were met. See Appendix D for the detailed 'Stop-go' criteria.

4.11. Timing of final analysis

The final analysis for the trial will occur once the database is locked (see Data Management Plan for details). Where possible this will be after the last randomised woman is discharged following the birth of her baby, the six weeks postpartum follow up is complete, the corresponding outcome data have been entered onto the trial database, and the data validated for analysis. This assumes that the trial has not been stopped early for any reason, such as based on DMC advice or funding body request.

4.12. Timing of other analyses

The consent form will record if potential participants would be willing to be contacted in the future about other studies tracking the long-term development and progress of their child. The analysis of any longer-term follow-up that occurs will be documented in a separate SAP.

4.13. Trial comparisons

All references in this document to 'group' refer to planned early term delivery at $38^{+0\text{-}3}$ weeks or usual care at term. In addition, descriptive statistics will be presented for women who consented but who were not randomised.

5. Statistical Principles

5.1. Confidence intervals and p-values

All estimates of differences between groups will be presented with two-sided 95% CIs.

5.2. Adjustments for multiplicity

No correction for multiple testing will be made. There are two co-primary outcomes, one for the mother and one for the baby. As these outcomes will be considered in combination, neither hierarchical testing nor adjustment for multiplicity were viewed as appropriate.

5.3. Analysis populations

All primary analyses (primary and secondary outcomes including safety outcomes) will be by intention-to-treat (ITT). Women and babies of women will be analysed in the intervention group to which the women were randomised, and all women and babies of women shall be included, whether or not women received the allocated intervention, to avoid any potential bias in the analysis. Although the neonatal co-primary outcome is based on a non-inferiority hypothesis, an ITT analysis (rather than a per-protocol analysis) was considered the most appropriate primary analysis since the intervention is about *planned* timing of delivery (as opposed to *actual* timing of delivery).

Complier Average Causal Effect (CACE) will also be carried out for the co-primary outcomes (see section 9.10 for further details regarding sensitivity analyses).

In addition, summary statistics on baseline data and maternal & neonatal outcomes prior to hospital discharge will be presented for women who were consented but could then not be randomised (see WILL Final Report Template).

5.4. Definition of adherence

Due to the nature of our control group (usual care at term) we will only define adherence (in a binary context; yes/no) in the planned early term delivery group. Women will be considered adherent if the timing of delivery initiation is between 38^{+0} and 38^{+3} weeks inclusive. Women who have spontaneous onset of labour or initiation of labour due to clinical need before 38^{+0} weeks will be considered adherent as the intervention is one of planned early term delivery at $38^{+0\text{-}3}$ weeks. Women will be considered non-adherent if the timing of delivery

initiation is not between 38^{+0} and 38^{+3} weeks inclusive because of busy labour ward or theatre schedules, or the reason for the timing of delivery was clinician preference or the woman's preference; timing of delivery based on clinician preference or the woman's preference will require completion of a Protocol Deviation Form.

It is possible that women in the planned early term delivery (at 38^{+0-3} weeks) group may be delivered at a gestational age outside the 38^{+0-3} window. Reasons for timing of delivery (i.e., spontaneous, based on clinical need etc.) will be recorded on the case report forms (CRF) and monitored. Reasons for midwife- or hospital doctor- initiated delivery before 38^{+0} weeks may include development of pre-eclampsia, a maternal end-organ complication associated with hypertensive disease (e.g., pulmonary oedema), or abnormal fetal heart rate or pattern. Reasons for failure to initiate delivery (by induction or elective Caesarean) before 38^{+3} weeks may include busy hospital induction or theatre schedules that allow for initiation of delivery only after 38^{+3} weeks.

The gestational age at birth will be monitored in both the 'planned early term delivery at 38^{+0-3} weeks' and the 'usual care at term' groups, to encourage adequate separation of gestational age at birth between the trial arms.

5.5. Handing protocol deviations

A protocol deviation is defined as a failure to adhere to the protocol. Examples include incorrectly applying the inclusion/exclusion criteria, timing of delivery based on clinician preference or the woman's preference, collecting data incorrectly, or conducting follow-up visits outside the specified visit window or not at all. We will apply a strict definition of ITT and include in the analysis all participants, as per the ITT population described in Section 5.3, regardless of deviation from the protocol[15]. This does not include participants who have specifically withdrawn consent for use of their data in the first instance; however, these outcomes will be explored as per other missing responses (see Section 9.3).

A postpartum assessment will be made at 6 weeks post randomisation via a self-completed questionnaire by the mother. All outcomes recorded on this questionnaire will be considered regardless of the timing of completion.

5.6. Unblinding

This is an unmasked study. The DMC will review interim data without being masked to treatment allocation. Unblinding of the Trial Statistician to the allocated intervention code will take place when monitoring and cleaning the data, when producing each interim DMC report, and after the database is locked for final analysis.

After the database is locked for final analysis, the table of key baseline characteristics will be reviewed by the chief investigator masked to allocation. This will identify any variables (ethnicity, Body Mass Index (BMI), prior severe hypertension in the index pregnancy, antihypertensive therapy at randomisation, gestational diabetes mellitus at randomisation and smoking status at randomisation) with potential imbalance between groups, to inform a sensitivity analysis where additional covariate adjustment including these baseline characteristics is conducted. See Section 9.10 for further details regarding sensitivity analyses.

6. Trial population

6.1. Recruitment

A flow diagram (as recommended by CONSORT[16]) will be produced to describe the participant flow through each stage of the trial. This will include information on the number (with reasons) of losses to follow-up (drop-outs and withdrawals) over the course of the trial. A template for reporting this is given in Section 4 of the WILL Final Report Template.

6.2. Baseline characteristics

The trial population will be tabulated as per Section 6 of the WILL Final Report Template. Categorical data will be summarised by number of participants, counts and percentages. Continuous data will be summarised by the number of participants, mean and standard deviation if deemed to be normally distributed, or number of participants, median and interquartile range if data are skewed, and ranges if appropriate. Tests of statistical significance will not be undertaken, nor CIs presented[17].

The minimisation, demographic and other baseline variables will be described for the total population, for the two randomised groups separately, and for those women who were consented but were not randomised. See Section 6 of the WILL Final Report Template.

7. Intervention

7.1. Description of the intervention

A template for reporting information on the intervention is given in Section 7 of the WILL Final Report Template and described below in 7.2.

7.2. Adherence to allocated intervention

Frequencies and percentages in the planned early term delivery (at 38^{+0-3} weeks) group by the adherence categories as defined in Section 5.4 will be produced. In both groups, the median gestational age at initiation and birth will also be provided with corresponding IQRs and ranges. A plot of gestational age through time by group will also be produced. A template for reporting adherence is given in Section 7 of the WILL Final Report Template.

8. Protocol deviations

Frequencies and percentages by group will be tabulated for the protocol deviations as per Section 10 of the WILL Final Report Template.

9. Analysis methods

9.1. Covariate adjustment

In the first instance, intervention effects between groups for all outcomes will be adjusted for the minimisation parameters listed in Section 4.6. Centre will be treated as a random intercept in the models, and all other factors as fixed effects. If there is concern that the allocated groups may not be balanced for a key baseline covariate(s), a sensitivity analysis will be considered to allow for further adjustment (see Section 5.6).

If covariate adjustment is not possible (e.g. the model does not converge), centre will be removed first. If this reduced model still fails to converge, unadjusted estimates will be produced.

In all cases, it will be made clear in the final report why an alternative model was fitted (e.g. not possible due to low event rate/lack of model convergence).

9.2. Distributional assumptions and outlying responses

Distributional assumptions (e.g. normality of regression residuals for continuous outcomes) will be assessed visually prior to analysis; although in the first instance, the proposed primary method of estimation in this analysis plan will be followed. For continuous outcomes, if responses are considered to be particularly skewed and/or distributional assumptions violated, sensitivity analyses will be performed (see Section 9.10) where medians and interquartile ranges will be reported alongside unadjusted differences in medians (and corresponding 95% CI) using bootstrapping methods (repetitions=1000, seed=123456).

If extreme values are apparent and considered to be affecting the integrity of the analysis, consideration will be given to removing the outlying response(s) in sensitivity analyses (see Section 9.10).

9.3. Handling missing data

In the first instance, analysis will be completed on received data only with every effort made to follow-up participants to minimise any potential for bias. To examine the possible impact of missing data on the results, and to make sure we are complying with the ITT principle, sensitivity analysis will be performed on the co-primary outcome measures only[18]. See Section 9.10 for further details regarding sensitivity analyses.

9.4. Data manipulations

See Appendix E: Data manipulations.

9.5. Analysis methods – primary outcomes

A template for reporting the co-primary outcomes is given in in Section 8 of the WILL Final Report Template. See section 9.1 for covariate adjustment and model convergence.

The maternal co-primary outcome measure will be summarised using frequencies and percentages. A logistic regression model, using a logit link function will be used, followed by the standardisation approach for covariate adjustment to calculate an adjusted RR (and corresponding 95% CI) and an adjusted risk difference (RD) (and corresponding 95% CI).

The analysis of the neonatal co-primary outcome measure will follow a similar method as for the maternal co-primary outcome. All babies born to women randomised will be included, no matter the status of the baby at birth (i.e., alive or stillborn).

9.6. Analysis methods – secondary outcomes

A template for reporting the secondary outcomes is given in Section 8 of the WILL Final Report Template.

Analyses for the binary secondary outcomes will be performed as per the co-primary outcomes.

For continuous secondary outcomes (e.g. the Childbirth Experience Questionnaire scores), means and standard deviations will be reported alongside adjusted mean differences (with 95% CIs) estimated using a linear regression model.

For count secondary outcomes (e.g. co-interventions: antenatal admission prior to birth), medians and interquartile rages will be reported alongside an adjusted incidence rate ratio (IRR) (and corresponding 95% CI) estimated using a Poisson regression model, or if there is evidence of over-dispersion a negative binomial regression model will be used. The natural logarithm of time in days from the date of randomisation to the date of birth will be added as an offset variable. In the event that we observe an excess of zero counts, we will consider estimating the adjusted IRR (and corresponding 95% CI) using a zero-inflated Poisson regression model, or if there is evidence of over-dispersion a zero-inflated Negative Binomial regression model.

9.7. Analysis methods – exploratory outcomes and analyses

Any data that do not form a pre-specified outcome will be presented using simple summary statistics by intervention group (i.e., numbers and percentages for binary data and means [or medians] and standard deviations [or inter-quartile ranges] for continuous normal [or non-normal] data). These will be reported in the secondary outcomes table in Section 8 of the WILL Final Report Template.

Women who were consented at 36^{+0}-37^{+6} weeks, but who were not randomised, will be presented using a descriptive analyses of the maternal and neonatal co-primary outcomes, caesarean delivery, and other secondary outcomes (except potential co-interventions and maternal satisfaction) to hospital discharge only. An extra column has been added in each table for reporting these in the WILL Final Report Template.

We will also perform a range of exploratory analyses for publication separately, such as investigation of the impact of including severe hypertension as part of the maternal co-primary composite outcome on the interpretation of our results. A separate SAP will be developed detailing these analyses.

9.8. Safety data

The number and percentage of participants experiencing any Serious Adverse Events (SAEs) or Related Unexpected SAEs (RUSAEs) will be presented by intervention group. The total number of SAEs and RUSAEs in each group will also be given along with a descriptive table of the details of the events.

A template for reporting this safety data is given in Section 9 of the WILL Final Report Template.

9.9. Planned subgroup analyses

Subgroup analyses will be undertaken on the two co-primary outcomes for subgroups based on the following variables:

(i) With the exception of recruiting centre, variables used in the minimisation algorithm (i.e. hypertension type [chronic or gestational hypertension] and prior Caesarean [yes/no])
(ii) other baseline variables of prognostic significance pre-specified as ethnicity [White/Black/Arab/Asian/Other or declined to answer], BMI [Underweight (<18.5)/Normal weight (18.5 – 24.9)/ Overweight (25.0 – 29.9)/ Obesity (≥30.0)], prior severe hypertension in the index pregnancy [yes/no], antihypertensive therapy at randomisation [yes/no], gestational diabetes mellitus at randomisation [yes/no], and smoking status at randomisation [yes/no].

Among the aforementioned subgroup analyses the following will be assumed as key subgroup analyses: ethnicity, BMI, gestational diabetes, and antihypertensive therapy.

The effects of these subgroups will be examined by including a treatment group by subgroup interaction parameter in the regression model. P-values from tests for statistical heterogeneity will be presented alongside the effect estimate and 95% CI within each subgroup. In addition to this, a ratio(s) (and 95% CI(s)) will be provided to quantify the difference between the treatment effects estimated within each subgroup. For hypertension type, prior Caesarean, gestational diabetes, antihypertensive therapy and smoking status, as these subgroup variables contain only two levels, one ratio will be provided ('gestational', 'no', 'no', 'no' and 'no' will be regarded as the reference groups respectively). For ethnicity, which has five levels, 'white' will be considered as the reference group and four ratios will be provided for the other levels in comparison to this reference. For BMI, which has four levels, 'normal weight (18.5 – 24.9)' will be considered as the reference group and three ratios will be provided for the other levels in comparison to this reference. The results of subgroup analyses will be treated with caution and will be used for the purposes of hypothesis generation only[19].

A template for reporting subgroup analyses is given in Section 8 of the WILL Final Report Template.

9.10. Sensitivity & Supportive analyses

The following sensitivity analyses will be limited to continuous outcomes and will consist of:

- A sensitivity analysis to assess distributional assumptions (where applicable, as described in section 9.2).
- A sensitivity analysis to assess outliers (where applicable, as described in section 9.2).

The following sensitivity analyses will be limited to both co-primary outcomes and the key secondary outcome and will consist of:

- Adjustment for baseline covariates if an imbalance is considered clinically important (on the ITT population).
- A restricted analysis whereby women and their babies in the planned early term delivery at 38^{+0-3} weeks group will be excluded if the women delivered before 38^{+0} weeks and women and their babies in the usual care at term group will be excluded if the women delivered before 39^{+0} weeks, in order to assess the robustness of the results when the two groups have at least one week separation at women's gestational ages at birth.
- Heterogeneity of treatment effect due to the change in the usual care arm, will be explored by including a treatment by group-change interaction parameter to the two primary analysis models. Group 1 will consist of those women and their associated babies who were randomised before the change was implemented in the usual care arm, and group 2 will consist of those women and their associated babies who were randomised after the change was implemented in the usual care arm.
- A CACE analysis be performed. See section 5.4 for definition of adherence. One-sided compliance will be assumed, so the proportion of always-takers will be 0.

The following sensitivity analysis will be undertaken for the maternal co-primary outcome only:

- The rate of missing maternal co-primary outcome data is expected to be extremely low (<1%) given that women are recruited near the end of pregnancy prior to delivery. If missing data exceeds 1% an analysis to assess the impact will be conducted. This analysis will explore the possibility that missing responses are 'missing not at random' (MNAR) using a tipping point approach.

The following sensitivity analyses will be undertaken for the neonatal co-primary outcome only:

- A restricted analysis including liveborns only.

- Since stillbirths, neonatal deaths without admission, or neonatal deaths with admission for <4 hours are not counted in the neonatal co-primary outcome (i.e. included as denominator only), the following sensitivity analysis will be conducted: Including in the numerator, stillbirths or babies who either died without admission to neonatal care, or died following admission to neonatal care for <4 hours, where newborn death is of a liveborn infant until primary discharge home or within the first 28 days of birth, whichever is earlier.

- The rate of missing neonatal co-primary outcome data is expected to be extremely low (<1%) given that women are recruited near the end of pregnancy prior to delivery. If missing data exceeds 1% an analysis to assess the impact will be conducted. This analysis will explore the possibility that missing responses are 'missing not at random' (MNAR) using a tipping point approach.

A template for reporting the sensitivity analyses is given in Section 8f the WILL Final Report Template.

10. Analysis of sub-randomisations

Not applicable.

11. Health economic analysis

As indicated in the protocol there will also be an economic analysis. The details of this analysis are documented separately in a Health Economics Analysis Plan (HEAP).

12. Statistical software

Statistical analysis will be undertaken in the following statistical software packages: SAS (version 9.4 or higher) and/or Stata (version 17.0 or higher).

13. References

(1) Cruz MO, Gao W, Hibbard JU. What is the optimal time for delivery in women with gestational hypertension? Am J Obstet Gynecol 2012 Sep;207(3):214-6.
(2) Hutcheon JA, Lisonkova S, Magee LA, von DP, Woo HL, Liu S, et al. Optimal timing of delivery in pregnancies with pre-existing hypertension. BJOG 2011 Jan;118(1):49-54.
(3) Vijgen SM, Koopmans CM, Opmeer BC, Groen H, Bijlenga D, Aarnoudse JG, et al. An economic analysis of induction of labour and expectant monitoring in women with gestational hypertension or pre-eclampsia at term (HYPITAT trial). BJOG 2010 Dec;117(13):1577-85.
(4) National Statistics. Vital Statistics: Population and Health Reference Tables. www ons gov uk 2015.
(5) Cluver C, Novikova N, Koopmans CM, West HM. Planned early delivery versus expectant management for hypertensive disorders from 34 weeks gestation to term. Cochrane Database Syst Rev 2017 Jan 15;1:CD009273.
(6) Hutcheon JA, Lisonkova S, Joseph KS. Epidemiology of pre-eclampsia and the other hypertensive disorders of pregnancy. Best Pract Res Clin Obstet Gynaecol 2011 Aug;25(4):391-403.
(7) Hamed HO, Alsheeha MA, Abu-Elhasan AM, Abd Elmoniem AE, Kamal MM. Pregnancy outcomes of expectant management of stable mild to moderate chronic hypertension as compared with planned delivery. Int J Gynaecol Obstet 2014 Oct;127(1):15-20.
(8) Koopmans CM, Bijlenga D, Groen H, Vijgen SM, Aarnoudse JG, Bekedam DJ, et al. Induction of labour versus expectant monitoring for gestational hypertension or mild pre-eclampsia after 36 weeks' gestation (HYPITAT): a multicentre, open-label randomised controlled trial. Lancet 2009 Sep 19;374(9694):979-88.
(9) The management of hypertensive disorders during pregnancy: NICE clinical guideline 107. www nice org/cg 107 2010.
(10) von Dadelszen P, Payne B, Li J, Ansermino JM, Broughton PF, Cote AM, et al. Prediction of adverse maternal outcomes in pre-eclampsia: development and validation of the fullPIERS model. Lancet 2011 Jan 15;377(9761):219-27.
(11) Duffy JM, van 't HJ, Gale C, Brown M, Grobman W, Fitzpatrick R, et al. A protocol for developing, disseminating, and implementing a core outcome set for pre-eclampsia. Pregnancy Hypertens 2016 Oct;6(4):274-8.

(12) British Association of Perinatal Medicine, Categories of Care (http://www.bapm.org/publications/documents/guidelines/CatsofcarereportAug11.pdf). 2011.
(13) Magee LA, von Dadelszen P., Rey E, Ross S, Asztalos E, Murphy KE, et al. Less-tight versus tight control of hypertension in pregnancy. N Engl J Med 2015 Jan 29;372(5):407-17.
(14) van der Tuuk K, Koopmans CM, Groen H, Mol BW, van Pampus MG. Impact of the HYPITAT trial on doctors' behaviour and prevalence of eclampsia in the Netherlands. BJOG 2011 Dec;118(13):1658-60.
(15). Gupta SK. Intention-to-treat concept: A review. Perspect Clin Res. 2011;2(3):109-112.
(16) Schulz KF, Altman DG, Moher D, for the CONSORT Group. CONSORT 2010 Statement: updated guidelines for reporting parallel group randomised trials. BMJ. 2010;340:c332.
(17) Altman DG, Dore CJ. Randomisation and baseline comparisons in clinical trials. Lancet. 1990;335:149–53.
(18) White IR, Horton NJ, Carpenter J, Pocock SJ. Strategy for intention to treat analysis in randomised trials with missing outcome data. BMJ. 2011;342:d40.
(19) Wand R, Lagakos SW, Ware JH, Hunter DJ, Drazen JM. Reporting of subgroups analyses in clinical trials. NEJM. 2007;357:2189-94.
(20) Villar J, Cheikh L, Victora CG, Ohuma EO, Bertino E, Altman DG, Lambert A, Papageorghiou AT, Carvalho M, Jaffer YA, Gravett MG, Purwar M, Frederick IO, Noble AJ, Pang R, Barros FC, Chumlea C, Bhutta ZA, Kennedy SH, for the International Fetal and Newborn Growth Consortium for the 21st Century (INTERGROWTH-21st). International standards for newborn weight, olength, and head circumference by gestational age and sex: the Newborn Cross-Sectional Study of the INTERGROWTH-21ST Project. Lancet 2014;384:857-68.
(21) Dencker, A., Taft, C., Bergqvist, L. et al. Childbirth experience questionnaire (CEQ): development and evaluation of a multidimensional instrument. BMC Pregnancy Childbirth 10, 81 (2010). https://doi.org/10.1186/1471-2393-10-81.
(22) Walker, K.F., Wilson, P., Bugg, G.J. et al. Childbirth experience questionnaire: validating its use in the United Kingdom. BMC Pregnancy Childbirth 15, 86 (2015). https://doi.org/10.1186/s12884-015-0513-4.
(23) Chatfield A, Caglia JM, Dhillon S, Hirst J, Cheikh Ismail L, Abawi K et al for the International Fetal and Newborn Growth Consortium for the 21st Century (INTERGROWTH-21st). Translating research into practice: the introduction of the INTERGROWTH-21st package of clinical standards, tools and guidelines into policies, programmes and services. BJOG 2013;120 (Suppl. 2): 139–142.

Appendix A: Deviations from SAP

This report below follows the statistical analysis plan dated *<insert effective date of latest SAP>* apart from following:

Section of report not following SAP	Reason
<insert section>	*<insert, e.g. exploratory analyses request by TMG>*

Appendix B: Trial schema

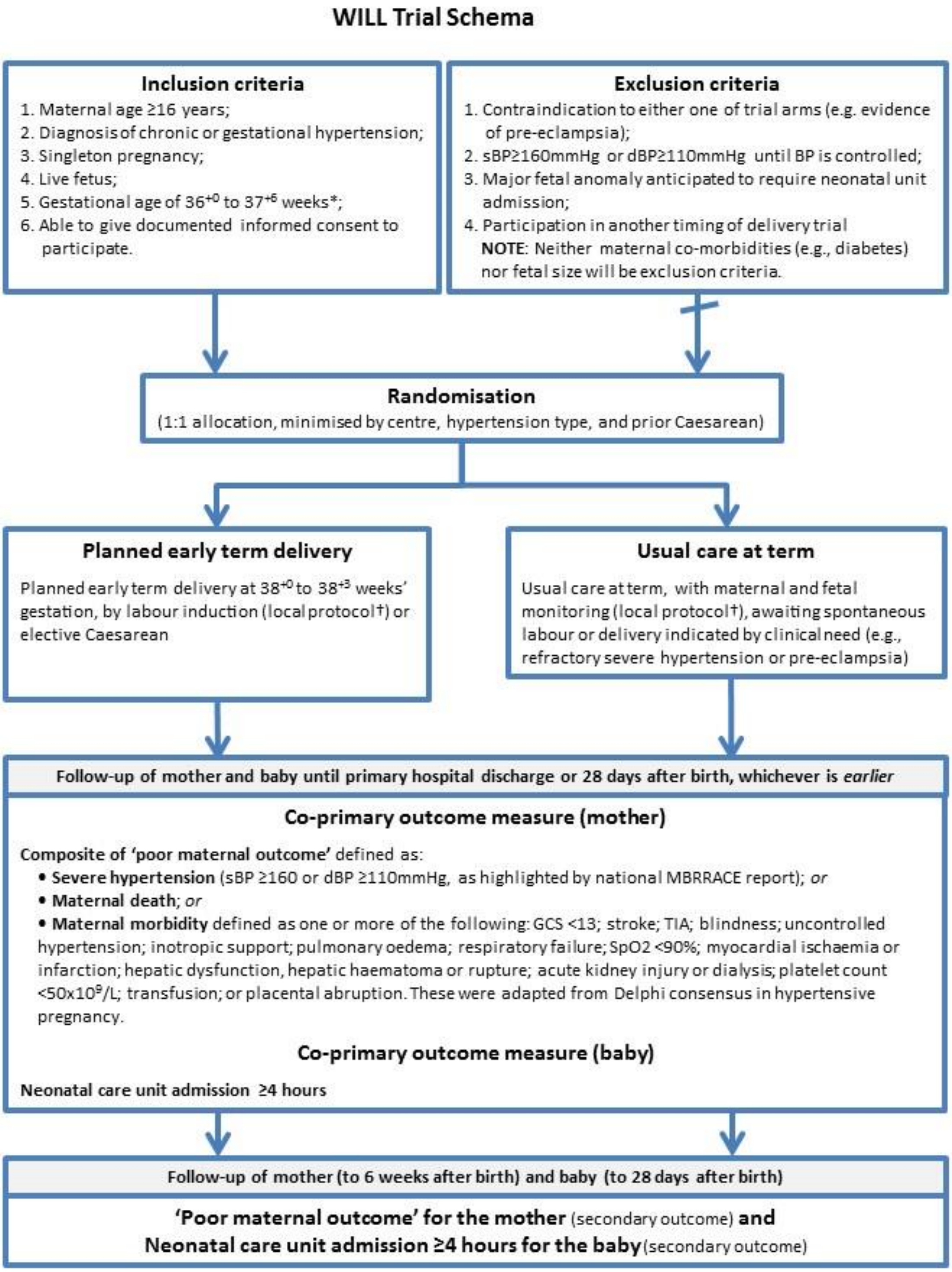

dBP (diastolic blood pressure), GCS (Glasgow Coma Scale), sBP (systolic blood pressure), SpO2 (peripheral capillary oxygen saturation), TIA (transient ischaemic attack)

**Women will be consented at 36^{+0} to 37^{+6} weeks' gestation, but will be randomised if they remain undelivered and well, from 37^{+0} to 37^{+6} weeks' gestation. This approach should optimise recruitment, minimise the number of women (<20%) who may go into spontaneous labour or require delivery for maternal/fetal reasons prior to 38^{+0} weeks' gestation, and allow for sufficient time for booking of labour induction (or elective Caesarean) in the 'Planned delivery' group.*

† NICE guidance compliant

WILL Flow Diagram V6.0 Apr 2022

Appendix C: Schedule of assessments

Trial participant schedule of events and summary of assessments

VISIT	Antenatal care at < 36^{+0} weeks' gestation	36^{+0} - 36^{+6} weeks' gestation	37^{+0} - 37^{+6} weeks' gestation	38^{+0} weeks' gestation to delivery	Randomisation to delivery	Postnatal (1) delivery to hospital discharge	Postnatal (2) hospital discharge to 6 weeks postpartum†
**Screening***	x	x	x				
**Eligibility check**		x	x				
**Valid informed consent**		x	x				
**Baseline data collection**		X (all consented)	X (all consented)				
**Randomisation‡**			x				
**Weekly contact until birth**				X (all randomised)			
**Outcome data collection**							
Maternal and fetal surveillance					X (all randomised)		
Maternal & neonatal outcomes prior hospital discharge						X (all consented)	
Maternal satisfaction outcome						X (all randomised)	
Maternal & neonatal outcomes that occur after hospital discharge							X (all randomised)
**SAEs** (as defined in Section 9.1)			X(all randomised)	X (all randomised)		X(all randomised)	X(all randomised)

** This will occur over the course of pregnancy. Women will be given a PIS to consider and discuss with their families based on potential eligibility related to chronic or gestational hypertension.*
† 'Poor maternal outcome' will be measured to 6 weeks after birth for the mother, and neonatal unit admission for ≥4 hours will be measured to 28 days for the baby.
‡ This can occur over the telephone if the woman had been screened and consented at 36^{+0-6} weeks' gestation during a face-to-face visit, and the woman is confirmed to remain well, without an indication for delivery (see Section 6.2).
¶ Collected via text messaging or online through Textlocal, or post or telephone, if necessary.

Appendix D: ‘Stop-go’ criteria

‘Stop-go’ criteria to be used in the internal pilot trial (N, % women or median):

	Progression criteria		
	Green (go)	Amber (assess and adapt)	Red (stop)
**Randomised of those consented**	≥80%	50-79%	<50%
**Recruitment** rate relative to overall target for 20 pilot sites*	≥60%	30-59%	<30%
**Randomised & delivered at <38+0 weeks** (of those consented)	<15%	15-50%	>50%
**Median between-group difference in gestational age at birth†**	≥7 days	4-6 days	0-3 days
**Action to be taken based on type and number of criteria met**	Proceed with protocol unchanged if ALL criteria are met	Adapt protocol if ONE/MORE criteria are met	Project not feasible and trial will be ended if TWO/MORE criteria are met

**Allowing for a delay in recruitment following site opening we will look for an average recruitment target of 110 women from 20 sites in 8 months.*
†Only the DMC reviewed this criterion, and reported to the TSC which of green, amber, or red criteria were met.

Appendix E: Data manipulations

The Trial Statistician will derive all responses from the raw data recorded in the database.

When a specific set of questions have been grouped and a ‘confirmatory question’ exists and asks to confirm that all the items in the group have been considered and only those ticked apply and those not ticked have not occurred, the Trial Statistician should check the responses on the ‘confirmatory question’ and on each item of this group and follow the guideline set out here:

Response to the confirmatory question	Response to each item in the set of questions in the database	Derived response for each item in the set of questions
YES	The responses to all items in the corresponding set will not change	
NO	YES	YES
	NO	MISSING
MISSING	YES	YES
	NO	MISSING

The following ‘confirmatory questions’ exist:

- Section 3 on the Screening, Consent and Key Baseline Variables Form confirming the contraindication(s) to either one of the trial arms,
- Section 4 on the Screening, Consent and Key Baseline Variables Form confirming the reason(s) why the woman does not wish to be re-contacted,
- Section 5 on the Screening, Consent and Key Baseline Variables Form confirming the reason(s) why the woman did not consent,
- Section 4 on the Additional Baseline Information Form confirming the indications of how Gestational Diabetes Mellitus (GDM) was managed,
- Section 4 on the Additional Baseline Information Form confirming the indications of nicotine product use,
- Section 3 on the Delivery Form confirming the methods used when the onset of labour was induced,
- Section 3 on the Delivery Form confirming the indications when the woman had induced or no labour (Caesarean before labour),
- Section 3 on the Delivery Form confirming what led to the indication when the woman had Caesarean in labour or operative vaginal mode of birth,
- Section 4 on the Maternal Outcome & PostPartum Management Form confirming the Maternal symptoms or signs of pre-eclampsia,
- Section 5 on the Maternal Outcome & PostPartum Management Form confirming the Abnormal laboratory tests,
- Section 6 on the Maternal Outcome & PostPartum Management Form confirming the Maternal complications,
- Section 6 on the Maternal Outcome & PostPartum Management Form confirming the basis of the definition of Placental abruption,
- Section 2 on the Neonatal Form confirming the principal indication for 1st admission,
- Section 2 on the Neonatal Form confirming the principal indication for 2nd admission,
- Section 2 on the Neonatal Form confirming the principal indication for 3rd admission,
- Section 2 on the Neonatal Form confirming the principal indication for 4th admission,
- Section 2 on the Neonatal Form confirming the principal indication for 5th admission, and,
- Section 2 on the Trial Withdrawal Form confirming the reason for withdrawal.

Outcome measures

- **Maternal co-primary outcome, defined as the composite of poor maternal outcome until primary hospital discharge home or 28 days after birth (whichever is earlier)**

Reported in Sections 5 and 6 on the Maternal Outcome and Postpartum Management Form, by answering the following questions:

6.2 Severe hypertension (if occurred after randomisation or after birth)

6.1 Maternal death

6.4 Glasgow Coma Score (GCS)<13

6.5 Stroke

6.6 Transient ischaemic attack

6.7 Eclampsia

6.8 Blindness

6.3 Uncontrolled hypertension

6.9 Inotropic support

6.10 Pulmonary oedema

6.11 Respiratory failure not due to Caesarean delivery

6.12 SpO2 (oxygen saturation) < 90%

6.13 Myocardial ischaemia or infarction

6.14 Hepatic dysfunction

6.15 Hepatic haematoma

6.16 Hepatic rupture

6.17 Acute kidney injury or dialysis

5.1 Platelet count $<50x10^9/L$

6.18 Transfusion

6.19 Placental abruption

Based on the above individually-defined binary outcomes for each component of maternal morbidities, a composite of poor maternal outcome will be defined as a binary outcome which can be coded as YES if and only if at least one of the above individually defined binary outcomes is answered YES. If all are recorded as NO, then the composite outcome will be coded as NO. If any are missing and at least one is YES, then the outcome will be coded as YES. If any are missing and the remainder are NO, then the outcome will be coded as missing.

Each of the data items listed above includes the timing (before or after birth) of the specific maternal complications or abnormal laboratory tests and the date of first occurrence or diagnosis, except for placental abruption which could only occur before birth. Each outcome will be summarised according to whether it occurred before or after birth, along with summary statistics for the gestational or postnatal age.

All outcomes will be included in the analysis whether or not they were adjudicated by the PI. A footnote will be added to the primary outcome table to indicate whether any of the reported maternal co-primary outcomes were not adjudicated.

- **Neonatal co-primary outcome, defined as neonatal care unit admission for ≥ 4 hours, up to primary hospital discharge home or 28 days of life, whichever is earlier**

Reported on Section 2 on the Neonatal Form by answering the questions:

2.1 Was the baby admitted to a neonatal care unit?

If yes, how many times was the baby admitted?

2.2.1.1 1st admission, date and time admitted to neonatal care

2.2.1.2 1st admission, date and time discharged from neonatal care

2.2.2.1 2nd admission, date and time admitted to neonatal care

2.2.2.2 2nd admission, date and time discharged from neonatal care

2.2.3.1 3rd admission, date and time admitted to neonatal care

2.2.3.2 3rd admission, date and time discharged from neonatal care

2.2.4.1 4th admission, date and time admitted to neonatal care

2.2.4.2 4th admission, date and time discharged from neonatal care

2.2.5.1 5th admission, date and time admitted to neonatal care

2.2.5.2 5th admission, date and time discharged from neonatal care

And on the Delivery Form by answering the question:

3.8 Status of baby at birth

For each admission, the duration of the admission will be calculated as (in hours):

(date discharged from neonatal care - date admitted to neonatal care) *24 + (hours discharged from neonatal care - hours admitted to neonatal care) + (minutes discharged from neonatal care - minutes admitted to neonatal care) /60

The neonatal co-primary outcome will be coded as YES if the answer to the question 2.1 “was the baby admitted to a neonatal care unit?” is a YES and at least one of the time durations calculated above is ≥4 hours. Unless at least one duration is ≥ 4 hours, if one of the time durations is missing, then the neonatal co-primary outcome should also be coded as missing.

The neonatal co-primary outcome will be coded as NO if the answer to the question 2.1 “was the baby admitted to a neonatal care unit?” is a NO, or if the answer to the question 2.1 “was the baby admitted to a neonatal care unit?” is a YES, but all time durations are <4 hours, or if the status of baby at birth is STILLBORN.

- **Key maternal outcome: Caesarean delivery**

Reported on the Delivery Form by answering the question

3.3 Mode of birth

A binary variable will be defined as YES if the mode of delivery is “Caesarean before labour” or “Caesarean in labour” and as NO if the mode of delivery is “spontaneous vaginal” or “instrumental vaginal”.

- **Instrumental vaginal delivery or Caesarean delivery**

Reported on the Delivery Form by answering the question

3.3 Mode of birth

A binary variable will be defined as YES if the mode of delivery is “Caesarean before labour” or “Caesarean in labour” or “Operative vaginal”, and as NO if the mode of delivery is “spontaneous vaginal”.

- **Infection of the stitches from the Caesarean wound, episiotomy or vaginal tear**

Reported on the Six-week Postpartum Form by answering the questions

3. Did you have stitches after the birth of your baby? These could be a result of a Caesarean delivery, episiotomy, or vaginal tear?

3a Since discharge from hospital after the birth of your baby, until the time when your baby was six weeks of age, have you had an infection of your stitches?

This outcome will be derived for those women who had stiches.

If question 3 is NO then the derived variable will be coded as NOT APPLICABLE.

If question 3 is answered YES and question 3a is answered YES then the derived variable will be coded as YES.

If question 3 is answered YES and question 3a is answered NO then the derived variable will be coded as NO.

If question 3 is answered YES or missing and question 3a is answered missing then the derived variable will be coded as missing.

- **Individual components of maternal co-primary outcome, up to discharge or 28 days postpartum (whichever is earlier)**

All the individual components of maternal co-primary outcome listed above will be analysed as secondary outcomes separately. The following components will be combined into one composite secondary outcome:

- Hepatic haematoma or rupture

Reported on the Maternal Outcome and Postpartum Management Form by answering the questions

6.15 Hepatic haematoma

6.16 Hepatic rupture

A binary variable will be defined as YES if at least one of the answers to the above two questions is YES, and NO if both are recorded as NO. All other responses will be regarded as missing.

- **Composite of poor maternal outcome (assessed as one or more of the components [presented descriptively] of the maternal co-primary outcome) measured at six weeks postpartum**

The search for maternal complications or abnormal laboratory tests for the maternal co-primary outcome is extended to six weeks postpartum. So, in addition to the corresponding data items used for defining the maternal co-primary outcome reported in Sections 5 and 6 on the Maternal Outcome and Postpartum Management Form, we will also use the data items reported on the Six-week postpartum form or SAE form.

Each individual maternal morbidity will be updated according to the following table:

**Randomisation to primary discharge home***	**Primary discharge home* to 6 weeks postpartum**	**Randomisation to 6 weeks postpartum**
YES	YES	YES
YES	NO	YES
YES	MISSING	YES
NO	YES	YES
NO	NO	NO
NO	MISSING	NO
MISSING	YES	YES
MISSING	NO	MISSING
MISSING	MISSING	MISSING

*or 28d postpartum, whichever is earlier

Note: *The existing outcome will be updated only if there is positive extended question, with the exception of Abruption, Platelet count, Uncontrolled hypertension, and GCS, none of which are assessed at six weeks postpartum.*

Severe hypertension:

In addition to data item 6.2 reported on the Maternal Outcome and Postpartum Management Form, the following data item reported on the Six-week postpartum form will also be used:

1. Since your discharge from hospital after the birth of your baby, until the time when your baby was six weeks of age, have you had very high blood pressure that required you to return to your GP practice, walk-in health centre, or hospital urgently?

Maternal death:

In addition to data item 6.1 reported on the Maternal Outcome and Postpartum Management Form, the following data items reported on the SAE form will also be used:

5.1 What was the nature of the expeditable SAE?

If death, what was the date of death?

A binary outcome for maternal death measured at six weeks postpartum will be defined using the randomisation to primary discharge home or 28d postpartum, whichever is earlier binary outcome for maternal death defined for constructing the maternal co-primary outcome and the binary variable will be updated based on the extra information provided in the answer to the above questions. For example, if the randomisation to primary discharge home or 28d postpartum, whichever is earlier binary outcome for maternal death is NO or missing but the answers to the above questions can be used to ascertain a death event after the date of randomisation but before six weeks postpartum then the binary outcome for maternal death measured at six weeks postpartum will be updated as YES.

For each of the additional maternal morbidities listed below, the following data item reported on the Six-week postpartum form should be answered YES in the first instance, in addition to answering the extra data items listed individually in each case:

2. Since your discharge from hospital after the birth of your baby, until the time when your baby was six weeks of age, have you had to stay in hospital for at least one night with a health problem?

Stroke or TIA:

These individual morbidities will be combined when measured for the six-week postpartum outcome. Only “stroke” is included in the questionnaire because the lay term for TIA is "mini-stroke" and therefore the six-week outcome includes both stroke and TIA.

In addition to data items 6.5 and 6.6 reported on the Maternal Outcome and Postpartum Management Form, the following data item reported on the Six-week postpartum form will also be used:

2a Stroke?

Eclampsia:

In addition to data item 6.7 reported on the Maternal Outcome and Postpartum Management Form, the following data items reported on the Six-week Postpartum Form will also be used:

2a Seizure(s)?

Blindness:

In addition to data item 6.8 reported on the Maternal Outcome and Postpartum Management Form, the following data item reported on the Six-week Postpartum Form will also be used:

2a Blindness?

Inotropic support:

In addition to data item 6.9 reported on the Maternal Outcome and Postpartum Management Form, the following data item reported on the Six-week Postpartum Form will also be used:

2a Intensive care unit admission where they gave you medication to keep your blood pressure from being too low?

Pulmonary oedema:

In addition to data item 6.10 reported on the Maternal Outcome and Postpartum Management Form, the following data item reported on the Six-week Postpartum Form will also be used:

2a Water in your lungs that required you to have oxygen, assistance breathing, or specific medication?

Respiratory failure not due to Caesarean delivery:

In addition to data item 6.11 reported on the Maternal Outcome and Postpartum Management Form, the following data item reported on the Six-week Postpartum Form will also be used:

2a Other shortness of breath requiring oxygen or other assistance breathing?

SpO_2 (oxygen saturation) < 90%:

In addition to data item 6.12 reported on the Maternal Outcome and Postpartum Management Form, the following data item reported on the Six-week Postpartum Form will also be used:

2a Other shortness of breath requiring oxygen or other assistance breathing?

Myocardial ischaemia or infarction:

In addition to data item 6.13 reported on the Maternal Outcome and Postpartum Management Form, the following data item reported on the Six-week Postpartum Form will also be used:

2a Heart attack or angina?

Hepatic dysfunction:

In addition to data item 6.14 reported on the Maternal Outcome and Postpartum Management Form, the following data item reported on the Six-week Postpartum Form will also be used:

2a Liver failure?

Hepatic haematoma or **Hepatic rupture:**

In addition to data items 6.15 and 6.16 reported on the Maternal Outcome and Postpartum Management Form, the following data item reported on the Six-week Postpartum Form will also be used:

2a A blood clot on the liver or liver rupture?

Acute kidney injury or dialysis:

In addition to data item 6.17 reported on the Maternal Outcome and Postpartum Management Form, the following data item reported on the Six-week Postpartum Form will also be used:

2a Kidney injury?

Transfusion:

In addition to data item 6.18 reported on the Maternal Outcome and Postpartum Management Form, the following data item reported on the Six-week Postpartum Form will also be used:

2a Transfusion of any blood products?

The composite of poor maternal outcome until primary hospital discharge home or 28 days after birth (whichever is earlier) will be updated and a new composite of poor maternal outcome measured at six weeks postpartum will be defined as a binary outcome which can be updated as YES if and only if any of the above individually defined binary outcomes was updated as YES.

- **Elevated liver enzymes (aspartate aminotransferase or alanine aminotransferase > 40 IU/L)**

Reported on the Maternal Outcome and Postpartum Management Form by answering the following question:

5.6 Elevated AST or ALT (>40 IU/L)

A binary variable will be defined as YES if the answer to the above question is YES and NO vice versa.

- **Platelet count <$100x10^9$/L**

Reported on the Maternal Outcome and Postpartum Management Form by answering the following questions:

5.1 Platelet count <$50x10^9$/L

5.2 Platelet count 50 to 99x10^9/L

A binary outcome will be defined as YES if at least one of the questions 5.1 or 5.2 is answered YES and as NO if both answers in the questions 5.1 and 5.2 are NO. All other responses will be regarded as missing.

- **Pre-eclampsia**

Reported on the Maternal Outcome and Postpartum Management Form by answering the following questions:

3.1 Did the woman develop proteinuria between consent and birth?*
5.7 Elevated serum creatinine of ≥90 micromole/L*
5.6 Elevated AST or ALT (>40 IU/L)*
6.7 Eclampsia*
6.4 Glasgow Coma Score (GCS) <13*
6.8 Blindness*
6.5 Stroke*
4.3 Clonus*
4.1 Headache*
4.2 Visual scotomata*
5.1 Platelet count <50x10^9/L*
5.2 Platelet count 50 to 99x10^9/L*
5.3 Platelet count 100 to 149x10^9/L*
5.4 Disseminated intravascular coagulation*
5.5 Haemolysis*
and on the Maternal & Fetal Surveillance and Antenatal Care Form by answering the following question:

3.2 Did the woman have a fetal ultrasound?

3.2.1 If yes, was there evidence of persistently absent or reduced end-diastolic flow by umbilical artery Doppler? (Note this question will be regarded as no if 'did the woman have a fetal ultrasound' is no)*

and on the Delivery Form by answering the following question:

3.8 Status of baby at birth

3.7 Birthweight (For birthweight centile we also need the questions: 3.4 Baby's date of birth & 3.6 Sex of baby reported on the Delivery Form and the question: 2.6 Please indicate Estimated Date of Delivery (EDD) reported on the Screening, Consent and Key Baseline Information Form.)

A binary outcome for Pre-eclampsia by ISSHP 2018 criteria will be defined as YES if any of the questions above indicated by (*) are yes, or the status of baby at birth is STILLBORN, or birthweight<10th centile using the INTERGROWTH 21st standards[20]. The outcome will be defined as NO if all of questions indicated by (*) are NO, the status of baby at birth is ALIVE, and birthweight is ≥10th centile. All other responses will be regarded as missing.

- **Postpartum haemorrhage (PPH)**

Reported on the Maternal Outcome and Postpartum Management Form by answering the following question:

6.20 Postpartum haemorrhage (PPH)

A binary variable will be defined as YES if the answer to the above question is YES and NO vice versa.

- **Sepsis (known or suspected maternal infection with two or more of Quick Sequential Organ Failure Assessment (qSOFA) criteria: respiratory rate ≥22/min, altered mentation, or systolic BP ≤100mmHg)**

Reported on the Maternal Outcome and Postpartum Management Form by answering the following question:

6.22 Known or suspected infection

Did the woman have a respiratory rate of 22/min or more?

Did the woman have altered mentation?

Did the woman have a sBP of 100mmHg or lower?

The outcome will be derived according to the following table:

**6.22 Known or suspected infection**	+	**Did the woman have a respiratory rate of 22/min or more? Did the woman have altered mentation? Did the woman have a systolic BP of 100mmHg or lower?**	=	**Sepsis (*as is defined above*)**
YES	+	2 YES (3rd answer could be anything)	=	YES
YES	+	2 MISSING (3rd answer could be anything)	=	MISSING
YES	+	1 YES+1 NO+MISSING	=	MISSING
YES	+	2 NO (3rd answer could be anything)	=	NO
NO*	+	Regardless of the answers here	=	NO
MISSING**	+	Regardless of the answers here	=	MISSING

***** *If a known or suspected infection is not selected and the confirmatory question is YES.*

****** *If a known or suspected infection is not selected and the confirmatory question is MISSING or NO.*

- **Intensive Therapy Unit admission (ITU)**

Reported on the Maternal Outcome and Postpartum Management Form by answering the following question:

6.21 Intensive therapy unit ITU) admission

A binary variable will be defined as YES if the answer to the above question is YES and NO vice versa.

- **Co-interventions: antihypertensive therapy (antepartum)**

Reported on the Maternal and Fetal Surveillance and Antenatal Care Form by answering the following question:

4.3 Did the woman take any antihypertensive medication? (from randomisation until admission for birth)

and on the Delivery Form by answering the following question:

2.3 Did the woman take any antihypertensive medication? (after admission and before the birth)

The outcome will be derived according to the following table:

**4.3 Did the woman take any antihypertensive medication? (from randomisation until admission for birth)**	+	**2.3 Did the woman take any antihypertensive medication? (after admission and before the birth)***	=	**Co-interventions: antihypertensive therapy (antepartum)**
YES	+	Regardless of the answer here	=	YES
Regardless of the answer here	+	YES	=	YES
NO	+	NO	=	NO
NO	+	MISSING	=	MISSING
MISSING	+	NO	=	MISSING
MISSING	+	MISSING	=	MISSING

**For women consented (if not randomised) or randomised after admission for birth, the timing of antihypertensive medication will be queried and YES will be accepted only if the antihypertensive medication was taken after consent date for women not randomised or randomisation date.*

Type of antihypertensive therapy [i.e. Labetalol, Methyldopa, Nifedipine (either long-acting or modified-release), and other] will be presented descriptively by combining the answers in the question:

If yes, please specify any antihypertensive medication

reported on the Maternal and Fetal Surveillance and Antenatal Care Form and the Delivery Form alike.

Each individual antihypertensive medication outcome will be derived according to the following table:

Individual antihypertensive medication reported on the Maternal and Fetal Surveillance and Antenatal Care Form	+	Individual antihypertensive medication reported on the Delivery Form	=	Individual antihypertensive medication (antepartum)
YES	+	Regardless of the answer here	=	YES
Regardless of the answer here	+	YES	=	YES
NO	+	NO	=	NO
NO	+	MISSING	=	MISSING
MISSING	+	NO	=	MISSING
MISSING	+	MISSING	=	MISSING

- **Co-interventions: antihypertensive therapy (postpartum)**

Reported on the Maternal Outcome and Postpartum Management Form by answering the following question:

7.1 Did the woman receive any antihypertensive medication? (after birth & before primary hospital discharge or 28 days postpartum)

A binary outcome will be defined as YES if the answer to the above question is YES and NO vice versa.

- **Co-interventions: antihypertensive therapy (both antepartum and postpartum)**
    - Co-interventions: antihypertensive therapy (antepartum)
    - Co-interventions: antihypertensive therapy (postpartum)

Co-interventions: antihypertensive therapy (both antepartum and postpartum) outcome will be derived according to the following table:

Co-interventions: antihypertensive therapy (antepartum)	+	Co-interventions: antihypertensive therapy (postpartum)	=	Co-interventions: antihypertensive therapy (both antepartum and postpartum)
YES	+	Regardless of the answer here	=	YES
Regardless of the answer here	+	YES	=	YES
NO	+	NO	=	NO
NO	+	MISSING	=	MISSING
MISSING	+	NO	=	MISSING
MISSING	+	MISSING	=	MISSING

- **Co-interventions: Magnesium Sulphate**

Reported on the Delivery Form by answering the following question:

2.2 Did the woman receive magnesium sulphate? (after admission and before the birth)

and on the Maternal Outcome and Postpartum Management Form by answering the following question:

7.2 Did the woman receive magnesium sulphate? (after birth & before primary hospital discharge or 28 days postpartum)

Co-interventions: Magnesium Sulphate outcome will be derived according to the following table:

2.2 Did the woman receive magnesium sulphate? (after admission and before the birth)*	+	7.2 Did the woman receive magnesium sulphate? (after birth & before primary hospital discharge or 28 days postpartum)	=	Co-interventions: Magnesium Sulphate
YES	+	Regardless of the answer here	=	YES

Regardless of the answer here	+	YES	=	YES
NO	+	NO	=	NO
NO	+	MISSING	=	MISSING
MISSING	+	NO	=	MISSING
MISSING	+	MISSING	=	MISSING

**For women consented (if not randomised) or randomised after admission for birth, the timing of magnesium sulphate will be queried and YES will be accepted only if the magnesium sulphate was taken after consent date for women not randomised or randomisation date.*

- **Co-interventions: bed rest at home**

Reported on the Maternal and Fetal Surveillance and Antenatal Care Form by answering the following question:

4.1 Was the woman put on bed rest at home? (from randomisation until admission for birth)

A binary outcome will be defined as YES if the answer to the above question is YES and NO vice versa.

- **Co-interventions: use of home BP monitoring**

Reported on the Maternal and Fetal Surveillance and Antenatal Care Form by answering the following question:

4.2 Did the woman use home BP monitoring? (from randomisation until admission for birth)

A binary outcome will be defined as YES if the answer to the above question is YES and NO vice versa.

- **Co-interventions: maternal blood or urine testing prior to delivery admission**

Reported on the Maternal and Fetal Surveillance and Antenatal Care Form by answering the following questions:

2.1 Did the woman have 'pre-eclampsia' blood and/or urine tests at hospital?

If YES, how many times?

A binary outcome will be defined as YES if the answer to the above question is YES and NO vice versa.

In addition, for each woman, the number of such testing performed will be defined and presented descriptively using the information provided in the answer to "If YES, how many times?". If no testing were performed, the variable will be coded as zero.

- **Co-interventions: outpatient visits (in office/clinic)**

Reported on the Maternal and Fetal Surveillance and Antenatal Care Form by answering the following questions:

2.2 Did the woman have any outpatient visits?

If yes, how many times?

A binary outcome will be defined as YES if the answer to the above question is YES and NO vice versa.

In addition, for each woman, the number of outpatient visits will be defined and presented descriptively using the information provided in the answer to "If YES, how many times?". If no outpatient visits, the variable will be coded as zero.

- **Co-interventions: outpatient visits (in her home)**

Reported on the Maternal and Fetal Surveillance and Antenatal Care Form by answering the following questions:

2.4 Was the woman seen in her home by a community midwife?

If yes, how many times?

A binary outcome will be defined as YES if the answer to the above question is YES and NO vice versa.

In addition, for each woman the number of outpatient visits will be defined and presented descriptively using the information provided in the answer to "If YES, how many times?". If no outpatient visits, the variable will be coded as zero.

- **Co-interventions: medical, day or maternity assessment unit visits**

Reported on the Maternal and Fetal Surveillance and Antenatal Care Form by answering the following questions:

2.3 Was the woman seen in a medical, day, or maternity assessment unit?

If yes, how many times?

A binary outcome will be defined as YES if the answer to the above question is YES and NO vice versa.

In addition, for each woman the number of the woman seen in a medical, day or maternity assessment unit will be defined and presented descriptively using the information provided in the answer to "If YES, how many times?". If no visits, the variable will be coded as zero.

- **Co-interventions: acute area visits (such as Accident and Emergency)**

Reported on the Maternal and Fetal Surveillance and Antenatal Care Form by answering the following questions:

2.5 Was the woman seen in an acute area?

If yes, how many times?

A binary outcome will be defined as YES if the answer to the above question is YES and NO vice versa.

In addition, for each woman the number of acute area visits will be defined and presented descriptively using the information provided in the answer to "If YES, how many times?". If no acute area visits, the variable will be coded as zero.

- **Co-interventions: number of antenatal admission days prior to birth**

Reported on the Maternal and Fetal Surveillance and Antenatal Care Form by answering the following questions:

2.6 Was the woman admitted to and discharged from hospital for at least ONE night before the admission during which she gave birth (or before birth if she did not give birth in hospital)? If yes, please enter how many times she was admitted.

If yes, date of admission and date of discharge for each admission.

For each admission, the number of antenatal admission days will be calculated as:

date of discharge - date of admission

For each woman, the total number of antenatal admission days will be calculated as the total number of days for each admission. A score of zero will be given to a woman who was not admitted to hospital before her admission for birth.

- **Co-interventions: fetal cardiotocography**

Reported on the Maternal and Fetal Surveillance and Antenatal Care Form by answering the following question:

3.1 Did the woman have a fetal cardiotograph (CTG)?

A binary outcome will be defined as YES if the answer to the above question is YES and NO vice versa.

- **Co-interventions: fetal ultrasound**

Reported on the Maternal and Fetal Surveillance and Antenatal Care Form by answering the following question:

3.2 Did the woman have a fetal ultrasound?

A binary outcome will be defined as YES if the answer to the above question is YES and NO vice versa.

- **Clinical indications for birth in both arms**

Reported on the Delivery Form by answering the following question:

3.2.3 If induced or no labour (Caesarean before labour), what led to the decision to deliver the woman?

Numbers and percentages of the following options will be presented

    - Dictated by study protocol for allocated group

- Maternal hypertension not controlled
- Maternal pre-eclampsia
- Other maternal reason(s) (Please specify below, e.g., PROM.)
- Poor fetal growth
- Abnormal fetal heart rate or pattern
- Abnormal umbilical artery Doppler
- Other fetal reason(s) (Please specify below.)
- Busy hospital induction or theatre schedules
- Woman's preference as only reason if timing not consistent with allocated group
- Clinician's preference as only reason if timing not consistent with allocated group
- Other (Please specify below.)

Additionally, numbers and percentages will be presented for the maternal reasons only and the fetal reason only, which will be grouped based on to the following table:

Indications	Maternal or fetal or both or N/A
Maternal hypertension not controlled	Maternal
Maternal pre-eclampsia	Maternal
Other maternal reason(s)	Maternal
Abnormal fetal heart rate or pattern	Fetal
Abnormal umbilical artery Doppler	Fetal
Other fetal reason(s)	Fetal

- **Maternal satisfaction assessed at hospital discharge or 28 days postpartum (whichever is earlier), as measured by the Childbirth Experience Questionnaire**

Women's satisfaction with the interventions and trial participation will be evaluated according to the Childbirth Experience Questionnaire (CEQ 2.0)[21], a 22-item self-administered questionnaire that has been validated in the UK. The response format was a 4-point Likert scale ranging from 1 (Totally agree), 2 (Mostly agree), 3 (Mostly disagree) to 4 (Totally disagree). The VAS-scales scores were transformed to categorical values, 0-40 = 1, 41-60 = 2, 61-80 = 3 and 81-100 = 4, which in a scale to 10, this corresponds to 0-4 = 1, 5-6 = 2, 7-8 = 3 and 9-10 = 4. Ratings of positively worded statements and the pain items were reversed so that higher scores reflect more positive scoring. Higher scales reflect greater satisfaction, as do higher domain scores that cover the following 4 scales:

- **Own capacity (8 items)**
  - <u>Labour and birth went as I had expected</u> [item will be reversed and scored as: 4 (Totally agree), 3 (Mostly agree), 2 (Mostly disagree) and 1 (Totally disagree)]
  - <u>I felt strong during labour and birth</u> [item will be reversed and scored as: 4 (Totally agree), 3 (Mostly agree), 2 (Mostly disagree) and 1 (Totally disagree)]
  - <u>I felt capable during labour and birth</u> [item will be reversed and scored as: 4 (Totally agree), 3 (Mostly agree), 2 (Mostly disagree) and 1 (Totally disagree)]
  - <u>I was tired during labour and birth</u> [item will **not** be reversed, it will be scored as: 1 (Totally agree), 2 (Mostly agree), 3 (Mostly disagree) and 4 (Totally disagree)]
  - <u>I felt happy during labour and birth</u> [item will be reversed and scored as: 4 (Totally agree), 3 (Mostly agree), 2 (Mostly disagree) and 1 (Totally disagree)]
  - <u>I felt that I handled the situation well</u> [item will be reversed and scored as: 4 (Totally agree), 3 (Mostly agree), 2 (Mostly disagree) and 1 (Totally disagree)]

- As a whole, how painful did you feel childbirth was? [item will be reversed and scored as: 0-4 = 4, 5-6 = 3, 7-8 = 2 and 9-10 = 1]

- As a whole, how much control did you feel you had during childbirth? [item will **not** be reversed, it will be scored as: 0-4 = 1, 5-6 = 2, 7-8 = 3 and 9-10 = 4]

- **Professional support (5 items)**
  - My midwife devoted enough time to me [item will be reversed and scored as: 4 (Totally agree), 3 (Mostly agree), 2 (Mostly disagree) and 1 (Totally disagree)]

  - My midwife devoted enough time to my partner [item will be reversed and scored as: 4 (Totally agree), 3 (Mostly agree), 2 (Mostly disagree) and 1 (Totally disagree)]

- My midwife kept me informed about what was happening during labour and birth [item will be reversed and scored as:
  4 (Totally agree), 3 (Mostly agree), 2 (Mostly disagree) and 1 (Totally disagree)]

  - My midwife understood my needs [item will be reversed and scored as: 4 (Totally agree), 3 (Mostly agree), 2 (Mostly disagree) and 1 (Totally disagree)]

  - I felt very well cared for by my midwife [item will be reversed and scored as: 4 (Totally agree), 3 (Mostly agree), 2 (Mostly disagree) and 1 (Totally disagree)]

- **Perceived safety (6 items)**
  - I felt scared during labour and birth [item will **not** be reversed, it will be scored as: 1 (Totally agree), 2 (Mostly agree), 3 (Mostly disagree) and 4 (Totally disagree)]

  - I have many positive memories from childbirth [item will be reversed and scored as: 4 (Totally agree), 3 (Mostly agree), 2 (Mostly disagree) and 1 (Totally disagree)]

  - I have many negative memories from childbirth [item will **not** be reversed, it will be scored as: 1 (Totally agree), 2 (Mostly agree), 3 (Mostly disagree) and 4 (Totally disagree)]

  - Some of my memories from childbirth make me feel depressed [item will **not** be reversed, it will be scored as: 1 (Totally agree), 2 (Mostly agree), 3 (Mostly disagree) and 4 (Totally disagree)]

  - My impression of the team's medical skills made me feel secure [item will be reversed and scored as: 4 (Totally agree), 3 (Mostly agree), 2 (Mostly disagree) and 1 (Totally disagree)]

  - As a whole, how secure did you feel during childbirth? [item will **not** be reversed, it will be scored as: 0-4 = 1, 5-6 = 2, 7-8 = 3 and 9-10 = 4]

- **Participation (3 items)**

  - I felt I could have a say whether I could be up and about or lie down [item will be reversed and scored as: 4 (Totally agree), 3 (Mostly agree), 2 (Mostly disagree) and 1 (Totally disagree)]

  - I felt I could have a say in deciding my birthing position [item will be reversed and scored as: 4 (Totally agree), 3 (Mostly agree), 2 (Mostly disagree) and 1 (Totally disagree)]

  - I felt I could have a say in the choice of pain relief [item will be reversed and scored as: 4 (Totally agree), 3 (Mostly agree), 2 (Mostly disagree) and 1 (Totally disagree)]

Where there are missing items, the half-scale method will be used so that when the respondent has answered at least half of the items in the scale the sum of the scores will be divided by the number of answered items. After calculating the CEQ subscale scores, the total CEQ score will be produced as an average of the 4 individual subscale scores[22]. For women who had an elective Caesarean, the Participation subscale score will not be calculated and their total score will be calculated by averaging the remainder three subscale scores (i.e. Own capacity, Professional support and Perceived safety). For the primary statistical analysis, all women will be included. However, due to the fact that the CEQ has not been validated for women having elective Caesarean section, the total and the subscale scores will also be presented for the group of women who had an elective Caesarean and for the group of women who did not.

The group of women who had an elective Caesarean will be determined by those women for whom it has been selected "*No labour (Caesarean before labour)*" on the question:

3.2 Onset of labour

reported on the Delivery Form.

- **Admission to hospital either for birth, or within 28 days after birth - Days of admission until birth**

Reported on the Screening, Consent and Key Baseline Information Form by answering the following question:

Date of consent:

On the Reconfirmation of Eligibility & Randomisation Form by answering the following question:

- Date of randomisation:

On the Maternal Outcome and Postpartum Management Form by answering the following questions:

2.1 Was the woman admitted to hospital EITHER for birth, or within 28 days after birth if she gave birth outside the hospital setting?

Date of admission

6.1 Maternal death – Before birth and if occurred, date of death

And on the Delivery Form by answering the following question:

- Baby's date of birth

Days of admission until birth for women consented (if not randomised) or randomised before admission for birth will be derived according to the following table:

**2.1 Was the woman admitted to hospital EITHER for birth, or within 28 days after birth if she gave birth outside the hospital setting?**	+	**6.1 Maternal death – Before birth**	=	**Days of admission until birth***
YES	+	NO	=	Baby's date of birth – Date of admission
YES	+	YES	=	Date of maternal death – Date of admission
YES	+	MISSING	=	Baby's date of birth – Date of admission
NO	+	-	=	0
MISSING	+	-	=	MISSING

* *If any of the dates are missing on these calculations then the outcome will be missing.*

Days of admission until birth for women consented (if not randomised) or randomised after admission for birth will be derived according to the following table:

**2.1 Was the woman admitted to hospital EITHER for birth, or within 28 days after birth if she gave birth outside the hospital setting?**	+	**6.1 Maternal death – Before birth**	=	**Days of admission until birth***
YES	+	NO	=	Baby's date of birth – Date of consent¥ or randomisation date
YES	+	YES	=	Date of maternal death – Date of consent¥ or randomisation date
YES	+	MISSING	=	Baby's date of birth – Date of consent¥ or randomisation date
NO	+	-	=	0
MISSING	+	-	=	MISSING

* *If any of the dates are missing on these calculations then the outcome will be missing.*
¥ *for non-randomised women.*

- **Admission to hospital either for birth, or within 28 days after birth - Days of admission after birth**

Reported on the Maternal Outcome and Postpartum Management Form by answering the following questions:

2.1 Was the woman admitted to hospital EITHER for birth, or within 28 days after birth if she gave birth outside the hospital setting?

Date of admission

7.3 Was the woman discharged HOME after birth?

If yes, please specify the date of woman's first discharge home after birth

If no, was the woman transferred to another hospital?

If yes, was the transfer prior to 28 days after birth?

Date of transfer to that hospital

Date of discharge from that hospital

On the Delivery Form by answering the following question:

- Baby's date of birth

On the SAE Form by answering the following questions:

5.1 What was the nature of the expeditable SAE?

Maternal death

If death, what was the date of death?

For women admitted to hospital for birth the days of admission after birth will be derived according to the following instructions:

- For a woman discharged home after birth:

Days of admission after birth=Date of woman's first discharge home after birth – Baby's date of birth

- For a woman transferred to another hospital and was discharged from that hospital:

Days of admission after birth=Date of discharge from that hospital – Baby's date of birth

- For a woman who died before was discharged home after birth or transferred to another hospital and died before was discharged from that hospital:

Days of admission after birth=Date of maternal death – Baby's date of birth

- For a woman where maternal death was not recorded and woman is still in the hospital she was admitted or the hospital she was transferred if this occurred:

Days of admission after birth=Date of dataset freeze – Baby's date of birth

For women admitted to hospital within 28 days after birth (if she gave birth outside the hospital setting) the days of admission after birth will be derived according to the following instructions:

- For a woman discharged home after admission:

Days of admission after birth=Date of woman's first discharge home after birth - Date of admission

- For a woman transferred to another hospital and was discharged from that hospital:

Days of admission after birth=Date of discharge from that hospital – Date of admission

- For a woman who died before was discharged home after admission; or was transferred to another hospital and died before was discharged from that hospital:

Days of admission after birth=Date of maternal death – Date of admission

- For a woman where maternal death was not recorded and woman is still in the hospital she was admitted or the hospital she was transferred if this occurred:

Days of admission after birth=Date of dataset freeze – Date of admission

- **Neonatal care unit admission for 4 hours or more assessed to 28 days after birth**

The neonatal co-primary outcome will be derived only among babies, whom their status at birth was alive, based on the answer of the following question reported on the Delivery Form:

3.8 Status of baby at birth;

and updated based on the extra information provided in the answer to the following question reported on the Six-week Postpartum Form:

4. Since your baby came home from hospital until he/she was 28 days old, have you taken your baby to Accident & Emergency or has your baby been admitted to hospital?

Neonatal care unit admission for 4 hours or more assessed to 28 days after birth will be updated according to the following table:

**Birth to primary discharge home***	**Primary discharge home* to 6 weeks postpartum**	**Birth to 6 weeks postpartum**
YES	YES	YES
YES	NO	YES
YES	MISSING	YES
NO	YES	YES
NO	NO	NO
NO	MISSING	NO
MISSING	YES	YES
MISSING	NO	MISSING
MISSING	MISSING	MISSING

*or 28d postpartum, whichever is earlier

Note: *The existing outcome will be updated only if there is positive extended question.*

- **Indication for neonatal care unit admission for ≥4 hours as a respiratory problem up to primary hospital discharge home or 28 days of life, whichever is earlier**

Reported on the Neonatal Form by answering the following questions:

2.1 Was the baby admitted to a neonatal care unit?

If yes, how many times was the baby admitted?

2.2.1.3 What was the principal indication for 1st admission? (as the principal category for admission in the BadgerNet summary).

2.2.1.1 1st admission, DATE and TIME admitted to neonatal care

2.2.1.2 1st admission, DATE and TIME discharged from neonatal care

2.2.2.3 What was the principal indication for 2nd admission? (as the principal category for admission in the BadgerNet summary).

2.2.2.1 2nd admission, DATE and TIME admitted to neonatal care

2.2.2.2 2nd admission, DATE and TIME discharged from neonatal care

2.2.3.3 What was the principal indication for 3rd admission? (as the principal category for admission in the BadgerNet summary).

2.2.3.1 3rd admission, DATE and TIME admitted to neonatal care

2.2.3.2 3rd admission, DATE and TIME discharged from neonatal care

2.2.4.3 What was the principal indication for 4th admission? (as the principal category for admission in the BadgerNet summary).

2.2.4.1 4th admission, DATE and TIME admitted to neonatal care

2.2.4.2 4th admission, DATE and TIME discharged from neonatal care

2.2.5.3 What was the principal indication for 5th admission? (as the principal category for admission in the BadgerNet summary).

2.2.5.1 5th admission, DATE and TIME admitted to neonatal care

2.2.5.2 5th admission, DATE and TIME discharged from neonatal care

Indication for neonatal care unit admission for ≥4 hours as a respiratory problem up to primary hospital discharge home or 28 days of life, whichever is earlier will be derived according to the following table:**2.1**	+	**If yes, how many times was the baby admitted?**	+	**Duration in hours of 1st or 2nd or 3rd or 4th or 5th admission (as is calculated in the neonatal co-primary outcome)**	+	**What was the principal indication for 1st or 2nd or 3rd or 4th or 5th admission? (as the principal category for admission in the BadgerNet summary).**	=	**Indication for neonatal care unit admission for ≥4 hours as a respiratory problem up to primary hospital discharge home or 28 days of life, whichever is earlier**

**Was the baby admitted to a neonatal care unit?**								
YES	+	1 or 2 or 3 or 4 or 5	+	At least one duration ≥ 4 hours	+	For the duration(s) ≥ 4 hours the indication is Respiratory disease	=	YES
YES	+	1 or 2 or 3 or 4 or 5	+	All the durations < 4 hours	+	N/A	=	NO
YES	+	1 or 2 or 3 or 4 or 5	+	At least one duration ≥ 4 hours	+	For the duration(s) ≥ 4 hours the indication is **NOT** Respiratory disease	=	NO
YES	+	1 or 2 or 3 or 4 or 5	+	At least one duration is missing and the remainder < 4 hours	+	-	=	MISSING
YES	+	1 or 2 or 3 or 4 or 5	+	At least one duration ≥ 4 hours	+	For the duration(s) ≥ 4 hours the indication is MISSING	=	MISSING
NO	+	-	+	-	+	-	=	NO
MISSING	+	-	+	-	+	-	=	MISSING

Note: *This outcome will be derived only among babies, whom their status at birth was alive, based on the answer of the following question reported on the Delivery Form:*

3.8 Status of baby at birth

- **Other indications for neonatal care unit admission ≥4 hours**

Indications for admission, as identified by the clinical team by the principle indication for admission on the BadgerNet and other indications, as identified clinically (e.g., 5-min Apgar score <7, birthweight <10th centile, birthweight >90th centile, sepsis work-up, hyper- or hypo-glycaemia, or other) will be presented descriptively for babies achieved the co-primary outcome (admission≥4 hours) and if they have 2 or more admissions ≥4 hours then the indications for each admission≥4 hours will be presented.

Reported on the Neonatal Form by answering the following questions:

2.2.1.3 What was the principal indication for 1st admission? (as the principal category for admission in the BadgerNet summary)

2.2.2.3 What was the principal indication for 2nd admission? (as the principal category for admission in the BadgerNet summary)

2.2.3.3 What was the principal indication for 3rd admission? (as the principal category for admission in the BadgerNet summary)

2.2.4.3 What was the principal indication for 4th admission? (as the principal category for admission in the BadgerNet summary)

2.2.5.3 What was the principal indication for 5th admission? (as the principal category for admission in the BadgerNet summary)

Note: *This outcome will be derived only among babies, whom their status at birth was alive, based on the answer of the following question reported on the Delivery Form:*

3.8 Status of baby at birth.

- **Respiratory morbidity**

Reported on the Neonatal Form by answering the following questions:

2.1 Was the baby admitted to a neonatal care unit?

2.3 Was supplemental oxygen given in the first 24 hours of life beyond the initial resuscitation period?

2.4 Was positive pressure ventilation (PPV) used in the first 72 hours of life beyond the initial resuscitation period?

Respiratory morbidity will be derived according to the following table:

**2.1 Was the baby admitted to a neonatal care unit?**	**+**	**2.3 Was supplemental oxygen given in the first 24 hours of life beyond the initial resuscitation period?**	**+**	**2.4 Was positive pressure ventilation (PPV) used in the first 72 hours of life beyond the initial resuscitation period?**	**=**	**Respiratory morbidity**
YES	+	YES	+	Regardless of the answer here	=	YES
YES	+	Regardless of the answer here	+	YES	=	YES
YES	+	NO	+	NO	=	NO
YES	+	NO	+	MISSING	=	MISSING
YES	+	MISSING	+	NO	=	MISSING
YES	+	MISSING	+	MISSING	=	MISSING
NO	+	-	+	-	=	NO
MISSING	+	-	+	-	=	MISSING

Note: *This outcome will be derived only among babies, whom their status at birth was alive, based on the answer of the following question reported on the Delivery Form:*

3.8 Status of baby at birth.

- **Clinical respiratory problem**

Reported on the Neonatal Form by answering the following question:

2.8 Did the baby have a clinical respiratory problem?

A binary outcome will be defined as YES if the answer to the above question is YES and NO vice versa.

Note: *This outcome will be derived only among babies, whom their status at birth was alive, based on the answer of the following question reported on the Delivery Form:*

3.8 Status of baby at birth.

- **Chest x-ray**

Reported on the Neonatal Form by answering the following question:

2.9 Did the baby have a chest X-ray(s)?

A binary outcome will be defined as YES if the answer to the above question is YES and NO vice versa.

In addition, the number of chest x-rays performed, the number of abnormal chest x-rays and the nature of abnormality will be presented descriptively.

Note: *This outcome will be derived only among babies, whom their status at birth was alive, based on the answer of the following question reported on the Delivery Form:*

3.8 Status of baby at birth.

- **Hypoxic-ischaemic encephalopathy, defined as therapeutic hypothermia for ≥72 hours**

Reported on the Neonatal Form by answering the following question:

2.5 Did the baby have evidence of hypoxic-ischaemic encephalopathy?

A binary outcome will be defined as YES if the answer to the above question is YES and NO vice versa.

Note: *This outcome will be derived only among babies, whom their status at birth was alive, based on the answer of the following question reported on the Delivery Form:*

3.8 Status of baby at birth.

- **Sepsis requiring antibiotics for at least five days, with confirmed blood or cerebrospinal fluid culture**

Reported on the Neonatal Form by answering the following question:

2.6 Did the baby have sepsis?

A binary outcome will be defined as YES if the answer to the above question is YES and NO vice versa.

Note: *This outcome will be derived only among babies, whom their status at birth was alive, based on the answer of the following question reported on the Delivery Form:*

3.8 Status of baby at birth.

- **Major operations**

Reported on the Neonatal Form by answering the following question:

2.7 Did the baby have a major operation?

A binary outcome will be defined as YES if the answer to the above question is YES and NO vice versa.

Note: *This outcome will be derived only among babies, whom their status at birth was alive, based on the answer of the following question reported on the Delivery Form:*

3.8 Status of baby at birth.

- **Birthweight**

Reported on the Delivery Form by the following data items:

3.4 Baby's date of birth

3.6 Sex of baby

3.7 Birthweight

and on the Screening, Consent and Key Baseline Information Form by the following data item:

2.6 Please indicate Estimated Date of Delivery (EDD).

Birth weight will be adjusted for gestational age and sex using the intergrowth standards. Centiles will be produced from this output.[23]

Note: *This outcome will be derived only among babies, whom their status at birth was alive, based on the answer of the following question reported on the Delivery Form:*

3.8 Status of baby at birth.

- **Apgar scores recorded at 1, 5 and 10 minutes**

Reported on the Delivery Form by the following data items:

4.1 Apgar score at 1 minute recorded?

4.2 Apgar score at 5 minutes recorded?

4.3 Apgar score at 10 minutes recorded?

Three continuous variables will be defined, respectively, based on each of the above data items. The score will take a missing value if it was not recorded.

Note: *This outcome will be derived only among babies, whom their status at birth was alive, based on the answer of the following question reported on the Delivery Form:*

3.8 Status of baby at birth.

- **Stillbirth**

Reported on the Delivery Form by answering the following question:

3.8 Status of baby at birth

A binary outcome will be defined as YES if the answer to the above question is "stillborn" and NO if the answer to the above question is "alive".

- **Neonatal death**

Reported on the Neonatal Form by answering the following question:

2.10 Did the baby die prior to discharge from hospital?

If yes, date on which the baby died

On the SAE Form by answering the following question:

5.1 What was the nature of the expeditable SAE? - Neonatal death

If death, what was the date of death?

On the Delivery Form by answering the following question:

3.4 Baby's date of birth

Neonatal death will be derived according to the following table:

On the Screening, Consent and Key Baseline Information Form by answering the following question:

2.6 Please indicate Estimated Date of Delivery (EDD):

And on the Reconfirmation Of Eligibility & Randomisation Form by answering the following question:

3.2 Date of randomisation

**2.10 Did the baby die prior to discharge from hospital?**	+	**3.4 Baby's date of birth**	+	**5.1 What was the nature of the expeditable SAE? - Neonatal death**<br>**If death, what was the date of death?**	=	**Neonatal death**
YES	+	N/A	+	Regardless of the answer here	=	YES
NO or MISSING	+	Date provided	+	YES **and**<br>Date of death-Baby's date of birth≤28 days	=	YES
NO or MISSING	+	Date provided	+	YES **and**<br>Date of death-Baby's date of birth>28 days	=	NO
NO or MISSING	+	MISSING	+	N/A	=	MISSING

Note: *This outcome will be derived only among babies, whom their status at birth was alive, based on the answer of the following question reported on the Delivery Form:*

3.8 Status of baby at birth

- **Breastfeeding established**

Reported on the Neonatal Form by answering the following question:

2.12 Was breastfeeding established at hospital discharge (or 28 days after birth, whichever was earlier)?

A binary outcome will be defined as YES if the answer to the above question is YES and NO vice versa.

Note: *This outcome will be derived only among babies, whom their status at birth was alive, based on the answer of the following question reported on the Delivery Form:*

3.8 Status of baby at birth.

- **Exclusive breastfeeding**

Reported on the Neonatal Form by answering the following questions:

2.12 Was breastfeeding established at hospital discharge (or 28 days after birth, whichever was earlier)?

If yes, was the baby receiving supplementary formula feeds?

Exclusive breastfeeding will be derived according to the following table:

**2.12 Was breastfeeding established at hospital discharge (or 28 days after birth, whichever was earlier)?**	**+**	**If yes, was the baby receiving supplementary formula feeds?**	**=**	**Exclusive breastfeeding**
YES	+	YES	=	NO
YES	+	NO	=	YES
YES	+	MISSING	=	MISSING
NO	+	-	=	NO
MISSING	+	YES	=	NO
MISSING	+	NO	=	YES
MISSING	+	MISSING	=	MISSING

Note: *This outcome will be derived only among babies, whom their status at birth was alive, based on the answer of the following question reported on the Delivery Form:*

3.8 Status of baby at birth

- **Gestational age at screening**

The gestational age at screening will be calculated by the formula: (280 – (EDD – Screening Date))/7.

The Estimate Date of Delivery (EDD) reported on the Screening, Consent and Key Baseline Information Form by answering the following question:

2.6 Please indicate Estimated Date of Delivery (EDD):

The screening date reported on the Screening, Consent and Key Baseline Information Form by answering the following question:

1.5 Date of screening :

The gestational age at screening for each woman will be used either as two separate figures indicating the week and the exact day in this week of the gestational age or as one figure of a decimal system.

- **Maternal age at consent**

Reported on the Screening, Consent and Key Baseline Information Form by answering the following questions:

1.3 Woman's DOB (e.g. JAN2017)
Date of consent:
Because woman's full date of birth is not collected for data protection reason (only month and year collected), the 15th of each month will be considered as the day in each date of birth in order to calculate the date of birth for each woman. Age at consent will be calculated by the formula:

(ConsentDate-DOB)/365.25

- **Maternal age at randomisation**

Reported on the Screening, Consent and Key Baseline Information Form by answering the following question:

1.3 Woman's DOB (e.g. JAN2017)

And on the Reconfirmation of Eligibility & Randomisation Form by answering the following question:

  - Date of randomisation:

Because woman's full date of birth is not collected for data protection reason (only month and year collected), the 15th of each month will be considered as the day in each date of birth in order to calculate the date of birth for each woman. Age at randomisation will be calculated by the formula:
(RandDate-DOB)/365.25

- **Gestational age at randomisation**

The gestational age at randomisation will be calculated by the formula: (280 – (EDD – RandDate))/7.

The Estimate Date of Delivery (EDD) reported on the Screening, Consent and Key Baseline Information Form by answering the following question:

2.6 Please indicate Estimated Date of Delivery (EDD):

The randomisation date reported on the Reconfirmation of Eligibility & Randomisation Form by answering the following question:

  - Date of randomisation:

The gestational age at randomisation for each woman will be used either as two separate figures indicating the week and the exact day in this week of the gestational age or as one figure of a decimal system.

- **Gestational age at consent**

The gestational age at consent will be calculated by the formula: (280 – (EDD – ConsentDate))/7.

The Estimate Date of Delivery (EDD) and the consent date reported on the Screening, Consent and Key Baseline Information Form by answering the following question:

2.6 Please indicate Estimated Date of Delivery (EDD):

Date of consent:

The gestational age at consent for each woman will be used either as two separate figures indicating the week and the exact day in this week of the gestational age or as one figure of a decimal system.

- **Gestational age at baby's date of birth**

The gestational age at baby's date of birth will be calculated by the formula: (280 – (EDD – BabyDOB))/7.
The Estimated Date of Delivery (EDD) is reported on the Screening, Consent and Key Baseline Information Form by answering the following question:
2.6 Please indicate Estimated Date of Delivery (EDD):
The baby's date of birth reported on the Delivery Form by answering the following question:
3.4 Baby's date of birth

The gestational age at baby's date of birth for each woman will be used either as two separate figures indicating the number of weeks and the number of remaining days of the gestational age or as one figure of a decimal system.

- **Gestational age at initiation of birth**

The gestational age at initiation of birth will be calculated only for women who were induced or had no labour (Caesarean before labour) and by using the formula: (280 – (EDD – Date of Initiation))/7.

The Estimate Date of Delivery (EDD) reported on the Screening, Consent and Key Baseline Information Form by answering the following question:

2.6 Please indicate Estimated Date of Delivery (EDD):

The initiation of delivery date reported on the Delivery Form by answering the following question:

3.2.2 If induced or no labour (Caesarean before labour), on what date was delivery initiated?

The gestational age at initiation of birth for each woman will be used either as two separate figures indicating the week and the exact day in this week of the gestational age or as one figure of a decimal system.

- **Days of admission to primary hospital episode**

Reported on the Delivery Form by answering the following question:

2.1 Was the baby born in hospital?

3.4 Baby's date of birth

3.8 Status of baby at birth

On the SAE Form by answering the following questions:

5.1 What was the nature of the expeditable SAE?

Neonatal death

If death, what was the date of death?

And on the Neonatal Form by answering the following questions:

2.1 Was the baby admitted to a neonatal care unit?

2.2.1.1 1st admission, DATE admitted to neonatal care

2.11 Was the baby discharged home?

If yes, date of discharge home

If no, was the baby transferred to another hospital?

If yes, and the baby was transferred to another hospital, was this prior to 28 days of life?

Date of discharge from that hospital

<u>For neonates who were born alive in the hospital the neonatal days of admission after birth will be derived according to the following instructions:</u>

- For a neonate discharged home after birth:

Days of admission after birth= Date of discharge home – Baby's date of birth

- For a neonate transferred to another hospital and was discharged from that hospital:

Days of admission after birth=Date of discharge from that hospital – Baby's date of birth

- For a neonate who died before was discharged home or transferred to another hospital and died before was discharged from that hospital:

Days of admission after birth= Date of neonatal death – Baby's date of birth

- For a neonate where neonatal death was not recorded and neonatal is still in the hospital they were admitted or the hospital they were transferred if this occurred:

Days of admission after birth=Date of dataset freeze – Baby’s date of birth

For neonates who were admitted to a neonatal care unit after birth the neonatal days of admission after birth will be derived according to the following instructions:

- For a neonate discharged home after admission:

Days of admission after birth=Date of discharge home - Date of admission

- For a neonate transferred to another hospital and was discharged from that hospital:

Days of admission after birth=Date of discharge from that hospital – Date of admission

- For a neonate who died before was discharged home after admission; or was transferred to another hospital and died before was discharged from that hospital:

Days of admission after birth=Date of neonatal death – Date of admission

- For a neonate where neonatal death was not recorded and they are still in the hospital they were admitted or the hospital they were transferred if this occurred:

Days of admission after birth=Date of dataset freeze – Date of admission

For neonates who were not born alive 0 days of admission will be recorded.

- **Time of completion of SIX-week Postpartum Form**

The time of completion of SIX-week Postpartum Form will be calculated by the formula:

AdministeredDate – BabyDOB

In which the AdministeredDate is reported on the SIX-week Postpartum Form by answering the following question:

Date questionnaire was administered:

And the BabyDOB is reported the Delivery Form by answering the following question:

3.4 Baby's date of birth
