# Supplementary material for: Determining optimal timing of birth for women with chronic or gestational hypertension at term: The WILL (When to Induce Labour to Limit risk in pregnancy hypertension) randomised trial
Source: PLoS Med. 2024 Nov 26;21(11):e1004481. doi: 10.1371/journal.pmed.1004481 (PMC11593758; doi:10.1371/journal.pmed.1004481)
Supplement: S2 Appendix — (DOCX) [file pmed.1004481.s004.docx]

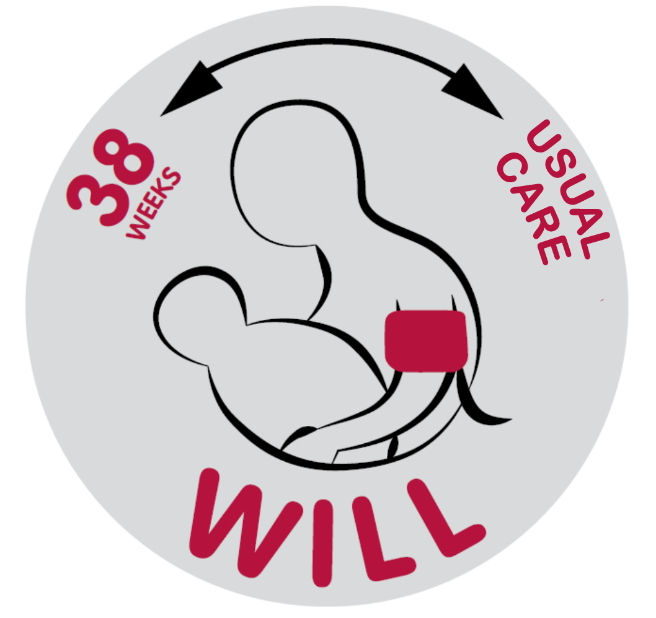


**Health Economics Analysis Plan (HEAP) for the WILL (When to Induce Labour to Limit risk in pregnancy hypertension) Trial**

Contents

[Section 1: Administrative Information 4](#_Toc150795268)

[1.1 HEAP Administrative Information 4](#_Toc150795269)

[Section 2: Trial Introduction & Background 5](#_Toc150795270)

[2.1 Trial Background and Rationale 5](#_Toc150795271)

[2.2 Aim of the Trial 5](#_Toc150795272)

[2.3 Objectives of the trial 5](#_Toc150795273)

[2.4 Trial population 5](#_Toc150795274)

[2.5 Intervention and comparators 6](#_Toc150795275)

[2.7 Trial start and end dates 6](#_Toc150795276)

[Section 3: Economic Approach 6](#_Toc150795277)

[3.1 Aims of economic evaluation 6](#_Toc150795278)

[3.2 Objectives of economic analysis 7](#_Toc150795279)

[3.3 Overview of economic analysis 7](#_Toc150795280)

[3.4 Jurisdiction 7](#_Toc150795281)

[3.5 Perspectives 7](#_Toc150795282)

[Section 4: Economic Data Collection and Management 7](#_Toc150795283)

[4.1 Statistical software use for health economic analysis 7](#_Toc150795284)

[4.2 Identification of resources 7](#_Toc150795285)

[4.3 Measurement of resource use data 8](#_Toc150795286)

[4.4 Valuation of resource use data 8](#_Toc150795287)

[4.5 Identification of outcomes 8](#_Toc150795288)

[4.6 Measurement of outcomes 8](#_Toc150795289)

[Section 5: Economic Data Analysis 8](#_Toc150795290)

[5.1 Analysis population 8](#_Toc150795291)

[5.2 Timing of analyses 8](#_Toc150795292)

[5.3 Discount rates for costs and benefits 9](#_Toc150795293)

[5.4 Cost-effectiveness threshold(s) 9](#_Toc150795294)

[5.5 Analysis of resource use 9](#_Toc150795295)

[5.6 Analysis of costs 9](#_Toc150795296)

[5.7 Analysis of outcomes 9](#_Toc150795297)

[5.8 Data cleaning for analysis 9](#_Toc150795298)

[5.9 Missing data 9](#_Toc150795299)

[5.10 Analysis of cost-effectiveness 10](#_Toc150795300)

[5.11 Sampling uncertainty 10](#_Toc150795301)

[Section 6: Reporting/Publishing 10](#_Toc150795302)

[6.1 Reporting standards 10](#_Toc150795303)

[6.2 Reporting deviations from the HEAP 10](#_Toc150795304)

[References 10](#_Toc150795305)

## Section 1: Administrative Information

## HEAP Administrative Information

| Title | | Health economics analysis plan for the WILL trial: a multi-centre randomised controlled trial to evaluate the clinical effectiveness and cost-consequences of planned early term delivery at 38^+0^ to 38^+3^ weeks’ gestation, compared with usual care at term, in pregnant women with chronic or gestational hypertension that develops by 37^+6^ weeks’ gestation. | | |
| --- | --- | --- | --- | --- |
| Trial registration number; registry | | ISRCTN77258279 (ISRCTN Registry) | | |
| Source of funding | | National Institute for Health Research, Health Technology Assessment; Reference number - 16/167/123 | | |
| Purpose of HEAP | | The purpose of this HEAP is to describe the analysis and reporting procedure intended for the economic analyses to be undertaken. The analysis plan is designed to ensure that there is no conflict with the protocol and associated statistical analysis plan. It is to be read in conjunction with them.  The plan also describes the circumstances under which amendments to planned analysis are permitted and the documentation of such changes; any deviations from this plan will be justified in the final report. The analysis plan is designed as a working document that will evolve throughout data collection, data cleaning and preliminary descriptive analysis. | | |
| Trial protocol version; date | | This document has been written based on the information contained in the trial protocol version 4.0, dated 25 May 2022 | | |
| Trial Statistical Analysis Plan (SAP) | | This document has been written based on the information contained in the trial SAP. | | |
| Trial HEAP version, date | | HEAP Version: 2.0, Date: 01 June 2023 | | |
| Roles and responsibilities | | This HEAP was prepared by Eleanor Williams and Mishal Javed (research fellow in health economics) and approved by Professor Tracy Roberts and Dr Jesse Kigozi. The trial health economist(s) [Mishal Javed] are responsible for conducting and reporting the economic evaluation in accordance with the HEAP. | | |
| APPROVALS  The following people have reviewed the Health Economics Analysis Plan and are in agreement with the contents. | | | | |
| **Role** | **Name** | | Signature | **Date** |
| Author | Mishal Javed | | **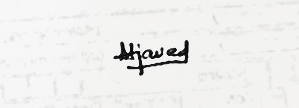** | 4 Dec 2023 |
| Lead Health Economist | Dr Jesse Kigozi | | **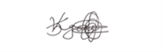** | 5 Dec 2023 |
| Chief Investigator | Prof Laura A. Magee | | **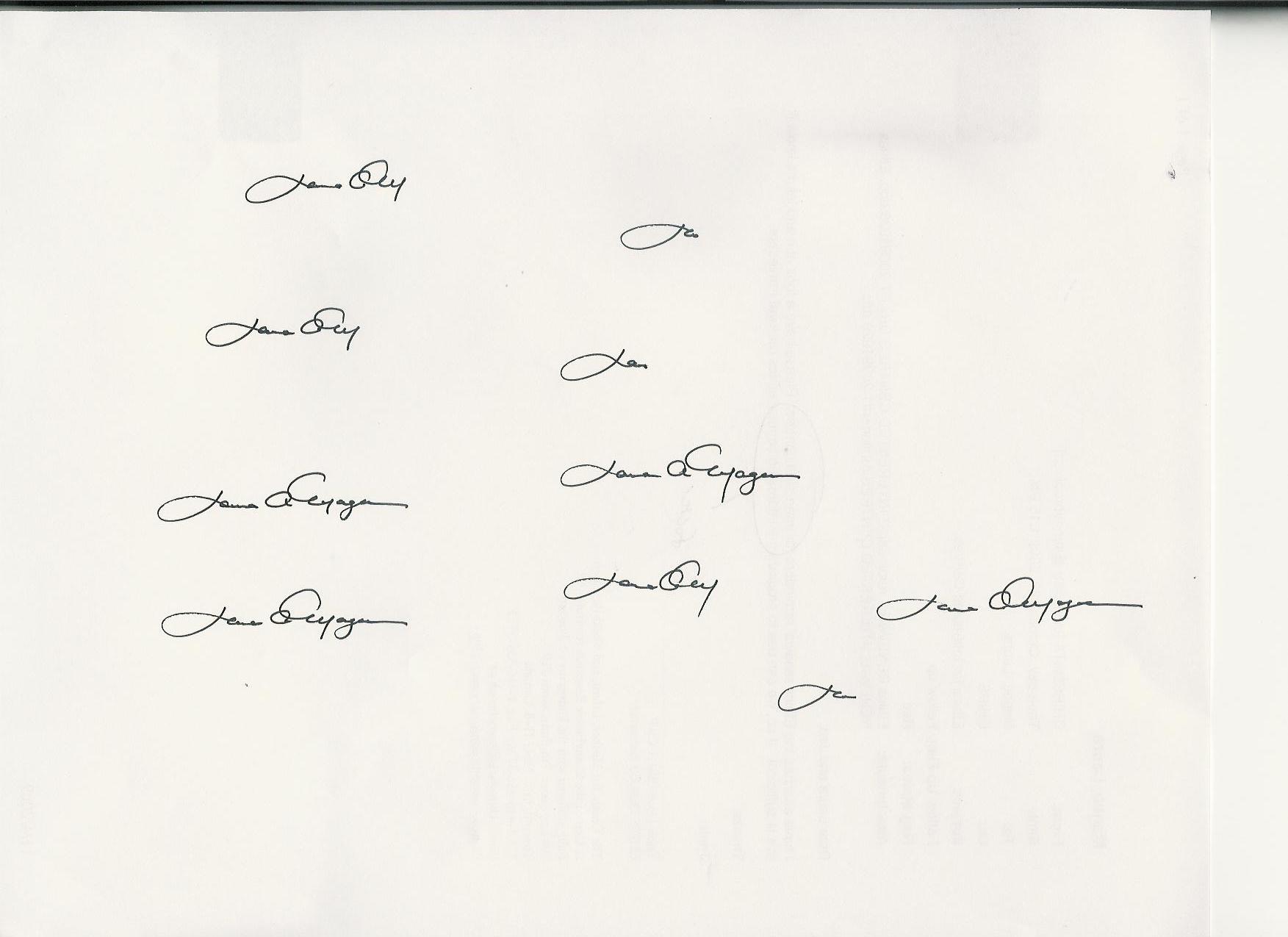** | 12 Dec 2023 |

## Section 2: Trial Introduction & Background

## 2.1 Trial Background and Rationale

Up to 55,000 pregnancies are complicated by chronic or gestational hypertension each year in the UK. Early delivery (at 37-38 weeks) may reduce maternal complications and stillbirth. However, it may increase neonatal morbidity and costs, related primarily to the cost of maternal and fetal surveillance during expectant care and possibly increased Caesarean deliveries. At present, there is no high-quality data on which to inform the care of these women.

The WILL trial is a UK-wide multi-centre randomised controlled trial to determine whether planned early term delivery at 38^+0^ to 38^+3^ weeks improves maternal and neonatal clinical outcomes relative to usual care at term. On 11 August 2022, the control arm was changed from ‘expectant care until at least 40^+0^ weeks’ to ‘usual care at term’. A full description of the study context, setting, patients and interventions is provided in the study protocol.

## 2.2 Aim of the Trial

Briefly, the WILL trial aims to inform optimal timing of delivery for women with chronic or gestational hypertension who reach term gestational age and are otherwise well. The study will provide data for women to make informed choices about maternal and perinatal risk and the NHS to plan services.

## 2.3 Objectives of the trial

Primary objective: to evaluate if planned early term delivery at 38^+0^ to 38^+3^ weeks’ gestation, compared with usual care at term, in pregnant women with chronic or gestational hypertension that develops by 37^+6^ weeks’ gestation reduces a composite of ‘poor maternal outcome’, without unduly increasing neonatal care unit admission for ≥ 4 hour measured to hospital discharge or 28 days after delivery (whichever is earlier).

Secondary objective: to evaluate the response of planned early term delivery at 38^+0^ to 38^+3^ weeks, compared with usual care at term, in pregnant women with chronic or gestational hypertension that develops by 37^+6^ weeks on maternal and neonatal clinical outcomes and cost-consequence outcomes from an NHS perspective.

## 2.4 Trial population

*Inclusion Criteria:* Patients of maternal age (16 years old and above) with a diagnosis of chronic or gestational hypertension and singleton pregnancy, live fetus (confirmed by auscultation of fetal heart tones within one week before consent) with gestational age of 36^+0^ to 37^+6^ weeks (measured by dating ultrasound or the last menstrual period). Patients must be able to give documented informed consent to participate.

*Exclusion criteria*: Contraindication to either one of the trial arms (e.g., evidence of pre-eclampsia), severe hypertension (i.e., BP ≥160mmHg systolic or ≥110mmHg diastolic) until BP is controlled (‘controlled’ blood pressure is defined as an sBP <160mmHg and dBP <110mmHg.), major fetal anomaly anticipated to require neonatal care unit admission, participation in another timing of delivery trial.

## 2.5 Intervention and comparators

Women will be randomised to one of two delivery timing approaches:

*Intervention:* Planned early term delivery at 38^+0^ to 38^+3^ weeks by labour induction (local protocol) or elective Caesarean (if previously indicated).

*Comparator:* Usual care at term, with maternal and fetal monitoring (local protocol), awaiting spontaneous labour or delivery indicated by clinical need (e.g., refractory severe hypertension or pre-eclampsia). On 11 August 2022, the control arm was changed from ‘expectant care until at least 40^+0^ weeks’ to ‘usual care at term’

2.6 Trial design

This is a pragmatic, parallel-group, open-label, multicentre, randomised controlled trial (with a 9-month internal pilot). A sample size of 1,080 pregnant women from NHS consultant lead maternity units from across the UK is needed for 90% power.

## 2.7 Trial start and end dates

Recruitment started in June 2019. The trial was paused from 24^th^ March to 6^th^ July 2020 due to COVID-19. Recruitment finished in December 2022. The end of trial will be five months after the last woman is recruited, based on the time needed for: women to deliver (up to five weeks after consent), follow-up to six weeks postpartum for maternal morbidity (six weeks), and data collection and cleaning (up to another eight weeks).

## Section 3: Economic Approach

## 3.1 Aims of economic evaluation

The economic evaluation will address the study hypothesis that a policy of planned early term delivery at 38^+0^ to 38^+3^ weeks (vs. a policy of usual care at term) will result in fewer women with poor maternal outcome, without increasing either problems for the baby (high level neonatal care for ≥4hr) or Caesarean delivery, and so lower health care costs.

## 3.2 Objectives of economic analysis

The objective of the economic evaluation is to estimate the costs and outcomes of planned early term delivery at 38^+0^ to 38^+3^ weeks for pregnant women with chronic or gestational hypertension in comparison with usual care at term.

## 3.3 Overview of economic analysis

The base-case economic analysis will take the form of a cost-consequence analysis (CCA). Given the composite nature of the outcomes which affect both the mother and baby, a CCA is deemed the most appropriate first approach to address the resource use implications. A CCA presents, in a disaggregated form, data on the incremental cost and the important consequences of the alternatives as assessed in the trial. Thus, it can be identified if any strategy shows clear dominance.

## 3.4 Jurisdiction

The trial is conducted in the UK which has a national health service (NHS), providing publicly funded healthcare, primarily free of charge at the point of use.

## 3.5 Perspectives

The economic analysis will be conducted from the NHS perspective; therefore, only direct healthcare costs are considered.

3.6 Time horizon

The economic analysis will compare the costs and consequences of each arm up to primary hospital discharge or 28 days postpartum (whichever is earlier) from the NHS consultant-led maternity unit.

## Section 4: Economic Data Collection and Management

## 4.1 Statistical software use for health economic analysis

StataSE version 16.1 or higher will be used for the exploratory and statistical analyses.^1^

## 4.2 Identification of resources

The following items of health care resource use that may differ between arms will be measured: outpatient visits; hospital admissions; tests of maternal or fetal well-being; obstetric care; and neonatal care.

## 4.3 Measurement of resource use data

Resource-use data will be collected until primary hospital discharge using case report forms (CRFs), completed by the trial staff. Data reported on each form will be consistent with the source data and any discrepancies will need to be clarified by site staff.

## 4.4 Valuation of resource use data

All resource use will be valued in monetary terms using appropriate UK unit costs estimated at the time of analysis (2021-2022). Estimates of the respective unit costs will be obtained from routine sources (e.g., NHS reference costs will be employed to value hospital resource use (e.g., Relph *et al.’s* systematic review of costing the impact of interventions during pregnancy in the UK)).^3-5^ Estimates of the unit costs will be checked against locally available data from hospitals in the study.

## 4.5 Identification of outcomes

The outcomes of the economic analysis were based on the disaggregated outcomes for the trial including poor maternal composite outcomes avoided, and neonatal care unit admission for ≥ 4 hours avoided.

Some secondary clinical outcomes will also be explored in the economic analysis, for example, Caesarean delivery and pre-eclampsia.

## 4.6 Measurement of outcomes

Measurements will be recorded at primary hospital discharge or 28 days postpartum, whichever is earliest.

## Section 5: Economic Data Analysis

## 5.1 Analysis population

The full analysis set will include all randomised participants, which is in accordance with the “intention to treat” (ITT) principle. In addition, a per-protocol set will include all participants in the full analysis set who were adherent to the protocol.

## 5.2 Timing of analyses

The final analysis will be conducted once all women have completed the 6-week postpartum assessment and corresponding outcome data have been entered onto the study database and validated as being ready for analysis. The economic analysis will take on the time-horizon of up until primary hospital discharge or 28 days postpartum (whichever is earlier).

## 5.3 Discount rates for costs and benefits

The time-horizon for the economic analysis is less than 12 months; therefore, discounting will not be applied.

## 5.4 Cost-effectiveness threshold(s)

Given that the base-line economic evaluation will take the form of a cost-consequence analysis (CCA), there is no defined cost-effectiveness threshold. The disaggregated format of the CCA necessitates the decision-maker forms their own opinion when comparing the alternative interventions.

## 5.5 Analysis of resource use

Differences in the use of services between randomised groups will be described.

## 5.6 Analysis of costs

Overall mean costs and measures of their variance for both arms of the trial will be calculated. Using non-parametric bootstrapping methods, we will estimate the difference in mean total costs between the two arms of the trial and 95% confidence intervals by analysing 1000 resamples.

## 5.7 Analysis of outcomes

Differences in the outcomes between randomised groups will be described but not compared statistically.

## 5.8 Data cleaning for analysis

Exploratory analysis will be undertaken to ensure ranges and distributions of variables used in the economic analysis are appropriate. Descriptive statistics of data, by arm, such as means, medians, and frequencies will be presented. We will liaise with trial statisticians in identifying issues with data such as miscoding. Data cleaning will include face validity tests conducted on data (e.g., to identify misspelt text) which will be checked against the source documents. Any remaining areas of uncertainty will be discussed between the health economists and, where necessary, referred for adjudication by a clinical expert. Corrections identified will be documented in the Stata code.

## 5.9 Missing data

The trial data will be examined for any missing data where appropriate. The appropriate method for dealing with missing data will depend on the prevalence of missing data and probable mechanism of missingness. For example, multiple imputation methods may be used if the data is missing at random (MAR).

## 5.10 Analysis of cost-effectiveness

As the economic analysis takes the form of a CCA, disaggregated costs and outcomes will be presented to provide indication of cost and the important consequences of the alternatives as assessed in the trial. An explicit statement of cost-effectiveness will not be made.

## 5.11 Sampling uncertainty

A non-parametric bootstrapping approach will be used to determine the level of sampling uncertainty relating to costs and outcome estimates.

## Section 6: Reporting/Publishing

## 6.1 Reporting standards

CHEERS guidelines will be followed when reporting the health economic evaluation, in a format that is appropriate to policymakers and stakeholders.

## 6.2 Reporting deviations from the HEAP

Any deviation from HEAP will be described and justified in the final published report. Circumstances under which changes will be permitted are as follows:

- development of statistical methods that are deemed more appropriate for the analysis
- clarification of currently debated issues
- preliminary data cleaning or analysis suggesting that planned analyses may require amendment

## References

1. StataCorp. 2019. *Stata Statistical Software: Release 16*. College Station, TX: StataCorp LLC.

2. Curtis, L. A. & Burns, A. 2019. *Unit Costs of Health and Social Care 2019*. Kent, UK: PSSRU.

3. NHS. 2019. *Reference Cost Collection: National Schedule of Reference Costs, 2018-19.* London, UK

4. British National Formulary, 2020. *BNF British National Formulary - NICE*. [online] Bnf.nice.org.uk. Available at: <https://bnf.nice.org.uk/> [Accessed 3 November 2020].

5. Relph, S., Delaney, L., Melaugh, A., Vieira, M.C., Sandall, J., Khalil, A., Pasupathy, D. and Healey, A., 2020. Costing the impact of interventions during pregnancy in the UK: a systematic review of economic evaluations. *BMJ open*, *10*(10), p.e040022.
