# Supplementary material for: Determining optimal timing of birth for women with chronic or gestational hypertension at term: The WILL (When to Induce Labour to Limit risk in pregnancy hypertension) randomised trial
Source: PLoS Med. 2024 Nov 26;21(11):e1004481. doi: 10.1371/journal.pmed.1004481 (PMC11593758; doi:10.1371/journal.pmed.1004481)
Supplement: S3 Appendix — (DOCX) [file pmed.1004481.s005.docx]

**SUPPLEMENTARY TABLES**

| **Document** | **Title** | **Page number** |
| --- | --- | --- |
| A | WILL Trial Study Group | 2 |
| B | Trial maternal co-primary outcome | 5 |
| C | Secondary outcomes: maternal, fetal/neonatal, and health economic | 7 |
| D | National Health Service (UK) reference costs in 2020/21 British Pounds | 9 |
| E | Reasons for declining participation | 10 |
| F | Baseline characteristics presented in detail | 11 |
| G | Adherence to intervention and birth initiation, presented in detail | 13 |
| H | Subgroup analyses for maternal and neonatal co-primary outcomes | 15 |
| I | Sensitivity analyses for maternal and neonatal co-primary outcomes, and key secondary outcome | 19 |
| J | Other maternal outcomes presented in detail | 22 |
| K | Mean costs in 2020/21 British Pounds | 26 |

**Table A**: The WILL (When to Induce Labour to Limit risk in pregnancy hypertension) Trial Study Group

| **From Co-investigator and Trial Management Groups** |
| --- |
| Laura A. Magee (Chief Investigator), Peter Brocklehurst, Lucy Chappell, Sean Cole, Jon Dorling, Ruth Evans, Max Feltham, Eleni Gkini, Marcus Green (PPIE), Pollyanna Hardy, Jennifer Hutcheon, Katie Kirkham, Lisa Leighton, Catherine Moakes, Ben Mol, Katie Morris, Mary Nulty, Paul Riley, Tracy Roberts, Janet Scott (PPIE), Joel Singer, Clive Stubbs, Kiran Sunner, Jim Thornton, Sue Tohill, Peter von Dadelszen, Julie Wade |
| **Trial Management Group** |
| Laura A. Magee (Chief Investigator), Peter von Dadelszen, Eleni Gkini, Katie Kirkham, Catherine Moakes, Paul Riley, Clive Stubbs, Jim Thornton, Sue Tohill |
| **Co-Investigator Group** |
| Laura A. Magee (Chief Investigator), Peter Brocklehurst, Lucy Chappell, Peter von Dadelszen, Jon Dorling, Pollyanna Hardy, Jennifer Hutcheon, Ben W. Mol, Tracy Roberts, Janet Scott, Joel Singer, Jim Thornton |
| **From WILL trial sites** |
| **Airedale General Hospital:** Sumita Bhuiya, Soumendra Nallapeta, Emma Dooks, Sophie Packham, Chantal McParland |
| **Birmingham Women's Hospital**, Birmingham: Diane Whitehouse, Chloe O'Hara, Connie Weston, Diane Mellers, Lesley Brittain, Phern Adams, Katie Morris, Rebecca Shakespeare, Emily Pain, Natalie Bayne, Sethenia Beckford, Sunena Verma |
| **Bradford Teaching Hospitals NHS Foundation Trust**, Bradford: Sudeepthi Kakara, Janet Wright, Amal Mighell, Jennifer Syson, Kari Swettenham, Shaila Seraj, Georgina Goodaire, Jenny Butler, Kate Pittendreigh, Liz Ingram, Hannah Brooks |
| **Cardiff and Vale University Health Board:** Maryanne Bray, Claire Bertorelli, Hannah Ritter, Emma Pugh |
| **Chesterfield Royal Hospital:** Janet Cresswell, Mary Kelly-Baxter, Li-Shan Yeoh, Shailly Sahu Bhansali, Vandana More, Fiona Warburton, Lauren Bishop, Nikit Kadam, Shefali Rathee, Deepika Goyal |
| **Croydon University Hospital**, Croydon: Bini Ajay, Geraldine Upson, Danielle Hake, Diana Opoku, Emma Wayman, Natalia Cwiek, Stacy Tregellas, Nikki Lee |
| **Cwm Taf Morgannwg UHB:** Lavinia Margarit, Joelle Pike, Kate Jones, Sophie-Mae Wheeler-Davies, Meena Ali, Vikki Drake, Deborah Jones |
| **East Surrey Hospital:** Indhuja Rajkumar, Ruth Habibi, Sarah Davies, Emma Crawley, Katie Prickett, Kopal Agarwal, Rebecca Low, Sumit Kar, Kate Stringer |
| **Glangwili Hospital:** Harinakshi Salian, Trudy Smith, Anangsha Kumar |
| **The James Cook University Hospital**, Middlesbrough: Deepika Meneni, Hazel Alexander, Helen Harwood, Kerry Hebbron, Lynn Whitecross, Mary Hodgers, Shilpa Mahadasu, Rebecca Fletcher |
| **King's College Hospital:** Nick Kametas, Yasmin Sana, Hayley Martin, Rebecca Jarman, Sophie Webster, Gillian Godwin, Leia Parry, Naomi Grimes |
| **King’s Mill Hospital:** Jyothi Rajeswary, Mandy Gill, Emily Omuvwie, Rachel Johnson, Susan Smith |
| **Kingston Hospital:** Gabrielle Bambridge, Danielle Hake, Isabel Bradley, Kristina Sexton, Lola Oshodi, Kathryn Sollesta |
| **University Hospitals of Leicester NHS Trust**, Leicester: Cornelia Wiesender, Claire Dodd, Rupa Modi, Beverley Cowlishaw, Gina Mulheron, Magdalena Kierzenkowska, Molly Patterson, Patricia Amos, Sharon Marie Bates, Sharon Raper, Anna Holt, Sarah Evans |
| **Liverpool Women’s Hospital**, **Liverpool:** Umber Agarwal, Ruth Cockerill, Amy Mahdi, Caroline Cunningham, Michelle Dower, Sian Rogers, Siobhan Holt, Carly Williams, Zora Castling, Linda Watkins, Rachel McFarland |
| **New Cross Hospital, Wolverhampton:** David Churchill, Ellmina McKenzie, Julie Icke, Laura Devison, Lindsey Jarosz, Philippa Rafferty |
| **North West Anglia NHS Foundation Trust, Peterborough City Hospital and Hinchingbrooke Hospital (closed):** Sreejith Kodakkattil, Charleen Lia, Coralie Huson, Jodi Carpenter, Kimberley Morris |
| **Northumbria Specialist Emergency Care Hospital:** Vinita Raheja, Angela Ayuk, Jessica Reynolds, Julie Wyton, Stacey Duffy |
| **Northwick Park Hospital:** Sahana Gupta, Anam Fayadh, Sean Connarty |
| **Nottingham City Hospital**, Nottingham**:** Kate Walker, Jim Thornton, Jane Cantliffe, Catriona Hussain, Carys Smith, Harriet Anderson, Lesley Hodgen, Megan Betteridge, Nahid Kazmi, Yvette Davis |
| **Princess Anne Hospital,** Southampton: Karen Brackley, Nicki Martin Fiona Walbridge, Rhea Hampton, Alexandra Kermack, Kate Raney |
| **Queen Elizabeth Hospital King’s Lynn:** Salman Kidwai, Aricsa Joshy, Hollie Curgunven, Esther Dorken, Uma Stephen Paul |
| **Queen's Medical Centre**, Nottingham: Kate Walker, Jim Thornton, Catriona Hussain, Jane Cantliffe Carys Smith, Lesley Hodgen, Nia Jones, Julie Shaw, Megan Betteridge, Nahid Kazmi, Yvette Davis |
| **Raigmore Hospital (Highland):** Charlotte Barr, Deborah McDonald, Donna Patience, Jandy Fernandes, Sadia Akhtar, Shona Macleod |
| **Royal Berkshire Hospital:** Patrick Bose, Anna Campbell, Catherine Young, Fidelma Lee, Sharon Westcar |
| **Royal Bolton Hospital (closed):** Rebecca Peart, Emma Tanton, Kat Rhead |
| **Royal Cornwall Hospital:** Kristin Fiedler, Ruth Bowen, Richard Keedwell |
| **Royal Oldham Hospital:** Stephy Mathen, Zainab Sarwar, Chloe Rishton, Chloe Scott, Marcus Cabrera-Dandy, Grainne O’Connor |
| **Royal United Hospital:** Bath (closed): Jane Farey, Nisha Verasingam, Mel Rich, Annette Moreton, Catherine Bressington, Jennifer Pullen, Sara Burnard, Wendy Duberry, Jessica Sellick |
| **Singleton Hospital**, Swansea: Madhuchanda Dey, Sharon Jones, Pauline Bird, Joelle Morgan, Charu Gupta |
| **South Tyneside General Hospital & Sunderland Royal Hospital**, South Tyneside & Sunderland NHS Foundation Trust, Sunderland: Aarti Ullal, Eileen Walton, Ashleigh Price, Janet Scollen, Judith Ormonde, Kirsten Herdman, Lesley Hewitt, Lucy Rowland |
| **Southend University Hospital:** Mandeep Singh, Sundararajah Raajkumar, Beena Saji |
| **St George's University Hospital:** Asma Khalil, Alice Perry, Emily Marler, Ijeoma Imuzeze, Sophie Robinson, Lolade Oshodi, Hafiza Koroma |
| **St James’s University Hospital,** The Leeds Teaching Hospitals NHS Trust, Leeds: Jonathan Nelson, Kathryn McNamara, Carina Craig, Del Endersby, Jayne Wagstaff, Kate Robinson, Hannah Barnes, Jane Gavin, Emily Scriven, Peter Owegie, Linden Stocker |
| **St Mary’s Hospital, Manchester**: Jenny Myers, Kate Stanbury, Christine Hughes, Sarah Lee, Kate Duhig, Flurina Michelotti, Olivia Moran |
| **St Mary’s Maternity Unit, Poole Hospital,** University Hospitals Dorset NHS Foundation Trust: Latha Vinayakarao, Louise Melson, Stephanie Grigsby, Susara Blunden, Annemie Legg, Julie Beeson |
| **St Michael’s Hospital, Bristol:** Melanie Griffin, Sarah Newell, Katharine Jane Thompson, Brittany Smart, Elizabeth Payne, Marie Pitchford, Jing Lin |
| **St Richard’s Hospital & Worthing Hospital:** Rahila Khan, Sophia Stone, Ahmed Elgarhy, Emma Meadows, Marian Flynn-Batham, Nikky Passmore, Vivienne Cannons |
| **St Thomas’ Hospital (paused)**, Guy’s and St Thomas’ NHS Foundation Trust, London: Declan Symington, Alice Lewin, Hayley Tarft, Jessamine Hunt, Julie Wade, Sue Tohill, Zoe Vowles, Maria Slaney, Rachel Woodcock, Hilary Thompson, Lucy Chappell |
| **The Great Western Hospital, Swindon:** Alex Van der Meer, Tracey Benn, Ru Davies |
| **The Royal Victoria Infirmary, Newcastle:** Gareth Waring, Jill Riches, Andrea Fenn, Aly Kimber, Susan Harrop, Falak Diab, Angela Phillipson |
| **University College London Hospital:** Daniel Stott, Amos Tetteh, Davide Casagrandi, Miriam Bourke, Eirini Vaikousi, Rita Sarquis, Morenike Folorunsho, Olivia Newth, Sarah Weist, Yaa Acheampong, Vidhya Ravikumar, Adele Powell, Amos Tatteh, Jennifer Tshibamba, Lauren Laville, Sarah Ekladios |
| **University Hospital North Tees:** Stephen Wild, Emily Slane, Julie Woollaston, Kirsty Farrington, Sharon Gowans, Vicky Collins |
| **University Hospital of North Durham:** Jemma Yorke, Vicki Atkinson, Shelly Wood |
| **University Hospital Wishaw (Lanarkshire):** Eleanor Jarvie, Carol Hollas, Denise Vigni |
| **Walsall Manor Hospital:** Tigist Mengistu, Robert Chadwick, Helen Haden, Lisa Richardson, Iwona Wojtuszko, Susan Musa |
| **West Middlesex University Hospital (closed)**: Middlesex: Joanna Girling, Amy Barker, Elaine Palmer, Louise Page, Grace Ryan, Lauren Trepte, Samantha Steele |
| **York Hospital (closed)**, York: Jacqueline Tang, Harriet Pearson, Jo Ingham, Nicola Spark, Samantha Roche, David Thompson |

**Table B**: Trial maternal co-primary outcome, as assessed from randomisation until primary hospital discharge home or 28 days after birth (whichever is earlier)*ł

| **One/more of:** | **Definition** |
| --- | --- |
| Severe hypertension | Systolic BP ≥160mmHg or diastolic BP ≥110mmHg, measured twice, 15min apart |
| Maternal death | As stated, irrespective of the cause |
| Maternal morbidity, defined as one or more of the following: |  |
| GCS <13 | - |
| Stroke | Acute symptoms of focal brain injury that have lasted >24 hours, with type (ischaemic or haemorrhage) confirmed by neuroimaging |
| TIA | Acute symptoms of focal brain injury that have lasted <24 hours |
| Eclampsia | Onset of convulsions in a woman with pre-eclampsia, and not attributable to other causes |
| Blindness | Partial/complete, or either retinal or cortical. Retinal detachment is defined as the peeling away of the retina from its underlying layer of support tissue diagnosed by ophthalmological exam. Cortical blindness is defined as loss of visual acuity in the presence of intact pupillary response to light |
| Uncontrolled hypertension | Hypertension requiring administration of 3 or more different parenteral [intravenous or intramuscular] antihypertensive agents within a 12 hour period |
| Inotropic support | Use of vasopressors to keep sBP > 90 mm Hg or a MAP >70 mmHg |
| Pulmonary oedema | Excess fluid in the lungs diagnosed clinically with one/more of oxygen saturation <95%, directive treatment (e.g., diuretic therapy), or x-ray confirmation) |
| Respiratory failure | Intubation, ventilation by endotracheal tube or non-invasively, or need for >50% oxygen for >1 hr, not due to Caesarean delivery |
| SpO2 <90% | - |
| Myocardial ischaemia or infarction | By characteristic ECG changes and markers of myocardial necrosis |
| Hepatic dysfunction | INR>1.2 in absence of DIC or treatment with warfarin, or, in the presence of DIC or treatment with warfarin: either mixed hyperbilirubinemia >1.0 mg/dL (or >17 μM) or hypoglycaemia <45 mg/dL (<2.5 mM) in absence of insulin |
| Hepatic haematoma or rupture | Presence of a blood collection under the hepatic capsule as confirmed by imaging or at laparotomy |
| Acute kidney injury or dialysis | One/more of: serum creatinine >150µM in absence of a baseline serum creatinine; rise in serum creatinine ≥26µM within 48 hours; >50% rise in serum creatinine within the past 7 days; urine output <0.5ml/kg/hr for >6hr); or new dialysis (of any type) |
| Platelet count <50x10^9^/L | - |
| Transfusion | Of any blood product |
| Placental abruption | Diagnosed either: (i) clinically, by abdominal pain or uterine contractions of sudden onset with one/more of: vaginal bleeding other than show, intrauterine fetal death or DIC; or (ii) by presence of a retroplacental clot at time of delivery; or (iii) by placental pathology demonstrating retroplacental clot or histological findings of a chronic abruption |

*BP (blood pressure), DIC (disseminated intravascular coagulation), ECG (electrocardiogram), GCS (Glasgow Coma Scale), MAP (mean arterial pressure), sBP (systolic blood pressure), SpO2 (peripheral arterial oxygen saturation), TIA (transient ischaemic attack)*

** For women who were consented but not randomised, the co-primary outcomes were assessed from consent (rather than randomisation).*

*ł Outcomes highlighted in blue are core maternal outcomes in hypertensive pregnancy [BJOG. 2020 Nov;127(12):1516-1526. doi: 10.1111/1471-0528.16319].*

**Table C:** Secondary outcomes: maternal, fetal/neonatal, and health economic*

| **Maternal** |
| --- |
| Caesarean delivery (key maternal), presented descriptively elective or non-elective, following either spontaneous onset labour or labour induction (that resulted in either no labour or labour) |
| Instrumental vaginal delivery or Caesarean delivery (vs. spontaneous vaginal delivery), with indications |
| Infection of the Caesarean wound, episiotomy, or vaginal tear, as applicable, at six weeks postpartum |
| Individual components of maternal co-primary outcome (as defined in **Table 1**), up to discharge or 28 days postpartum (whichever is earlier) |
| Poor maternal outcome (assessed as one or more of the components of the maternal co-primary outcome) measured at six weeks postpartum (as assessed post-discharge after birth by maternal questionnaire) |
| Elevated liver enzymes (AST or ALT >40 IU/L) |
| Platelet count <100x10^9^/L |
| Pre-eclampsia by ISSHP 2018 criteria*[6] |
| PPH (perceived abnormal bleeding following birth and either hypotension or medical/surgical intervention for postpartum haemorrhage) |
| Sepsis (known or suspected maternal infection with two or more of Quick Sequential Organ Failure Assessment criteria: respiratory rate ≥22/min, altered mentation, or systolic BP ≤100mmHg) |
| ITU admission (to receive advanced respiratory support alone or monitoring and support for two or more organ systems) |
| Potential co-interventions (post-randomisation), before birth admission unless otherwise specified: |
| Antihypertensive therapy taken and type (antepartum, postpartum, or at either time point) |
| Magnesium sulphate (antepartum or postpartum) |
| Bedrest at home |
| Use of home BP monitoring |
| Maternal blood or urine testing at the laboratory prior to birth admission, and number of such episodes of testing (median [IQR]) |
| Seen as outpatient (in office/clinic) and number of visits (median [IQR]) |
| Seen as outpatient (in her home) and number of visits (median [IQR]) |
| Where available, seen in medical, day, or maternity assessment unit and number of visits (median [IQR]) |
| Seen in an acute care area (such as Accident & Emergency) for urgent/emergent visit other than in labour and number of such visits (median [IQR]) |
| Number of antenatal admission days prior to birth (median [IQR]) |
| Underwent fetal cardiotocography |
| Underwent fetal ultrasound |
| Clinical indications for birth |
| Maternal satisfaction assessed at hospital discharge or 28 days postpartum (whichever is earlier), as measured by the Childbirth Experience Questionnaire, assessed as the overall score, and domain scores (i.e., own capacity, professional support, perceived safety, and participation) |
| Serious adverse eventǂ (i.e., that resulted in death, was life-threatening, required hospitalisation or prolongation of existing hospitalisation, resulted in persistent or significant disability or incapacity, may have jeopardised the pregnancy, or may have required intervention to prevent one of the other outcomes listed above) |
| **Fetal/neonatal** |
| Neonatal care unit admission ≥4 hours assessed to 28 days after birth |
| Indication for neonatal care unit admission for ≥4 hours as a respiratory problem, as identified by the clinical team by the principle indication for admission on the BadgerNet discharge summary (with the clinical diagnosis presented descriptively, as meconium aspiration syndrome, pneumonia, pneumothorax/pneumomediastinum, transient tachypnoea of the newborn, or ‘other’ [specified]) |
| Other indications, as identified clinically, will be presented descriptively (e.g., 5-min Apgar score <7, birthweight <10th centile, birthweight >90th centile, sepsis work-up, hyper- or hypo-glycaemia, or other) |
| Respiratory morbidity, defined as the need for supplemental oxygen and/or positive pressure ventilation beyond the initial resuscitation period |
| Clinical respiratory problem, defined as meconium aspiration syndrome, pneumonia, pneumothorax/pneumomediastinum, transient tachypnoea of newborn, or other [unspecified]) |
| Chest x-ray, N performed, N abnormal and nature of abnormality (i.e., meconium aspiration syndrome, pneumonia, pneumothorax/pneumomediastinum, transient tachypnoea of newborn, or other [unspecified]) |
| HIE, defined as therapeutic hypothermia for ≥72 hours |
| Sepsis requiring antibiotics for at least five days, with confirmed blood or cerebrospinal fluid culture |
| Major operation (laparotomy, thoracotomy, craniotomy, or other) |
| Birthweight |
| Apgar scores (recorded at 1, 5, and 10 minutes) |
| Stillbirth (i.e., death of a fetus after randomisation) |
| Neonatal death (of a liveborn infant within the first 28 days of birth) |
| Breastfeeding established assessed at hospital discharge or 28 days postpartum (whichever is earlier) |
| Exclusive breastfeeding assessed at hospital discharge or 28 days postpartum (whichever is earlier) |
| **Health economic** |
| Cost-consequence analysis from NHS perspective (enrolment to hospital discharge) |

*BP (blood pressure), ITU (intensive care unit), PPH (postpartum haemorrhage), AST or ALT (aspartate aminotransferase or alanine aminotransferase), GCS (Glasgow Coma Scale), DIC (Disseminated Intravascular Coagulation), IQR (Inter Quartile Range), HIE (Hypoxic Ischaemic Encephalopathy), NHS (National Health Service).*

** Outcomes highlighted in blue are core maternal, fetal, or neonatal outcomes in hypertensive pregnancy [Duffy J, Cairns AE, Richards-Doran D, van 't Hooft J, Gale C, Brown M, et al. A core outcome set for pre-eclampsia research: an international consensus development study. BJOG. 2020;127(12):1516-26] [1].*

*ł Pre-eclampsia is defined by ISSHP 2018 criteria[6], as chronic or gestational hypertension with development of one or more of the following new-onset conditions at ≥20 weeks: (i) proteinuria; (ii) serum creatinine ≥90µM; (iii) elevated AST or ALT to >40 IU/L; (iv) neurological complications including eclampsia, altered mental status [as measured by GCS<13], blindness, stroke, clonus, severe headache, persistent visual scotomata); (iv) haematological complications (i.e., platelet count <150x109/L, DIC, haemolysis); or (v) uteroplacental dysfunction (including fetal growth restriction defined as birthweight<10th centile presented descriptively[19], abnormal umbilical artery Doppler waveform analysis, or stillbirth).*

*ǂ Adverse events were captured via pre-defined outcome measures listed above.*

**Table D**: National Health Service (UK) reference costs, in 2020/2021 British Pounds(22)

| Resource use items | Unit cost | Source |
| --- | --- | --- |
| Outpatient visits |  |  |
| Obstetric outpatient | 186.06 | Total Outpatient Attendances (501)^(2)^ |
| Antenatal emergency visit | 170.46 | Total Outpatient Attendances (180) |
| MAU and/or DAU | 169.84 | Total HRGs (NZ22Z) |
| Community midwife | 117.54 | Total Outpatient Attendances (560) |
| Hospital admissions |  |  |
| Antenatal inpatient admission | 573.13 | Total HRGs (NZ16Z) |
| Maternal inpatient stay for delivery or within 28 days after birth | 215.95 | RP (NZ25Z, NZ26A, NZ26B, NZ27Z) |
| Maternal ICU admission | 1815.92 | CC (CCU12 XA03Z-XA07Z) |
| Tests of maternal or fetal wellbeing |  |  |
| Maternal blood and/or urine tests | 2.18 | DAPS (DAPS04, DAPSO5, DAPS08, DAPS09) |
| Foetal ultrasound | 172.88 | Total HRGs (NZ21Z-NZ22Z) |
| Foetal cardiotocograph | 169.84 | Total HRGs (NZ22Z) |
| Umbilical artery doppler | 169.84 | Total HRGs (NZ22Z) |
| Chest X-ray | 49.32 | IMAG (PF) |
| Obstetric care |  |  |
| Normal delivery (no induction) | 2729.34 | Total HRGs (NZ30A-NZ30C) |
| Normal delivery (induced) | 4064.33 | Total HRGs (NZ31A-NZ34C) |
| Assisted delivery (no induction) | 3635.84 | Total HRGs (NZ40A-NZ40C) |
| Assisted delivery (induced) | 5677.46 | Total HRGs (NZ41A-NZ44C) |
| Planned caesarean section | 5485.13 | Total HRGs (NZ50A-NZ50C) |
| Emergency caesarean section | 7586.18 | Total HRGs (NZ51A-NZ51C) |
| Neonatal care |  |  |
| Neonatal normal care unit | 769.10 | CC (XA05Z) |
| Neonatal intensive care unit | 1816.37 | CC (XA01Z) |
| Neonatal high dependency unit | 1242.97 | CC (XA02Z) |
| Neonatal special care unit | 860.38 | CC (XA03Z-XA04Z) |

*CC (critical care), DAPS (directly accessed pathology services), DAU (day assessment unit), HRG (healthcare resource groups), ICU (intensive care unit), IMAG (diagnostic imaging), MAU (medical assessment unit), PF (plain film), RP (regular day or night admissions).*

**Table E**: Reasons for declining to participate, among 582 eligible potential participants*

| **Reason(s) for not consentingł** | **N=582 women** |
| --- | --- |
| Did not want to take part in research | 119 (20.4%) |
| Did not want to be induced, if at all possible | 111 (19.1%) |
| Wanted to await onset of spontaneous labour | 84 (14.4%) |
| Wanted to be delivered at 38+0-6 weeks | 55 (9.5%) |
| Wanted to be delivered at 39+0-6 weeks | 60 (10.3%) |
| Wanted to be delivered at 40+0-6 weeks | 33 (5.7%) |
| Care-provider did not want them to participate | 84 (14.4%) |
| Other | 230 (39.5%) |
| Missing confirmatory question | 12 (2.1%) |

** Of 2822 women screened, 1030 met eligibility criteria.*

*ł Reasons for not participating are not mutually exclusive; percentages may total more than 100%.*

**Table F**: Baseline characteristics (mean±SD or N women (%) unless otherwise stated), with grey shading of characteristics not presented in Table 1.

|  | **Planned early term delivery at 38^+0-3^ wks**  **(N=201)** | **Usual care at term**  **(N=202)** |
| --- | --- | --- |
| **Demographic and other baseline variables** |  |  |
| Maternal age at randomisation (years) | 31.5±5.9 | 31.9±5.7 |
| Mother’s self-declared ethnicity |  |  |
| White | 157 (78.1) | 158 (78.2) |
| Black | 13 (6.5) | 17 (8.4) |
| Arab | 2 (1.0) | 1 (0.5) |
| South Asian | 16 (8.0) | 9 (4.5) |
| Other | 13 (6.5) | 16 (7.9) |
| Declined to give information | 0 | 1 (0.5) |
| Body mass index (kg/m^2^) |  |  |
| <18.5 | 0 | 0 |
| 18.5 – 24.9 | 30 (14.9) | 35 (17.3) |
| 25.0 – 29.9 | 59 (29.4) | 48 (23.8) |
| ≥30 | 112 (55.7) | 119 (58.9) |
| Class I obesity (30.0 – 34.9) | 47 (23.4) | 51 (25.3) |
| Class II obesity (35.0 – 39.9) | 33 (16.4) | 31 (15.4) |
| Class III obesity ($\geq$ 40.0) | 32 (15.9) | 37 (18.3) |
| **Hypertension type*** |  |  |
| Chronic | 96 (47.8) | 99 (49.0) |
| Pre-pregnancy | 64 | 69 |
| In pregnancy <20 weeks | 32 | 30 |
| Gestational | 105 (52.2) | 103 (51.0) |
| Previous severe hypertension (sBP≥160mmHg or dBP≥110mmHg) during this pregnancy | 17 (8.5) | 25 (12.4) |
| **Pre-pregnancy medical and obstetric history** |  |  |
| Pre-gestational diabetes | 1 (0.5) | 4 (2.0) |
| Diabetes (type I) | 1 (0.5) | 0 (0) |
| Diabetes (type II) | 0 (0) | 4 (2.0) |
| Renal disease | 4 (2.0) | 5 (2.5) |
| Autoimmune disease (including APAS) | 9 (4.5) | 5 (2.5) |
| HIV positive | 1 (0.5) | 0 (0) |
| Nulliparous | 96 (47.8) | 98 (48.5) |
| In parousł women | (N=105) | (N=104) |
| Prior Caesarean* | 15 (14.3) | 16 (15.4) |
| Prior gestational hypertension | 61 (58.1) | 56 (53.9) |
| Prior pre-eclampsiaǂ | 29 (27.6) | 24 (23.1) |
| **This pregnancy** |  |  |
| Conceived by artificial reproductive technologyǁ | 9 (4.5) | 6 (3.0) |
| Developed gestational diabetes in this pregnancy | 19 (9.5) | 18 (8.9) |
| Required metformin | 9/19 (47.4) | 11/18 (61.1) |
| Require insulin | 0 | 4/18 (22.2) |
| Nicotine use after 20 wks of current pregnancy | 10 (5.0) | 13 (6.4) |
| Cigarettes/cigars/pipe | 8/10 (80.0) | 11/13 (84.6) |
| Vaping | 3/10 (30.0) | 2/13 (15.4) |
| Other | 1/10 (10.0) | 0 |
| Taking low-dose aspirin to prevent pre-eclampsia | 134 (66.7) | 134 (66.3) |
| Taking calcium supplementation to prevent pre-eclampsia | 6 (3.0) | 5 (2.5) |
| **At trial enrolment** |  |  |
| GA at consent (wks) (median [IQR]) | 37.0 [36.4, 37.3] | 37.0 [36.7, 37.4] |
| 36^+0^-36^+6^ weeks | 94 (46.8) | 72 (35.6) |
| 37^+0^-37^+6^ weeks | 107 (53.2) | 130 (64.4) |
| GA at randomisation (wks) (median [IQR]) | 37.1 [37.0, 37.4] | 37.3 [37.0, 37.4] |
| **BP and antihypertensives at enrolment** |  |  |
| Taking antihypertensive medication at consent | 156 (77.6) | 165 (81.7) |
| Taking one agent | 146/156 (93.6) | 153/165 (92.7) |
| Taking two or more agents | 10/156 (6.4) | 12/165 (7.3) |
| Agents taken¶ |  |  |
| Labetalol | 106/156 (68.0) | 128/165 (77.6) |
| Nifedipine | 32/156 (20.5) | 40/165 (24.2) |
| Nifedipine long-acting | 1/32 (3.1) | 6/40 (15.0) |
| Nifedipine modified-release | 31/32 (96.9) | 35/40 (87.5) |
| Methyldopa | 23/156 (14.7) | 5/165 (3.0) |
| Other** | 5/156 (3.2) | 5/165 (3.0) |
| Most recent sBP (mmHg) before consent | 131.7±11.2 | 132.9±10.0 |
| Systolic <140 | 154 (76.6) | 151 (74.8) |
| Systolic 140-149 | 37 (18.4) | 39 (19.3) |
| Systolic 150-159 | 10 (5.0) | 12 (5.9) |
| Systolic ≥160 | 0 (0) | 0 (0) |
| Most recent dBP (mmHg) before consent | 83.4± 8.3 | 83.1±8.5 |
| Diastolic BP <90 | 155 (77.1) | 157 (77.7) |
| Diastolic BP 90-99 | 40 (19.9) | 41 (20.3) |
| Diastolic BP 100-109 | 6 (3.0) | 4 (2.0) |
| Diastolic BP ≥110 | 0 (0) | 0 (0) |
| Device used to take BP |  |  |
| Automated device (any type) | 146 (72.6) | 143 (70.8) |
| Aneroid device (manual) | 38 (18.9) | 46 (22.8) |
| Unknown | 17 (8.5) | 13 (6.4) |
| Currently using home BP monitoring | 117 (58.2) | 110 (54.5) |

*BP (blood pressure), dBP (diastolic blood pressure), GA (gestational age), IQR (interquartile range [25^th^ percentile, 75^th^ percentile]), sBP (systolic blood pressure), SD (standard deviation), wks (weeks)*

** Minimisation variable, in addition to study site.*

*ł Number of previous deliveries of fetus at ≥22^+0^ wks, ≥500g birthweight, or a crown-heel length ≥25cm.*

*ǂ Pre-eclampsia was defined as gestational hypertension with proteinuria or one/more relevant end-organ complications [NICE NG133 guidelines].*

*ǁ Defined as in vitro fertilisation with or without intracytoplasmic sperm injection, donor egg, or donor sperm.*

*¶ Responses are not mutually exclusive.*

*** Other antihypertensive therapy in the intervention arm was amlodipine (N=4) and felodopine, and in the control arm, amlodipine (N=4) and hydralazine (N=1).*

**Table G**: Adherence to intervention and birth initiation (N (%) or median [IQR]), with grey shading of information not presented in Table 2.

|  | **Planned early term delivery at 38^+0-3^ wks**  **(N=201)** | **Usual care at term**  **(N=202)** |
| --- | --- | --- |
| **Adherence** |  |  |
| Adherent* | 184 (91.5) | NA |
| Reasons for non-adherence: | (N=17) | - |
| Busy hospital induction or theatre schedules | 11/17 (64.6) | - |
| Womens’ preference | 2/17 (11.8) | - |
| Clinicians’ preference | 1/17 (5.9) | - |
| Spontaneous birth at GA above 38^+3^ wks | 2/17 (11.8) | - |
| Withdrawal from treatment | 1/17 (5.9) | - |
| GA at initiation of birth (induction or no labour) | 38.1 [38.0, 38.3] | 39.0 [38.6, 39.7] |
| GA at birth (all women) | 38.4 [38.3, 38.6] | 39.3 [38.7, 39.9] |
| Method of delivery initiation |  |  |
| Spontaneous onset of labour | 8 (4.0) | 45 (22.3) |
| No labour (elective Caesarean) | 18 (8.9) | 18 (8.9) |
| Induced | 175 (87.1) | 139 (68.8) |
| ARM | 119/175 (68.0) | 96/139 (69.1) |
| ARM only | 39/119 (32.8) | 36/96 (37.5) |
| ARM and prostaglandin | 80/119 (67.2) | 60/96 (62.5) |
| Prostin® E2 vaginal gel | 81/175 (46.3) | 55/139 (39.6) |
| Dinoprostone vaginal insert | 71/175 (40.6) | 50/139 (36.0) |
| Balloon catheter in cervix | 25/175 (14.3) | 18/139 (12.9) |
| Misoprostol | 0/175 (-) | 0/139 (-) |
| Otherł | 40/175 (22.9) | 32/139 (23.0) |
| For women who were induced or had no labourǂ | (N=193) | (N=157) |
| Reasons for decision to deliver |  |  |
| Dictated only by study protocol for allocated group | 152 (78.8) | 26 (16.6) |
| Dictated by study protocol for allocated group & one/more clinical reasons | 15 (7.8) | 2 (1.3) |
| Maternal reasons |  |  |
| Maternal reasons only | 16 (8.3) | 62 (39.5) |
| Maternal hypertension not controlled | 9 (4.7) | 29 (18.5) |
| Maternal pre-eclampsia | 2 (1.0) | 13 (8.3) |
| Other maternal reason(s)ǁ | 10 (5.2) | 47 (29.9) |
| Fetal reasons |  |  |
| Fetal reasons only | 8 (4.2) | 34 (21.7) |
| Poor fetal growth | 3 (1.6) | 9 (5.7) |
| Abnormal fetal heart rate or pattern | 2 (1.0) | 4 (2.6) |
| Abnormal umbilical artery Doppler | 0 | 0 |
| Other fetal reason(s)ǁ | 7 (3.6) | 33 (21.0) |
| Busy hospital induction or theatre schedules** | 12 (6.2) | 6 (3.8) |
| Woman’s preferencełł | 2 (1.0) | 10 (6.4) |
| Clinician's preferencełł | 1 (0.5) | 8 (5.1) |

*ARM (artificial rupture of membranes), BP (blood pressure), GA (gestational age), GDM (gestational diabetes), NA (not applicable), IQR (interquartile range as [25^th^ percentile, 75^th^ percentile]), wks (weeks)*

** Adherence was defined as timing of delivery initiation consistent with the allocated group or if earlier, delivery timing as a result of either spontaneous onset of labour or delivery for clinical need. This was defined as a binary variable only in the intervention group.*

*ł Other methods of induction in the intervention group (N=40) were: oxytocin (N=33), membrane sweep, and an osmotic dilator (N=6); and in the control group (N=32): oxytocin (N=24), membrane sweep, and an osmotic dilator (N=7).*

*ǂ Reasons were not mutually exclusive.*

*ǁ Other maternal reasons in the intervention group (N=10) were: failed induction of labour, prelabour rupture of membranes (N=3), raised serum creatinine, obstetric cholestasis, BP not well-controlled, GDM (on diet) diagnosed after randomisation, and COVID-19 infection; and in the control group (N=47): failed induction (N=3), obstetric cholestasis, prelabour rupture of membranes (N=6), early labour (N=3), poorly-controlled BP (N=10), GDM on metformin and insulin, anxiety (N=5), reached 40 wks (N=2), maternal request (N=4), maternal symptoms (N=4), ‘PIH’ (N=2), low platelet count (N=2), pyrexia, vaginal bleeding, poor glycaemic control (N=2). Other fetal reasons (N=7) in the intervention group were: reduced fetal movements (N=2), severe polydramnios, breech presentation (N=2), and suspected fetal macrosomia (N=2); and in the control group (N=33): reduced fetal movements (N=14), suspected fetal macrosomia (N=8), breech presentation or unstable lie (N=6), cardiotocographic abnormalities (N=2), polyhydramnios (N=2), and reduced amniotic fluid volume.*

*** This refers to requests that were not timely given the volume of requests in the unit.*

*łł When given as only reason for timing of initiation of birth that was not consistent with allocated group.*

**Table H**: Subgroup analyses for maternal and neonatal co-primary outcomes

|  | **Planned early term delivery at 38^+0-3^ wks**  **(N=201)** | **Usual care**  **at term**  **(N=202)** | **Interaction**  **p-value** | **Adjusted risk ratio***  **[95% CI]; p-value** | **Ratio of subgroup effects**  **[95% CI]; p-value** |
| --- | --- | --- | --- | --- | --- |
| **Maternal co-primary outcome:**  **Poor maternal outcomeł** |  |  |  |  |  |
| **Minimisation variables** |  |  |  |  |  |
| Hypertension type (n/n (%)) |  |  | 0.532 |  |  |
| Chronic hypertension | 10/96 (10.4) | 11/99 (11.1) |  | 0.97 [0.45 to 2.08]; 0.941 | 0.74[0.27 to 1.99]; 0.546 |
| Gestational hypertension | 17/105 (16.2) | 13/103 (12.6) |  | 1.32 [0.71 to 2.46]; 0.385 | REF |
| Prior Caesarean (n/n (%)) |  |  | 0.978 |  |  |
| Yes | 1/15 (6.7) | 1/16 (6.3) |  | 1.23 [0.09 to 16.89]; 0.878 | 1.06 [0.07 to 15.26]; 0.966 |
| No | 26/186 (14.0) | 23/186 (12.4) |  | 1.16 [0.71 to 1.88]; 0.551 | REF |
| **Key subgroup variables** |  |  |  |  |  |
| Ethnicity (n/n (%)) |  |  | - |  |  |
| White | 23/157 (14.7) | 20/158 (12.7) |  | Not estimable (model did not converge) | Not estimable (model did not converge) |
| Black | 1/13 (7.7) | 1/17 (5.9) |  |  |  |
| Arab | 0/2 (0) | 0/1 (0) |  |  |  |
| Asian | 0/16 (0) | 0/9 (0) |  |  |  |
| Other or declined to answer | 3/13 (23.1) | 3/17 (17.7) |  |  |  |
| Body mass index (kg/m^2^) (n/n (%)) |  |  | 0.322 |  |  |
| Normal weight (18.5 – 24.9) | 3/30 (10.0) | 5/35 (14.3) |  | 0.92 [0.25 to 3.34]; 0.894 | REF |
| Overweight (25.0 – 29.9) | 5/59 (8.5) | 6/48 (12.5) |  | 0.64 [0.22 to 1.82]; 0.400 | 0.69 [0.13 to 3.69]; 0.669 |
| Obesity (≥30.0) | 19/112 (17.0) | 13/119 (10.9) |  | 1.54 [0.84 to 2.81]; 0.162 | 1.68 [0.40 to 7.03]; 0.480 |
| GDM at randomisation (n/n (%)) |  |  | **-** |  |  |
| Yes | 0/19 (0) | 4/18 (22.2) |  | Not estimable (model did not converge) | Not estimable (model did not converge) |
| No | 27/182 (14.8) | 20/184 (10.9) |  |  |  |
| Antihypertensive at randomisation (n/n (%) |  |  | 0.702 |  |  |
| Yes | 24/156 (15.4) | 21/165 (12.7) |  | 1.22 [0.74 to 2.01]; 0.427 | 1.32 [0.28 to 6.36]; 0.727 |
| No | 3/45 (6.7) | 3/37 (8.1) |  | 0.93 [0.21 to 4.07]; 0.918 | REF |
| **Other subgroup variables** |  |  |  |  |  |
| Prior severe hypertension (index pregnancy, (n/n(%)) |  |  | 0.391 |  |  |
| Yes | 3/17 (17.7) | 6/25 (24.0) |  | 0.76 [0.25 to 2.28]; 0.624 | 0.57 [0.17 to 1.95]; 0.370 |
| No | 24/184 (13.0) | 18/177 (10.2) |  | 1.33 [0.78 to 2.28]; 0.297 | REF |
| Smoking at randomisation (n/n (%)) |  |  | **-** |  |  |
| Yes | 0/10 (0) | 3/13 (23.1) |  | Not estimable (model did not converge) | Not estimable (model did not converge) |
| No | 27/191 (14.1) | 21/189 (11.1) |  |  |  |
| **Neonatal co-primary outcome:**  **Neonatal care unit admission for ≥ 4 hours** |  |  |  |  |  |
| **Minimisation variables** |  |  |  |  |  |
| Hypertension type, n/n (%) |  |  | 0.547 |  |  |
| Chronic hypertension | 9/96 (9.4) | 11/99 (11.1) |  | 0.90¥ [0.40 to 2.05]; 0.802 | 0.60¥ [0.12 to 3.13]; 0.547 |
| Gestational hypertension | 5/105 (4.8) | 3/103 (2.9) |  | 1.49¥ [0.37 to 6.08]; 0.576 | REF |
| Prior Caesarean, n/n (%) |  |  | - |  |  |
| Yes | 0/15 (0) | 0/16 (0) |  | Not estimable (model did not converge) | Not estimable (model did not converge) |
| No | 14/186 (7.5) | 14/186 (7.5) |  |  |  |
| **Key subgroup variables** |  |  |  |  |  |
| Ethnicity (n/n (%)) |  |  | - |  |  |
| White | 12/157 (7.6) | 12/158 (7.6) |  | Not estimable (model did not converge) | Not estimable (model did not converge) |
| Black | 0/13 (0) | 1/17 (5.9) |  |  |  |
| Arab | 0/2 (0) | 0/1 (0) |  |  |  |
| Asian | 1/16 (6.3) | 0/9 (0) |  |  |  |
| Other or declined to answer | 1/13 (7.7) | 1/17 (5.9) |  |  |  |
| Body mass index (kg/m^2^) (n/n (%)) |  |  | 0.733 |  |  |
| Normal weight (18.5 – 24.9) | 1/30 (3.3) | 2/35 (5.7) |  | 0.55¥ [0.05 to 5.51]; 0.611 | REF |
| Overweight (25.0 – 29.9) | 2/59 (3.4) | 2/48 (4.2) |  | 0.77¥ [0.12 to 5.04]; 0.786 | 1.40¥ [0.07 to 27.42]; 0.823 |
| Obesity (≥30.0) | 11/112 (9.8) | 10/119 (8.4) |  | 1.27¥ [0.57 to 2.80]; 0.557 | 2.31¥ [0.20 to 26.61]; 0.502 |
| GDM at randomisation (n/n (%)) |  |  | 0.687 |  |  |
| Yes | 2/19 (10.5) | 1/18 (5.6) |  | 1.61¥ [0.17 to 15.63]; 0.681 | 1.63¥ [0.15 to 17.85]; 0.690 |
| No | 12/182 (6.6) | 13/184 (7.1) |  | 0.99¥ [0.47 to 2.06]; 0.975 | REF |
| Antihypertensive at randomisation (n/n (%)) |  |  | 0.445 |  |  |
| Yes | 9/156 (5.8) | 12/165 (7.3) |  | 0.86¥ [0.38 to 1.95]; 0.715 | 0.51¥ [0.09 to 2.93]; 0.454 |
| No | 5/45 (11.1) | 2/37 (5.4) |  | 1.67¥ [0.37 to 7.64]; 0.508 | REF |
| **Other subgroup variables** |  |  |  |  |  |
| Prior severe hypertension (index pregnancy (n/n(%)) |  |  | - |  |  |
| Yes | 0/17 (0) | 3/25 (12.0) |  | Not estimable (model did not converge) | Not estimable (model did not converge) |
| No | 14/184 (7.6) | 11/177 (6.2) |  |  |  |
| Smoking at randomisation (n/n (%)) |  |  | - |  |  |
| Yes | 1/10 (10.0) | 0/13 (0) |  | Not estimable (model did not converge) | Not estimable (model did not converge) |
| No | 13/191 (6.8) | 14/189 (7.4) |  |  |  |

*CI (confidence interval), GDM (gestational diabetes mellitus), REF (reference), wks (weeks)*

***^*^*** *Risk ratio was adjusted for minimisation variables (centre, hypertension type and prior Caesarean) as categorical covariates, with centre included as a random effect. A value<1 favours planned early term delivery. The p-value was generated from the marginal standardisation model, which followed the mixed effects logistic regression.*

*ł A composite of systolic blood pressure ≥160mmHg, maternal death, and maternal comorbidities adapted from Delphi consensus in hypertensive pregnancy, measured until primary hospital discharge home or 28 days after birth (whichever is earlier).*

*¥ Prior Caesarean removed due to convergence issues.*

**Table I:** Sensitivity analyses for maternal and neonatal co-primary outcomes*

|  | **Planned early term delivery at 38^+0-3^ weeks**  **(N=201)** | **Usual care at term**  **(N=202)** | **Interaction**  **p-value** | **Adjusted risk ratioł**  **[95% CI]; p-value** | **Risk differenceł**  **[95% CI]; p-value** |
| --- | --- | --- | --- | --- | --- |
|  |  |  |  |  |  |
| Poor maternal outcome¶, n (%) | 27 (13.4) | 24 (11.9) | NA | 1.23ǁ [0.7 to 1.98]; 0.383 | 0.03 ǁ [-0.04 to 0.10]; 0.384 |
| Neonatal care unit admission ≥ 4hrs, n (%) | 14 (7.0) | 14 (6.9) | NA | 1.09** [0.54 to 2.18]; 0.818 | 0.01** [-0.05 to 0.06]; 0.818 |
| Caesarean delivery, n (%) | 58 (28.9) | 72 (35.6) | NA | 0.81ǁ [0.61 to 1.07]; 0.144 | -0.07ǁ [-0.16 to 0.02]; 0.141 |
|  |  |  |  |  |  |
| Poor maternal outcome¶, n (%) | 25 (13.0) | 14 (10.2) | NA | 1.32 [0.74 to 2.35]; 0.351 | 0.03 [-0.04 to 0.11];0.340 |
| Neonatal care unit admission ≥ 4hrs, n (%) | 14 (7.3) | 12 (8.8) | NA | 0.90łł [0.44 to 1.86]; 0.777 | -0.01łł [-0.07 to 0.05]; 0.779 |
| Caesarean delivery, n (%) | 55 (28.5) | 47 (34.3) | NA | 0.82 [0.59 to 1.12]; 0.207 | -0.06 [-0.16 to 0.04]; 0.212 |
|  |  |  |  |  |  |
| **Poor maternal outcome**¶, n (%) |  |  | 0.036 |  |  |
| Randomised before change of control group | 19/173 (11.0) | 23/175 (13.1) |  | 0.89 [0.52 to 1.52]; 0.672 | 0.14ǂǂ [0.02 to 1.07]; 0.058 |
| Randomised after change of control group | 8/28 (28.6) | 1/27 (3.7) |  | 6.41 [0.90 to 45.64]; 0.063 | REF |
| **Neonatal care unit admission ≥ 4hrs**, n (%) |  |  | 0.879 |  |  |
| Randomised before change of control group | 12/173 (6.9) | 12/175 (6.9) |  | 1.06łł [0.50 to 2.25]; 0.876 | 1.17ǂǂ [0.16 to 8.61]; 0.878 |
| Randomised after change of control group | 2/28 (7.1) | 2/27 (7.4) |  | 0.91łł [0.14 to 5.71]; 0.918 | REF |
| **Caesarean delivery, n (%)** |  |  | 0.732 |  |  |
| Randomised before change of the control group | 48/173 (27.8) | 61/175 (34.9) |  | 0.79 [0.58 to 1.08]; 0.142 | 0.86ǂǂ [0.41 to 1.82]; 0.696 |
| Randomised after change of the control group | 10/28 (35.7) | 11/27 (40.7) |  | 0.92 [0.47 to 1.81]; 0.809 | REF |

*CI (confidence interval), hrs (hours), ITT (intention-to-treat), RR (risk ratio), RD (risk difference)*

*** *Other pre-specified sensitivity analyses that were not included were: (i) a complier average causal effect analysis, which was not performed due to analytical difficulties in applying this analysis under a marginal standardisation approach; (ii) a tipping point analysis planned to assess the effect of missing responses, but there were none; (iii) a restricted analysis for the neonatal outcome including only liveborns, but there were no stillbirths; (iv) an analysis including in the numerator stillbirths or babies who either died without admission to neonatal care, or died following admission to neonatal care for <4 hours, where newborn death is of a liveborn infant until primary discharge home or within the first 28 days of birth, whichever is earlier, but there were not any stillbirths, or neonatal deaths; and (v) examination of heterogeneity of treatment effect due to the change in the usual care arm, as so few women (14%) were randomised to usual care at term before the trial was stopped .*

***ł*** *RR and RD were adjusted for minimisation variables (centre, hypertension type and prior Caesarean) as categorical covariates, with centre included as a random effect; an RR <1 or an RD <0 favours planned early term delivery.* *The p-value was generated from the marginal standardisation model, which followed the mixed effects logistic regression.*

*ǂ Baseline covariates that were considered potentially unbalanced were:* *BMI, ethnicity, and prior severe hypertension.*

*¶ A composite of systolic blood pressure ≥160mmHg, maternal death, and maternal comorbidities adapted from Delphi consensus in hypertensive pregnancy, measured until primary hospital discharge home or 28 days after birth (whichever is earlier).*

*ǁ For this model, BMI and prior severe hypertension were also included as categorical fixed effects covariates, but ethnicity was not due to convergence issues.*

*** For this model, BMI Body mass index and prior severe hypertension were also included as categorical fixed effects covariates but ethnicity and prior Caesarean were not included due to convergence issues.*

*łł For this model, prior Caesarean was removed due to convergence issues.*

*ǂǂ Ratio of control group change effect.*

**Table J**: Other maternal outcomes (N (%) or median [IQR]), with grey shading of information not presented in Table 3.

| **Outcomes** | **Planned early term delivery at 38^+0-3^ wks**  **(N=201)** | **Usual care at term**  **(N=202)** | **Adjusted risk ratio***  **[95% CI]; p-value** | **Adjusted risk differenceł**  **[95% CI]; p-value** |
| --- | --- | --- | --- | --- |
| Other maternal outcomes |  |  |  |  |
| Pre-eclampsiaǂ | 56 (27.9) | 76 (37.6) | 0.74 [0.56 to 0.98];  0.039 | -0.10 [-0.19 to -0.01]; 0.036 |
| Before birth | 40 | 54 | NA | NA |
| Gestational age (wks) | 38.0 [37.6, 38.2] | 38.3 [37.7, 39.1] | NA | NA |
| After birth | 16 | 22 | NA | NA |
| Components of definition |  |  |  |  |
| Proteinuria | 22 | 32 | NA | NA |
| Headache | 14 | 21 | NA | NA |
| Visual scotomata | 1 | 2 | NA | NA |
| Platelet count <150x10^9^/L | 11 | 9 | NA | NA |
| Elevated serum creatinine of ≥90 micromol/L | 4 | 6 | NA | NA |
| Elevated AST or ALT(>40IU/L) | 7 | 13 | NA | NA |
| Fetal growth restriction (BW <10^th^ centile) | 8 | 12 | NA | NA |
| Elevated AST or ALT (>40 IU/L) | 7 (3.5) | 13 (6.4) | 0.54 [0.22 to 1.33];  0.183 | -0.03 [-0.07 to 0.01]; 0.176 |
| Platelet count <100x10^9^/L | 1 (0.5) | 1 (0.5) | 1.01ǁ [0.06 to 15.97];  0.997 | 0.00003ǁ [-0.01 to 0.01]; 0.997 |
| Mode of birth |  |  |  |  |
| Vaginal (non-instrumental) | 122 (60.7) | 107 (53.0) | 0.84^**^[0.67 to 1.05];  0.121 | -0.08^**^[-0.17 to 0.02]; 0.118 |
| Vaginal birth (instrumental) | 21 (10.4) | 23 (11.4) |  |  |
| Caesarean (no labour) | 39 (19.4) | 34 (16.8) |  |  |
| Caesarean (in labour) | 19 (9.5) | 38 (18.8) |  |  |
| Postpartum haemorrhage | 29 (14.4) | 33 (16.3) | 0.89 [0.57 to 1.40];  0.623 | -0.02 [-0.09 to 0.05]; 0.623 |
| Timing after birth | 0 [0, 0] | 0 [0, 0] | NA | NA |
| Sepsis | 2 (1.0) | 0 (0) | Not estimable | Not estimable |
| Before birth | 1 | 0 | NA | NA |
| After birth | 1 | 0 | NA | NA |
| Intensive therapy unit admission | 3 (1.5) | 0 (0) | Not estimable | Not estimable |
| Before birth | 0 | 0 | NA | NA |
| After birth | 3 | 0 | NA | NA |
| To receive advanced respiratory support | 1 | 0 | NA | NA |
| To support two/more organ systems | 2 | 0 | NA | NA |
| Duration of first admission (days) | 1.0 [1.0, 2.0] | NA | NA | NA |
| Other serious maternal complicationsłł | 1 (0.5) | 1 (0.5) | NA | NA |
| Other neonatal outcomes |  |  |  |  |
| Birthweight centile | 55.8 (27.2) | 57.7 (27.7) | NA | Adjusted mean difference  -1.88¥ [-7.24 to 3.47]; 0.490 |
| Birthweight <10^th^ centile | 8 (4.0) | 12 (5.9) | NA | NA |
| 1-min Apgar | 8.6 (1.3) | 8.3 (1.7) | NA | Adjusted mean difference  0.27ǂǂ [-0.02 to 0.56]; 0.070 |
| 5-min Apgar | 9.5 (0.9) | 9.5 (0.9) | NA | Adjusted mean difference  0.06¥ [-0.11 to 0.23]; 0.485 |
| 5-min Apgar score <7 | 5/199 (2.5) | 5/201 (2.5) | NA | NA |
| 10-min Apgar | 9.8 (0.5) | 9.8 (0.5) | NA | Adjusted mean difference  0.03¥ [-0.08 to 0.14]; 0.614 |
| Respiratory problems |  |  |  |  |
| As indication for high-level neonatal care for ≥4hrs | 4 (2.0) | 7 (3.5) | 0.59ǁ [0.18 to 1.95];  0.390 | -0.02ǁ [-0.05 to 0.02]; 0.384 |
| Requiring interventionǁǁ | 5 (2.5) | 10 (5.0) | 0.51ǁ [0.18 to 1.46];  0.208 | -0.02ǁ [-0.06 to 0.01]; 0.196 |
| Oxygen given | 5 | 10 |  |  |
| Positive pressure ventilation | 1 | 7 |  |  |
| Defined clinically¶¶ | 6 (3.0) | 9 (4.5) | 0.67ǁ [0.24 to 1.86];  0.445 | -0.01ǁ [-0.05 to 0.02];  0.441 |
| Chest x-ray performed | 6 (3.0) | 7 (3.5) | 0.87ǁ [0.30 to 2.54];  0.799 | -0.004ǁ [-0.04 to 0.03];  0.799 |
| Abnormal X-ray, n (%)*** | 1/6 (16.7) | 1/7 (14.3) | NA | NA |
| Hypoxic-ischaemic encephalopathy | 0 (0) | 1 (0.5) | Not estimable | Not estimable |
| Sepsis requiring antibiotics for at least five days | 2 (1.0) | 5 (2.5) | 0.40ǁ [0.08 to 2.04];  0.271 | -0.01ǁ [-0.04 to 0.01]; 0.253 |
| Major operation | 0 (0) | 1 (0.5) | Not estimable | Not estimable |
| Breastfeeding established | 125 (62.2) | 115 (56.9) | 1.09 [0.93 to 1.28];  0.277 | 0.05 [-0.04 to 0.15];  0.276 |
| Exclusive breastfeeding | 90 (45.0) | 87 (43.1) | 1.05 [0.84 to 1.30];  0.689 | 0.02 [-0.08 to 0.12];  0.689 |

*AST (aspartate aminotransferase), ALT (alanine aminotransferase), BW (birthweight), hrs (hours), IQR (interquartile range as [25^th^ percentile, 75^th^ percentile]), SD (standard deviation), wks (weeks)*

* *Risk ratio was adjusted for minimisation variables (centre, hypertension type and prior Caesarean) as categorical covariates, with centre included as a random effect. A value <1 favours planned early term delivery. The p-value was generated from the marginal standardisation model, which followed the mixed effects logistic regression.*

***^ł^*** *Risk difference and mean difference were adjusted for minimisation variables (centre, hypertension type and prior Caesarean) as categorical covariates, with centre included as a random effect. A value <0 favours planned early term delivery.* *The p-value was generated from the marginal standardisation model, which followed the mixed effects logistic regression.*

*ǂ There were none of the following pre-eclampsia criteria met: Glasgow Coma Scale <13, stroke, eclampsia, blindness, clonus, platelet count <50x10^9^/L, disseminated intravascular coagulation, haemolysis, abnormal umbilical artery Doppler, or stillbirth.*

*ǁ For this model, prior Caesarean was removed due to convergence issues.*

*** Instrumental vaginal delivery or Caesarean delivery vs. non-instrumental vaginal delivery.*

*łł Other serious maternal complications in the intervention group (N=1) were seizure likely due to epilepsy (after birth); and in the control group (N=1): poorly-controlled BP (before birth).*

*¥ Mean difference adjusted for minimisation variables (centre, hypertension type and prior Caesarean) as categorical covariates with centre included as a random effect, a value<0 favours planned early term delivery.*

*ǂǂ Mean difference adjusted for minimisation variables (hypertension type and prior Caesarean) as categorical covariates, a value<0 favours planned early term delivery (centre removed from the model due to convergence issues).*

*ǁǁ Respiratory morbidity was defined as the need for supplemental oxygen and/or positive pressure ventilation beyond the initial resuscitation period.*

*¶¶ł Clinical respiratory problem was defined as: transient tachypnoea of newborn (0 in intervention vs. 7 in control groups), meconium aspiration syndrome (0 vs. 1, respectively), pneumonia (0 vs. 0, respectively), pneumothorax/pneumomediastinum (1 vs. 0, respectively), or other (6 vs. 3, respectively).*

**** The abnormal chest x-ray findings were pneumothorax/pneumomediastinum (1 in intervention) and right lung field more hazy than left (1 in control).*

**Table K**: Mean costs in 2020/21 British Pounds

|  | Intervention:  Planned early term birth  (N=201) | | Control:  Usual care at term  (N=202) | | Difference in mean costs (intervention minus control group) (95% CI) | *P* value* |
| --- | --- | --- | --- | --- | --- | --- |
|  | **Mean** | **SD** | **Mean** | **SD** |  |  |
| Outpatient visits | 302.92 | 425.87 | 538.23 | 418.71 | -235.32 (-309.45 to -154.13) | 0.000 |
| Obstetric outpatient | 135.15 | 252.37 | 242.25 | 262.01 | -107.10 (-154.76 to -58.00) | 0.000 |
| Antenatal emergency department | 5.09 | 37.77 | 10.97 | 51.24 | -5.88 (=15.19 to +2.50) | 0.199 |
| Maternal MAU or DAU | 135.20 | 207.54 | 254.76 | 253.14 | -119.56 (-167.90 to -75.77 | 0.000 |
| Community midwife | 27.48 | 85.57 | 30.26 | 73.51 | -2.77 (=19.36 to +12.39) | 0.731 |
| Hospital admissions | 1043.34 | 613.35 | 956.97 | 477.05 | +86.37 (-15.34 to +200.42) | 0.110 |
| Antenatal inpatient admission | 25.66 | 118.83 | 39.72 | 156.72 | -14.06 (-41.49 to +13.72) | 0.319 |
| Maternal inpatient stay for delivery or within 28days after birth | 990.58 | 535.26 | 917.25 | 442.94 | +73.32 (-20.78 to +167.87) | 0.133 |
| Maternal intensive care unit admission | 27.10 | 220.74 | 0.00 | 0.00 | +27.10 (+8.65 to +61.41) | 0.058 |
| Tests of maternal and fetal wellbeing | 156.44 | 170.61 | 259.29 | 188.39 | -102.84 (-136.65 to -67.78) | 0.000 |
| Maternal blood and/or urine tests | 1.48 | 2.27 | 2.73 | 3.13 | -1.26 (-1.81 to -0.72) | 0.000 |
| Fetal ultrasound | 36.12 | 70.46 | 72.75 | 85.56 | -36.62 (-50.77 to -19.78) | 0.000 |
| Fetal cardiotocograph | 79.43 | 84.95 | 110.14 | 81.29 | -30.72 (-48.76 to -15.73) | 0.000 |
| Umbilical artery doppler | 35.49 | 69.22 | 71.47 | 84.06 | -35.98 (-49.87 to -19.43) | 0.000 |
| Chest X-rays | 3.93 | 38.95 | 2.20 | 13.29 | +1.73 (-2.32 to +9.37) | 0.549 |

*CI (confidence interval), DAU (day assessment unit), MAU (medical assessment unit), SD (standard deviation).*

** Costs were compared between groups by regression analysis, with bootstrapped bias-corrected 95% CIs.*

***References***

1. Duffy J, Cairns AE, Richards-Doran D, van 't Hooft J, Gale C, Brown M, et al. A core outcome set for pre-eclampsia research: an international consensus development study. BJOG. 2020;127(12):1516-26. doi: 10.111/471-0528.16319. Epub 2020 Jun 21.
