# Supplementary material for: Determining optimal timing of birth for women with chronic or gestational hypertension at term: The WILL (When to Induce Labour to Limit risk in pregnancy hypertension) randomised trial
Source: PLoS Med. 2024 Nov 26;21(11):e1004481. doi: 10.1371/journal.pmed.1004481 (PMC11593758; doi:10.1371/journal.pmed.1004481)
Supplement: Attachment — Submitted filename: Review WILL study Laura Magee et al. PLOS Medicine 2024.docx [file pmed.1004481.s006.docx]

**REVIEW WILL study PLOS one**

### **Criteria for publication**

In addition to being conducted in accordance with ethical standards and scientifically valid, research published in PLOS Medicine should fulfill each of the following criteria:

1. The research question is an important one to the community of researchers in this general area.

*Yes, it does, it is the largest international trial so far in the field of (early) near term induction of labour in chronic or gestational hypertension in a large multicenter, open-labeled, randomized trial including an economic cost analysis. After a thorough and well described analysis of the date, the authors propose earlier timing of delivery in case of both chronic and gestational hypertension in pregnancy based on the results of the WILL trial.*

2. The results provide a substantial advance over existing knowledge, with clear implications for patient care, public policy, or clinical research agendas.

*Yes, it does, it shows that early induction of labour at 38 + 0-3/7 weeks in case of mild chronic and gestational hypertension is non-inferior to care as usual regarding short term maternal and neonatal outcomes while adding potential benefits by reducing the number of Caesarean sections. The research group is planning an intentional patient data analysis mainly to corroborate the latter, as lower then expected adverse event rates do not justify mounting another randomized trial.*

3. Published together with an Author Summary written for general readers, the article is of interest to clinicians and policymakers who are not specialists in this topic.

*Yes, it changes current treatment protocols regarding chronic or gestational hypertension in pregnancy by adding new information underpinning shared decision making between patient and doctors regarding optimal timing of delivery. Based on the results of this study it has become clear that this proposed change in protocol will potentially reduce number of caesarean sections without an increase in neonatal admissions to the NICU, as well as save costs and reduce outpatient visits in an already overloaded national health system.*

**Review**

- What are the main claims of the paper and how significant are they for the discipline?

*The main claim of the WILL trial is that planned early term birth at 38+0-3 weeks may be the preferred clinical option to do in case of chronic or gestational hypertension in pregnancy. It is the first national and largest international RCT in this field in a diverse study population making the results generalisable to real-world care of women in the United Kingdom. The WILL trial shows that in women with chronic or gestational hypertension who reach term and remain well, planned early term birth at 38+0-3 weeks, vs. usual care at term, averagely results in a six days earlier delivery. However, the expectant management group still requires iatrogenic induction of labour in 78% before spontaneous onset of labour. The authors conclude that planned early term birth at 38+0-3 weeks is non-inferior regarding adverse maternal and fetal/newborn co-primary outcomes compared with usual care at term. Planned early term birth (vs. usual care at term) did not increase (and may potentially reduce) Caesarean. There was a reduction in pre-eclampsia (defined broadly) for the woman, with no evidence of increased health problems for the baby. Finally, healthcare utilisation (e.g., monitoring of maternal-fetal wellbeing and obstetric outpatient visits) and associated costs were lower in the planned early term birth (vs. usual care at term) group, while the direction of costs overall favoured planned early term birth.*

- Are the claims properly placed in the context of the previous literature?

*Yes, comparisons between the current and previous relevant literature have been made appropriately and addressed at. The discussion has however focused on short term outcomes and in that context, the authors state that the 6 days earlier time of birth is not likely to cause harm. The authors refer to the Cochrane Data Base of Systematic Review on Induction of Labour at or beyond 37 weeks of gestation, which results are stated to underpin this assumption. A similar conclusion has (very recently) been made by Grobman based on the ARRIVE trial data, saying that “ Even when the only indication for delivery is the achievement of a full-term gestational age, evidence suggests that multiple different outcomes, including cesarean delivery, hypertensive disorders of pregnancy, neonatal respiratory impairment, and perinatal mortality, are less likely when induction is performed. This information underscores the importance of making the preferences of pregnant individuals for different birth processes and outcomes central to the approach to delivery timing”.*

Middleton P, Shepherd E, Morris J, Crowther CA, Gomersall JC. Induction of labour at or beyond 37 weeks' gestation. Cochrane Database Syst Rev. 2020;7:CD004945.

Grobman WA. The role of labor induction in modern obstetrics. American Journal of Obstetrics and Gynaecology. 2024; 230(3), S662-S66. Doi.org/10.1016/j.ajog.2022.03.019.

- Have the authors treated the literature fairly?

*Not fully, as the authors do not mention any potential harmful long term effects on maternal or neonatal outcome, while reference to current literature (The Cochrane Data Base of Systematic Review on Induction of Labour at or beyond 37 weeks’ of gestation) clearly states that data on long term effects looking at neurodevelopment at childhood are still lacking. The decision to induce labour is always a balance of risks and consequences for child and mother on the short as well as on the long term. In a relatively uncomplicated pregnancy before 40 weeks such as could be interpreted as described in the WILL study, long term effects of induction of labour on neurocognitive development should at least be discussed in the light of the existing literature.*

Bentley JP, Roberts CL, Bowen JR, Martin AJ, Morris JM, Nassar N. Planned Birth Before 39 Weeks and Child Development: A Population-Based Study. Pediatrics. 2016 Dec;138(6):e20162002. doi: 10.1542/peds.2016-2002.

Burger RJ, Mol BW, Ganzevoort W, et al. Offspring school performance at age 12 after induction of labor vs non-intervention at term: A linked cohort study. *Acta Obstet Gynecol Scand*. 2023; 102: 486-495. doi:[10.1111/aogs.14520](https://doi.org/10.1111/aogs.14520)

Davies-Tuck M., Wallace EM, Homer CSE. Why ARRIVE should not thrive in Australia, Women and Birth. 2018, 31(5), 339-340, doi.org/10.1016/j.wombi.2018.08.168.

Grobman WA. The role of labor induction in modern obstetrics. American Journal of Obstetrics and Gynaecology. 2024; 230(3), S662-S66. Doi.org/10.1016/j.ajog.2022.03.019.

Odd D, Glover Williams A, Winter C, Draycott T. Associations between early term and late/post term infants and development of epilepsy: A cohort study. PLoS One. 2018 Dec 31;13(12):e0210181. doi: 10.1371/journal.pone.0210181.

- Do the data and analyses fully support the claims? If not, what other evidence is required?

*The claims stated can be concluded from the current study, even though the required number of inclusions has not been achieved.*

***Note for the editor: please perform additional check statistical validity.***

- *PLOS Medicine* encourages authors to publish detailed methods as supporting information online. Do any particular methods used in the manuscript warrant such publication? If a protocol is already provided, for example for a randomized controlled trial, are there any important deviations from it?

*Yes, there is a study protocol available and there have been important deviations from it as the study has been stopped prematurely by the sponsor before including the desired number per group. Also, the primary stated desired gestational age of delivery in the expected management group changed from term to 39 weeks of gestation in the final phase of randomisation (17%). (On 11/August/2022 (after randomisation of 348 women), the control group was changed from ‘expectant care until at least 40+0 weeks’ to ‘usual care at term’, to reflect practice change related to timed birth in other populations(13, 14), the COVID-19 pandemic(15), and draft national labour induction guidance suggesting timed birth at 39 weeks may be appropriate for women at increased risk of term complications)*

If so, have the authors explained adequately why the deviations occurred? *Yes, the deviations and its potential effects have been adequately explained in the article.*

- Is this paper outstanding in its discipline? If yes, what makes it outstanding? If not, why not?

*Yes, it is outstanding as it adds novel insights to the current knowledge on optimal timing of delivery in case of constitutional or gestational hypertension at near term in the context of the National Health System of the UK.*

- Does the study conform to any [relevant guidelines](https://journals.plos.org/plosmedicine/s/best-practices-in-research-reporting#loc-reporting-guidelines-for-specific-study-types) such as CONSORT, MIAME, QUORUM, STROBE, and the [Fort Lauderdale agreement](https://www.sanger.ac.uk/wp-content/uploads/fortlauderdalereport.pdf)?

*Yes, a Consort checklist table has been provided.*

- Are details of the methodology sufficient to allow the experiments to be reproduced?

*Yes, there is sufficient detail to allow reproduction.*

- Is any software created by the authors freely available?

*Not applicable*

- Is the manuscript well organized and written clearly enough to be accessible to non-specialists?

*Yes, it is well written and easy to read, in such a manner that non-specialists can sufficiently follow the train of thoughts of the authors in conducting the trial, understand the suggested adjustments of the study protocol and the effect thereof on the robustness of the data.*
