# Supplementary material for: Determining optimal timing of birth for women with chronic or gestational hypertension at term: The WILL (When to Induce Labour to Limit risk in pregnancy hypertension) randomised trial
Source: PLoS Med. 2024 Nov 26;21(11):e1004481. doi: 10.1371/journal.pmed.1004481 (PMC11593758; doi:10.1371/journal.pmed.1004481)
Supplement: Attachment — Submitted filename: WILL - RESPONSE R1 - 20240625-final.docx [file pmed.1004481.s007.docx]

Please find below our Response, in standard font, with editors’ and reviewers’ comment in italics.

**COMMENTS FROM EDITORS**

*One of the subject reviewers has commented in their review: “It is unclear to the non-statistician reader how the statement “The lower limit of the aRD 95% CI did not include the pre-specified effect size of an 8 percentage point reduction’ translates to their final conclusion ‘As such, despite our lower-than-planned sample size, we were not underpowered to find target differences in our co-primary outcomes.’” Please revise this statement to substantiate your conclusion relating to underpower, in a manner t32hat will be well understood amongst a general readership.*

We have reworded the text to improve clarify, as follows: “The 95% CI of the aRD (-0.05 to +0.09) did not include the pre-specified effect size of -0.08 (which corresponds to a ‘poor maternal’ outcome event rate being 8% lower in the intervention vs. control groups I absolute terms) (page 21, lines 291-3).

*It is important that the upper and lower boundaries of the non-inferiority margins selected are made clear and justified in the manuscript.*

To further clarify, we have added the phrase, “(i.e., the upper bound of the 95% CI is <8%)” to the following statement: “For the neonatal co-primary outcome, we estimated 540/group would have 88% power to detect a non-inferiority margin of 8%, assuming a control group rate of 23% (i.e., the upper bound of the 95% CI is <8%), two-sided alpha=0.05, and 90% power to detect a 10% decrease in Caesarean[(10)]…” (page 9, lines 194-7).

**COMMENTS FROM REVIEWERS**

**REVIEWER #1**

*Criteria for publication*

*In addition to being conducted in accordance with ethical standards and scientifically valid, research published in PLOS Medicine should fulfill each of the following criteria:*

*1. The research question is an important one to the community of researchers in this general area.*

*Yes, it does, it is the largest international trial so far in the field of (early) near term induction of labour in chronic or gestational hypertension in a large multicenter, open-labeled, randomized trial including an economic cost analysis. After a thorough and well described analysis of the date, the authors propose earlier timing of delivery in case of both chronic and gestational hypertension in pregnancy based on the results of the WILL trial.*

Thank you for this positive summary.

*2. The results provide a substantial advance over existing knowledge, with clear implications for patient care, public policy, or clinical research agendas.*

*Yes, it does, it shows that early induction of labour at 38 + 0-3/7 weeks in case of mild chronic and gestational hypertension is non-inferior to care as usual regarding short term maternal and neonatal outcomes while adding potential benefits by reducing the number of Caesarean sections. The research group is planning an intentional patient data analysis mainly to corroborate the latter, as lower then expected adverse event rates do not justify mounting another randomized trial.*

Thank you for this positive summary. May we suggest

*3. Published together with an Author Summary written for general readers, the article is of interest to clinicians and policymakers who are not specialists in this topic.*

*Yes, it changes current treatment protocols regarding chronic or gestational hypertension in pregnancy by adding new information underpinning shared decision making between patient and doctors regarding optimal timing of delivery. Based on the results of this study it has become clear that this proposed change in protocol will potentially reduce number of caesarean sections without an increase in neonatal admissions to the NICU, as well as save costs and reduce outpatient visits in an already overloaded national health system.*

Thank you for this positive summary.

*Review*

** What are the main claims of the paper and how significant are they for the discipline?*

*The main claim of the WILL trial is that planned early term birth at 38+0-3 weeks may be the preferred clinical option to do in case of chronic or gestational hypertension in pregnancy. It is the first national and largest international RCT in this field in a diverse study population making the results generalisable to real-world care of women in the United Kingdom. The WILL trial shows that in women with chronic or gestational hypertension who reach term and remain well, planned early term birth at 38+0-3 weeks, vs. usual care at term, averagely results in a six days earlier delivery. However, the expectant management group still requires iatrogenic induction of labour in 78% before spontaneous onset of labour. The authors conclude that planned early term birth at 38+0-3 weeks is non-inferior regarding adverse maternal and fetal/newborn co-primary outcomes compared with usual care at term. Planned early term birth (vs. usual care at term) did not increase (and may potentially reduce) Caesarean. There was a reduction in pre-eclampsia (defined broadly) for the woman, with no evidence of increased health problems for the baby. Finally, healthcare utilisation (e.g., monitoring of maternal-fetal wellbeing and obstetric outpatient visits) and associated costs were lower in the planned early term birth (vs. usual care at term) group, while the direction of costs overall favoured planned early term birth.*

Thank you for this positive summary.

** Are the claims properly placed in the context of the previous literature?*

*Yes, comparisons between the current and previous relevant literature have been made appropriately and addressed at. The discussion has however focused on short term outcomes and in that context, the authors state that the 6 days earlier time of birth is not likely to cause harm. The authors refer to the Cochrane Data Base of Systematic Review on Induction of Labour at or beyond 37 weeks of gestation, which results are stated to underpin this assumption. A similar conclusion has (very recently) been made by Grobman based on the ARRIVE trial data, saying that " Even when the only indication for delivery is the achievement of a full-term gestational age, evidence suggests that multiple different outcomes, including cesarean delivery, hypertensive disorders of pregnancy, neonatal respiratory impairment, and perinatal mortality, are less likely when induction is performed. This information underscores the importance of making the preferences of pregnant individuals for different birth processes and outcomes central to the approach to delivery timing".*

*Middleton P, Shepherd E, Morris J, Crowther CA, Gomersall JC. Induction of labour at or beyond 37 weeks' gestation. Cochrane Database Syst Rev. 2020;7:CD004945.*

*Grobman WA. The role of labor induction in modern obstetrics. American Journal of Obstetrics and Gynaecology. 2024; 230(3), S662-S66. Doi.org/10.1016/j.ajog.2022.03.019.*

Thank you for this positive summary.

** Have the authors treated the literature fairly?*

*Not fully, as the authors do not mention any potential harmful long term effects on maternal or neonatal outcome, while reference to current literature (The Cochrane Data Base of Systematic Review on Induction of Labour at or beyond 37 weeks' of gestation) clearly states that data on long term effects looking at neurodevelopment at childhood are still lacking. The decision to induce labour is always a balance of risks and consequences for child and mother on the short as well as on the long term. In a relatively uncomplicated pregnancy before 40 weeks such as could be interpreted as described in the WILL study, long term effects of induction of labour on neurocognitive development should at least be discussed in the light of the existing literature.*

*Bentley JP, Roberts CL, Bowen JR, Martin AJ, Morris JM, Nassar N. Planned Birth Before 39 Weeks and Child Development: A Population-Based Study. Pediatrics. 2016 Dec;138(6):e20162002. doi: 10.1542/peds.2016-2002.*

*Burger RJ, Mol BW, Ganzevoort W, et al. Offspring school performance at age 12 after induction of labor vs non-intervention at term: A linked cohort study. Acta Obstet Gynecol Scand. 2023; 102: 486-495. doi:10.1111/aogs.14520 Davies-Tuck M., Wallace EM, Homer CSE. Why ARRIVE should not thrive in Australia, Women and Birth. 2018, 31(5), 339-340, doi.org/10.1016/j.wombi.2018.08.168.*

*Grobman WA. The role of labor induction in modern obstetrics. American Journal of Obstetrics and Gynaecology. 2024; 230(3), S662-S66. Doi.org/10.1016/j.ajog.2022.03.019.*

*Odd D, Glover Williams A, Winter C, Draycott T. Associations between early term and late/post term infants and development of epilepsy: A cohort study. PLoS One. 2018 Dec 31;13(12):e0210181. doi: 10.1371/journal.pone.0210181.*

Thank you for raising this important caveat about long-term outcomes. To our Discussion/Interpretation, we have added the following statement: “Also, observational data have raised concerns that in the general population, across the whole range of gestational at birth, gestational age has a strong, dose-dependent relationship with special educational needs, including mild learning disabilities such as dyslexia and attention deficit hyperactivity disorder[(32)]. While data from non-randomised comparisons of labour induction or expectant care at term have been reassuring with regards to neurodevelopmental outcomes[(33, 34)], condition-specific data are needed. WILL participants were asked for consent for collection of routinely-collected health data, including those measuring school performance” (page 36, lines 419-26).

** Do the data and analyses fully support the claims? If not, what other evidence is required?*

*The claims stated can be concluded from the current study, even though the required number of inclusions has not been achieved.*

We agree, thank you.

** PLOS Medicine encourages authors to publish detailed methods as supporting information online. Do any particular methods used in the manuscript warrant such publication? If a protocol is already provided, for example for a randomized controlled trial, are there any important deviations from it?*

*Yes, there is a study protocol available and there have been important deviations from it as the study has been stopped prematurely by the sponsor before including the desired number per group. Also, the primary stated desired gestational age of delivery in the expected management group changed from term to 39 weeks of gestation in the final phase of randomisation (17%). (On 11/August/2022 (after randomisation of 348 women), the control group was changed from 'expectant care until at least 40+0 weeks' to 'usual care at term', to reflect practice change related to timed birth in other populations(13, 14), the COVID-19 pandemic(15), and draft national labour induction guidance suggesting timed birth at 39 weeks may be appropriate for women at increased risk of term complications)*

Thank you for this summary with which we agree, but would like to provide one clarification. The control arm of the trial changed from ‘expectant care until at least 40+0 weeks’ gestation’, to usual care at term. 40+0 weeks is a woman’s expected date of delivery, but ‘term’ gestational age is strictly defined as 37+0 to 41+6 weeks gestational age.

Our study protocol has been published [Kirkham K et al. WILL (When to induce labour to limit risk in pregnancy hypertension): Protocol for a multicentre randomised trial. Pregnancy Hypertens 2023;32:35-42).

*If so, have the authors explained adequately why the deviations occurred?*

*Yes, the deviations and its potential effects have been adequately explained in the article.*

Thank you.

** Is this paper outstanding in its discipline? If yes, what makes it outstanding? If not, why not?*

*Yes, it is outstanding as it adds novel insights to the current knowledge on optimal timing of delivery in case of constitutional or gestational hypertension at near term in the context of the National Health System of the UK.*

Thank you.

** Does the study conform to any relevant guidelines such as CONSORT, MIAME, QUORUM, STROBE, and the Fort Lauderdale agreement?*

*Yes, a Consort checklist table has been provided.*

We agree.

** Are details of the methodology sufficient to allow the experiments to be reproduced?*

*Yes, there is sufficient detail to allow reproduction.*

Thank you.

** Is any software created by the authors freely available?*

*Not applicable*

We concur.

** Is the manuscript well organized and written clearly enough to be accessible to non-specialists?*

*Yes, it is well written and easy to read, in such a manner that non-specialists can sufficiently follow the train of thoughts of the authors in conducting the trial, understand the suggested adjustments of the study protocol and the effect thereof on the robustness of the data.*

Thank you.

**REVIEWER #2** (James Wason)

*This paper reports a RCT evaluating an intervention of inducing labour early in women with hypertension. Due to the pandemic the trial was stopped early, although a sample size of 400 was recruited, meaning this trial provides useful data. The paper reported the trial very well and I only had a few minor comments below.*

*1. Abstract ‘key secondary outcome was Caesarean’ – should this be ‘proportion of births that were Caesarean’?*

We have reworded to say “Caesarean births” (page 3, lines 60).

*2. Abstract: I note that one secondary outcome is reported in the abstract although there were multiple secondary outcomes. Generally it’s considered good reporting practice to report all secondary endpoints or none in the abstract. Here the Caesarean outcome is described as a key secondary one so maybe it’s appropriate to report it. However given the abstract could have additional words, it might be useful to add something like ‘No other secondary outcomes showed notable differences between arms’.*

Given the opportunity to provide the reader with more detail, we have added other secondary outcomes that were significantly different between groups: “With regards to other secondary outcomes In the intervention (vs. control) group, pre-eclampsia incidence was lower (56, 28% vs. 76, 38%, respectively; aRR 0.74, 95% CI 0.56-0.98), as were absolute mean costs (£-407.80, 95% CI -793.47 to +39.55), related to significantly lower mean costs for outpatient visits (£-235.32, 95% CI -309.45 to -154.13) and tests of maternal-fetal wellbeing (£-102.84, 95% CI -136.65 to -67.78)” (page 3-4, lines 70-4).

*3. Page 6, line 125: could the random element be provided?*

Yes, of course. The following statement has been added: “A ‘random element’ was included in the minimisation algorithm, so that each woman had a probability of 20%, of being randomised to the opposite intervention that they would have otherwise received”. (page 7, lines 158-60)

*4. Page 7, line 159: presumably the alpha was two-sided, could this be added? Could the alpha level for the non-inferiority outcome be added?*

The alpha for all statistical analyses (superiority and non-inferiority outcome) was 0.05 (consistent with our presentation of 95% CI) and two-sided. This has now been clarified in the Methods (page 9, lines 192-8).

*5. Page 7, it was not clear to me whether (in the original plan) both co-primary outcomes would have to be significant to recommend the intervention, or just one. Presumably the former given there was no multiple testing correction planned?*

Prior to the outcomes being known, whether or not the intervention would be recommended was dependant on the totality of the evidence, which included the key secondary (Caesarean births) and other secondary outcomes, including costs. As such, we have never planned to undertake multiple testing correction, as per our protocol: “Estimates of between-group differences will be presented with two-sided 95% confidence intervals (CIs), adjusted for the minimisation variables [24]. No adjustment for multiple comparisons will be made” [Kirkham K et al. WILL (When to induce labour to limit risk in pregnancy hypertension): Protocol for a multicentre randomised trial. Pregnancy Hypertens 2023;32:35-42].

*6. Results: I was surprised that no p-values (at least for the co-primary endpoints) were given, with the trial having pre-specified hypotheses. Perhaps the authors feel it is not appropriate given the trial didn’t meet its enrolment criteria. Personally I would recommend they are given for the blue rows in Table 2, but if the authors feel strongly it’s not warranted (especially if the SAP stated it wouldn’t be done) then I’ll not insist.*

We planned not to present p-values, as per our published protocol [Kirkham K et al. WILL (When to induce labour to limit risk in pregnancy hypertension): Protocol for a multicentre randomised trial. Pregnancy Hypertens 2023;32:35-42]. We made this decision so the interpretation of results would depend on the width of 95% CI and whether they included clinically important differences. We understand this to be a common request from journals.

**REVIEWER #3**

*This is a non -masked randomized trial in women with non-severe GHTN or CHTN enrolled at 36-37 weeks and allocated to either planned delivery at 38 weeks or until at least 40 weeks' gestation. There are 2 co-primary outcomes; One is composite poor maternal and one neonatal morbidity. The planned sample size was 1080 participants: 540/ each arm. Unfortunately, the trial was stopped prematurely by the Funding agency after 403 (< 40%) subjects were randomized due to poor recruitment. The authors conclude that on balance, planned early term birth at 38 weeks may be the preferred clinical option for women with CHTN or GHTN.*

We agree with this summary.

*There are major issues with the manuscript:*

We believe that we have presented a robust response to the reviewer’s concerns such that if s/he may still disagree with us on some of these points, s/he may agree that there is a spectrum of opinion on the topic. This is acknowledged by our previous Discussion/Limitations section, which highlights that we: reached 37% of our target sample size, recruited women with chronic or gestational hypertension, and revised our control arm slightly late in recruitment (page 37, lines 432-38).

*1. Women with GHTN have a different pathophysiology than those with CHTN. Therefore, it was inappropriate to lump them together unless they had adequate sample size for each group with a planned secondary analysis.*

While the pathophysiology of the underlying hypertension may be different in women with chronic vs. gestational hypertension, we feel that combining them is justified. First, there is precedent. They were previously combined for in the international CHIPS trial of differential blood pressure control in pregnancy [Magee LA et al. N Engl J Med 2015;372(5):407-17], with no evidence of subgroup effects by hypertension type. Importantly, similar results were seen in the American CHAP trial when the study population was restricted to women with chronic hypertension [Tita AT et al. N Engl J Med 2022;386(19):1781-1792]. Second, women with either chronic or gestational hypertension are at increased risk of progression to pre-eclampsia at term gestational age, and so the clinical question to be addressed is the same (i.e., should one proceed with timed birth before pre-eclampsia or other problems develop, or should wait for complications to occur and then react with induction/Caesarean). Peer review has supported combining types of pregnancy hypertension in such circumstances, including the landmark HYPITAT I [Koopmans CM et al. Induction of labour versus expectant monitoring for gestational hypertension or mild pre-eclampsia after 36 weeks' gestation (HYPITAT): a multicentre, open-label randomised controlled trial. Lancet 2009;374(9694):979-88] and II trials [Broekhuijsen K et al. Lancet 2015;385(9986):2492-501] of timed birth in pregnancy hypertension, and the ongoing giantPANDA trial of nifedipine vs. labetalol [ISRCTN reference number 12792616].

We had listed our decision to combine chronic and gestational hypertension (with minimisation of randomisation by hypertension type) as a potential limitation, with qualification, as follows: “We included women with either chronic or gestational hypertension, but results for the co-primary outcomes were similar by hypertension type” (page 37, lines 434-6).

*2. Given, the premature termination of the trial, the conclusions in the abstract and manuscript should be changed to state that it the optimal timing of delivery for either group of women remains unknown.*

We have been open about the nature and reasons for premature withdrawal of funding by our Sponsor and a mandated end to the trial. What the reviewer suggests implies that the information provided by this trial is not relevant to clinical practice. While we would not suggest that our results are definitive, we do believe that they should inform practice until further information becomes available. Given the low adverse event rates in this era of blood pressure control – internationally (CHIPS trial) and in the United States (CHAP trial) – we suggest that further information may not be forthcoming in the near future; this is stated at the end of the Discussion/Interpretation (page 36, lines 416-9).

*3. It was inappropriate to change timing of delivery for the usual care group in the middle of the trial.*

As described in the Methods, “the control group was changed from ‘expectant care until at least 40^+0^ weeks’ to ‘usual care at term’, to reflect practice change related to timed birth in other populations(13, 14), the COVID-19 pandemic(15), and draft national labour induction guidance suggesting timed birth at 39 weeks may be appropriate for women at increased risk of term complications(16)” (page 7, lines 164-8). The change was approved by all WILL Oversight Committees, our funder, and the relevant Research Ethics Board. This suggests that while there may be a spectrum of opinion on this issue, our decision was reasonable.

*4. The number of women with GHTN in this trial was only 208 (105 v.103), and for CHTN it was only 195 (96 v. 99). Therefore, it is difficult to draw any conclusions or recommendations from this trial, particularly since the HYPITAT trial had more women with GHTN than this trial, and recommendation is to do delivery at 37 weeks' gestation.*

The differences between WILL and HYPITAT has been a source of confusion. WILL recruited women who developed gestational hypertension preterm, but who remained well at term, and randomised them to planned birth at 38+0-3 weeks (vs. expectant care). In contrast, HYPITAT recruited women who developed gestational hypertension from 36+0 weeks and randomised them to immediate birth (vs. expectant care). In HYPITAT, 188/756 women were recruited at 37+0-6 weeks and as it is not known what proportion of these women had gestational hypertension (which was 65% overall), it is likely that no more than 122 women in HYPITAT would have been eligible for WILL.

*5. Almost 80% of enrolled subjects were receiving antihypertensive medications at enrollment, yet the authors considered these women to have non-severe hypertension. Thus, the authors should state that their findings apply only to women on medications, and does not address non- severe patients without medications.*

Women with non-severe hypertension are those with BP ≥140/90mmHg by all international standards in pregnancy. Internationally, such women are now recommended to receive antihypertensive therapy to reach a target BP of at least 135/85mmHg [Magee LA et al. The 2021 International Society for the Study of Hypertension in Pregnancy classification, diagnosis & management recommendations for international practice. Pregnancy Hypertens 2022;27:148-69]; therefore, our study results will apply to those receiving evidence-based care. Our Discussion highlights that our findings “may be due to WILL being undertaken in the current era of good BP control” (page 35, lines 391-2). We still regard women with antihypertensive treated non-severe hypertension to have the condition of hypertension, in the same way as we regard those with diabetes to remain so afflicted even when their hyperglycaemia is treated with oral agents or insulin.

**REVIEWER #4**

*This is an admirable study which aimed to answer an important and highly relevant question in clinical practice - the timing of birth management of this group of women who do not have preeclampsia is much more unclear than for those with preeclampsia. It is disappointing for the trial team especially that the trial was stopped early by the funder, it must have been extremely difficult conducting a trial of this scale during the pandemic anyhow.*

Thank you for your generous and sympathetic thoughts.

*Nil concerns about general methods - outlined in great detail and all seem appropriate for the intended analyses - however conclusions drawn from the findings of the primary analysis (8% aRD met) in the context of significantly smaller sample size needs further evaluation and discussion. It is unclear to the non-statistician reader how the statement "The lower limit of the aRD 95% CI did not include the pre-specified effect size of an 8 percentage point reduction' translates to their final conclusion 'As such, despite our lower-than-planned sample size, we were not 267 underpowered to find target differences in our co-primary outcomes.'*

This is the comment highlighted in the Editor’s comments. Please see our Response on page 1 of this response.

*The limitations of the trial are documented openly however, a post-hoc power calculation would be useful to ascertain the significance of the actual trial findings. The recruitment of only 37% of the target should be included in the abstract, this is a major limitation.*

Rather than undertaking a post-hoc power calculation, we present 95% CI for our trial findings, so that the reader can interpret the between-group differences consistent with the data.

We have inserted into the abstract that 403 recruits represents 37.3% of target recruitment (page 3, lines 60-1).

*The Discussion is comprehensive and the trial findings are well-placed in the context of current literature. The increase in development of preeclampsia (0.74 (0.56 to 0.98) in the control group has not been pulled out at all but should be discussed - this seems like a major finding.*

Thank you for highlighting this. Another reviewer has highlighted that we should add other secondary outcomes to the abstract, which we have now done (as per our Response to Reviewer #2, Comment 2, above).

We have added the following to the Results: “…the absolute reduction translates into a number-needed-to-treat for benefit (NNTB) of 10” (page 22, lines 314-5), and in the Discussion/Summary of findings (page 34, line 367).

In the Discussion/Interpretation, we have added further to the statement about pre-eclampsia, as follows: “…planned early term birth is associated with a clinically important lower risk of progression to pre-eclampsia, albeit potentially, associated with a smaller, increased risk of transfusion; this stands alone as an intervention that reduces the risk of progression to pre-eclampsia at term in women with chronic or gestational hypertension, similar to development of *de novo* pre-eclampsia in the ARRIVE trial of timed birth at term for low-risk nulliparous women(13) (page 36, lines 407-11).”

**GENERAL EDITORIAL REQUESTS**

*Please ensure that the paper adheres to the PLOS Data Availability Policy.*

We have provided a Data Availability Statement after the Conclusions, as follows: “Requests for data should be directed to the corresponding author. Requests will be assessed for scientific rigour before being granted. Data will be anonymised and securely transferred. A data-sharing agreement might be required” (page 38, lines 458-61).

We are unable to make participant-level trial data publicly available, as participants did not consent to this and this could compromise patient privacy. We are, however, able to share non-identifiable participant-level data upon request, with an appropriate data sharing agreement in place.

*We expect all researchers with submissions to PLOS in which author-generated code underpins the findings in the manuscript to make all author-generated code available without restrictions upon publication of the work. In cases where code is central to the manuscript, we may require the code to be made available as a condition of publication. Authors are responsible for ensuring that the code is reusable and well documented. Please make any custom code available, either as part of your data deposition or as a supplementary file. Please add a sentence to your data availability statement regarding any code used in the study; eg “The code used in the analysis is available from Github [URL] and archived in Zenodo [DOI link]” Please review our guidelines at* [*https://eur03.safelinks.protection.outlook.com/?url=https%3A%2F%2Fjournals.plos.org%2Fplosmedicine%2Fs%2Fmaterials-software-and-code-sharing&data=05%7C02%7Claura.a.magee%40kcl.ac.uk%7Cdd3a317c29a8428472e708dc84a58e2d%7C8370cf1416f34c16b83c724071654356%7C0%7C0%7C638531096542832517%7CUnknown%7CTWFpbGZsb3d8eyJWIjoiMC4wLjAwMDAiLCJQIjoiV2luMzIiLCJBTiI6Ik1haWwiLCJXVCI6Mn0%3D%7C0%7C%7C%7C&sdata=UqU6v9lwpit2xtD79u0ZTWw7EsJ%2B8yx1Wq8c3bLrMIs%3D&reserved=0*](https://eur03.safelinks.protection.outlook.com/?url=https%3A%2F%2Fjournals.plos.org%2Fplosmedicine%2Fs%2Fmaterials-software-and-code-sharing&data=05%7C02%7Claura.a.magee%40kcl.ac.uk%7Cdd3a317c29a8428472e708dc84a58e2d%7C8370cf1416f34c16b83c724071654356%7C0%7C0%7C638531096542832517%7CUnknown%7CTWFpbGZsb3d8eyJWIjoiMC4wLjAwMDAiLCJQIjoiV2luMzIiLCJBTiI6Ik1haWwiLCJXVCI6Mn0%3D%7C0%7C%7C%7C&sdata=UqU6v9lwpit2xtD79u0ZTWw7EsJ%2B8yx1Wq8c3bLrMIs%3D&reserved=0) *and ensure that your code is shared in a way that follows best practice and facilitates reproducibility and reuse. Because Github depositions can be readily changed or deleted, we encourage you to make a permanent DOI’d copy (e.g. in Zenodo) and provide the URL.*

Sharing the analytic code, which is not central to the manuscript, would require that the participant-level data be made available too. Please see our Data Availability Statement above.

*We ask every co-author listed on the manuscript to fill in a contributing author statement, making sure to declare all competing interest*.

All authors have completed COI statements which have been uploaded. That we have no competing interests to declare is stated in a Declarations statement, as per the original manuscript (just before the References) (page 38, lines 463-66).

*Please upload any figures associated with your paper as individual TIF or EPS files with 300dpi resolution at resubmission.*

Figure 1 has been uploaded in the format requested.

*Please provide the name(s) of the institutional review board(s) that provided ethical approval.*

This has been added as an Ethical Approval statement: “The trial was approved by the NHS Health Research Authority London Fulham Research Ethics Committee (reference 18/LO/2033)” (page 37, lines 449-51).

**FORMATTING - GENERAL**

*Abstract: Please structure your abstract using the PLOS Medicine headings (Background, Methods and Findings, Conclusions). Please combine the Methods and Findings sections into one section, “Methods and findings”.*

The abstract has been formatted as requested.

*At this stage, we ask that you include a short, non-technical Author Summary of your research to make findings accessible to a wide audience that includes both scientists and non-scientists.*

The Author Summary has been inserted as requested, following the Abstract (page 4-5, lines 86-113).

*Please express the main results with 95% CIs as well as p values. When reporting p values please report as p<0.001 and where higher as the exact p value p=0.002, for example. Throughout, suggest reporting statistical information as follows to improve clarity for the reader “22% (95% CI [13%,28%]; p</=)”. Please be sure to define all numerical values at first use.*

In our SAP, we stated that, “Null hypotheses have not been stated as we are not presenting p-values or discussing statistical significance, but will focus on the treatment effect measures and 95% Cis”. However, we recognise that this conflicts with PLoS Med policy. As such, we have added the p values to all tables, main and supplementary.

*Please include page numbers and line numbers in the manuscript file. Use continuous line numbers (do not restart the numbering on each page).*

The formatting has been done as advised, throughout.

*Please cite the reference numbers in square brackets. Citations should precede punctuation.*

The referencing has been done as requested throughout.

**FIGURES AND TABLES**

*Please provide titles and legends for all figures and tables (including those in Supporting Information files).*

This has been done as advised.

*Please define all abbreviations used in each figure/table (including those in Supporting Information files).*

This has been done as advised.

*Please consider avoiding the use of red and green in order to make your figure more accessible to those with color blindness.*

This has been done as advised.

**SUPPLEMENTARY MATERIAL**

*Please note that supplementary material will be posted as supplied by the authors. Therefore, please amend it according to the relevant comment.*

This has been done as advised.

**REFERENCES**

*PLOS uses the numbered citation (citation-sequence) method and first six authors, et al. Please ensure that journal name abbreviations match those found in the National Center for Biotechnology Information (NCBI) databases and are appropriately formatted and capitalised.*

This has been done as advised.

*Where website addresses are cited, please include the complete URL and specify the date of access (e.g. [accessed: 12/06/2023]).*

This has been done as advised.

*PLOS Medicine requires that all trials be prospectively registered in one of registries recognized by WHO. Please ensure that study registration details are included in the Methods section.*

This has been done as advised (page 4, line 79; page 7, lines 140-141).

*Please structure the Methods section using the following sub-headings: Study design and participants,* *Randomization and masking, Procedures, Outcomes, Statistical analysis.*

This has been done as advised.

*Please ensure that all prespecified outcomes (primary, secondary, and exploratory) are listed in the Methods/Outcomes section and indicate whether there are outcomes that are not presented in the current report.*

This has been done as advised (page 8, line 189-90).

*Please specify the dates (Month Day, Year) during which study enrollment and follow up occurred.*

This has been detailed at the beginning of the Results (page 10, line 229).

*Please include absolute numbers wherever you report percentages; eg, n/N (%)*

This has been detailed as advised, other than in the Discussion where the results are reiterated in brief.

*Please present the safety data for the study including numbers of specific events and whether or not adverse events are thought to be related to treatment. AEs should be reported in the abstract, per CONSORT and CONSORT-Harms.*

There were no serious adverse events, as reported in the abstract (page 4, line 74) and results (page 32, line 347).

*Please complete the CONSORT checklist and ensure that all components of CONSORT are present in the manuscript, including how randomization was performed, allocation concealment, blinding of intervention, definition of lost to follow-up, power statement. When completing the checklist, please use section and paragraph numbers, rather than page numbers.*

The CONSORT checklist page numbers have been updated to reflect the revisions (Supplementary Appendix B).

*Please report your abstract according to CONSORT for abstracts, following the PLOS Medicine abstract structure (Background, Methods and Findings, Conclusions).*

This has been done as advised.

*If your trial had to undergo important modifications in response to extenuating circumstances, please complete the CONSERVE-CONSORT checklist and provide in your Supporting Information; When completing the checklist, please use section and paragraph numbers, rather than page numbers.*

The CONSERVE-CONSORT checklist has been completed and uploaded as advised, as part of Supplementary Appendix B.

*Please add the following statement, or similar, to the Methods: "This study is reported as per XXXX guideline (S1 Checklist)."*

To the Methods, we have added, “…the study is reported as per CONSORT guidelines (Supplementary Appendix B, CONSORT AND CONSERVE-CONSORT checklists)” (page 7, line 144-45).

*In keeping with our commitment to Open Science, please include the original study protocol document (as approved by the ethics committee) and analysis plan (including any amendments) as Supporting Information to be published with the manuscript if accepted.*

The protocol has been uploaded as Supplementary Appendix A and the SAP as Supplementary Appendix B.

*Please note that PLOS Medicine requires prospective, public registration of a data sharing plan (as part of mandatory clinical trials registration) for all clinical trials that began enrollment on or after January 1, 2019, in accordance with ICMJE requirements.*

Please see the Data Availability Statement above.
