# Supplementary material for: Determining optimal timing of birth for women with chronic or gestational hypertension at term: The WILL (When to Induce Labour to Limit risk in pregnancy hypertension) randomised trial
Source: PLoS Med. 2024 Nov 26;21(11):e1004481. doi: 10.1371/journal.pmed.1004481 (PMC11593758; doi:10.1371/journal.pmed.1004481)
Supplement: Attachment — Submitted filename: RESPONSE R2 - 20240814.docx [file pmed.1004481.s008.docx]

Please find below our Response, in standard font, with the editors’ and reviewers’ comments in italics.

**COMMENTS FROM EDITORS**

*Please expand the discussion to include the points made by reviewer 3. Some of the comments appear to have been addressed in the rebuttal but not in the manuscript itself e.g. the change in the management of the control group. Please ensure that the reviewer's concerns are addressed fully in the discussion section.*

Thank you for the opportunity to respond further to Reviewer #3. We have reviewed all of their prior comments and our responses. We have either added additional detail to text previously in the manuscript (e.g., antihypertensive therapy), or added detail that we had provided only in response to the reviewer (e.g., HYPITAT vs. WILL trial distinction). The change in management of control group was previously in the Discussion and Discussion/Limitations, as below, but we have highlighted this for the reviewer.

**REVIEWER #1**

*The authors have added tekst in answer to the comment on long term outcomes*

*They answer:*

*Thank you for raising this important caveat about long-term outcomes. To our Discussion/Interpretation, we have added the following statement: "Also, observational data have raised concerns that in the general population, across the whole range of gestational age at birth, gestational age has a strong, dose-dependent relationship with special educational needs, including mild learning disabilities such as dyslexia and attention deficit hyperactivity disorder[(32)]. While data from non-randomised comparisons of labour induction or expectant care at term have been reassuring with regards to neurodevelopmental outcomes[(33, 34)], condition-specific data are needed. WILL participants were asked for consent for collection of routinely-collected health data, including those measuring school performance" (page 36, lines 419-26). **

*Furthermore, the authors have made thorough and sufficient comments and/or alterations in the article where deemed necessary in reply to the feedback of the other reviewers. As such, the WILL study from professor Magee and co workers (PMEDICINE-D-24-01330_R2) is judged to be acceptable for publication in PLOS Medicine.*

Thank you very much for your review, constructive criticism, and now this positive feedback on our efforts to improve the work.

**REVIEWER #2**

*Thank you to the authors for addressing my previous comments.*

Thank you for your time in reviewing the manuscript, providing suggestions for improvement, and for this positive feedback.

*On my previous comment about secondary outcomes in the abstract, I apologise for not being precise in my request (I had meant to mention *all* secondary endpoints or none).*

*I don't think it's appropriate to just have significant secondary endpoints reported in the abstract but acknowledge it's not easy to report them all properly: the authors could address this by adding 'Other secondary endpoints did not show significant differences between groups'.*

We apologise for the misunderstanding. We have added the text to the abstract as suggested: “In the intervention (vs. control) group, pre-eclampsia incidence was lower (56, 28% vs. 76, 38%, respectively; aRR 0.74, 95% CI 0.56-0.98), as were absolute mean costs (£-407.80, 95% CI -793.47 to +39.55), related to significantly lower mean costs for outpatient visits (£-235.32, 95% CI -309.45 to -154.13) and tests of maternal-fetal wellbeing (£-102.84, 95% CI -136.65 to -67.78); other secondary endpoints did not show significant differences between groups” (p4, lines 70-75).

**REVIEWER #3**

*It is unfortunate that the authors did not respond to the suggested changes.*

*I still do not believe that their conclusions are accurate given the several limitations of their study that I emphasized in my review. They have inadequate sample size , they lumped two conditions with different pathophysiology, they changed management of the control group , etc.*

*The authors responses are not convincing and the literature they cite has no relevance to the above concerns*.

It was our sincere intention to address the reviewer’s concerns, and we remain committed to doing so. As such, we have returned to the feedback initially provided by Reviewer #3 and our responses to that feedback, both pasted here and highlighted in light blue. Our additional responses are below each section, in normal font. Specifically, we have addressed recruiting a mixed population of chronic and gestational hypertension, and changing the control arm management, below, as well as other issues raised. In doing so, we have expanded the Discussion/Limitations and revised the Conclusions.

With regards to the comment about inadequate sample size, we have: (i) removed the following statement of the Discussion/Summary of findings: “As such, despite our lower-than-planned sample size, we were not underpowered to find target differences in our co-primary outcomes” (p35); and (ii) added the following statement to the Discussion/Limitations: “The event rates of the two co-primary outcomes were lower than anticipated; while the relative risk of 0.68 set *a priori* for the maternal co-primary outcome was excluded, and the clinically-important changes specified in absolute risks were also achieved, those changes in absolute risk for the maternal (8% reduction) and neonatal (8% non-inferiority margin) co-primary outcomes were unrealistically large given the lower-than-anticipated event rates” (p38-39, lines475-488).

*Reviewer’s comment: This is a non -masked randomized trial in women with non-severe GHTN or CHTN enrolled at 36-37 weeks and allocated to either planned delivery at 38 weeks or until at least 40 weeks' gestation. There are 2 co-primary outcomes; One is composite poor maternal and one neonatal morbidity. The planned sample size was 1080 participants: 540/ each arm. Unfortunately, the trial was stopped prematurely by the Funding agency after 403 (< 40%) subjects were randomized due to poor recruitment. The authors conclude that on balance, planned early term birth at 38 weeks may be the preferred clinical option for women with CHTN or GHTN.*

Our initial response (R1): We agree with this summary.

We would like to highlight that our the majority of our recruitment occurred during the COVID-19 pandemic. Our internal pilot was from 3 June 2019 until 20 March 2020, followed by a pause during the first pandemic lockdown in the UK. As such, the main recruitment (7 July 2020 to 19 December 2022) was entirely during the pandemic, and we were stopped by the funder as part of ‘pandemic reset’ designed to substantially reduce the size of the trial portfolio.

*Reviewer’s comment: There are major issues with the manuscript:*

Our initial response (R1): We believe that we have presented a robust response to the reviewer’s concerns such that if s/he may still disagree with us on some of these points, s/he may agree that there is a spectrum of opinion on the topic. This is acknowledged by our previous Discussion/Limitations section, which highlights that we: reached 37% of our target sample size, recruited women with chronic or gestational hypertension, and revised our control arm slightly late in recruitment (page 37, lines 432-38).

We have reviewed all of the reviewer’s comments and our responses. We have either added additional detail in the manuscript, or added detail that we had provided only in response to the reviewer.

*Reviewer’s comment 1. Women with GHTN have a different pathophysiology than those with CHTN. Therefore, it was inappropriate to lump them together unless they had adequate sample size for each group with a planned secondary analysis.*

Our initial response (R1): While the pathophysiology of the underlying hypertension may be different in women with chronic vs. gestational hypertension, we feel that combining them is justified. First, there is precedent. They were previously combined for in the international CHIPS trial of differential blood pressure control in pregnancy [Magee LA et al. N Engl J Med 2015;372(5):407-17], with no evidence of subgroup effects by hypertension type. Importantly, similar results were seen in the American CHAP trial when the study population was restricted to women with chronic hypertension [Tita AT et al. N Engl J Med 2022;386(19):1781-1792]. Second, women with either chronic or gestational hypertension are at increased risk of progression to pre-eclampsia at term gestational age, and so the clinical question to be addressed is the same (i.e., should one proceed with timed birth before pre-eclampsia or other problems develop, or should [one] wait for complications to occur and then react with induction/Caesarean). Peer review has supported combining types of pregnancy hypertension in such circumstances, including the landmark HYPITAT I [Koopmans CM et al. Induction of labour versus expectant monitoring for gestational hypertension or mild pre-eclampsia after 36 weeks' gestation (HYPITAT): a multicentre, open-label randomised controlled trial. Lancet 2009;374(9694):979-88] and II trials [Broekhuijsen K et al. Lancet 2015;385(9986):2492-501] of timed birth in pregnancy hypertension, and the ongoing giantPANDA trial of nifedipine vs. labetalol [ISRCTN reference number 12792616].

We had listed our decision to combine chronic and gestational hypertension (with minimisation of randomisation by hypertension type) as a potential limitation, with qualification, as follows: “We included women with either chronic or gestational hypertension, but results for the co-primary outcomes were similar by hypertension type” (page 37, lines 434-6).

We understand that the reviewer does not regard references about antihypertensive therapy for chronic or gestational hypertension as germane to a discussion of timed birth in this population. However, these references were provided to illustrate the other high-impact publications, from our group and from others, have taken such an approach. All have undertaken planned subgroup analyses, as we have in the WILL trial, although no such analyses were powered to address the research question in each of chronic or gestational hypertension populations.

We have expanded our comment on this topic within the Discussion/Limitations, as follows: “We included women with either chronic or gestational hypertension; while recruiting a population of mixed hypertensive type is common in pregnancy hypertension trials(2) and results for the co-primary outcomes were similar by hypertension type, such subgroup analyses were predictably underpowered” (p38, lines 469-472).

*Reviewer’s comment 2. Given, the premature termination of the trial, the conclusions in the abstract and manuscript should be changed to state that it the optimal timing of delivery for either group of women remains unknown.*

Our initial response (R1): We have been open about the nature and reasons for premature withdrawal of funding by our Sponsor and a mandated end to the trial. What the reviewer suggests implies that the information provided by this trial is not relevant to clinical practice. While we would not suggest that our results are definitive, we do believe that they should inform practice until further information becomes available. Given the low adverse event rates in this era of blood pressure control – internationally (CHIPS trial) and in the United States (CHAP trial) – we suggest that further information may not be forthcoming in the near future; this is stated at the end of the Discussion/Interpretation (page 36, lines 416-9).

The conclusions of the abstract (p4, lines 77-79) and manuscript (p39, lines 491-493) have been revised, as follows: “Pending definitive data, the WILL trial findings provide reassurance about planned early term birth at 38^+0-3^ weeks as a clinical option for women with chronic or gestational hypertension who reach this gestational age undelivered”. We have highlighted in our Discussion that it may be difficult to mount another such trial given our low event rates in this blood pressure control era, so it is important to state the clinical relevance of the findings.

*Reviewer’s comment 3. It was inappropriate to change timing of delivery for the usual care group in the middle of the trial.*

Our initial response (R1): As described in the Methods, “the control group was changed from ‘expectant care until at least 40^+0^ weeks’ to ‘usual care at term’, to reflect practice change related to timed birth in other populations(13, 14), the COVID-19 pandemic(15), and draft national labour induction guidance suggesting timed birth at 39 weeks may be appropriate for women at increased risk of term complications(16)” (page 7, lines 164-8). The change was approved by all WILL Oversight Committees, our funder, and the relevant Research Ethics Board. This suggests that while there may be a spectrum of opinion on this issue, our decision was reasonable.

Our prior response emphasised that this was a decision that was thoroughly debated within our oversight committees, and ethics-approved. It reflected current clinical practice. However, it may also be useful to emphasise the statement that we made in our prior Discussion/Limitations that, “While the label of our control arm was changed to ‘usual care at term’, this applied only to the final 14% of recruits, and throughout, the control group reflected current practice” (p38, lines 472-474).

*Reviewer’s comment 4. The number of women with GHTN in this trial was only 208 (105 v.103), and for CHTN it was only 195 (96 v. 99). Therefore, it is difficult to draw any conclusions or recommendations from this trial, particularly since the HYPITAT trial had more women with GHTN than this trial, and recommendation is to do delivery at 37 weeks' gestation.*

Our initial response (R1): The differences between WILL and HYPITAT has been a source of confusion. WILL recruited women who developed gestational hypertension preterm, but who remained well at term, and randomised them to planned birth at 38+0-3 weeks (vs. expectant care). In contrast, HYPITAT recruited women who developed gestational hypertension from 36+0 weeks and randomised them to immediate birth (vs. expectant care). In HYPITAT, 188/756 women were recruited at 37+0-6 weeks and as it is not known what proportion of these women had gestational hypertension (which was 65% overall), it is likely that no more than 122 women in HYPITAT would have been eligible for WILL.

The differences between the HYPITAT and WILL trials have been a source of confusion, including in reviews of the first WILL application to NIHR (UK funder). However, WILL is about planned birth for women who have developed gestational hypertension and now are close to term gestational age and remain well. The trials are fundamentally different, and having Professor Ben Mol (HYPITAT) as a co-investigator on WILL makes this point clearly.

We have now added an explanation about the difference between HYPITAT and WILL, formerly provided only to the reviewer, to our Discussion/Limitations, as follows: “Nevertheless, WILL is the largest randomised evaluation of timed birth for this group of women who reach term gestational age and remain well; as women in the HYPITAT trial were recruited when they developed gestational hypertension (or pre-eclampsia), 188/756 women in the trial overall were recruited at 37+0-6 weeks (when they were randomised to labour induction within 24 hours or ongoing expectant care) when women in WILL were randomised to planned timed birth at 38+0-3 wks vs. usual care, and 65% of the HYPITAT study population overall had gestational hypertension, so it is likely that no more than 122 women in HYPITAT would have been eligible for WILL” (p38, lines 462-469).

*Reviewer’s comment 5. Almost 80% of enrolled subjects were receiving antihypertensive medications at enrollment, yet the authors considered these women to have non-severe hypertension. Thus, the authors should state that their findings apply only to women on medications, and does not address non- severe patients without medications.*

Our initial response (R1): Women with non-severe hypertension are those with BP ≥140/90mmHg by all international standards in pregnancy. Internationally, such women are now recommended to receive antihypertensive therapy to reach a target BP of at least 135/85mmHg [Magee LA et al. The 2021 International Society for the Study of Hypertension in Pregnancy classification, diagnosis & management recommendations for international practice. Pregnancy Hypertens 2022;27:148-69]; therefore, our study results will apply to those receiving evidence-based care. Our Discussion highlights that our findings “may be due to WILL being undertaken in the current era of good BP control” (page 35, lines 391-2). We still regard women with antihypertensive treated non-severe hypertension to have the condition of hypertension, in the same way as we regard those with diabetes to remain so afflicted even when their hyperglycaemia is treated with oral agents or insulin.

That the WILL trial was undertaken in an era of BP control is actually a strength. As such, we have added it to the Discussion/Strengths, as follows: “A strength of the trial was its generalisability to real-world care of women with chronic or gestational hypertension, through inclusion of women with co-morbidities, contemporary treatment (control) of BP with antihypertensive therapy, and comparison of planned early term birth with usual clinical practice” (p38, lines 456-459). However, to provide a balanced argument, we have added the following statement to the Discussion/Limitations: “The findings may not be generalisable to where hypertension is not treated with antihypertensive therapy, despite international recommendations” (p38, lines 474-475).

**REVIEWER #4**

*All comments and concerns have been adequately addressed and appropriates additions made to the manuscript. Well done.*

Many thanks for your constructive feedback and for recognising our efforts to improve the manuscript.

**ADDITIONAL EDITORIAL INSTRUCTIONS**

** We ask every co-author listed on the manuscript to fill in a contributing author statement, making sure to declare all competing interests. If any of the co-authors have not filled in the statement, we will remind them to do so when the paper is revised. If all statements are not completed in a timely fashion this could hold up the re-review process. If new competing interests are declared later in the revision process, this may also hold up the submission. Should there be a problem getting one of your co-authors to fill in a statement we will be in contact. Please do not add or remove authors without first discussing this with the handling editor. You can see our competing interests policy here:* [*https://eur03.safelinks.protection.outlook.com/?url=http%3A%2F%2Fjournals.plos.org%2Fplosmedicine%2Fs%2Fcompeting-interests&data=05%7C02%7Claura.a.magee%40kcl.ac.uk%7Cd08f0769009b487c9bf508dcb7c9f5b0%7C8370cf1416f34c16b83c724071654356%7C0%7C0%7C638587325242418375%7CUnknown%7CTWFpbGZsb3d8eyJWIjoiMC4wLjAwMDAiLCJQIjoiV2luMzIiLCJBTiI6Ik1haWwiLCJXVCI6Mn0%3D%7C0%7C%7C%7C&sdata=in2UmxztWrPrRYSiPA8kqzEapipp0tSzo6fnFvoegtw%3D&reserved=0*](https://eur03.safelinks.protection.outlook.com/?url=http%3A%2F%2Fjournals.plos.org%2Fplosmedicine%2Fs%2Fcompeting-interests&data=05%7C02%7Claura.a.magee%40kcl.ac.uk%7Cd08f0769009b487c9bf508dcb7c9f5b0%7C8370cf1416f34c16b83c724071654356%7C0%7C0%7C638587325242418375%7CUnknown%7CTWFpbGZsb3d8eyJWIjoiMC4wLjAwMDAiLCJQIjoiV2luMzIiLCJBTiI6Ik1haWwiLCJXVCI6Mn0%3D%7C0%7C%7C%7C&sdata=in2UmxztWrPrRYSiPA8kqzEapipp0tSzo6fnFvoegtw%3D&reserved=0).

All authors have complied COI statements.

** Please upload any figures associated with your paper as individual TIF or EPS files with 300dpi resolution at resubmission; please read our figure guidelines for more information on our requirements:* [*https://eur03.safelinks.protection.outlook.com/?url=http%3A%2F%2Fjournals.plos.org%2Fplosmedicine%2Fs%2Ffigures&data=05%7C02%7Claura.a.magee%40kcl.ac.uk%7Cd08f0769009b487c9bf508dcb7c9f5b0%7C8370cf1416f34c16b83c724071654356%7C0%7C0%7C638587325242427855%7CUnknown%7CTWFpbGZsb3d8eyJWIjoiMC4wLjAwMDAiLCJQIjoiV2luMzIiLCJBTiI6Ik1haWwiLCJXVCI6Mn0%3D%7C0%7C%7C%7C&sdata=cop4N0ZcH0dCjblEC4P1vEKX04t7cLPjQR2Otc5zv74%3D&reserved=0*](https://eur03.safelinks.protection.outlook.com/?url=http%3A%2F%2Fjournals.plos.org%2Fplosmedicine%2Fs%2Ffigures&data=05%7C02%7Claura.a.magee%40kcl.ac.uk%7Cd08f0769009b487c9bf508dcb7c9f5b0%7C8370cf1416f34c16b83c724071654356%7C0%7C0%7C638587325242427855%7CUnknown%7CTWFpbGZsb3d8eyJWIjoiMC4wLjAwMDAiLCJQIjoiV2luMzIiLCJBTiI6Ik1haWwiLCJXVCI6Mn0%3D%7C0%7C%7C%7C&sdata=cop4N0ZcH0dCjblEC4P1vEKX04t7cLPjQR2Otc5zv74%3D&reserved=0).

Our previously uploaded Figure complies with these instructions.

** While revising your submission, please upload your figure files to the PACE digital diagnostic tool,* [*https://eur03.safelinks.protection.outlook.com/?url=https%3A%2F%2Fpacev2.apexcovantage.com%2F&data=05%7C02%7Claura.a.magee%40kcl.ac.uk%7Cd08f0769009b487c9bf508dcb7c9f5b0%7C8370cf1416f34c16b83c724071654356%7C0%7C0%7C638587325242433565%7CUnknown%7CTWFpbGZsb3d8eyJWIjoiMC4wLjAwMDAiLCJQIjoiV2luMzIiLCJBTiI6Ik1haWwiLCJXVCI6Mn0%3D%7C0%7C%7C%7C&sdata=hf3lN5EAwwS%2BJhdE4yHHGJseeEEPuNHcCRaZ3%2BYuCoU%3D&reserved=0*](https://eur03.safelinks.protection.outlook.com/?url=https%3A%2F%2Fpacev2.apexcovantage.com%2F&data=05%7C02%7Claura.a.magee%40kcl.ac.uk%7Cd08f0769009b487c9bf508dcb7c9f5b0%7C8370cf1416f34c16b83c724071654356%7C0%7C0%7C638587325242433565%7CUnknown%7CTWFpbGZsb3d8eyJWIjoiMC4wLjAwMDAiLCJQIjoiV2luMzIiLCJBTiI6Ik1haWwiLCJXVCI6Mn0%3D%7C0%7C%7C%7C&sdata=hf3lN5EAwwS%2BJhdE4yHHGJseeEEPuNHcCRaZ3%2BYuCoU%3D&reserved=0)*. PACE helps ensure that figures meet PLOS requirements. To use PACE, you must first register as a user. Then, login and navigate to the UPLOAD tab, where you will find detailed instructions on how to use the tool. If you encounter any issues or have any questions when using PACE, please email us at* [*PLOSMedicine@plos.org*](mailto:PLOSMedicine@plos.org)*.*

This was previously done, as suggested.

** Please ensure that the paper adheres to the PLOS Data Availability Policy (see* [*https://eur03.safelinks.protection.outlook.com/?url=http%3A%2F%2Fjournals.plos.org%2Fplosmedicine%2Fs%2Fdata-availability&data=05%7C02%7Claura.a.magee%40kcl.ac.uk%7Cd08f0769009b487c9bf508dcb7c9f5b0%7C8370cf1416f34c16b83c724071654356%7C0%7C0%7C638587325242438506%7CUnknown%7CTWFpbGZsb3d8eyJWIjoiMC4wLjAwMDAiLCJQIjoiV2luMzIiLCJBTiI6Ik1haWwiLCJXVCI6Mn0%3D%7C0%7C%7C%7C&sdata=ElxsFlu4yEBzVEj%2BR8pefByNalErCFm028ECMJdo8OU%3D&reserved=0*](https://eur03.safelinks.protection.outlook.com/?url=http%3A%2F%2Fjournals.plos.org%2Fplosmedicine%2Fs%2Fdata-availability&data=05%7C02%7Claura.a.magee%40kcl.ac.uk%7Cd08f0769009b487c9bf508dcb7c9f5b0%7C8370cf1416f34c16b83c724071654356%7C0%7C0%7C638587325242438506%7CUnknown%7CTWFpbGZsb3d8eyJWIjoiMC4wLjAwMDAiLCJQIjoiV2luMzIiLCJBTiI6Ik1haWwiLCJXVCI6Mn0%3D%7C0%7C%7C%7C&sdata=ElxsFlu4yEBzVEj%2BR8pefByNalErCFm028ECMJdo8OU%3D&reserved=0)*), which requires that all data underlying the study's findings be provided in a repository or as Supporting Information. For data residing with a third party, authors are required to provide instructions with contact information (web or email address) for obtaining the data. Please note that a study author cannot be the contact person for the data. PLOS journals do not allow statements supported by "data not shown" or "unpublished results." For such statements, authors must provide supporting data or cite public sources that include it.*

The data reside with the Birmingham Clinical Trials Unit, and we have now named <name> as the contact, rather than the Chief Investigator which we now understand is not permissible (as below). The Data Availability Statement is as follows: “Requests for data should be directed to Hiten Mistry ([hiten.mistry@kcl.ac.uk](mailto:hiten.mistry@kcl.ac.uk)). Requests will be assessed for scientific rigour before being granted. Data will be anonymised and securely transferred. A data-sharing agreement might be required”.

** We expect all researchers with submissions to PLOS in which author-generated code underpins the findings in the manuscript to make all author-generated code available without restrictions upon publication of the work. In cases where code is central to the manuscript, we may require the code to be made available as a condition of publication. Authors are responsible for ensuring that the code is reusable and well documented. Please make any custom code available, either as part of your data deposition or as a supplementary file. Please add a sentence to your data availability statement regarding any code used in the study, e.g. "The code used in the analysis is available from Github [URL] and archived in Zenodo [DOI link]" Please review our guidelines at* [*https://eur03.safelinks.protection.outlook.com/?url=https%3A%2F%2Fjournals.plos.org%2Fplosmedicine%2Fs%2Fmaterials-software-and-code-sharing&data=05%7C02%7Claura.a.magee%40kcl.ac.uk%7Cd08f0769009b487c9bf508dcb7c9f5b0%7C8370cf1416f34c16b83c724071654356%7C0%7C0%7C638587325242443119%7CUnknown%7CTWFpbGZsb3d8eyJWIjoiMC4wLjAwMDAiLCJQIjoiV2luMzIiLCJBTiI6Ik1haWwiLCJXVCI6Mn0%3D%7C0%7C%7C%7C&sdata=IT7k7aJkiQGvJE7fMFGrUjrZkUGDGwhg2yoQzLDhjE8%3D&reserved=0*](https://eur03.safelinks.protection.outlook.com/?url=https%3A%2F%2Fjournals.plos.org%2Fplosmedicine%2Fs%2Fmaterials-software-and-code-sharing&data=05%7C02%7Claura.a.magee%40kcl.ac.uk%7Cd08f0769009b487c9bf508dcb7c9f5b0%7C8370cf1416f34c16b83c724071654356%7C0%7C0%7C638587325242443119%7CUnknown%7CTWFpbGZsb3d8eyJWIjoiMC4wLjAwMDAiLCJQIjoiV2luMzIiLCJBTiI6Ik1haWwiLCJXVCI6Mn0%3D%7C0%7C%7C%7C&sdata=IT7k7aJkiQGvJE7fMFGrUjrZkUGDGwhg2yoQzLDhjE8%3D&reserved=0) *and ensure that your code is shared in a way that follows best practice and facilitates reproducibility and reuse. Because Github depositions can be readily changed or deleted, we encourage you to make a permanent DOI'd copy (e.g. in Zenodo) and provide the URL.*

Sharing the analytic code, which is not central to the manuscript, would require that the participant-level data be made available too. Please see our Data Availability Statement above.

** If the data are not freely available, please describe briefly the ethical, legal, or contractual restriction that prevents you from sharing it in the Data Availability Statement. Please also include an appropriate contact (web or email address) for inquiries. Please note that a study author cannot be the contact person for the data.*

Please see our Data Availability Statemen, with Hiten Mistry as the contact person. He is neither a study author nor involved in the trial.

** Please provide a copy of the original trial protocol (as provided to and approved by the ethics committee) and the original statistical analysis plan as supplementary files. Please confirm whether the SAP was finalised prior to data lock, and that data was not accessible to the investigators prior to finalisation of the SAP. Please also provide a copy of the ethical approval document as a supplementary file.*

The original trial protocol, original statistical analysis plan (SAP), and original ethical approval have been uploaded as supplementary files. The SAP was finalised prior to data lock, and the data were not accessible to the investigators prior to finalisation of the SAP.

**FORMATTING - GENERAL**

** Abstract: Please structure your abstract using the PLOS Medicine headings (Background, Methods and Findings, Conclusions). Please combine the Methods and Findings sections into one section.*

This was done during the first revision, thank you.

** At this stage, we ask that you include a short, non-technical Author Summary of your research to make findings accessible to a wide audience that includes both scientists and non-scientists. The Author Summary should immediately follow the Abstract in your revised manuscript. This text is subject to editorial change and should be distinct from the scientific abstract. Ideally each sub-heading should contain 2-3 single sentence, concise bullet points containing the most salient points from your study. In the final bullet point of 'What Do These Findings Mean?', please include the main limitations of the study in non-technical language. Please see our author guidelines for more information:* [*https://eur03.safelinks.protection.outlook.com/?url=https%3A%2F%2Fjournals.plos.org%2Fplosmedicine%2Fs%2Frevising-your-manuscript%23loc-author-summary&data=05%7C02%7Claura.a.magee%40kcl.ac.uk%7Cd08f0769009b487c9bf508dcb7c9f5b0%7C8370cf1416f34c16b83c724071654356%7C0%7C0%7C638587325242447552%7CUnknown%7CTWFpbGZsb3d8eyJWIjoiMC4wLjAwMDAiLCJQIjoiV2luMzIiLCJBTiI6Ik1haWwiLCJXVCI6Mn0%3D%7C0%7C%7C%7C&sdata=LXwsWpDwhX7MPMmLin1%2FmhkXWjXOV%2BK49bxqelT6qqo%3D&reserved=0*](https://eur03.safelinks.protection.outlook.com/?url=https%3A%2F%2Fjournals.plos.org%2Fplosmedicine%2Fs%2Frevising-your-manuscript%23loc-author-summary&data=05%7C02%7Claura.a.magee%40kcl.ac.uk%7Cd08f0769009b487c9bf508dcb7c9f5b0%7C8370cf1416f34c16b83c724071654356%7C0%7C0%7C638587325242447552%7CUnknown%7CTWFpbGZsb3d8eyJWIjoiMC4wLjAwMDAiLCJQIjoiV2luMzIiLCJBTiI6Ik1haWwiLCJXVCI6Mn0%3D%7C0%7C%7C%7C&sdata=LXwsWpDwhX7MPMmLin1%2FmhkXWjXOV%2BK49bxqelT6qqo%3D&reserved=0).

This was included, as instructed, during the first revision, thank you.

** Please express the main results with 95% CIs as well as p values. When reporting p values please report as p<0.001 and where higher as the exact p value p=0.002, for example. Throughout, suggest reporting statistical information as follows to improve clarity for the reader "22% (95% CI [13%,28%]; p</=)". Please be sure to define all numerical values at first use.*

In our SAP, we stated that, “Null hypotheses have not been stated as we are not presenting p-values or discussing statistical significance, but will focus on the treatment effect measures and 95% CIs”. However, we recognised that this conflicts with *PLoS Med* policy. As such, we had previously added the p values to all tables, main and supplementary. We have now added the p values, as relevant, to the text (p15, line 273; p22, lines 309, 324, 319, 326, 327; p23, line 333; p33, line 370).

** Please include page numbers and line numbers in the manuscript file. Use continuous line numbers (do not restart the numbering on each page).*

This was included, as instructed, during the first revision, thank you.

** Please cite the reference numbers in square brackets. Citations should precede punctuation.*

This was included, as instructed, during the first revision, thank you.

**FIGURES AND TABLES**

** Please provide titles and legends for all figures and tables (including those in Supporting Information files).*

A legend was added for Figure 1. All other tables in the main document and Supplementary Appendix had legends from the prior submission.

** Please define all abbreviations used in each figure/table (including those in Supporting Information files).*

Any omissions have been revised in the main document and Supplementary Appendix, thank you.

** Please consider avoiding the use of red and green in order to make your figure more accessible to those with color blindness.*

We have used only blue in our tables and figures.

**SUPPLEMENTARY MATERIAL**

** Please note that supplementary material will be posted as supplied by the authors. Therefore, please amend it according to the relevant comments outlined here.*

This has been done, as requested.

** Please cite your Supporting Information as outlined here:* [*https://eur03.safelinks.protection.outlook.com/?url=https%3A%2F%2Fjournals.plos.org%2Fplosmedicine%2Fs%2Fsupporting-information&data=05%7C02%7Claura.a.magee%40kcl.ac.uk%7Cd08f0769009b487c9bf508dcb7c9f5b0%7C8370cf1416f34c16b83c724071654356%7C0%7C0%7C638587325242452000%7CUnknown%7CTWFpbGZsb3d8eyJWIjoiMC4wLjAwMDAiLCJQIjoiV2luMzIiLCJBTiI6Ik1haWwiLCJXVCI6Mn0%3D%7C0%7C%7C%7C&sdata=zUbqsmdR7WcjogL6F1yWNgjsItdyL%2BsvUqbkqNtqFa8%3D&reserved=0*](https://eur03.safelinks.protection.outlook.com/?url=https%3A%2F%2Fjournals.plos.org%2Fplosmedicine%2Fs%2Fsupporting-information&data=05%7C02%7Claura.a.magee%40kcl.ac.uk%7Cd08f0769009b487c9bf508dcb7c9f5b0%7C8370cf1416f34c16b83c724071654356%7C0%7C0%7C638587325242452000%7CUnknown%7CTWFpbGZsb3d8eyJWIjoiMC4wLjAwMDAiLCJQIjoiV2luMzIiLCJBTiI6Ik1haWwiLCJXVCI6Mn0%3D%7C0%7C%7C%7C&sdata=zUbqsmdR7WcjogL6F1yWNgjsItdyL%2BsvUqbkqNtqFa8%3D&reserved=0)

The file is less than 10MB and contains only tables. The instructions say that one may use almost any description as the item name of supporting information, as long as it contains an “S” and number. As such, we have retained the format of ‘Table S#’, rather than ‘S# Table’. We are happy to revise if required.

**REFERENCES**

** PLOS uses the numbered citation (citation-sequence) method and first six authors, et al.*

This was done previously, as advised.

** Please ensure that journal name abbreviations match those found in the National Center for Biotechnology Information (NCBI) databases (*[*https://eur03.safelinks.protection.outlook.com/?url=http%3A%2F%2Fwww.ncbi.nlm.nih.gov%2Fnlmcatalog%2Fjournals&data=05%7C02%7Claura.a.magee%40kcl.ac.uk%7Cd08f0769009b487c9bf508dcb7c9f5b0%7C8370cf1416f34c16b83c724071654356%7C0%7C0%7C638587325242456321%7CUnknown%7CTWFpbGZsb3d8eyJWIjoiMC4wLjAwMDAiLCJQIjoiV2luMzIiLCJBTiI6Ik1haWwiLCJXVCI6Mn0%3D%7C0%7C%7C%7C&sdata=vJP6ngzsm5lNVGRZjqBa9feHDkZKCe6SCTbL%2BVank2s%3D&reserved=0*](https://eur03.safelinks.protection.outlook.com/?url=http%3A%2F%2Fwww.ncbi.nlm.nih.gov%2Fnlmcatalog%2Fjournals&data=05%7C02%7Claura.a.magee%40kcl.ac.uk%7Cd08f0769009b487c9bf508dcb7c9f5b0%7C8370cf1416f34c16b83c724071654356%7C0%7C0%7C638587325242456321%7CUnknown%7CTWFpbGZsb3d8eyJWIjoiMC4wLjAwMDAiLCJQIjoiV2luMzIiLCJBTiI6Ik1haWwiLCJXVCI6Mn0%3D%7C0%7C%7C%7C&sdata=vJP6ngzsm5lNVGRZjqBa9feHDkZKCe6SCTbL%2BVank2s%3D&reserved=0)*), and are appropriately formatted and capitalised.*

This was done previously, thank you.

** Where website addresses are cited, please include the complete URL and specify the date of access (e.g. [accessed: 12/06/2023]).*

This was previously done, as advised.

** Please also see* [*https://eur03.safelinks.protection.outlook.com/?url=https%3A%2F%2Fjournals.plos.org%2Fplosmedicine%2Fs%2Fsubmission-guidelines%23loc-references&data=05%7C02%7Claura.a.magee%40kcl.ac.uk%7Cd08f0769009b487c9bf508dcb7c9f5b0%7C8370cf1416f34c16b83c724071654356%7C0%7C0%7C638587325242460519%7CUnknown%7CTWFpbGZsb3d8eyJWIjoiMC4wLjAwMDAiLCJQIjoiV2luMzIiLCJBTiI6Ik1haWwiLCJXVCI6Mn0%3D%7C0%7C%7C%7C&sdata=qUhBYvxmpJtJzNGmvwECqAEMvljURoRovCz2wby9UWk%3D&reserved=0*](https://eur03.safelinks.protection.outlook.com/?url=https%3A%2F%2Fjournals.plos.org%2Fplosmedicine%2Fs%2Fsubmission-guidelines%23loc-references&data=05%7C02%7Claura.a.magee%40kcl.ac.uk%7Cd08f0769009b487c9bf508dcb7c9f5b0%7C8370cf1416f34c16b83c724071654356%7C0%7C0%7C638587325242460519%7CUnknown%7CTWFpbGZsb3d8eyJWIjoiMC4wLjAwMDAiLCJQIjoiV2luMzIiLCJBTiI6Ik1haWwiLCJXVCI6Mn0%3D%7C0%7C%7C%7C&sdata=qUhBYvxmpJtJzNGmvwECqAEMvljURoRovCz2wby9UWk%3D&reserved=0) *for further details on reference formatting.*

The references were formatted by EndNote, using the format advised.

**RCTs**

** PLOS Medicine requires that all trials be prospectively registered in one of registries recognized by WHO. Please ensure that study registration details are included in the Methods section.*

This was done previously, as advised.

** Please structure the Methods section using the following sub-headings: Study design and participants, Randomization and masking, Procedures, Outcomes, Statistical analysis.*

This was done previously, as advised.

** Please clarify and explain all discrepancies between the paper and protocol. If the outcomes were not prespecified in the protocol, please define them in the Methods (Outcomes section) as post hoc and explain why they were added. Post-hoc comparisons should be presented as hypothesis generating rather than conclusive.*

We had changed “instrumental” vaginal birth to “operative” vaginal birth as our preference but with the same meaning, but to avoid the appearance of changing the outcomes, we have reverted to “instrumental” in the text, main tables, and supplementary tables. Also, we presented a descriptive breakdown of Caesarean that we have now added to Table S3, for transparency. We apologise for this oversight.

** Please ensure that all prespecified outcomes (primary, secondary, and exploratory) are listed in the Methods/Outcomes section and indicate whether there are outcomes that are not presented in the current report.*

This was done previously, as advised.

** Please specify the dates (Month Day, Year) during which study enrollment and follow up occurred.*

This was presented at the beginning of the Results.

** Please include absolute numbers wherever you report percentages; eg, n/N (%)*

This was done previously as advised, other than in the Discussion where the results are reiterated in brief.

** Please present the safety data for the study including numbers of specific events and whether or not adverse events are thought to be related to treatment. AEs should be reported in the abstract, per CONSORT and CONSORT-Harms.*

There were no serious adverse events, as reported in the abstract and results. We have now added the following to the methods (“Adverse events were captured via pre-defined outcome measures. A serious adverse event was one that resulted in death, was life-threatening, required hospitalisation or prolongation of existing hospitalisation, resulted in persistent or significant disability or incapacity, may have jeopardised the pregnancy, or may have required intervention to prevent one of the other outcomes listed above”; p8-9, lines 194-198) and Table S3 that defines the secondary outcomes (Supplementary Appendix).

** Please complete the CONSORT checklist (*[*https://eur03.safelinks.protection.outlook.com/?url=https%3A%2F%2Fwww.equator-network.org%2Freporting-guidelines%2Fconsort%2F&data=05%7C02%7Claura.a.magee%40kcl.ac.uk%7Cd08f0769009b487c9bf508dcb7c9f5b0%7C8370cf1416f34c16b83c724071654356%7C0%7C0%7C638587325242464488%7CUnknown%7CTWFpbGZsb3d8eyJWIjoiMC4wLjAwMDAiLCJQIjoiV2luMzIiLCJBTiI6Ik1haWwiLCJXVCI6Mn0%3D%7C0%7C%7C%7C&sdata=aASYSIcj7qqLUA1K%2FS5VUapdi4OAALgYG7L6kPx%2F1Zo%3D&reserved=0*](https://eur03.safelinks.protection.outlook.com/?url=https%3A%2F%2Fwww.equator-network.org%2Freporting-guidelines%2Fconsort%2F&data=05%7C02%7Claura.a.magee%40kcl.ac.uk%7Cd08f0769009b487c9bf508dcb7c9f5b0%7C8370cf1416f34c16b83c724071654356%7C0%7C0%7C638587325242464488%7CUnknown%7CTWFpbGZsb3d8eyJWIjoiMC4wLjAwMDAiLCJQIjoiV2luMzIiLCJBTiI6Ik1haWwiLCJXVCI6Mn0%3D%7C0%7C%7C%7C&sdata=aASYSIcj7qqLUA1K%2FS5VUapdi4OAALgYG7L6kPx%2F1Zo%3D&reserved=0)*) and ensure that all components of CONSORT are present in the manuscript, including how randomization was performed, allocation concealment, blinding of intervention, definition of lost to follow-up, power statement. When completing the checklist, please use section and paragraph numbers, rather than page numbers.*

The CONSORT and CONSERVE-CONSORT checklists were submitted previously as Supplementary Appendix B. The page numbers have been checked against the new, revised version of the manuscript.

** Please report your abstract according to CONSORT for abstracts, following the PLOS Medicine abstract structure (Background, Methods and Findings, Conclusions)* [*https://eur03.safelinks.protection.outlook.com/?url=https%3A%2F%2Fwww.equator-network.org%2Freporting-guidelines%2Fconsort-abstracts%2F&data=05%7C02%7Claura.a.magee%40kcl.ac.uk%7Cd08f0769009b487c9bf508dcb7c9f5b0%7C8370cf1416f34c16b83c724071654356%7C0%7C0%7C638587325242468611%7CUnknown%7CTWFpbGZsb3d8eyJWIjoiMC4wLjAwMDAiLCJQIjoiV2luMzIiLCJBTiI6Ik1haWwiLCJXVCI6Mn0%3D%7C0%7C%7C%7C&sdata=%2FNwBnHYtAOn4hRD%2BgssI3s0YoZY5VRwYdfqjcqMMnE4%3D&reserved=0*](https://eur03.safelinks.protection.outlook.com/?url=https%3A%2F%2Fwww.equator-network.org%2Freporting-guidelines%2Fconsort-abstracts%2F&data=05%7C02%7Claura.a.magee%40kcl.ac.uk%7Cd08f0769009b487c9bf508dcb7c9f5b0%7C8370cf1416f34c16b83c724071654356%7C0%7C0%7C638587325242468611%7CUnknown%7CTWFpbGZsb3d8eyJWIjoiMC4wLjAwMDAiLCJQIjoiV2luMzIiLCJBTiI6Ik1haWwiLCJXVCI6Mn0%3D%7C0%7C%7C%7C&sdata=%2FNwBnHYtAOn4hRD%2BgssI3s0YoZY5VRwYdfqjcqMMnE4%3D&reserved=0)

This was done on the previous submission, thank you.

** If your trial had to undergo important modifications in response to extenuating circumstances, please complete the CONSERVE-CONSORT checklist and provide in your Supporting Information; (*[*https://eur03.safelinks.protection.outlook.com/?url=https%3A%2F%2Fwww.equator-network.org%2Freporting-guidelines%2Fguidelines-for-reporting-trial-protocols-and-completed-trials-modified-due-to-the-covid-19-pandemic-and-other-extenuating-circumstances-the-conserve-2021-statement%2F&data=05%7C02%7Claura.a.magee%40kcl.ac.uk%7Cd08f0769009b487c9bf508dcb7c9f5b0%7C8370cf1416f34c16b83c724071654356%7C0%7C0%7C638587325242472643%7CUnknown%7CTWFpbGZsb3d8eyJWIjoiMC4wLjAwMDAiLCJQIjoiV2luMzIiLCJBTiI6Ik1haWwiLCJXVCI6Mn0%3D%7C0%7C%7C%7C&sdata=DYgl1k9mF%2BPn0EXuClifkD5DNpMbypgoD8ISUfchjmA%3D&reserved=0*](https://eur03.safelinks.protection.outlook.com/?url=https%3A%2F%2Fwww.equator-network.org%2Freporting-guidelines%2Fguidelines-for-reporting-trial-protocols-and-completed-trials-modified-due-to-the-covid-19-pandemic-and-other-extenuating-circumstances-the-conserve-2021-statement%2F&data=05%7C02%7Claura.a.magee%40kcl.ac.uk%7Cd08f0769009b487c9bf508dcb7c9f5b0%7C8370cf1416f34c16b83c724071654356%7C0%7C0%7C638587325242472643%7CUnknown%7CTWFpbGZsb3d8eyJWIjoiMC4wLjAwMDAiLCJQIjoiV2luMzIiLCJBTiI6Ik1haWwiLCJXVCI6Mn0%3D%7C0%7C%7C%7C&sdata=DYgl1k9mF%2BPn0EXuClifkD5DNpMbypgoD8ISUfchjmA%3D&reserved=0)*). When completing the checklist, please use section and paragraph numbers, rather than page numbers.*

The CONSORT and CONSERVE-CONSORT checklists were submitted previously as Supplementary Appendix B. The page numbers have been checked against the new, revised version of the manuscript.

** In keeping with our commitment to Open Science, please include the study protocol document and analysis plan (including any amendments) as Supporting Information to be published with the manuscript if accepted.*

The latest versions were included in the prior submission. In addition, we have now uploaded the original protocol and SAP, as requested above.

** Please note that PLOS Medicine requires prospective, public registration of a data sharing plan (as part of mandatory clinical trials registration) for all clinical trials that began enrollment on or after January 1, 2019, in accordance with ICMJE requirements.*

Please see the Data Availability Statement. Also, the Birmingham Clinical Trials Unit ‘Data Sharing - Outline Request for Data Form’ has been uploaded.

** In compliance with data protection regulations, you may request that we remove your personal registration details at any time. (Use the following URL:* [*https://eur03.safelinks.protection.outlook.com/?url=https%3A%2F%2Fwww.editorialmanager.com%2Fpmedicine%2Flogin.asp%3Fa%3Dr&data=05%7C02%7Claura.a.magee%40kcl.ac.uk%7Cd08f0769009b487c9bf508dcb7c9f5b0%7C8370cf1416f34c16b83c724071654356%7C0%7C0%7C638587325242476785%7CUnknown%7CTWFpbGZsb3d8eyJWIjoiMC4wLjAwMDAiLCJQIjoiV2luMzIiLCJBTiI6Ik1haWwiLCJXVCI6Mn0%3D%7C0%7C%7C%7C&sdata=AyaroFgE62Gm1RQ3s1MJCmJVwANikm4orw8AFzeUlBg%3D&reserved=0*](https://eur03.safelinks.protection.outlook.com/?url=https%3A%2F%2Fwww.editorialmanager.com%2Fpmedicine%2Flogin.asp%3Fa%3Dr&data=05%7C02%7Claura.a.magee%40kcl.ac.uk%7Cd08f0769009b487c9bf508dcb7c9f5b0%7C8370cf1416f34c16b83c724071654356%7C0%7C0%7C638587325242476785%7CUnknown%7CTWFpbGZsb3d8eyJWIjoiMC4wLjAwMDAiLCJQIjoiV2luMzIiLCJBTiI6Ik1haWwiLCJXVCI6Mn0%3D%7C0%7C%7C%7C&sdata=AyaroFgE62Gm1RQ3s1MJCmJVwANikm4orw8AFzeUlBg%3D&reserved=0)*). Please contact the publication office if you have any questions.*

This is fine, thank you.
